# The Photochemical Mediated Ring Contraction of 4*H*-1,2,6-Thiadiazines To Afford 1,2,5-Thiadiazol-3(2*H*)-one 1-Oxides

**DOI:** 10.1021/acs.orglett.3c02673

**Published:** 2023-09-11

**Authors:** Emmanouil Broumidis, Christopher G. Thomson, Brendan Gallagher, Lia Sotorríos, Kenneth G. McKendrick, Stuart A. Macgregor, Martin J. Paterson, Janet E. Lovett, Gareth O. Lloyd, Georgina M. Rosair, Andreas S. Kalogirou, Panayiotis A. Koutentis, Filipe Vilela

**Affiliations:** † Institute of Chemical Sciences, School of Engineering & Physical Sciences, Heriot-Watt University, Edinburgh, EH14 4AS, United Kingdom; ‡ SUPA School of Physics and Astronomy and BSRC, University of St Andrews, St. Andrews, KY16 9SS, United Kingdom; § Joseph Banks Laboratories, School of Chemistry, University of Lincoln, Brayford Pool, Lincoln LN6 7TS, United Kingdom; ∥ Department of Life Sciences, School of Sciences, European University Cyprus, 6 Diogenes Str., Engomi, P.O. Box 22006, 1516 Nicosia, Cyprus; ⊥ Department of Chemistry, University of Cyprus, P.O. Box 20537, 1678 Nicosia Cyprus; # Continuum Flow Lab, School of Engineering & Physical Sciences, Heriot-Watt University, Edinburgh, EH14 4AS, United Kingdom

## Abstract

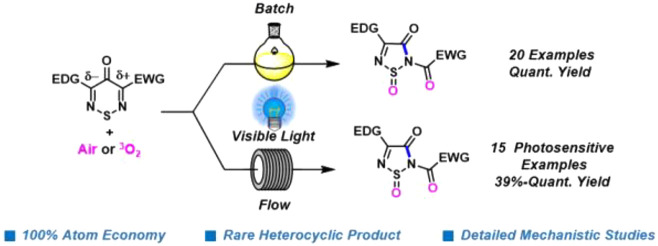

1,2,6-Thiadiazines treated with visible light and ^3^O_2_ under ambient conditions are converted into difficult-to-access 1,2,5-thiadiazole 1-oxides (35 examples, yields of 39–100%). Experimental and theoretical studies reveal that 1,2,6-thiadiazines act as triplet photosensitizers that produce ^1^O_2_ and then undergo a chemoselective [3 + 2] cycloaddition to give an endoperoxide that ring contracts with selective carbon atom excision and complete atom economy. The reaction was optimized under both batch and continuous-flow conditions and is also efficient in green solvents.

1,2,5-Thiadiazole is a privileged motif in medicinal chemistry,^1−3^ and appears in the beta blocker Timolol^4^ that was patented as early as 1968 (Figure 1). The chemistry and applications of 1,2,5-thiadiazoles have been extensively reviewed.^2,3,5,6^ 1,2,5-Thiadiazol-3(2*H*)-one 1-oxides are a rare subclass with ∼15 structures reported in the literature.^6^ Interestingly, in 1982, a Merck patent described 1,2,5-thiadiazol-3(2*H*)-one 1-oxides, *cf.*, compound **1**, as elastase inhibitors for the treatment of diseases like pancreatitis, emphysema, and rheumatoid arthritis (Figure 1).^7a^

**Figure 1:**
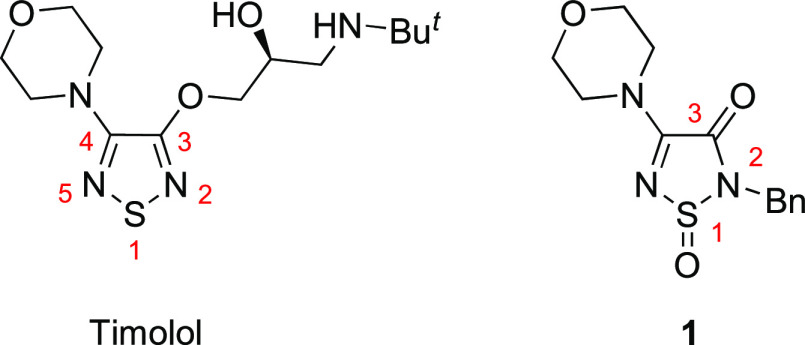
Structures of Timolol and elastase inhibitor **1**.

The known route to these useful 1,2,5-thiadiazol-3(2*H*)-one 1-oxides, which is multistep, can involve toxic thionyl chloride, and suffers from overall low yields initially. 3,4-dialkoxy-1,2,5-thiadiazole 1-oxides are prepared either from dialkyl oxalimidate and SOCl_2_ or via the *m*-CPBA-mediated S-oxidation of 3,4-dialkoxy-1,2,5-thiadiazole; then alkali displacement of the alkoxide affords the thiadiazol-3-ones, which can undergo N2 alkylation or acylation.^7a,7b^ Worthy of note, 1,2,5-thiadiazole 1-oxides are nonaromatic, thus highly electrophilic and thermally unstable.^8^ Therefore, there is a need to discover new mild and practical methods to allow exploration of this previously difficult-to-access chemical space.

Recently, we reported the unexpected ring contraction of spiro(benzo[*d*][1,3]dioxole-2,4′-[1,2,6]thiadiazines) **2** to thiadiazoles **3** in high yields, under thermal and Brønsted or Lewis acid-catalyzed conditions (Scheme 1),^9,10^ In the current work, while investigating 3,5-diphenyl-4*H*-1,2,6-thiadiazin-4-one (**4a**) as a potential photosensitizer for singlet oxygen (^1^O_2_) production, rapid photobleaching of the chromophore led to a new colorless product **5a**. The loss of color indicated reduced conjugation [**5a**: λ_max_(DCM) 296 nm (log ε = 4.26) vs **4a**: λ_max_(DCM) 431 nm (log ε = 3.44)^11^], while ^13^C NMR spectroscopy showed 11 resonances indicating a loss of symmetry, and both MS (ESI^+^) and elemental analysis suggested a molecular formula of C_15_H_10_N_2_O_3_S supporting the addition of two oxygen atoms [see the Supporting Information (SI)]. The structure of the product was determined by X-ray crystallography to be racemic 2-benzoyl-4-phenyl-1,2,5-thiadiazol-3(2*H*)-one 1-oxide (**5a**) (Figure 2).

**Figure 2:**
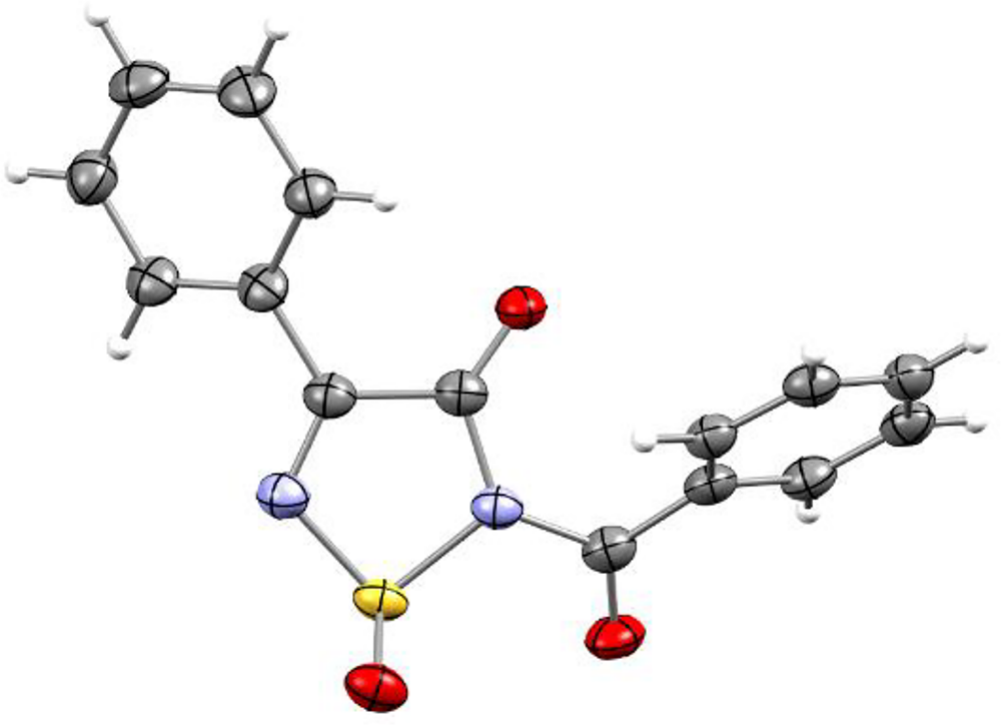
Geometry of 2-benzoyl-4-phenyl-1,2,5-thiadiazol-3(2*H*)-one 1-oxide (**5a**) in the crystal (CCDC No. 2167996). Thermal ellipsoids are at 50% probability.

**Scheme 1:**
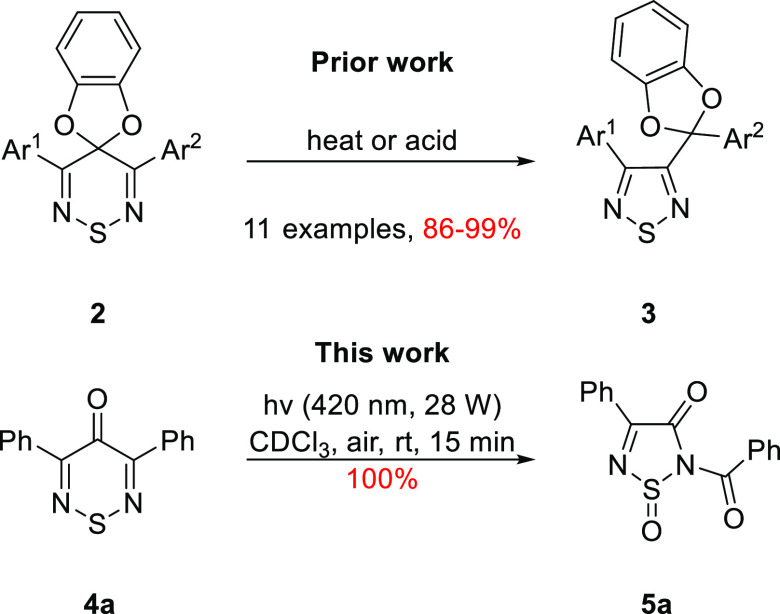
Reported Thermal and Acid Catalyzed Transformation of 1,2,6-Thiadiazines to 1,2,5-Thiadiazoles and Current Photooxidation of 3,5-Diphenyl-4*H*-1,2,6-Thiadiazin-4-one (**4a**)

The remarkable ring contraction presented herein leads to oxidation of an imine motif to an imide and the selective mono-oxidation of the ring sulfur to a stereogenic sulfoxide with only the substrate, solvent, visible light, and air present under ambient conditions.

After our initial discovery, we investigated the conditions to optimize the reaction and gain mechanistic insight. The reaction did not proceed in the absence of light or when irradiated with monochromatic sources that did not overlap with the absorption spectrum of thiadiazine **4a**. Nor did the reaction proceed under a N_2_ atmosphere, indicating that ^3^O_2_ was the source of the additional two oxygen atoms in the resulting thiadiazole **5a**.

The effect of temperature gave the first evidence that ^1^O_2_ was involved in the reaction, which displayed a negative, approximately linear relationship between conversion and temperature (Figure S5 in the SI). The lifetime of ^1^O_2_ in CHCl_3_ can be significantly extended at lower temperatures,^12^ providing more time for the substrate and ^1^O_2_ to react. While lower temperatures were beneficial, the reaction achieved full conversion in <15 min under ambient conditions with irradiation from a 420 nm LED module (28 W). In the interest of operational simplicity, our studies proceeded using these optimized conditions.

Reaction kinetics were screened in various solvents and revealed that the initial rate fitted a first-order kinetic model (see section S2.5 in the SI for details). The reaction rate strongly depended on the solvent environment, and the initial reaction rate in deuterated solvents was about an order of magnitude greater than that in their protonated equivalents (seeFigure S6B in the SI).

The standard reaction conditions were reoptimized under continuous flow (see Figure 3, as well as section SI.4 in the SI). Initially, the “green” solvent dimethyl carbonate was used to optimize the flow system and explore the potential for developing a sustainable process. Optimal process conditions were identified, and thiadiazole **5a** was obtained in >98% yield within 10 min residence time of the flow photoreactor, comparable to the optimized batch conditions in CDCl_3_. Applying the optimized conditions in CDCl_3_ led to the quantitative formation of product **5a** after only 1 min of residence time of the UV-150 reactor coil with a back pressure of 3 bar and using pure ^3^O_2_, a 15-fold reduction in reaction time from the batch protocol. The successful flow synthesis enabled the continuous production of thiadiazole **5a** without concern over light attenuation effects that typically hinder the scale-up of photochemical processes in batch.^13^ From a productivity point-of-view, **5a** was obtained with full conversion and a space-time yield of 199.6 g L^–1^ h^–1^

**Figure 3:**
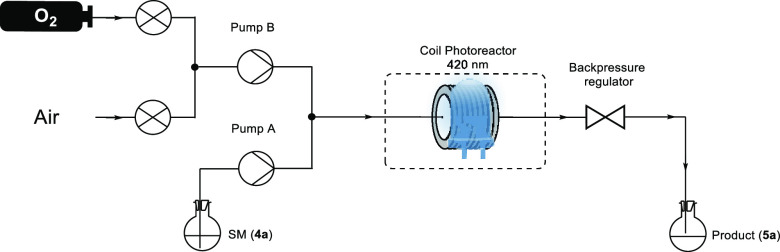
Experimental setup that was used for the reaction under continuous flow conditions.

The reaction conditions were then applied to a range of 3,5-disubstituted 1,2,6-thiadiazines with varying substitution patterns (Table 1). The batch protocol was prioritized for substrate screening, and the flow procedure was reserved for more challenging substrates.

**Table 1:**
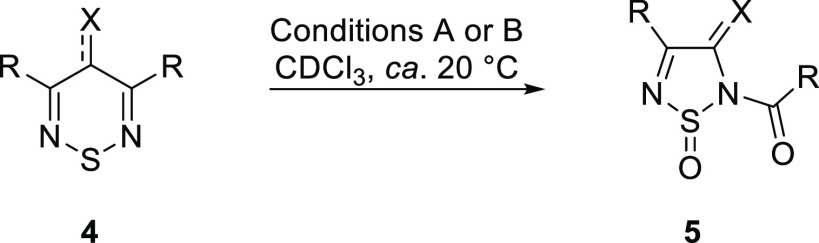
Transformation of 1,2,6-Thiadiazines **4** (0.0375 mmol) into 1,2,5-Thiadiazoles **5** [a]

entry	R	X	time (min)	yield **5** (Cond., %)
1	Ph	O	15	**5a** (A, 100)
2	Ph	O	1	**5a** (B, 100)
3	2-Tol	O	30	**5b** (A, 100)
4	3-Tol	O	2	**5c** (B,^b^ 97)
5	4-Tol	O	15	**5d** (A, 100)
6	2-MeOC_6_H_4_	O	240	**5e** (A, 100)
7	3-MeOC_6_H_4_	O	25	**5f** (A, 100)
8	4-MeOC_6_H_4_	O	15	**5g** (A, 100)
9	2-ClC_6_H_4_	O	120	**5h** (A, 100)
10	3-ClC_6_H_4_	O	25	**5i** (A, 100)
11	4-ClC_6_H_4_	O	15	**5j** (A, 100)
12	4-BnOC_6_H_4_	O	15	**5k** (A, 100)
13	4-MeO_2_CC_6_H_4_	O	15	**5l** (A, 100)
14	thien-2-yl	O	15	**5m** (A, 100)
15	thien-3-yl	O	35	**5n** (A, 100)
16	*N*-Me-pyrrolyl	O	90	**5o** (A, 100)
17	fur-2-yl	O	10	**5p** (A, -c)
18	2-Ph-ethynyl	O	240	**5q** (A, 100)
19	MeO	O	15	**5r** (A, 100)
20	BnO	O	15	**5s** (A, 100)
21	PhO	O	2	**5t** (B,^b^ 93)
22	Ph	(OCH_2_)_2_	10	**5u** (B,^b^ 91)
23	4-Tol	(OCH_2_)_2_	10	**5v** (B,^b^ 94)
24	2-Ph-ethynyl	(OCH_2_)_2_	60	**5w** (B,^b^ 90)
25	4-FC_6_H_4_	(OCH_2_)_2_	60	**5x** (B,^b^ 95)
26	Ph	C_6_H_4_O_2_	10	**5y** (B,^b^ 96)
27	4-Tol	C_6_H_4_O_2_	10	**5z** (B,^b^ 88)

a Condition A: batch, air, *h*ν (420 nm, 28 W); condition B: flow, O_2_, *h*ν (420 nm, 60 W).

b Condition A led to degradation of product.

c Degradation.

The reaction worked well with methyl, methoxy, and chlorophenyl substituents to provide the ring contracted products **5b**–**5j** in quantitative yields, regardless of the substitution pattern. The *m*-tolyl derivative **5c** photodecomposed under batch conditions but was isolated via the flow protocol in 97% yield (Table 1, entry 4). This highlighted the benefit of precise control over irradiation exposure when performing a photochemical process with flow chemistry.^14^

In general, the reaction rates decreased in the order *para* > *meta* ≫ *ortho* based on substitution. Additionally, *para*-substituted benzylether **5k** and methoxycarbonyl **5l** also followed this trend and were isolated in quantitative yield after 15 min of irradiation in batch. As the electronics of the aryl systems vary dramatically between the different substituents and ring positions, steric hindrance is the most probable cause of this effect.

Heteroaryl-substituted thiadiazines reacted smoothly, providing ring-contracted products **5m**–**5o** in a quantitative yield. In contrast, furyl-substituted thiadiazine **5p** decomposed; furans undergo [4 + 2] cycloadditions with ^1^O_2_ to produce endoperoxides that readily ring open to form hydroxy lactones or carbonyl-substituted olefins.^15,16^ Tentatively, the decomposition of the furyl analogue supported our proposed mechanism involving ^1^O_2_.

The reaction also succeeded with alkynylthiadiazine **4q**, which afforded thiadiazole **5q** in a quantitative yield. However, the reaction was relatively sluggish, requiring 4 h of irradiation. We then explored a series of thiadiazines with nonaromatic 3,5-substituents. Alkylethers **4r** and **4s** were quantitatively converted into thiadiazoles **5r** and **5s**, respectively, while phenylether **4t** photodecomposed using the batch protocol but was converted to thiadiazole **5t** in 93% yield under flow.

Amine derivatives were not well-tolerated, with most analogues being either unreactive or rapidly decomposed under both batch and flow conditions; in two cases, the thiadiazine sulfoxide and sulfone products were identified (see section S2.7 in the Supporting Information). Interestingly, such derivatives can be readily prepared from non-S-oxidized thiadiazines using classic oxidants and do not show propensity to ring contract.^17^

Protection of the pre-existing C4 carbonyl with ethylene glycol and catechol acetal derivatives was then explored. In general, all the acetal-protected thiadiazoles **5u**–**5z** displayed reduced photostability: the reversible photoinduced cleavage of acetal-protected benzaldehydes with UV irradiation has been reported,^18^ which may explain the instability of these compounds when overirradiated in batch. Despite our best efforts, we were unable to determine the chemical structures of the photodecomposition products. However, acetal-protected thiadiazoles were isolated in ∼90% yield using the flow protocol. No significant differences in stability or reactivity between ethylene glycol and catechol acetals were observed.

The ring contraction of the acetal derivatives supported that (i) the additional oxygen atoms installed via the reaction end up on the ring sulfur and external amide carbonyl; (ii) the C4 carbonyl does not migrate; and, (iii) the cleavage of the C–C bond during the ring contraction does not depend on Norrish-type radical processes induced by direct excitation of the carbonyl.^19^

To assess the selectivity preferences for the reaction, with respect to the excised carbon, we screened a series of asymmetric thiadiazines **4aa**–**4ak** with different R-groups substituted at the 3- and 5-ring positions (see Table 2).

**Table 2:**
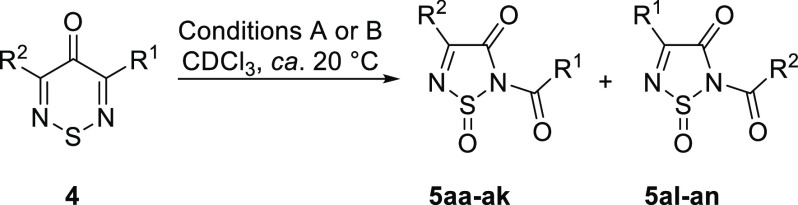
Transformation of Asymmetrical 1,2,6-Thiadiazines **4** (0.0375 mmol) into 1,2,5-Thiadiazoles **5**^a^

entry	R^1^	R^2^	time (min)	yield **5**(Cond., %)
1	4-MeO_2_CC_6_H_4_	4-MeOC_6_H_4_	10	**5aa** (A, 100)
2	Ph	MeO	15	**5ab** (A, 100)
3	Ph	PhO	15	**5ac** (A, –b)
4	Ph	PhO	5	**5ac** (B, 39)
5	3-O_2_NC_6_H_4_	MeO	1440	**5ad** (A, –b)
6	3-O_2_NC_6_H_4_	MeO	5	**5ad** (B, 82)
7	4-O_2_Nthien-2-yl	thien-2-yl	60	**5ae** (A, 100)
8	Ph	PhNH	5	**5af** (B, 94)
9	PhO	PhNH	2	**5ag** (B, 86)
10	PhS	PhNH	2	**5ah** (B, 91)
11	4-Tol	4-MeOC_6_H_4_	15	**5ai/5al** (A,^c^ 100)
12	2-MeOC_6_H_4_	3-MeOC_6_H_4_	10	**5aj/5am** (A,^d^ 100)
13	4-MeOC_6_H_4_	2-MeOC_6_H_4_	10	**5ak/5an** (A,^e^ 100)

a Cond. A: Batch, air, hv (420 nm, 28 W), Cond. B: flow, O_2_, hv (420 nm, 60 W).

b Degradation.

c Inseparable mixture of **5ai** and **5al** (**5ai**/**5al**, 69:31).

d Inseparable mixture of **5aj** and **5am** (**5aj**/**5am**, 60:40).

e Inseparable mixture of **5ak** and **5an** (**5ak**/**5an** 60:40).

Pleasingly, most asymmetric thiadiazines showed excellent chemoselectivity, and a single product was isolated. Bisaryl thiadiazine **4aa** displayed accelerated reactivity and complete selectivity for excising the carbon bound to the more electron deficient *para*-(methoxycarbonyl)phenyl, as shown by X-ray crystallography (Figure 4).

**Figure 4:**
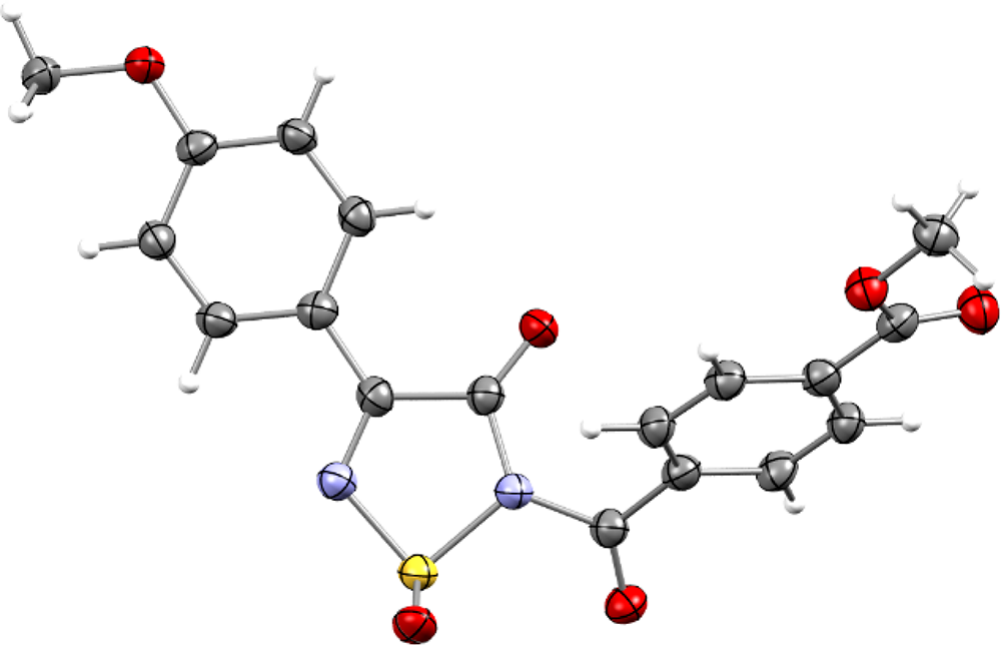
Geometry of methyl 4-[4-(4-methoxyphenyl)-1-oxido-3-oxo-2,3-dihydro-1,2,5-thiadiazole-2-carbonyl]-benzoate (**5aa**) in the crystal (CCDC: 2168224). Thermal ellipsoids at 50% probability.

Thiadiazines **4ab**–**4ad**, bearing a 3-ether and 5-phenyl substituent, also reacted selectively by excising the phenyl bearing carbon atom. Reaction of thiadiazine **4ae** bearing nitrothienyl and thienyl substituents was slower (1 h) and again showed complete selectivity for excising the carbon bound to the more electron deficient hetarene. In contrast to the symmetric amino thiadiazines, asymmetric secondary monoaminothiadiazines **4af**–**4ah** gave the expected thiadiazoles. In all three cases, the nonamine bound carbon was selectively excised and single thiadiazoles **5af**–**5ah** were respectively obtained in high yields.

A set of three asymmetric 3,5-bisphenylthiadiazines **4ai**–**4ak**, bearing ring substituents with similar electron-donating character, led to the formation of mixtures of both potential ring contracted products. In the case of 3-(4-methoxyphenyl)-5-(4-tolyl)thiadiazine **4ai**, the tolyl bound carbon was preferentially excised, leading to a 69:31 mixture of thiadiazoles **5ai** and **5al**, respectively. Asymmetric methoxyphenyl-substituted thiadiazines **4aj** and **4ak** allowed a comparison of the preference of carbon excision with respect to the *ortho*, *meta*, and *para* position of the methoxy group. Interestingly, in the unsymmetrical *ortho*/*meta*-methoxyphenylthiadiazine **4aj** we observed only a slight preference for the contraction at the 2-methoxyphenyl bound carbon. In *ortho*/*para*-methoxyphenyl-thiadiazine **4ak** we also observed a slight preference but this time for the contraction at the 4-methoxyphenyl bound carbon, leading to 60:40 mixtures of thiadiazoles **5aj/5am** and **5ak/5an**, respectively.

To investigate the reaction mechanism, control reactions were performed with ROS traps to help confirm the presence of ^1^O_2_ under the reaction conditions (see section S2.2 in the Supporting Information). Notably, in the presence of ^1^O_2_ traps such as α-terpinene and Ph_3_P, full conversion of thiadiazine **4a** to thiadiazole **5a** required significantly longer reaction times (1 and 2 h, respectively) and the expected characteristic ^1^O_2_-trapped adducts, ascaridole, and Ph_3_PO, were observed.^13,20,21^ Performing the reaction in the absence of irradiation and with dark ^1^O_2_ generator systems also enabled the conversion to thiadiazole **5a**, indicating that the reaction mechanism was not reliant on a photochemical rearrangement of thiadiazine **4a** or its electronic excited states. The reaction was also performed in the presence of Methylene Blue, a known ^1^O_2_ photosensitizer, which has a visible light absorption spectrum that is chromatically orthogonal to that of substrate **4a** (see Figure S7B in the Supporting Information). With 1 mol % loading of the orthogonal photosensitizer and red-light irradiation (620 nm), under otherwise identical conditions, full conversion to thiadiazole **5a** was achieved within 1 h. This further suggested that ^1^O_2_ could independently drive the reaction and enabled the reaction to be performed with longer irradiation wavelengths, which avoided the excitation of more complex substrates containing chromophores. Further experiments ruled out the involvement of superoxide in the reaction mechanism, as well as the ^1^O_2_ mediated sulfide oxidation to sulfoxide (see section S2.2 in the Supporting Information).

On the basis of the above observations, we propose that irradiation of thiadiazine **4** generates excited 4* that undergoes energy transfer to ^3^O_2_ to generate ^1^O_2_, while returning to the ground state (see Figure 5a). The reaction of **4a** with ^1^O_2_ was then modeled computationally at the CASPT2/6-31G**//UB3LYP/6-31G** level of theory (see Figure 5b). This suggests the initial formation of an endoperoxide intermediate, **Int(4a–5a)**, at +7.4 kcal/mol that proceeds via a concerted [3 + 2] addition of ^1^O_2_ across the {(Ph)C^3^–N^2^=S^1^} moiety (**TS(4a-5a)1**, + 9.6 kcal/mol). A [3 + 2] cycloaddition involving ^1^O_2_ has been suggested before, albeit lacking the necessary data for verification.^22^ From **Int(4a-5a)** O–O bond cleavage occurs with concomitant ring contraction in a single step via **TS(4a-5a)2** at +21.7 kcal/mol. This entails the simultaneous cleavage of the C^3^–C^4^ bond and the formation of a new N^2^–C^4^ amide bond. The formation of thiadiazole **5a** is highly exergonic (Δ*G* = −85.7 kcal/mol), and the overall barrier of 21.7 kcal/mol is consistent with the room temperature reactivity observed experimentally. **TS(4a-5a)2** is both the rate- and selectivity-determining transition state.

**Figure 5:**
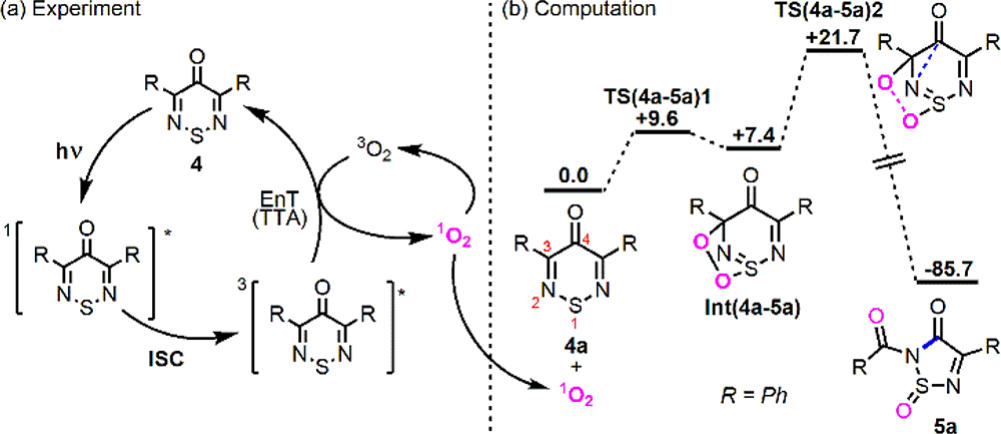
(a) Proposed mechanism for the formation of ^1^O_2_ upon irradiation of thiadiazines, **4**. (b) Computed free-energy profile (CASPT2(16,13)/6-31G**//UB3LYP(CHCl_3_)/6-31G**; kcal/mol) for the reaction of ^1^O_2_ with **4a** to form **5a**.

Worthy of note, the discovery of a [3 + 2] cycloaddition of ^1^O_2_ advances its known chemistry, and this reactivity can, potentially, be more broadly developed. ^1^O_2_ can selectively oxidize unsaturated hydrocarbons and electron-rich heteroatoms, and readily undergoes pericyclic reactions with olefins and diene substrates.^23^ This reactivity has been used to synthesize various complex natural products and active pharmaceutical ingredients,^24^ including the semisynthesis of artemisinin, a crucial antimalarial drug.^25^ New reports mainly focus on the known ^1^O_2_ reactivity toward complex synthetic targets.^24,26^

In conclusion, we report the photochemically mediated ring contraction of 1,2,6-thiadiazines under ambient aerobic conditions to provide a sustainable and atom economic route to elusive 1,2,5-thiadiazol-3(2*H*)-one 1-oxides in generally quantitative yields and chromatography-free purification. To the best of our knowledge, the reaction proceeds via the first example of a ^1^O_2_ [3 + 2] cycloaddition, expanding its known chemistry.

## Data Availability

The data underlying this study are available in the published article and its Supporting Information.

## Supporting Information Available

Experimental section, optimization, mechanistic and kinetic studies, computational studies, X-ray crystallography data for **4a**, **4m**, **5a**, **5b**, **5i**, **5l**, **5m**, **5n**, **5u**, **5x**, **5aa**, **5ac**, **5af**, **5ah**, **17a**, **17b**, **18a**, **18b**, **23**, and **27**, and NMR spectra of all new compounds (PDF)
