# Correction to “The Photochemical Mediated Ring Contraction of 4*H*‑1,2,6-Thiadiazines To Afford 1,2,5-Thiadiazol-3(2*H*)‑one 1‑Oxides”

**DOI:** 10.1021/acs.orglett.5c02589

**Published:** 2025-09-17

**Authors:** Emmanouil Broumidis, Christopher G. Thomson, Brendan Gallagher, Lia Sotorríos, Kenneth G. McKendrick, Stuart A. Macgregor, Martin J. Paterson, Janet E. Lovett, Gareth O. Lloyd, Georgina M. Rosair, Andreas S. Kalogirou, Panayiotis A. Koutentis, Filipe Vilela

There was a formatting error during uploading of the original Supporting Information document, which caused the NMR spectra at the end of the document to appear cropped. This new version addresses that.

## Supplementary Material



## Notes

Experimental section, optimization, mechanistic and kinetic studies, computational studies, X-ray crystallography data for **4a**, **4m**, **5a**, **5b**, **5i**, **5l**, **5m**, **5n**, **5u**, **5x**, **5aa**, **5ac**, **5af**, **5ah**, **17a**, **17b**, **18a**, **18b**, **23**, and **27**, and NMR spectra of all new compounds (PDF)

