# Supplementary material for: The Photochemical Mediated Ring Contraction of 4H-1,2,6-Thiadiazines To Afford 1,2,5-Thiadiazol-3(2H)-one 1-Oxides
Source: Org Lett. 2023 Sep 11;25(37):6907–12. doi: 10.1021/acs.orglett.3c02673 (PMC10521009; doi:10.1021/acs.orglett.3c02673)

Supporting Information

The Photochemical Mediated Ring Contraction of 4*H*-1,2,6-Thiadiazines to Afford 1,2,5-Thiadiazol-3(2*H*)-one 1-Oxides

Emmanouil Broumidis,[a] Christopher G. Thomson,[a] Brendan Gallagher,[a] Lia Sotorríos,[a] Kenneth G. McKendrick,[a] Stuart A. Macgregor,[a,*] Martin J. Paterson,[a] Janet E. Lovett,[b] Gareth O. Lloyd,[c] Georgina M. Rosair,[a] Andreas S. Kalogirou,[d,e] Panayiotis A. Koutentis,[e,*] and Filipe Vilela[a,f,*]

[a] Institute of Chemical Sciences, School of Engineering & Physical Sciences, Heriot-Watt University, Edinburgh, EH14 4AS, UK.

[b] SUPA School of Physics and Astronomy and BSRC, University of St Andrews, St Andrews, KY16 9SS, UK.

[c] Joseph Banks Laboratories, School of Chemistry, University of Lincoln, Brayford Pool, Lincoln, LN6 7TS, UK.

[d] Department of Life Sciences, School of Sciences, European University Cyprus, 6 Diogenes Str., Engomi, P. O. Box 22006, 1516 Nicosia, Cyprus.

[e] Department of Chemistry, University of Cyprus, P. O. Box 20537, 1678 Nicosia, Cyprus.

[f] Continuum Flow Lab, School of Engineering & Physical Sciences, Heriot-Watt University, Edinburgh, EH14 4AS, UK.

- Correspondence: F.Vilela@hw.ac.uk; S.A.Macgregor@hw.ac.uk; koutenti@ucy.ac.cy

Table of Contents

S1 Experimental Section S3
S1.1 General methods and materials S3
S1.2 Experimental setup (Method A and Method B) S4
S1.3 Reaction optimization under batch conditions (Method A) S5
S1.4 Reaction optimization under continuous flow conditions (Method B) S5
S2 Details of Experimental Mechanistic and Kinetic Studies S8
S2.1 Optimization and Mechanistic Studies S8
S2.2 Trapping experiments S10
S2.3 Concurrent ring contraction of two symmetrical 1,2,6-thiadiazines S13
S2.4 Equivalents of O_2 needed for the complete transformation of **4a** to **5a** S15
S2.5 Kinetic analysis S15
S2.6 Proposed kinetic mechanism S22
S2.7 Limitations of the scope S22
S3 Computational Studies S26
S3.1 Methodology S26
S3.2 Computed structures (Å) and energies (a.u.) S29
S4 Synthesis and Characterization S38
S4.1 Preparation of 4*H*-1,2,6-thiadiazine starting materials S38
S4.1.1 Preparation of symmetrical 3,5-diaryl-4*H*-1,2,6-thiadiazinones S38
S4.1.2 Preparation of asymmetrical 3,5-diaryl-4*H*-1,2,6-thiadiazinones S40
S4.2 Preparation of 1,2,5-thiadiazole *S*-oxides from 4*H*-1,2,6-thiadiazines S44
S4.2.1 Symmetric derivatives S44
S4.2.2 Asymmetric derivatives S52
S4.3 Characterization of isolated side-products and reference compounds S57
S5 Single Crystal X-ray Diffraction Data Collection and Refinement Details and Discussion S60
S6 References S79
S7 ^{1}H and ^{13}C NMR Spectra of New Compounds S103

S1 Experimental Section

S1.1 General methods and materials

Chemicals used were commercially available except those whose synthesis is described herein. Anhydrous $MgSO_4$ was used for drying organic extracts and all volatiles were removed under reduced pressure. Reaction mixtures and column eluents were monitored by TLC using commercial aluminium backed thin layer chromatography (TLC) plates (Merck Kieselgel 60 F_{254}); the plates were observed under UV light at 254 and 365 nm. The technique of flash chromatography was used throughout for all non-TLC scale chromatographic separations using Merck Silica Gel 60 (< 0.063 mm). Melting points were determined using a Stuart SMP10 digital melting point apparatus or a PolyTherm-A, Wagner & Munz, Kofler Hotstage Microscope apparatus. Small scale (μL) liquid handling measurements were made using variable volume (10‑100 μL) single channel Gilson PIPETMAN precision micropipettes. Solvents used for recrystallization are indicated after the melting point. UV-vis spectra were obtained using a Perkin-Elmer Lambda-25 UV/vis spectrophotometer and inflections are identified by the abbreviation "inf". IR spectra were recorded on a Thermo Scientific Nicolet iS5 FTIR spectrometer with iD5 ATR accessory or a Shimadzu FTIR-NIR Prestige-21 spectrometer with Pike Miracle Ge ATR accessory and broad, strong, medium and weak peaks are represented by br, s, m and w, respectively. ^{1}H and ^{13}C NMR spectra were recorded on either a Bruker AVANCE III HD machine (at 400 and 100 MHz, respectively), a Bruker Avance 300 (at 300 and 75 MHz, respectively), or a 500 machine (at 500 and 125 MHz, respectively). Chemical shifts (δ) are expressed in ppm and coupling constants *J* are given in Hz. Data are represented as follows: chemical shift, multiplicity (s singlet, d doublet, t triplet, q quartet, m multiplet and/or multiple resonances, br s broad singlet). Deuterated solvents were used for homonuclear lock and the signals are referenced to the deuterated solvent peaks. For the acquisition of mass spectra the samples were prepared as detailed below and analyzed by positive ion nanoelectrospray (nES) using a Thermo Scientific™ LTQ Orbitrap XL™ ETD Hybrid Ion Trap-Orbitrap Mass Spectrometer. Matrix-Assisted Laser Desorption/Ionization-Time Of Flight (MALDI-TOF) mass spectra (+ve mode) were recorded on a Bruker Autoflex III Smartbeam instrument (Bruker), ESI-APCI+ mass spectra were recorded on a Model 6110 Quadrupole MSD, Agilent Technologies and ES-API spectra on a Model 1260 Infinity II Quadrupole MSD, Agilent Technologies. Flow reactions were carried out with a commercial E-series Photochem UV-150 reactor by Vapourtec Ltd, with two V-3 peristaltic pumps and using an LED module emitting at 420 nm with 61 W light output and a light intensity of 10.2 $W \cdot cm^{-2}$. For light driven batch reactions, a commercially available LED module was used, emitting at 420 nm/620 nm with 28 W light output and a light intensity of 3.5 $W \cdot cm^{-2}$ (OSA Opto lights, OLM-018 series, Berlin, Germany), placed at a distance of 5 cm from the reaction vessel. For the collection of LC-MS data a Shimadzu LC-2040C 3D Plus instrument was used. EPR studies were carried out using an ActiveSpectrum microESR instrument, operating at X-band (9.8 GHz) frequency. Sample tubes were Blaubrand micropipettes. Cristaseal putty was used for sealing. Acquisition parameters were the following: Microwave power, 10 mW; Sweep field range, 3333‑3657 G; Sweep time, 14.9 s; Number of points, 2592; Number of sweeps, 5; Temperature, 26.7 °C. Elemental analysis was carried out at London Metropolitan University, with a Thermo Scientific™ FLASH 2000 CHNS Analyzer. For structure determination using single crystal X-ray diffraction samples were mounted on a Molecular Dimensions Litholoop in mineral oil. Data was collected on a Bruker D8 Venture diffractometer using either a copper or molybdenum microsource sealed tube with triumph monochromator and the Apex3

suite of programs. The crystal was kept at 100.0 K during data collection. Using Olex2,[1] the structure was solved with the SHELXT structure solution program using Intrinsic Phasing and refined with the SHELXL refinement package using Least Squares minimisation.[2]

S1.2 Experimental setup (Method A and Method B)

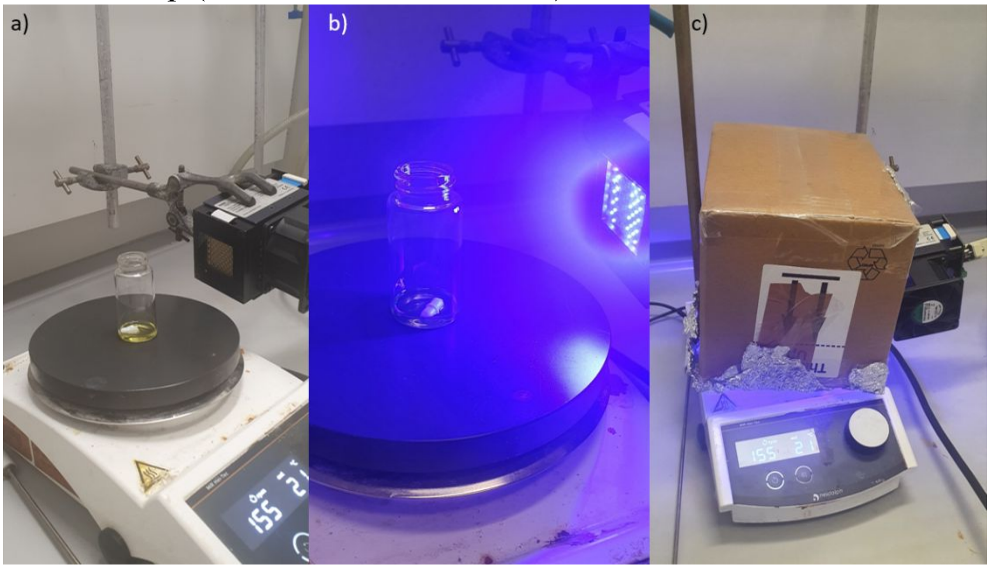

Figure S1. Photographs of batch setup (Method A). a) A 5 mL vial charged with 1,2,6-thiadiazine SM dissolved in $CDCl_3$ (1 mL). The lid is not used to allow atmospheric O_2 in the vial. b) Reaction underway. c) A cardboard box lined with aluminium foil is placed on top of the hotplate to limit photon leakage and ensure the safety of the operator.

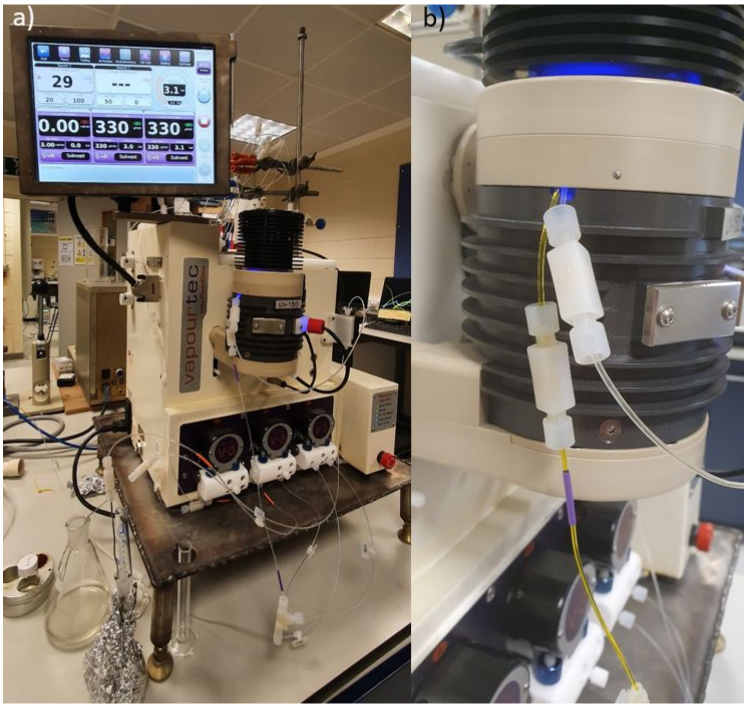

Figure S2. Photographs of the flow reaction setup (Method B). a) Vapourtec E-series equipped with the Photochem UV-150 module. b) Close-up photograph of the UV-150 reactor during operation. The tube on the left containing the yellow-colored solution is flowing towards the reactor. The tube of the right is the outlet of the reactor, and as it can be seen, the fluid is colorless, indicating that the reaction is complete.

S1.3 Reaction optimization under batch conditions (Method A)

Following the initial discovery of the ring contraction of thiadiazine **4a**, a brief optimization screen of the effects of temperature, irradiation wavelength and the exclusion of oxygen was performed before carrying out more detailed mechanistic and kinetic studies, discussed in Section S2. The results are displayed below in Table S1, and are discussed in the manuscript.

Table S1. Parameter optimization for the ring contraction of thiadiazine **4a** to thiadiazole **5a**.[a]

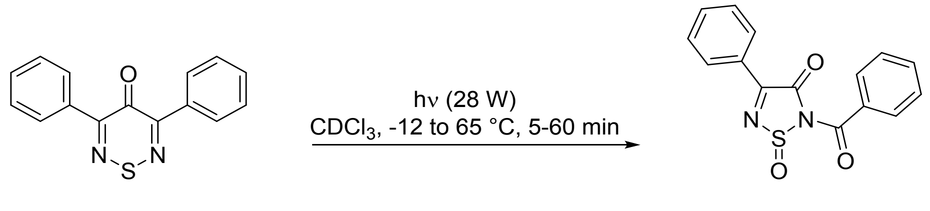

4a **5a**

Entry	Temp. (° C)	Light (nm)	Time (min)	Conversion (%)[b]
**1**	20	dark	60	0
**2**	20	390	30	100
**3**	20	420	15	100
**4**	20	520	60	0
**5**	20	620	60	0
**6**	-12	420	5	81
**7**	0	420	5	73
**8**	20	420	5	56
**9**	40	420	5	41
**10**	65	420	5	8
**11**[c]	20	420	60	0

[a] Conditions: **4a** (0.375 μmol) dissolved in $CDCl_3$ (1 mL); [b] Determined by ^1H NMR; [c] Under N_2 atmosphere.

S1.4 Reaction optimization under continuous flow conditions (Method B)

To optimize the reaction under continuous flow conditions, we used a commercial Vapourtec Ltd E-series flow chemistry system equipped with their UV-150 photochemical reactor (420 nm LED module, 60 W). The UV-150 reactor consist of an LED array placed in the center of a coil of transparent perfluoroalkoxy alkane (PFA) tubing (1 mm ID). The total internal volume of the coil reactor was 3.33 mL. The flow system was setup as illustrated in Figure S3. Two peristaltic pumps were used: the first one (pump A) pumped a solution of **4a** (C = 37.5 mM) in 100 mL of dimethyl carbonate (DMC). The second pump (pump B), was used to introduce either air from the surrounding atmosphere or pure O_2 gas from a cylinder.

The two flowing streams were then mixed at a T-junction, creating a gas-liquid slug-flow regime which enters the irradiated coil reactor. The residence time of reaction mixture was varied by synchronously altering the flow rates of pumps A + B. The backpressure of the flow system was controlled using a back-pressure regulator module (BPR), which enabled the system to be pressurized between 0-10 bar. It was observed that when the pressure was raised above 2 bar, the flow regime changed from a heterogeneous slug-flow to a homogeneous expanded liquid phase. Finally, the fluid was collected in a 10 mL round bottom flask. Both the input and receiver flasks were covered in aluminium foil for the duration of the experiments to prevent background

conversion from external ambient light sources. The performance of each run was evaluated by subjecting the contents of the collection flask to HPLC analysis.

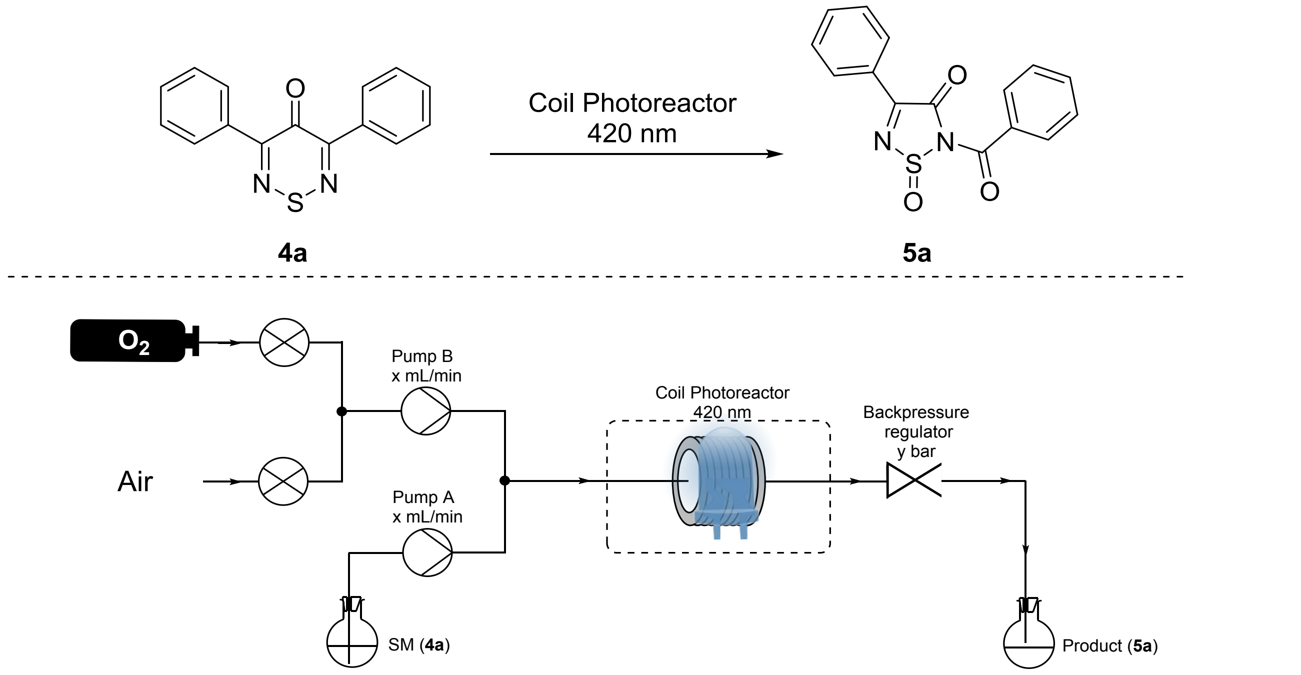

Figure S3. Diagram showing the experimental set-up that was used for the optimization of the ring contraction under continuous flow conditions.

The flow system was evaluated at four residence times under various conditions of pressure, O_2 source and solvent, the results of which are displayed in Figure S4. Using O_2 directly from a cylinder in place of air at ambient pressure did provide an increase in conversion across all residence times, although there were diminishing returns as residence times increase. For example, at 0.5 min residence time, conversion under O_2 was enhanced by 7.5% with respect to air but at 10 min the increase was only 2.2%. Similarly, increasing back-pressure of the O_2 flow system from 0-3 bar also increased conversion, by 16.4% at 0.5 min residence time. However, back-pressure also suffered from diminishing returns, as increasing pressure from 3 to 7 bar only yielded a 1.3% increase in conversion at 0.5 min residence time.

Ultimately, the screening showed that conversion in DMC solvent could be significantly enhanced through a combination of applied back pressure, residence time and using O_2 as the oxygen source, achieving a maximum conversion of 98.2% after 10 min of reactor residence at 5 bar. Although 5 bar provided the maximum conversion, we selected 3 bar as the optimal back-pressure as the difference in conversion was only -0.1% and it was far easier to maintain this back-pressure within the system.

Finally, we tested the optimized back-pressure at the four residence times using $CDCl_3$ as this solvent provides the fastest kinetics of all the solvents screened in batch (see Section S2.4). Unsurprisingly, the system was significantly enhanced when using $CDCl_3$ as solvent and achieved near-full conversion (99.2%) after 0.5 min residence time with pure O_2 gas and 3 bar of back-pressure. Increasing the residence time to 1 min achieved full conversion of the starting material, and the ring contracted product **5a** was isolated in quantitative yield. In the interest of minimizing the irradiation exposure of starting materials and products during the flow synthesis, we

elected to use $CDCl_3$, O_2 supplied by a cylinder, and a back-pressure of 3 bar for our optimized conditions in the substrate scope, referred to as Method B within the manuscript.

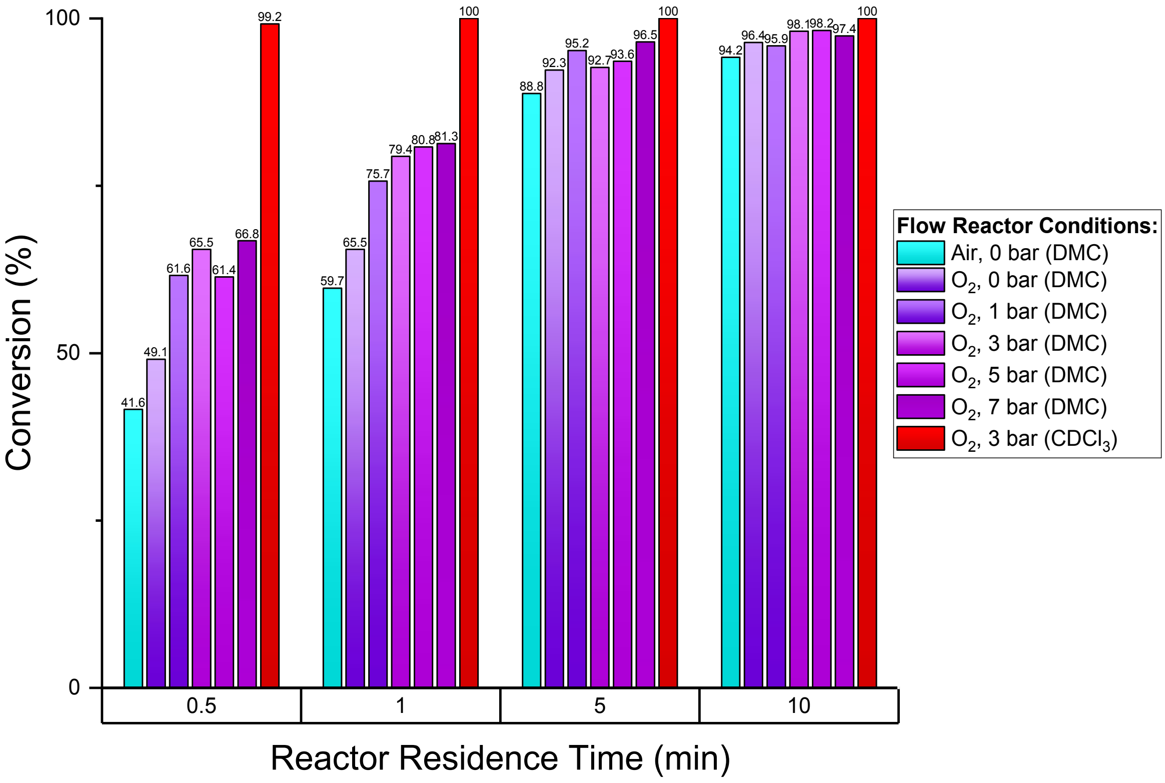

Figure S4. Results of the flow optimization studies. DMC: dimethyl carbonate.

S2 Details of Experimental Mechanistic and Kinetic Studies

S2.1 Optimization and Mechanistic Studies

Following our initial discovery, we investigated the effects of various conditions to optimize the reaction and provide mechanistic insight. A series of control experiments confirmed that the reaction did not proceed in the absence of light or when irradiating with monochromatic light sources that did not overlap with the absorption spectrum of thiadiazine **4a**. Additionally, the reaction did not proceed under a N_2 atmosphere, confirming that $^3O_2$ was required and the source of the additional two oxygen atoms on the resulting thiadiazole **5a**. The reaction was repeated under an inert atmosphere while injecting varying volumes of $^3O_2$ to the reaction vessel, which gave a clear linear relationship with a gradient of ~1, indicating that the reaction proceeds via one equivalent of thiadiazine **4a** reacting with a single molecule of $^3O_2$ and providing 100% atom economy (see Section S2.4).

The effect of temperature provided the first evidence that $^1O_2$ may be involved in the reaction: the reaction displayed a negative, approximately linear relationship between conversion and temperature (Figure S5). The lifetime of $^1O_2$ in $CHCl_3$ can be significantly extended at reduced temperatures,[3] providing more time for the substrate and $^1O_2$ to react and rationalizing our experimental observations. Although reduced temperatures were beneficial, the reaction achieved full conversion in < 15 min under ambient conditions with irradiation from a 420 nm LED module (28 W), and in the interest of operational simplicity, we proceeded with our investigation using these optimized conditions.

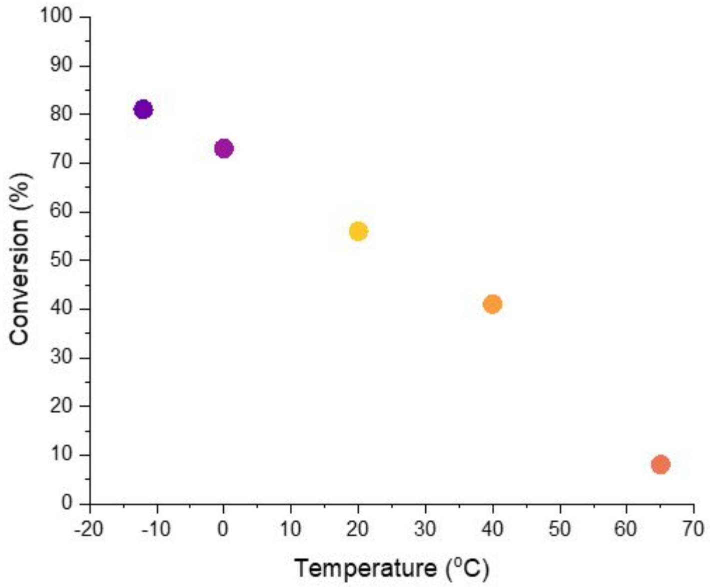

Figure S5. Plot of conversion to thiadiazole **5a** *vs* reaction temperature.

The reaction kinetics were then screened in a variety of solvents, as the lifetime of $^1O_2$ strongly depends on the solvent environment.[4,5] Specifically, we compared the reaction rate between protonated and deuterated solvents as the latter prolong the lifetime of $^1O_2$ by reducing vibronic energy transfer between $^1O_2$ and the solvent, which consequently reduces non-radiative decay of $^1O_2$.[6,7] The full kinetic profile of the reaction was qualitatively similar for all solvents. The profiles did not perfectly fit either a 1st or 2nd order kinetic model as they transitioned between different kinetic regimes after achieving a certain conversion of thiadiazine **4a** to thiadiazole **5a**,

consistent with our proposed mechanism (*vide infra*). Hence, the initial rate of reaction, between approximately 0-70% conversion, which fits relatively well to a 1st order kinetic model was used to compare the various solvents empirically.

As anticipated, the reaction rate displayed a strong dependence on solvent environment and the initial reaction rate in deuterated solvents was generally an order of magnitude greater than their protonated equivalent (Figure S6A). Dimethyl carbonate was trialed as a green solvent alternative and provided similar kinetics to ethyl acetate and dichloromethane (DCM). Although dimethyl carbonate does not provide the most efficient reaction kinetics, it is a sustainable solvent alternative that was compatible with the reaction and could be enhanced through flow chemistry (*vide infra*).[8] The observed initial rate constant for the reaction was plotted against literature values for the lifetime of $^1O_2$ in the various solvents, revealing a strong positive correlation (Figure S6B). We suggest this is compelling evidence for the involvement of $^1O_2$ in the reaction. Dimethyl sulfoxide (DMSO) displayed significantly slower kinetics, with an observed initial rate constant that was two orders of magnitude lower than other non-deuterated solvents. As well as DMSO providing the lowest $^1O_2$ lifetime (5.5 μs), it is also known that DMSO can react with $^1O_2$ to form dimethyl sulfone, providing a competitive process that removes $^1O_2$ and rationalizes the significant decrease in reaction rate - providing further evidence of the involvement of $^1O_2$.[9]

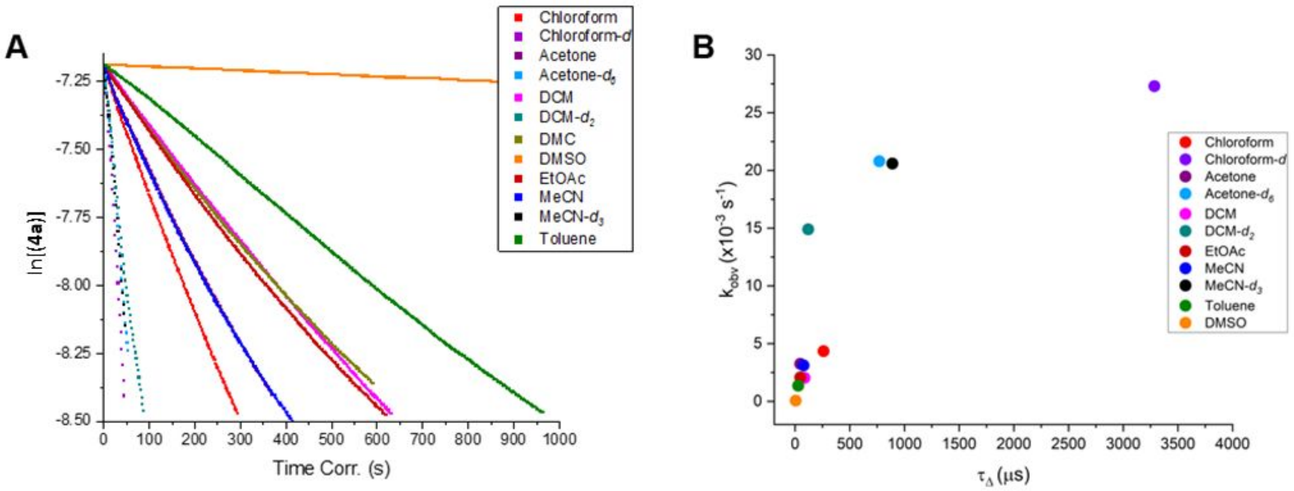

Figure S6. A) Kinetic data of the conversion of thiadiazine **4a** under irradiation in various solvents, fit to a 1st order kinetic model. The conversion was monitored by UV-Vis, measuring the absorbance at 400 nm with continuous sampling (see section S2.5). B) Plot of the observed 1st order rate constants in each of the solvents vs literature values for the lifetime of singlet oxygen in that solvent (τ_{Δ}).

The reaction’s kinetic profile was also investigated while varying some additional parameters (Figure S7A): the reaction rate was enhanced when performed under an atmosphere of pure $^3O_2$ and reduced when the intensity of irradiance was reduced (1 *vs* 2 LED modules). Additionally, only the initial rate, and not the observed 1st order rate constant, was significantly affected when the initial concentration of thiadiazine **4a** was reduced by 60% (0.25 *vs* 0.1 mg/mL). The details of these experiments and extracted rate constants are presented section S2.5. Following this, we performed a series of experiments with ROS traps to help confirm the presence of $^1O_2$ (see section S2.2). Notably, in the presence of $^1O_2$ traps such as α-terpinene and Ph_3P, full conversion of thiadiazine **4a** to thiadiazole **5a** required significantly longer reaction times (1 and 2 h, respectively) and the expected characteristic $^1O_2$-trapped adducts, ascaridole and Ph_3PO, were observed.[10-12] Performing the reaction in the absence of irradiation and with dark $^1O_2$ generator systems also enabled the conversion to thiadiazole **5a**,

indicating that the reaction mechanism was not reliant on a photochemical rearrangement of thiadiazine **4a** or its electronic excited states. Finally, the reaction was performed in the presence of a known $^1O_2$ photosensitizer, methylene blue, which has a visible light absorption spectrum that is chromatically orthogonal to that of the substrate **4a** (Figure S7B). With 1 mol% loading of the orthogonal photosensitizer and red-light irradiation (620 nm), under otherwise identical conditions, full conversion to thiadiazole **5a** was achieved within 1 h.

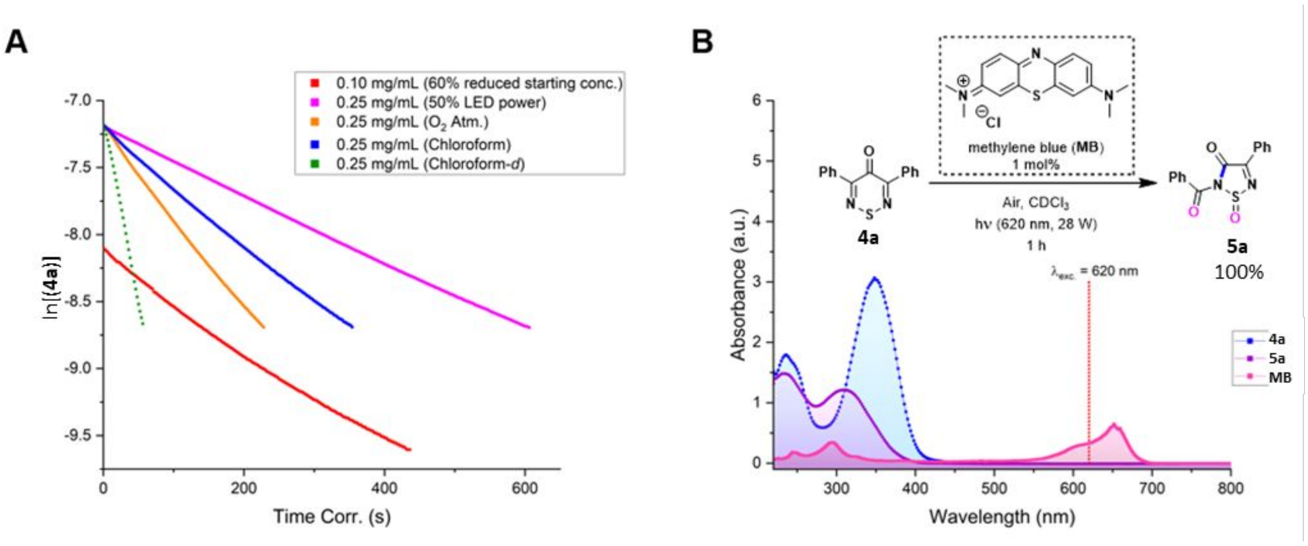

Figure S7. A) First order kinetic models for the conversion of **4a** under various conditions, monitored by UV-Vis measurement of the absorbance at 400 nm with continuous sampling. The blue trace corresponds to standard conditions under air. B) Reaction scheme for the conversion of **4a** to **5a** using an orthogonal photosensitizer, methylene blue chloride (MB). The reaction scheme is superimposed over UV-Vis. spectra for **4a**, **5a** and MB, displaying that the visible light absorbances of the three compounds are orthogonal. The red dashed line indicates the excitation wavelength of maximum intensity for the monochromatic LED module used in the reaction.

S2.2 Trapping experiments

To gain insight into the underlying mechanism that drives the ring contraction, a series of control and intermediate trapping studies were conducted (Table S2). Use of TEMPO as an additive (Table S2, entry 1) led to a longer reaction requiring 45 min for completion instead of 15, which implies the potential involvement of radical species. DABCO and Ph_3P (Table S2, entries 2 & 3) were used as selective $^1O_2$ traps and both stalled the reaction; the former completely inhibited the formation of thiadiazole **5a**, while the latter increased the reaction time to 120 min with concomitant production of Ph_3PO, which was detected by ^{31}P NMR. These results, along with the impact of deuterated solvents on the reaction times offer compelling evidence that $^1O_2$ is involved in the ring contraction. The addition of *t*-BuOH (Table S2, entry 4), a hydroxyl radical ($OH^{·-}$) trap, did not affect the reaction time or the conversion percentage, and thus hydroxyl radical participation was excluded from the mechanism. We then used 1,4-benzoquinone (BQ), a well-known $O_2^{·-}$ trap (Table S2, entry 5), which slowed the reaction time to 45 min but the expected trapping product, hydroquinone, was only detected in trace quantities. This indicates that the reaction was inhibited by benzoquinone not through the trapping of $O_2^{·-}$, but potentially due to additional light attenuation, increased concentration of the reaction solution or quenching the excited state of thiadiazine **4a**.

Table S2. Control experiments for the ring contraction of thiadiazine **4a** to thiadiazole **5a** (Method A).

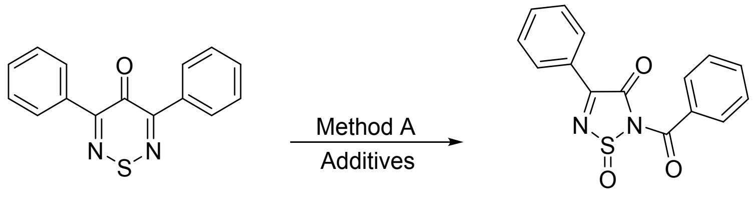

4a **5a**

**Entry**	**Additive**	**Function**	**Solvent**	**Light wavelength (nm)**	**Reaction time (min)**	**Conversion to 5a (%)**
**1**	TEMPO, 2 equiv.	Radical trap[13]	$CDCl_3$	420	45	100
**2**	DABCO, 2 equiv.	$^1O_2$ trap[14]	$CDCl_3$	420	1440	trace
**3**	Ph_3P, 2 equiv.	$^1O_2$ trap[15]	$CDCl_3$	420	120	100
**4**	*t*-BuOH, 2 equiv.	OH^- trap[16]	$CDCl_3$	420	15	100
**5**	1,4-Benzoquinone, 2 equiv.	$O_2^{\cdot-}$ trap[17]	$CDCl_3$	420	45	100
**6**	DMPO, 5 equiv.	$O_2^{\cdot-}$ and $^1O_2$ trap[17,18]	$CDCl_3$	420	1440	0, 33% of **4a** left
**7**	$CuCl_2$, 2 equiv.	SET trap[13]	Acetone-d_6	420	1440	Trace
**8**	1,4-Dimethoxybenzene	R_2S^{+*} trap[19]	$CDCl_3$	420	120	100
**9**	Ph_2SeO, 2 equiv.	Persulfoxide trap[20]	$CDCl_3$	420	15	100
**10**	Methylene blue chloride, 1 mol%	Chromatically orthogonal $^1O_2$ photosensitizer[9]	$CDCl_3$	620	60	100
**11**	CaO_2□$2H_2O_2$, 5 equiv.	dark $^1O_2$ generator[21]	THF-d_8	-	120	48
**12**	1,4-Dimethylnaphthalene endoperoxide, 5 equiv.	dark $^1O_2$ generator[22]	$CDCl_3$	-	60	57
**13**	KO_2 in 18-crown-6 (50:50), 5 equiv.	$O_2^{\cdot-}$ source[7]	DMSO-d_6	-	1440	0
**14**	KO_2 in 18-crown-6 (50:50), 5 equiv., N_2	$O_2^{\cdot-}$ source	DMSO-d_6	420	1440	0

The participation of $^1O_2$ was further supported by using the trap 5,5-dimethyl-1-pyrroline-*N*-oxide (DMPO) as additive (Table S2, entry 6). This trapping species is also able to trap $O_2^{\cdot-}$, resulting in a common intermediate, but it is unlikely that $O_2^{\cdot-}$ is present based on other control experiments (*vide infra*). DMPO is also commonly used as an ROS spin trap in electron paramagnetic resonance (EPR) spectroscopy.[17] When an equimolar mixture of thiadiazine **4a** and DMPO were irradiated for 10 min using a UV lamp (320-400 nm), an EPR signal was obtained (Figure S8), but its complex morphology and the resulting hyperfine splitting constants meant that the identity of the EPR-active adduct(s) responsible for the signal remained inconclusive, albeit proving ROS generation when thiadiazine **4a** is irradiated.

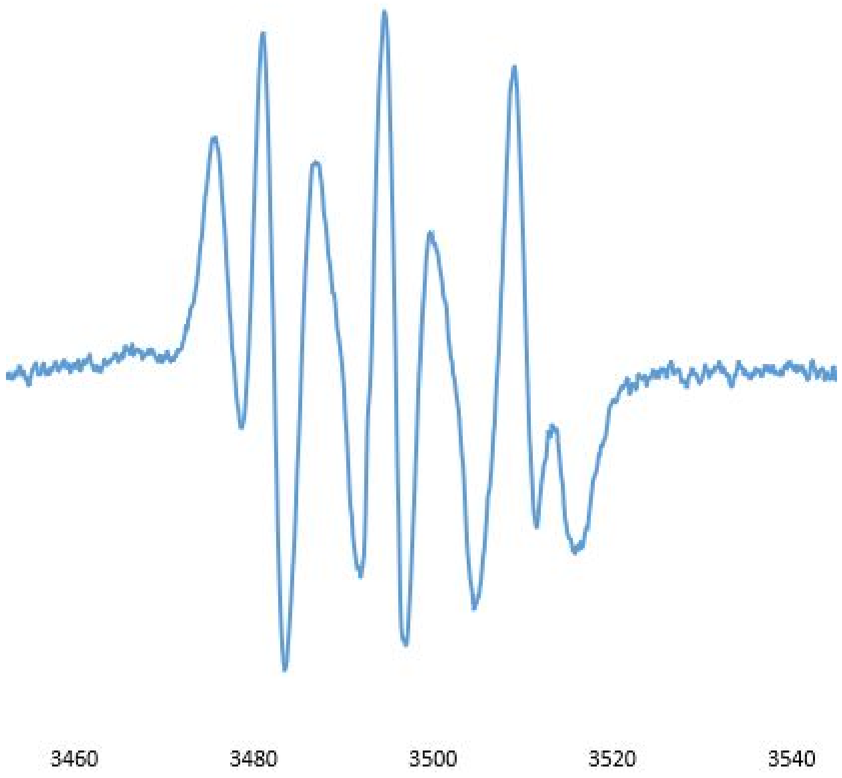

Figure S8. EPR spectrum obtained upon irradiation (320-400 nm, exposure 10 min) of an aerated chlorobenzene (CB) solution containing dissolved thiadiazine **4a** (20 mM) and DMPO (20 mM). Microwave power was 10 mW, number of sweeps 5, sweep time 14.9 sec, and sample temperature 26.4 °C. In the absence of DMPO, complete conversion of thiadiazine **4a** to thiadiazole **5a** occurs in 60 min under the aforementioned conditions (confirmed by ^1H NMR), without generating any EPR-active adducts.

Use of $CuCl_2$ (Table S2, entry 7) led to no reaction, implying that single electron transfer (SET) processes were potentially needed for the ring contraction to occur. However, other trapping experiments contradict the presence of SET processes, and the addition of $CuCl_2$ may have had other unexpected effects on the reaction so this is inconclusive.

Use of the sulfide trap 1,4-dimethoxybenzene (Table S2, entry 8) led to a suppression of the formation of thiadiazole **5a**, taking 2 h for full consumption of thiadiazine **4a**. Use of diphenylselenoxide (Table S2, entry 9), which selectively reacts with persulfoxide anions (RS^+OO^-) to form diphenylselenone had no effect on the reaction time, while LC-MS analysis showed the absence of the selenone, which makes the involvement of persulfoxide anion intermediates unlikely. A series of known dark $^1O_2$ sources were also investigated to assess the role of light on the reaction mechanism. These included an orthogonal photosensitizer, methylene blue (Table S2, entry 10), and two dark $^1O_2$ generators, which slowly release $^1O_2$ in solution at room temperatures (Table S2, entries 11 & 12). In all cases, the thiadiazole **5a** was formed in 48-100% conversion. Despite using a 5 equivalent excess of dark singlet oxygen generators, complete conversion was not attained. This was attributed to other non-reactive pathways for the decay of $^1O_2$ - we assume primarily through vibronic energy transfer with solvent molecules which leads $^1O_2$ to decay non-radiatively to its ground state, $^3O_2$.[4] The observation that the reaction can be driven by dark $^1O_2$ generators is strong evidence that $^1O_2$ is implicated in the reaction mechanism and also shows that the ring contraction is not dependent on the thiadiazine, or an intermediate, being in an electronically excited state.

Finally, we employed a dark source of $O_2^{\cdot -}$ (Table S2, entry 13), and no conversion was observed. To exclude whether an electronic excited state of thiadiazine **4a** and superoxide were required to drive the reaction, we performed the same system under an inert atmosphere with irradiation from the 420 nm LED module (Table S2, entry 14). Again, no conversion to thiadiazole **5a** was observed. Considering Table S2, entries 5, 13 & 14, we conclude that it is highly unlikely that superoxide is involved in the reaction mechanism and this was further supported by computational studies (see Section S4).

S2.3 Concurrent ring contraction of two symmetrical 1,2,6-thiadiazines

A control experiment was performed to assess whether it was likely that the 3,5-substituents of the thiadiazines fully dissociate from the core during the ring contraction. To explore this, an equal mixture of two symmetrical thiadiazines, **4k** and **4l**, was produced and subjected to standard batch irradiation conditions (Figure S9). These thiadiazines undergo the ring contraction independently to yield ring contracted products **5k** and **5l**, respectively, and if dissociation and recombination processes were occurring, we would expect to see some formation of the mixed products **A** and **B**. However, mixed products were not observed as all of the ^{1}H NMR resonances were rationalized as a combination of **5k** and **5l**, by comparison with their isolated ^{1}H NMR spectra. Additionally, diffusion-ordered NMR spectroscopy (DOSY) revealed that the reaction mixture was indeed a binary mixture of **5k** and **5l** (Figure S10). This suggests that the substituents of the thiadiazine rings remain bound to the core as it contracts, and is therefore an intramolecular process.

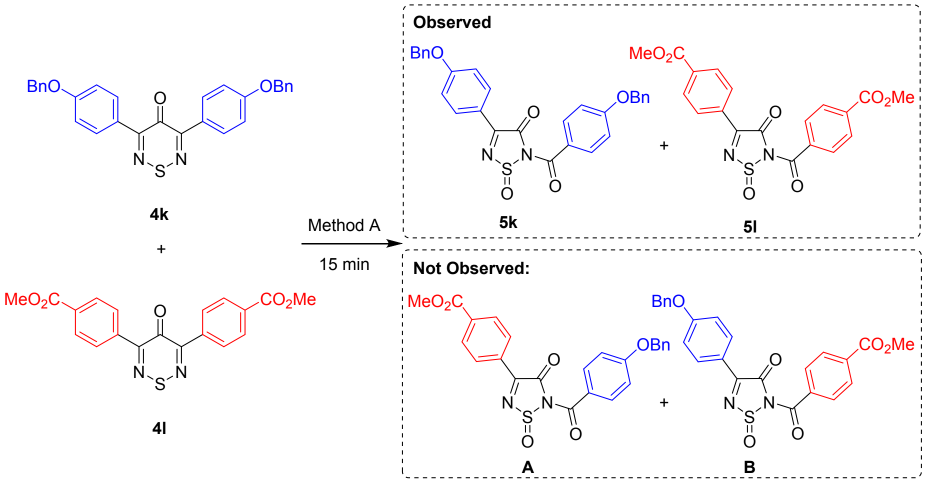

Figure S9. Potential outcomes when two symmetrical 1,2,6-thiadiazines are irradiated together, simultaneously, if the 3,5-substituents of the thiadiazines dissociate during the reaction and able to exchange.

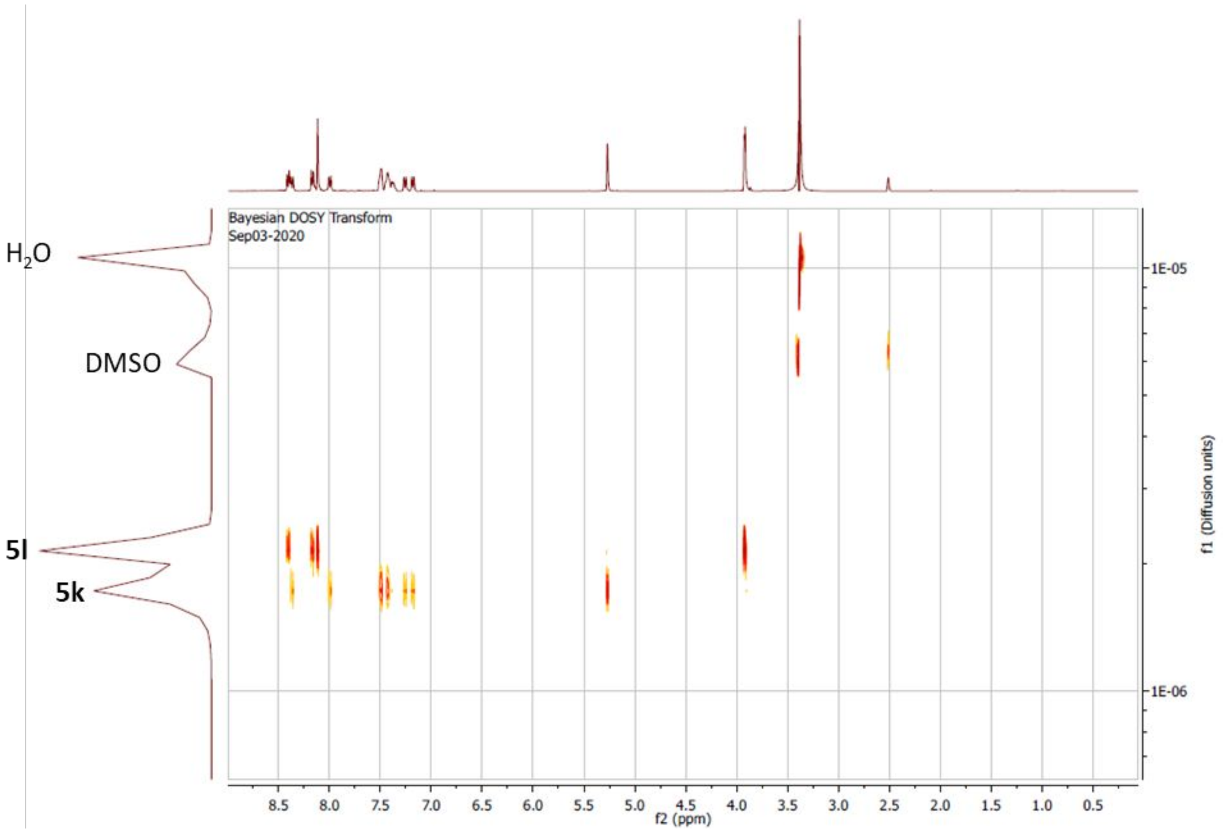

Figure S10. DOSY spectrum after irradiating (1 h) a mixture of **4k** and **4l** in DMSO-d_6. The presence of two distinct diffusion coefficients for each of the products **5k** and **5l** support that these are the only species present in the solution.

S2.4 Equivalents of O_2 needed for the complete transformation of **4a** to **5a**

To assess the stoichiometry of the reaction, we performed the reaction under standard conditions (batch, $CDCl_3$, 37.5 mM, 100 mL scale), but using degassed solvent (freeze-pump-thaw) and under an inert N_2 atmosphere. Performing the reaction in the absence of oxygen yielded no conversion of thiadiazine **4a** to thiadiazole **5a**, and the starting material was recovered in full. This confirmed that the reaction requires oxygen. We then performed the reaction under the same conditions, whilst injecting volumes of oxygen to achieve stoichiometric ratios of thiadiazine **4a** to O_2. To account for the large volume and slow dissolution of O_2 from the reactor headspace to the solution phase, the reaction was run for up to 3 h and conversion was monitored periodically by ^{1}H NMR. The results of the experiments were plotted as conversion *vs* O_2 mol equivalents (Figure S11). Linear regression of the data revealed a gradient which was approximately equal to 1, indicating that thiadiazine **4a** and O_2 react in a 50:50 ratio to achieve conversion to thiadiazole **5a**. This also suggested that both of the oxygen atoms that result on the ring contracted product originate from a single O_2 molecule.

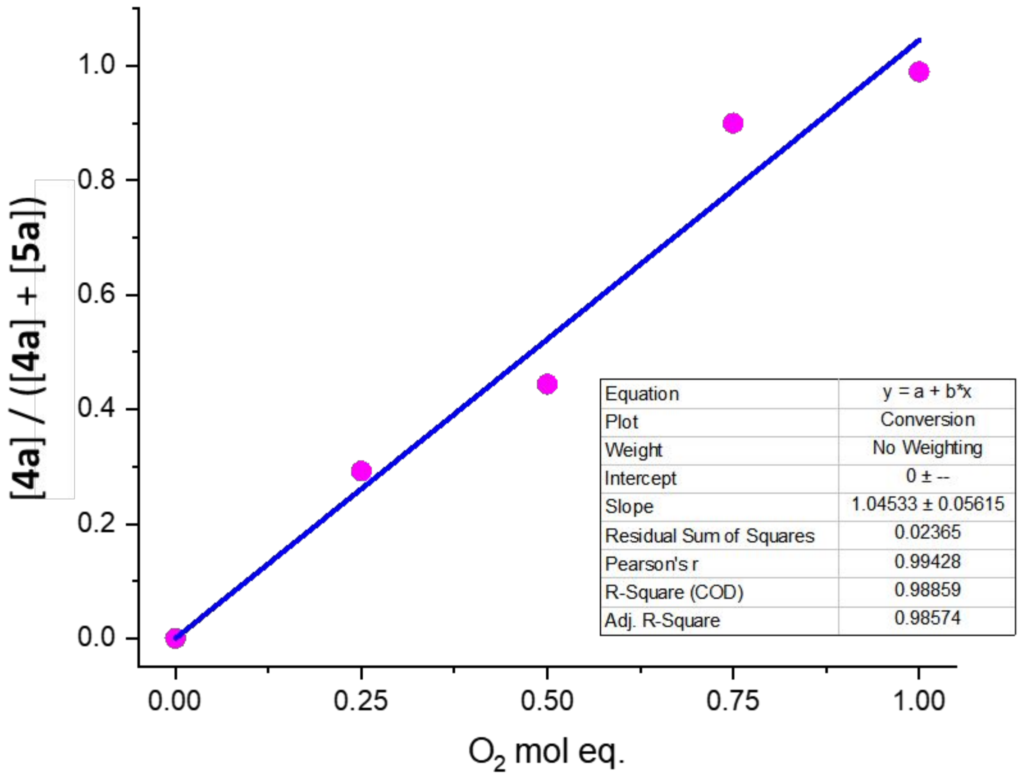

Figure S11. Chart showing the relationship between conversion of **4a** to **5a** and O_2 mol equivalents.

S2.5 Kinetic analysis

A series of experiments were performed to assess the kinetics of the reaction in various solvents and under different conditions. The model reaction of converting thiadiazine **4a** to thiadiazole **5a** was studied under batch conditions using continuous flow sampling setup (Figure S12), which sampled the crude reaction mixture to a

spectrofluorometer (PASCO PS2600 wireless spectrofluorometer) using a flow-through cuvette (Hellma GmbH & Co. KG; 176-761-15-40, 5 mm path length, V = 140 μL). The reaction mixture was pumped using a peristaltic pump of the Vapourtec Ltd. E-series flow chemistry system *via* transparent polyfluoroalkoxy alkane (PFA) tubing (1 mm ID) at a flow rate of 5 mL/min. The total internal volume of the sampling loop and spectrometer, including the tubing and cuvette chamber, was approximately 1.71 mL. This provided a theoretical residence time of reaction mixture in the flow system of *ca.* 20 s. Automated reaction monitoring was performed by setting the spectrometer to monitor the absorbance intensity of the reaction mixture at 400 nm, with a bandwidth of 1 nm and a 0.5 Hz sample rate. 400 nm was chosen as it is an orthogonal wavelength which thiadiazine **4a** absorbs and the product **5a** does not. Although the absorbance spectrum of thiadiazine **4a** did display some mild solvatochromism, a sufficient absorbance intensity at 400 nm for reaction monitoring was present in all solvents and the absorbance of thiadiazole **5a** remained orthogonal.

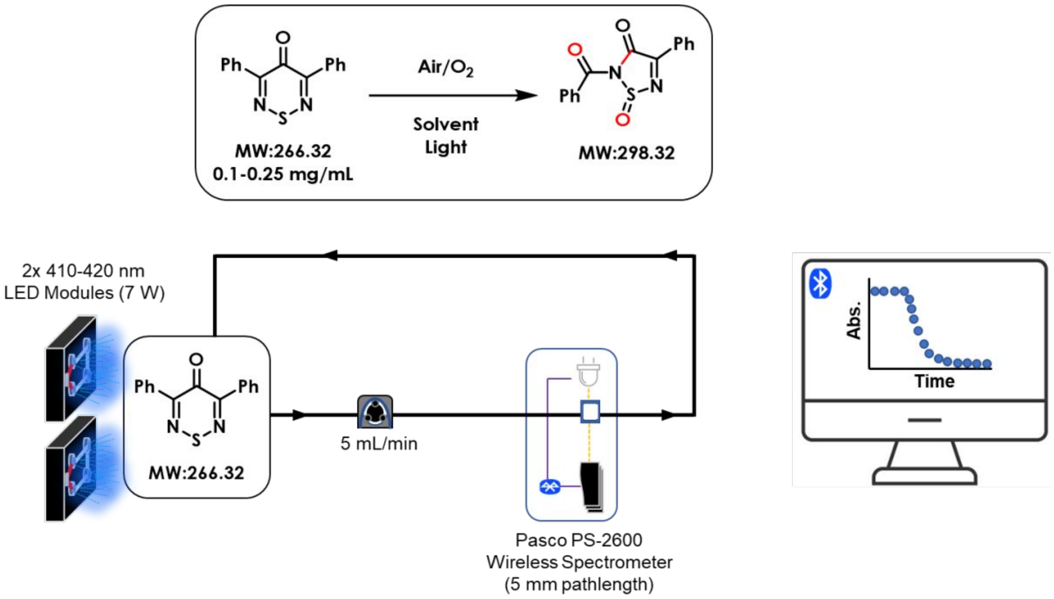

Figure S12. Continuous flow sampling loop setup for reaction monitoring and kinetic analysis.

Reactions were performed at a 10 mL scale and at a reduced concentration, from the optimized reaction conditions presented in the manuscript, of 9.38 × 10^{-4} mM wrt thiadiazine **4a** (standard conditions: 37.6 mM), which was necessary for spectral clarity. In general, reactions were performed with air-saturated solvents and the reaction vessel was left open to air during the reaction. The reaction vessel was irradiated by two LED array modules, built in-house. The modules feature six 3 W violet LEDs (410-420 nm) affixed to an aluminium heatsink. The spectral data of the LEDs can be obtained on the suppliers website (Future Eden Ltd., UK, product webpage: https://futureeden.co.uk/products/3w-violet-led-410-420nm-epiled-with-star-pcb-heatsink). The two led modules were place around the reaction vessel at 90° to each other and at equal distance (~7 cm).
Analysis was performed by first preparing the reaction solution in a 20 mL pear-shaped flask, sealed with a septum. The septum was pierced by stainless steel needles which connected to the flow reactor system, and a needle which opened allowed gas to exchange between the reactor headspace and the atmosphere. Reaction mixture was flowed through the system and the visible light absorbance of the mixture was recorded by the spectrometer, prior to irradiation. The spectrometer was then set to measure the absorbance intensity of the

reaction mixture until a stable absorbance steady-state was established, indicating the absorbance intensity of thiadiazine **4a** at starting concentration. Once this was achieved, the LED modules were activated and the decay in absorbance intensity, as thiadiazine **4a** converts to thiadiazole **5a**, was monitored by the spectrometer until a new absorbance steady-state was achieved, indicating full conversion, which was validated by analyzing the reaction mixture *via* ^{1}H NMR spectroscopy. An example of reaction monitoring raw-data is presented below (Figure S13, part A), for a reaction performed at 0.1 mg/mL initial concentration wrt thiadiazine **4a**. The data sets were processed by subtracting the long-time baseline, normalizing the absorbance intensity and cropping the time periods before the reaction was initiated and after the 2nd absorbance steady-state was achieved (Figure S13, part B). The normalized absorbance data were then converted to relative concentration using the known quantity of thiadiazine **4a** added to the reactor by assuming that absorbance and concentration are linearly proportional, in accordance with the Beer-Lambert Law (Figure S13, part C).

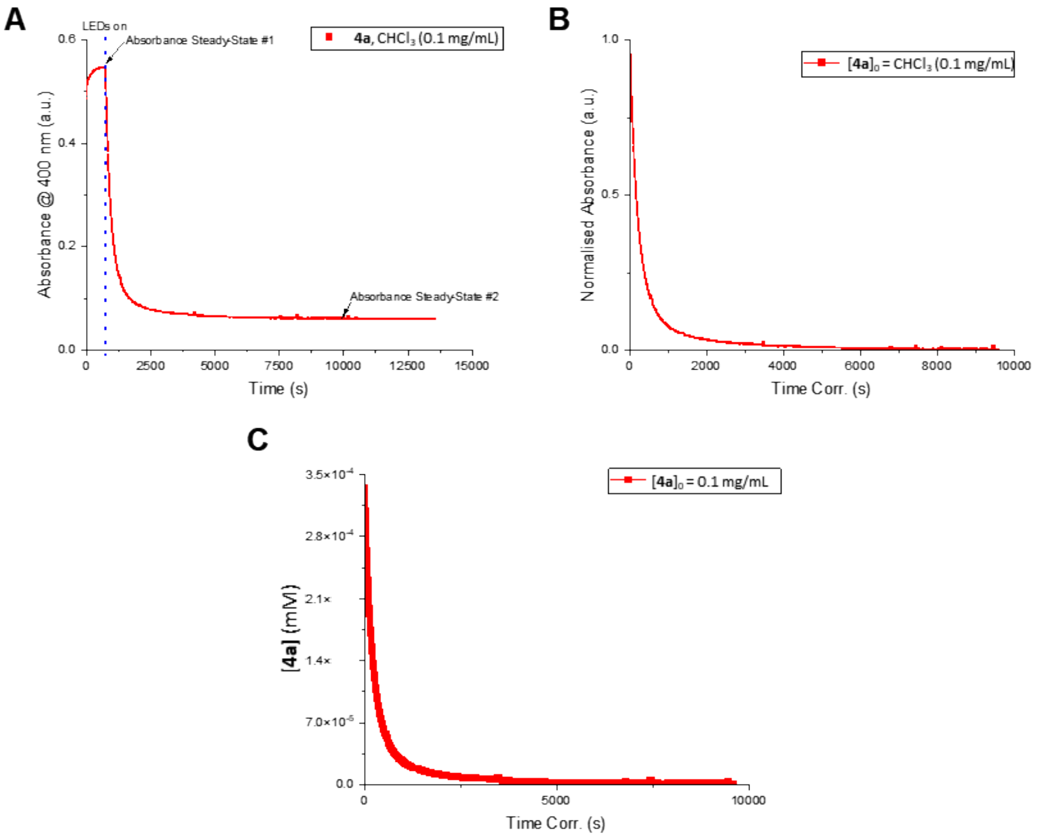

Figure S13. (A) Reaction monitoring trace using raw data from the spectrophotometer. The absorbance steady-states have been labelled. The dashed blue line indicates approximately when the LED modules were activated. (B) Normalized absorbance *vs* corrected time for the raw data. The data prior to the LEDs activation, and after the 2nd absorbance steady-state is first achieved, is removed and time is corrected so that $t = 0$ when the absorbance intensity drops below the steady-state value and does not return to it. (C) The same normalized trace with absorbance converted to concentration using the known quantity of thiadiazine **4a** added to the reactor.

The concentration data’s natural logarithm (ln[**4a**]) and reciprocal (1/[**4a**]) were then plotted *vs* time to assess if the data fitted a simple 1st or 2nd order kinetic profile (Figure S14). Neither resulted in linear relationships when considering the entire kinetic profile, however, the early sections of the profiles (<500 s, <75% conversion) fit relatively well to a 1st order model. This process was repeated for each of the experiments in various solvents and conditions applied.

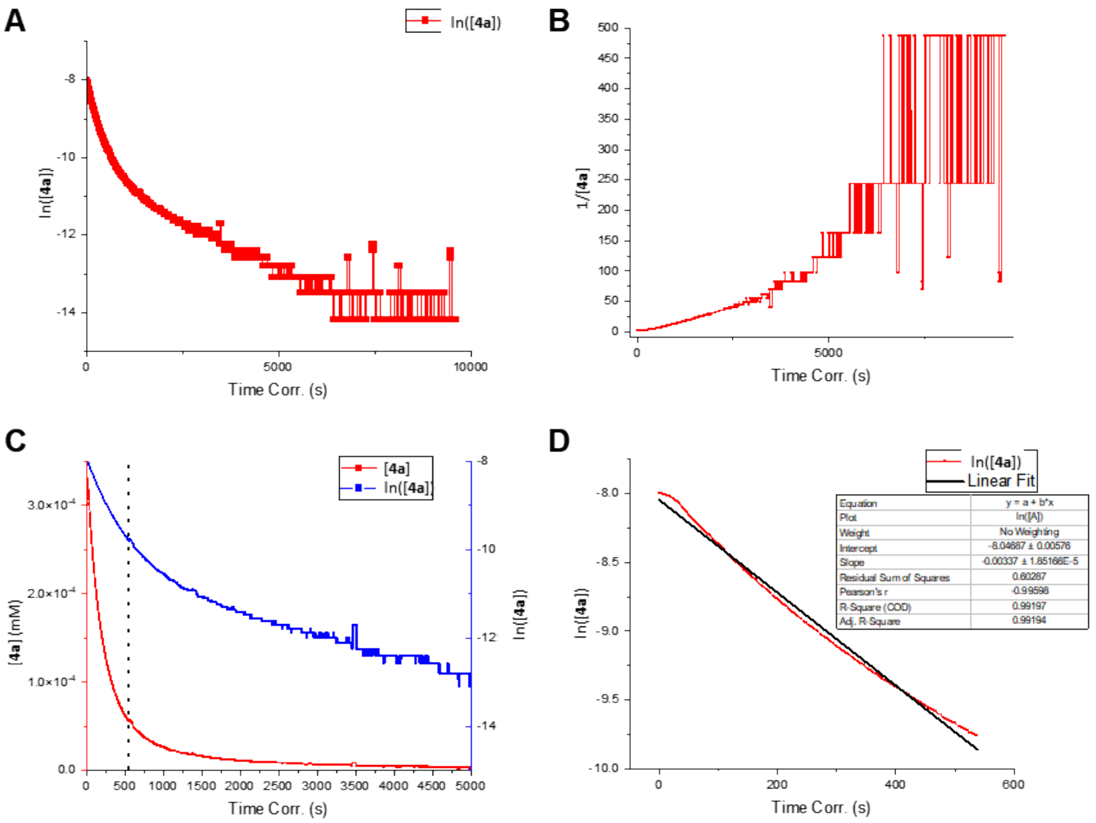

Figure S14. (A) plot of ln([**4a**]) *vs* time for the full reaction monitoring trace displayed in Figure S13C. (B) plot of 1/[**4a**] *vs* time for the full reaction monitoring trace displayed in Figure S13C. (C) Stacked plot of [**4a**] (red, left axis) and ln([**4a**]) (blue, right axis) *vs* corrected time. The dotted black line indicates the approximate time when the reaction begins to deviate from a 1st order kinetic regime. (D) plot of ln([**4a**]) *vs* corrected time for the initial reaction kinetics with linear regression analysis.

It was observed that the initial 10-20 s of each kinetic trace were inconsistent because 'time = 0' had been assigned subjectively, and due to an induction period of approximately 20 s based on the volume of the flow system. To correct this, the data for each trace was cropped to examine ln([**4a**]) between 90-25% of the initial concentration and reaction time was corrected to zero at 90% remaining [**4a**]. The effect of this on the kinetic traces can be seen in Figure S15. The observed 1st order rate constants (k_{obv}) for the initial reaction rate were obtained *via* linear regression analysis of the ln([**4a**]) traces for each solvent, and are presented in Table S3.

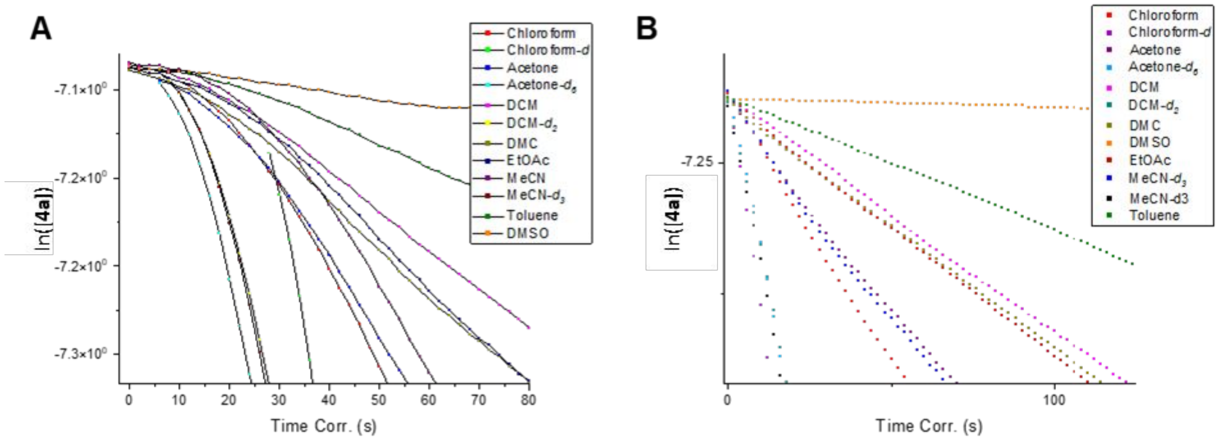

Figure S15. Comparison of initial few seconds of the kinetic traces (A) before and (B) after they were adjusted to show [**4a**] between 90-25% of the initial concentration, removing the subjective assignment of t = 0, and the variable induction period from the analysis.

Table S3. Summary of experimentally observed rate constants in each of the solvents tested.

Entry	Solvent	k_{obv} / s^{-1}
**1**	$CHCl_3$	(4.35+/-0.013) × 10^{-3}
**2**	$CDCl_3$	(2.73+/-0.033) × 10^{-2}
**3**	Acetone	(3.25+/-0.013) × 10^{-3}
**4**	Acetone-d_6	(2.08+/-0.026) × 10^{-2}
**5**	DCM	(2.04+/-0.004) × 10^{-3}
**6**	DCM-d_2	(1.49+/-0.021) × 10^{-2}
**7**	Dimethyl carbonate (DMC)	(1.97+/-0.006) × 10^{-3}
**8**	EtOAc	(2.08+/-0.007) × 10^{-3}
**9**	MeCN	(3.12+/-0.017) × 10^{-3}
**10**	MeCN-d_3	(2.06+/-0.030) × 10^{-2}
**11**	Toluene	(1.36+/-0.002) × 10^{-3}
**12**	DMSO	(6.57+/-0.001) × 10^{-5}

The accelerated reaction kinetics observed in deuterated solvents was indicative of the involvement of $^1O_2$. To assess this, the lifetime of $^1O_2$ (τ_Δ) in each of the solvents were obtained from literature references.[23-25] Values from reference 23 were averaged and the reported error is the standard deviation of the values for the same solvent. The values obtained are displayed in Table S4. The values for lifetime in DMSO are taken from references 24 and 25, as these are more recent articles and were in close agreement with each other, whereas there was only a single value in reference 23. The reported lifetime of $^1O_2$ in DCM-d_2 is surprisingly low, but we were unable to find another reference for τ_Δ in this solvent.

Table S4. Summary of literature values for the lifetime of $^1O_2$ in the solvents tested.

Entry	Solvent	τ_Δ (μs)[a]	τ_Δ (μs)
**1**	$CHCl_3$	261+/-127	-
**2**	$CDCl_3$	3285+/-3142	-
**3**	Acetone	48+/-4	-
**4**	Acetone-d_6	770+/-140	-
**5**	DCM	86.5+/-23	-
**6**	DCM-d_2	120	-
**7**	Dimethyl Carbonate (DMC)	-	-
**8**	EtOAc	47.5	-
**9**	MeCN	77+/-4	-
**10**	MeCN-d_3	890+/-330	-
**11**	Toluene	28.6+/-0.7	-
**12**	DMSO	30	5.5+/-0.3,[b] 5.6[c]

[a] Values from ref. 23. [b] Value from ref. 24. [c] Value from ref. 25.

A plot was formed of the observed initial rate constants for each of the solvents, using the refined rate constants discussed above, *vs* τ_Δ in each of the solvents (Figure S16). The plot shows a clear positive, non-linear relationship between the observed rate constant and lifetime of singlet oxygen which we suggest as compelling evidence that $^1O_2$ is involved in the reaction mechanism.

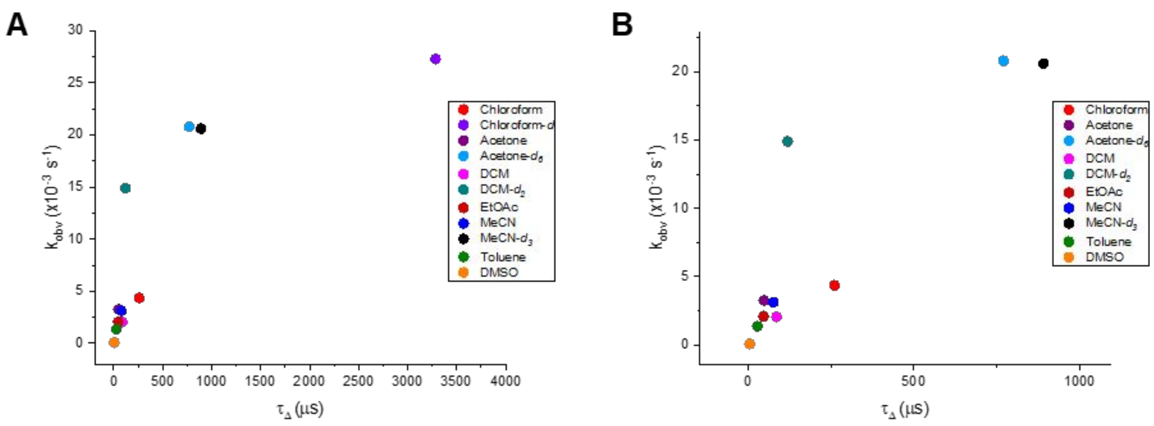

Figure S16. (**A**) Plot of k_{obv} *vs* τ_Δ for each of the solvents screened. (**B**) Plot of k_{obv} *vs* τ_Δ, excluding chloroform-*d* for clarity of the cluster of points at $\tau_\Delta < 250$ μs.

In addition to screening kinetics in various reaction solvents, the same analysis was performed to analyze the effect of other conditions on the reaction, displayed in Figure S7A. Kinetic data for the reaction in $CHCl_3$ and $CDCl_3$ are included for reference. The conditions tested included: *(i)* reducing the starting concentration of thiadiazine **4a** by 60% (0.10 *vs* 0.25 mg/mL), *(ii)* Removing one of the LED modules, effectively reducing the irradiation intensity by 50%, and *(iii)* performing the reaction under an atmosphere of pure O_2 gas and using O_2 enriched solvent *via* sparging the solvent with O_2 for 1 h prior to use. The observed rate constants are summarized in Table S5.

Table S5. Summary of the observed 1st order rate constants under various conditions.

Entry	Conditions	k_{obv} / s^{-1}
**1**	[**4a**]$_0$ = 0.10 mg/mL	(3.42+/-0.018) × 10^{-3}
**2**	[**4a**]$_0$ = 0.25 mg/mL, 1 × LED	(2.50+/-0.003) × 10^{-3}
**3**	[**4a**]$_0$ = 0.25 mg/mL, O_2 Atm.	(6.64+/-0.027) × 10^{-3}
**4**	[**4a**]$_0$ = 0.25 mg/mL	(4.35+/-0.013) × 10^{-3}
**5**	[**4a**]$_0$ = 0.25 mg/mL, $CDCl_3$ Solvent	(2.73+/-0.033) × 10^{-2}

Reducing the starting concentration did reduce the observed rate constant, but not substantially. Reducing the light irradiation intensity by 50% had an almost proportional effect on the observed rate constant, which was reduced by 43%. Performing the reaction under a O_2 atmosphere significantly enhanced the observed rate constant, which was increased by approximately 53% relative to the same experiment performed under air.

S2.6 Proposed kinetic mechanism

The following mechanism contains the minimum number of steps that we believe are required to be consistent with the observations presented in the presented in the manuscript and previous sections of the supporting information:

(1) $S + h\nu \rightarrow S^*$

(2) $S^* + {}^3O_2 \rightarrow ROS$

(3) $S + ROS \rightarrow P$

(4) S^* (+ solvent) $\rightarrow S$

(5) ROS (+ solvent) $\rightarrow {}^3O_2$

(6) $S^* + S \rightarrow S + S$

Assuming a photostationary state is established rapidly, relative to loss of the substrate through (3), the rate of reaction will obey:

$$v_3 = \frac{J_1 k_2 k_3 [O_2][S]^2}{(k_3[S] + k_5)(k_2[O_2] + k_4 + k_6[S])}$$

Where:

S = substrate (**4a**)

S* = excited state of substrate, formed directly or indirectly by absorption

P = product (**5a**)

ROS = reactive oxygen species, believed to be ${}^1O_2$

J_1 = effective pseudo 1st order rate constant for absorption.

$k_{i = 2\text{-}6}$ = rate constants for steps (2)-(6); (4) and (5) are pseudo 1st order in the presence of a fixed solvent concentration and maybe include other radiative or non-radiative processes.

Apparent 1st order kinetics in [S] will be observed at early times in the reaction, provided either (a) $k_3[S] >> k_5$ or (b) $k_6[S] >> k_2[O_2] + k_4$. However, (a) is not consistent with the observation that the rate is sensitive to the lifetime of ${}^1O_2$ and hence on k_5. Therefore, (b) is the more probable explanation. At sufficiently long times, as [S] declines, the reaction will approach 2nd order in [S].

S2.7 Limitations of the scope

Some substrates that reacted poorly in the optimized reaction conditions are illustrated below (Scheme S1). In summary, symmetric and asymmetric 3-chlorothiadiazines **6** were not tolerated in the reaction and either decomposed or were unreactive under batch and flow conditions. In particular, 3-chlorothiadiazines **6a**, **6c** and **6d** degraded under batch conditions after 15, 60 and 60 min, respectively, while with thiadiazine **6b** no reaction was observed after 1440 min. Similar results were also observed in flow conditions. Assuming the chloro-substituted carbon is excised, the resulting *N*-acyl chlorides would likely be unstable and so it was not surprising that they decomposed to form complex mixtures. Interestingly, in the reaction of 3,5-dichloro-4*H*-1,2,6-thiadiazin-4-one (**6a**), attempts to identify the products from the resulting crude mixture led to single crystals

which were identified by X-ray diffraction as parabanic acid **8** (Scheme S1). This shows that loss of sulfur monoxide (or dioxide) is one of the potential decomposition pathways.

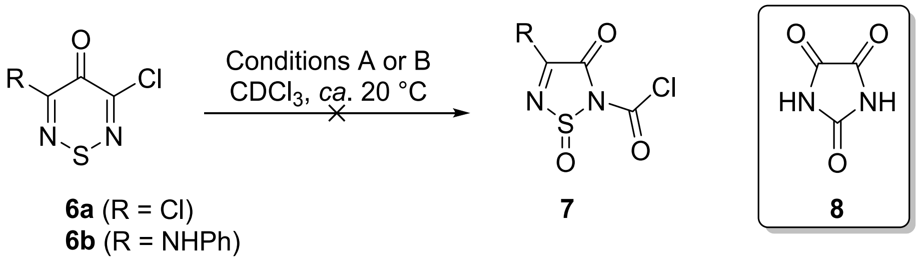

6a (R = Cl)
6b (R = NHPh)
6c (R = OPh)
6d (R = SPh)

Scheme S1. Reactions of 3-chlorothiadiazines **6**.

Two other analogues that led to unstable products were benzylsulfide **9** and *p*-nitrophenyl derivative **10** (Scheme S2). In particular, under flow conditions compound **9** decomposed after 5 min, while the same result was observed with compound **10** after 10 min reaction time. Similar results were also observed in batch conditions.

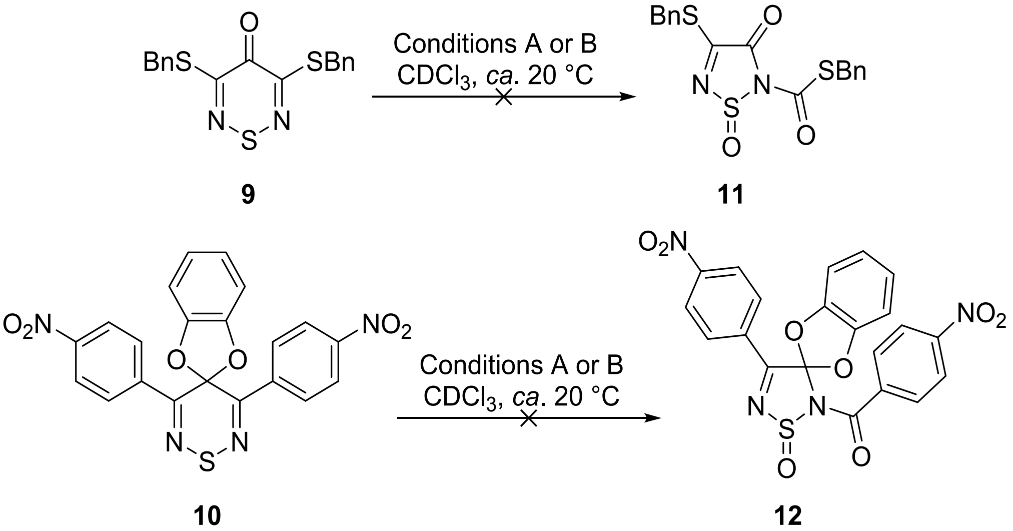

Scheme S2. Reactions of thiadiazines **9** and **10**.

When investigating the reaction of 3,5-diaminothiadiazines we found that thiadiazines substituted (either symmetric or asymmetric) with 1° and 2° amines, were unreactive or decomposed under the reaction conditions (Scheme S3). In particular, reactions of symmetric thiadiazines **13a-c** all led to decomposition under both batch and flow conditions while asymmetric thiadiazine **13d** was not reactive. Interestingly, 3-amino-substituted 5-phenyl-, 5-phenoxy and 5-thio-phenyl-thiadiazines **4af**, **4ag** and **4ah** reacted successfully to give thiadiazole products (see main manuscript, Table 2). The crystal structures of those compounds revealed a hydrogen-bonding interaction between the thiadiazine nitrogen and phenyl C-H (see Section S5). It is possible that this interaction stabilizes the thiadiazine and prevents generation or reaction with singlet oxygen when both substituents are 2° amines with available protons.

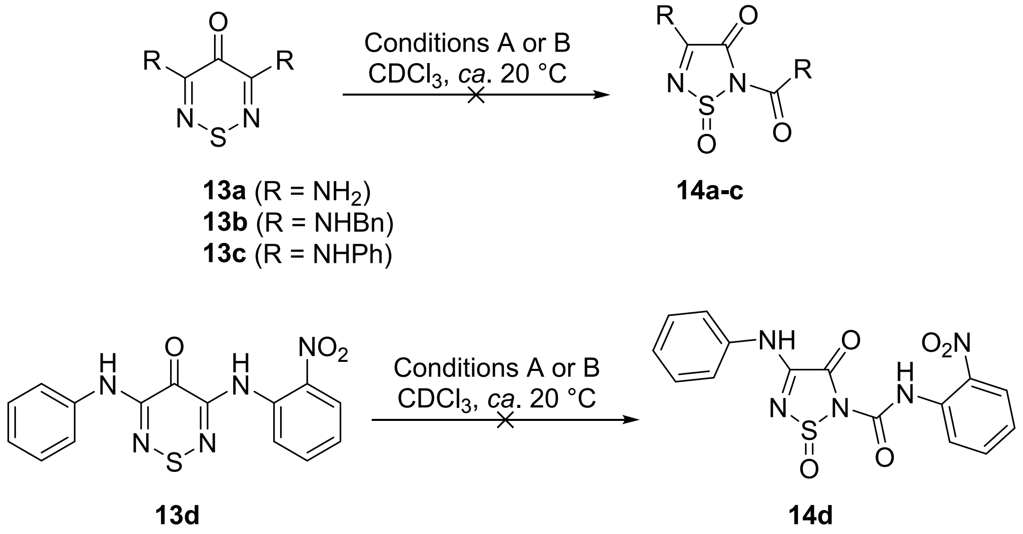

Scheme S3. Reactions of 3,5-diaminothiadiazines **13a-d**.

Reactions of two tertiary amines **15a** and **15b** did not yield the desired ring contracted products, however, batch irradiation for 24 h yielded significant quantities (62% and 44%, respectively) of the thiadiazine sulfones **18a** and **18b** (Scheme S4), which were isolated and their characterization data matched to the literature.[26] Thiadiazine sulfoxides **17a**, **17b**, and thiadiazole **16b** were also tentatively identified by LC-MS, however, thiadiazole **16a** was not identified in the crude reaction mixture.

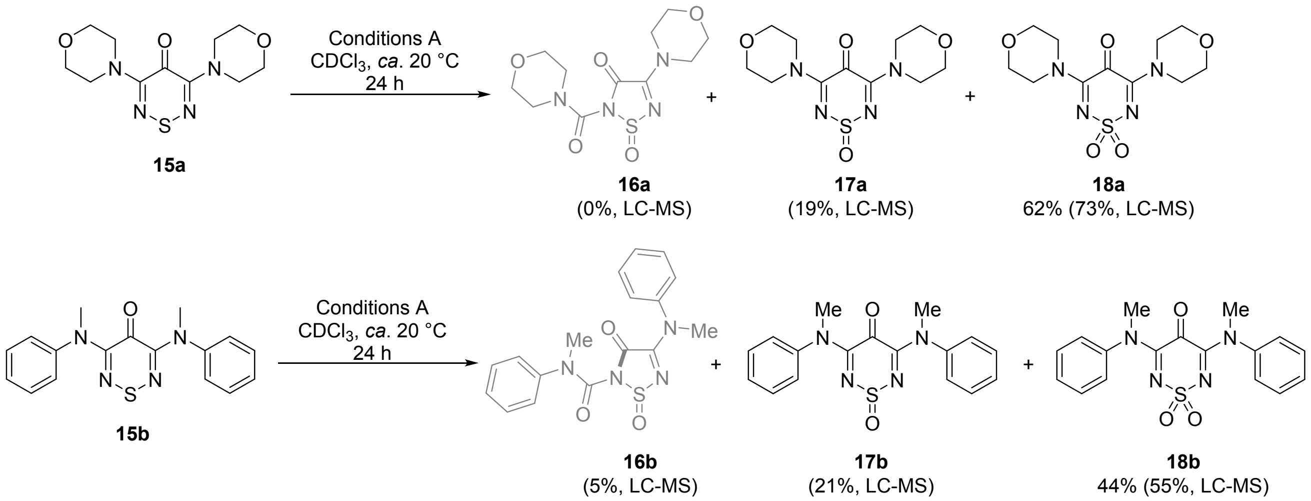

Scheme S4. Reactions of 3,5-diaminothiadiazines.

Finally, substitutions at the thiadiazine C4 position besides acetal, such as thione **19a**, imine **19b** and dicyanoylidene **19c** were not tolerated (Scheme S5). Thione **19a** led to degradation with both batch and flow conditions, while imine **19b** and dicyanoylidene **19c** were unreactive after 24 h in batch conditions.

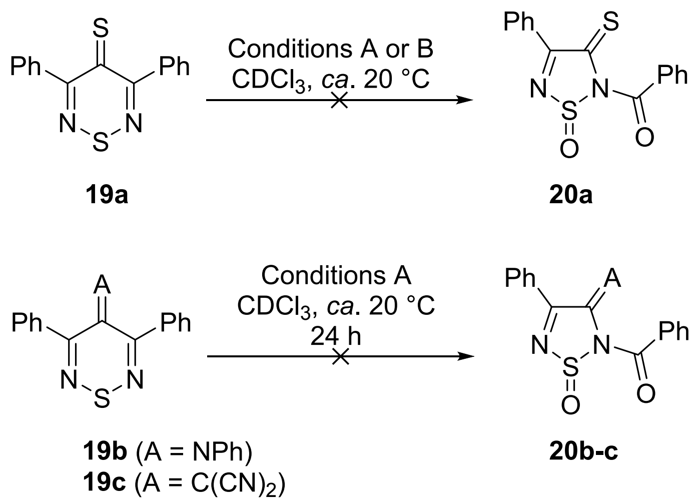

Scheme S5. Reactions of thiadiazines with alternative C4 substituents.

S3 Computational Studies

The mechanism of the reaction of thiadiazine **4a** with singlet oxygen was investigated computationally. Figure S17 shows that the initial addition of $^1O_2$ to thiadiazine **4a** can occur in two ways to give a common endoperoxide intermediate **I** at 7.4 kcal/mol: i) a concerted [3 + 2] addition across the C(Ph)–N=S moiety, via **TS(4a-5a)1** at 9.6 kcal/mol; or ii) an end-on addition of $^1O_2$ onto C3 via **TS(4a-5a)3** at 18.7 kcal/mol followed by barrierless S–O bond formation. From **I**, O–O bond cleavage occurs with concomitant ring contraction in a single step via **TS(4a-5a)2** at 21.7 kcal/mol. This process entails the simultaneous cleavage of the O–O and C3–C4 bonds as well as the formation of a new amide bond between N2 and C4 (Figure S17). The formation of the thiadiazole in **5a** is very exergonic (ΔG = -85.7 kcal/mol) and proceeds with an overall barrier of 21.7 kcal/mol, consistent with the room temperature reactivity observed experimentally. **TS(4a-5a)2** will be both the rate-determining and selectivity-determining transition state.

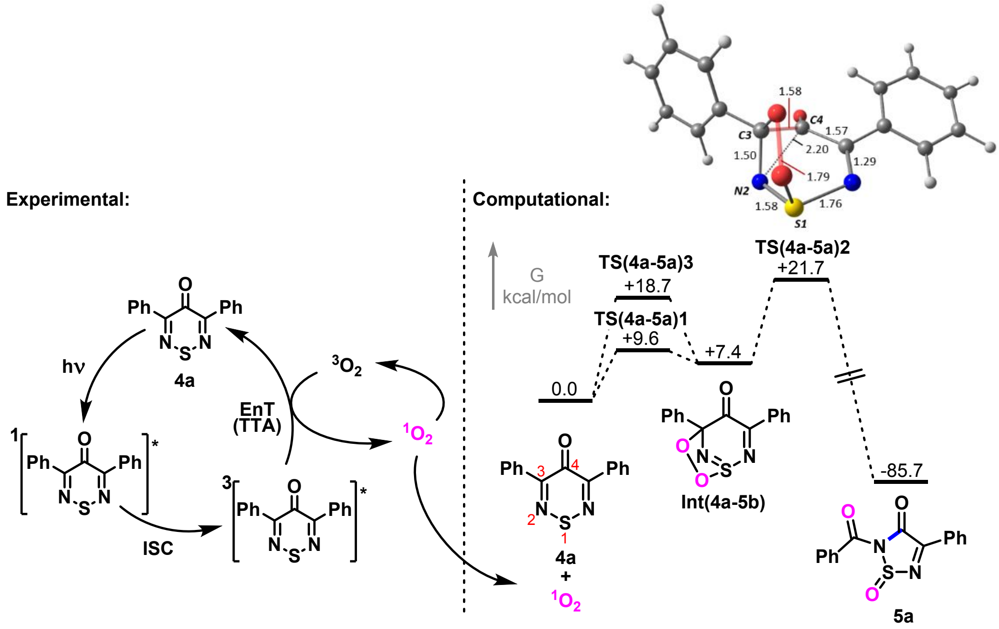

Figure S17. Proposed mechanism for the light mediated ring contraction of 1,2,6-thiadiazines to 1,2,5-thiadiazole 1-oxides. Computed free energy profiles (CASPT2(16,13)/6-31G**//UB3LYP(CHCl3)/6-31G**; kcal/mol) for the reaction of $^1O_2$ with thiadiazine **4a** to form thiadiazole **5a**. Geometry of **TS(4a-5a)3**. Breaking bonds in red, forming bond as a fragmented line. Key distances in Å.

Below we present a mechanism that we believe contains the minimum number of elementary steps to be consistent with the findings of the experimental and computational mechanistic studies and kinetics. In summary (Figure S17), the mechanism includes absorption by the substrate, thiadiazine **4a**; energy transfer from excited **4a** to $^3O_2$ to generate $^1O_2$ and return ground state **4a**; $^1O_2$ then subsequently reacts with the substrate to form the product, thiadiazole **5a**, via the [3 + 2] cycloaddition-ring contraction cascade discussed above; quenching of the $^1O_2$ by the solvent; and quenching of the excited state of the substrate, either by the solvent or by self-quenching. Assuming that establishment of a photostationary state is rapid relative to reaction, and with appropriate relative rates of other processes, this mechanism is consistent with the observed switch from approximately 1st order to 2nd order kinetics during the course of the reaction and with the observed sensitivity of the rate to other parameters (O_2 concentration, light intensity, and temperature).

To the best of our knowledge, concerted [3 + 2] cycloadditions with $^1O_2$ are unprecedented, with its existence previously only being tentatively suggested.[27] The discovery of this reactivity expands the already rich diversity

of $^1O_2$ applications in chemical synthesis, unlocking new potential to achieve complex targets and novel chemical structures.

S3.1 Methodology

Geometry optimizations were run with Gaussian 16 (Revision A.03).[28] Two different protocols were used for this optimizations; initially CASSCF[29,30] (16,13) was employed with the 6-31G** basis set.[31,32] However, following difficulties locating the key ring contraction/O-O cleavage transition state with this approach, an alternative approach using UB3LYP[33]/6-31G** was employed with single point energies corrected at the CASPT2[34,35] (16,13)/6-31G** level (see Figure S18 for a comparison of these approaches). Stationary points were characterized with either numerical frequency calculations (CASSCF) or analytical frequency calculations (UB3LYP). UB3LYP Transition states (one negative frequency) were characterized *via* IRC calculations and subsequent geometry optimizations to confirm adjacent minima (no negative frequencies).

The choice of active space was explored using the simplified model **4a^H^** (*a*, Figure S18). The active space of the substrate comprised the 7 π orbitals (8 electrons) present in the ring and the C=O group (*b*, Figure S18), while the active space of the oxygen (*c*, Figure S18) consisted of both the σ and π molecular orbitals resulting from the combination of 2*p* orbitals (8 electrons in 6 orbitals). This gave a (16,13) total active space.

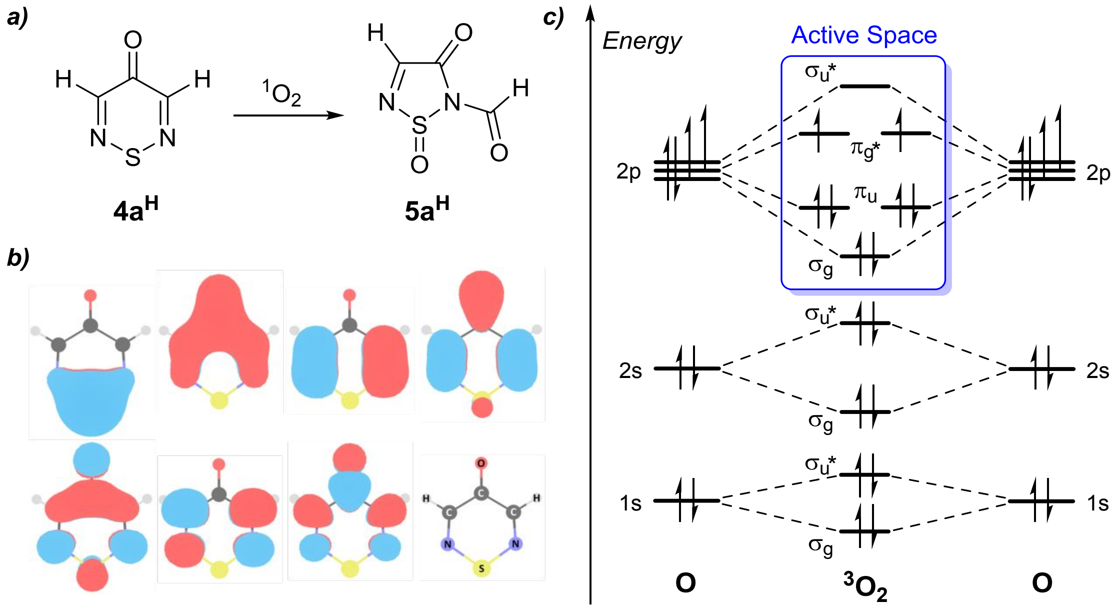

Figure S18. *a*) Model reaction for initial CASSCF calculations; *b*) orbitals included in the active space for the substrate; and, *c*) the molecule of oxygen, using the ground triplet state as an example.

The potential energy surface of the reaction between **4a^H^** and singlet oxygen (electronic state $^1\Delta_g$) was investigated using this protocol and the stationary points were identified (Figure S19). The final energies were computed in Molpro (Version 2012.1)[36] using CASPT2. Owing to the large size of the active space, the internally contracted 2nd order Rayleigh Schrödinger perturbation theory (RS2C)[37] was selected and the ionization potential-electron affinity shift IPEA[38] (ϵ = 0.02) was used to avoid intruder state problems.

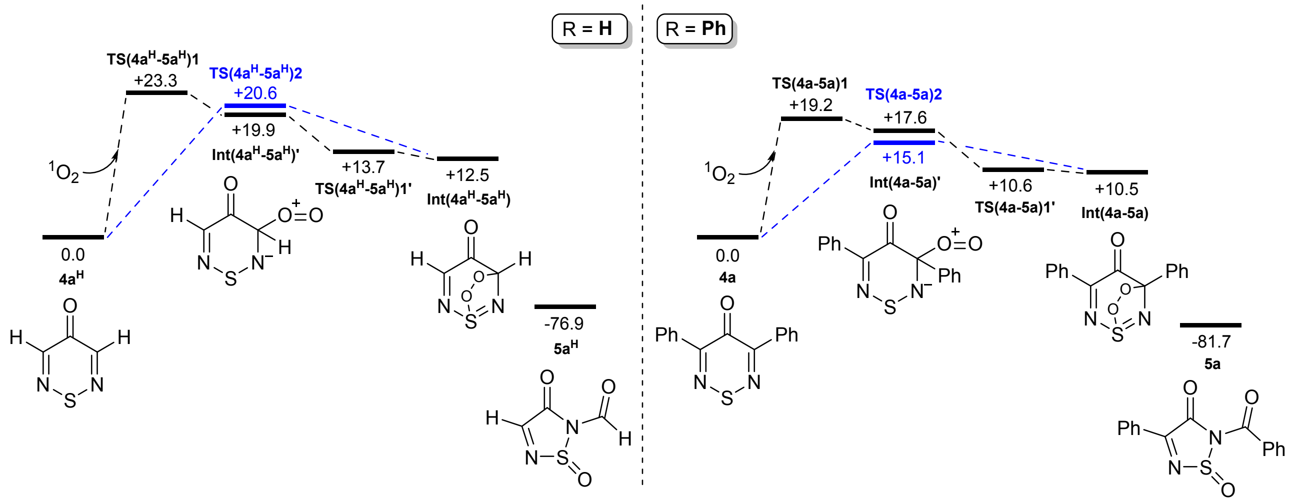

Figure S19. Computed free energy profiles (CASPT2/6-31G**//CASSCF/6-31G**; kcal/mol) for the reaction of $^1O_2$ with **4a^H** (left) and **4a** (right). Free energies in kcal/mol.

The end-on attack of singlet oxygen can occur with an energy barrier of 23.3 kcal/mol *via* **TS(4a^H-5a^H)1** (left, Figure S19), affording intermediate **Int(4a^H-5a^H)'** to then form endoperoxide **Int(4a^H-5a^H)** in a barrierless process *via* **TS(4a^H-5a^H)1'**. It is also possible for the concerted addition of $^1O_2$ to occur through **TS(4a^H-5a^H)2** to directly provide intermediate **Int(4a^H-5a^H)**. No ring contraction transition state could be located using CASSCF. The use of the parent substrate, thiadiazine **4a**, used experimentally, led to a similar energy diagram (right, Figure S19) where again no transition state connecting the endoperoxide intermediate **Int(4a-5a)** and the product could be identified.

The potential energy surface of the reaction of thiadiazine **4a^H** was explored with UB3LYP/6-31G**. Following this methodology equivalent intermediates and structures were located along with **TS(4a^H-5a^H)3**, a ring contraction/O-O cleavage transition state connecting the endoperoxide intermediate **Int(4a^H-5a^H)** and the final thiadiazole **5a^H**. The energy of these structures was computed using CASPT2 and, as shown in Figure S20, the energy profile is very similar regardless of the optimization protocol.

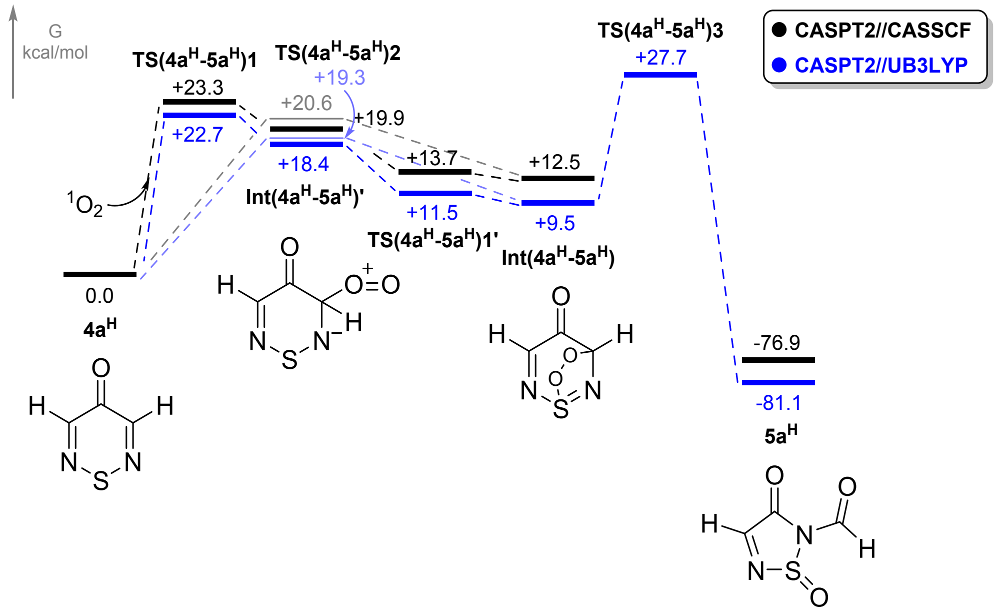

Figure S20. Free energy profiles for the reaction of $^1O_2$ with thiadiazine **4a^H** to form thiadiazole **5a^H** comparing results at the CASPT2/6-31G**//CASSCF/6-31G** (black) and CASPT2/6-31G**//UB3LYP/6-31G** (blue) levels of theory. Free energies in kcal/mol.

We thus chose CASPT2/6-31G**//UB3LYP/6-31G** as our theory level to compute the reaction of the experimental substrate **4a** using the PCM[39] method with chloroform as solvent. The resulting energy diagram is shown in Figure S17.

All geometries are provided as a separate XYZ file.

S3.2 Computed structures (Å) and energies (a.u.)

$4a^H$ CASPT2//CASSCF
CASSCF energy = -696.072424
Enthalpy 0K = -696.014071
Enthalpy 298K = -696.008325
Free energy 298K = -696.043201
CASPT2 energy = -696.960704
Low freq. = 261.3135 cm-1
Second freq. = 469.3745 cm-1

S	0.000000	0.000000	0.000000
N	-1.344486	-0.000256	0.984609
C	-1.264312	-0.000264	2.262938
C	0.000000	0.000000	3.032365
C	1.264316	0.000000	2.262928
N	1.344540	-0.000052	0.984602
O	0.000021	-0.000918	4.243978
H	-2.180147	-0.000730	2.830972
H	2.180131	-0.000500	2.830998

$^1O_2$ CASPT2//CASSCF

CASSCF energy = -149.659585
Enthalpy 0K = -149.656199
Enthalpy 298K = -149.652889
Free energy 298K = -149.675179
CASPT2 energy = -149.902804
Low freq. = 1486.4728 cm-1

O	1.239380	0.000000	0.000000
O	0.000000	0.000000	0.000000

TS($4a^H$-$5a^H$)1 CASPT2//CASSCF

CASSCF energy = -845.677320
Enthalpy 0K = -845.614395
Enthalpy 298K = -845.606585
Free energy 298K = -845.646275
CASPT2 energy = -846.843781
Low freq. = -650.1659 cm-1
Second freq. = 120.9149 cm-1

S	0.000000	0.000000	0.000000
N	-1.319899	0.230139	0.917814
C	-1.269875	-0.103452	2.229830
C	0.000000	0.000000	3.012702
C	1.275084	0.000000	2.258323
N	1.359599	-0.026810	0.982430
O	-0.018717	0.053977	4.220236
H	-2.158462	0.122674	2.792170
H	2.185112	0.003414	2.834864
O	-1.486126	-1.849516	2.020696
O	-0.421404	-2.412949	1.548234

Int($4a^H$-$5a^H$)' CASPT2//CASSCF

CASSCF energy = -845.687154
Enthalpy 0K = -845.621456
Enthalpy 298K = -845.613850
Free energy 298K = -845.653032
CASPT2 energy = -846.852336
Low freq. = 122.4108 cm-1
Second freq. = 243.4489 cm-1

S	0.000000	0.000000	0.000000
N	-1.318615	0.243362	0.906554
C	-1.291895	-0.205278	2.256718
C	0.000000	0.000000	3.021184
C	1.274765	0.000000	2.261168
N	1.359539	-0.036904	0.986462
O	-0.016772	0.141381	4.220033
H	-2.128751	0.195009	2.805268
H	2.183975	0.005687	2.839019
O	-1.596370	-1.649369	2.147786
O	-0.477300	-2.333272	1.824546

TS($4a^H$-$5a^H$)1' CASPT2//CASSCF

CASSCF energy = -845.677083
Enthalpy 0K = -845.611023
Enthalpy 298K = -845.604399
Free energy 298K = -845.641468
CASPT2 energy = -846.863596
Low freq. = -167.4008 cm-1
Second freq. = 246.3166 cm-1

S	0.000000	0.000000	0.000000
N	-1.161575	0.569496	0.836380
C	-1.247047	-0.107407	2.084929
C	0.000000	0.000000	2.930795
C	1.309341	0.000000	2.205911
N	1.433238	-0.050149	0.935256
O	-0.032001	0.052615	4.135480
H	-2.142643	0.118504	2.637834
H	2.200176	0.015348	2.813135
O	-1.415090	-1.510323	1.639632
O	-0.376135	-1.938603	0.805771

Int(4a^H-5a^H) CASPT2//CASSCF

CASSCF energy = -845.687365
Enthalpy 0K = -845.619176
Enthalpy 298K = -845.612476
Free energy 298K = -845.649629
CASPT2 energy = -846.867235
Low freq. = 237.6923 cm-1
Second freq. = 313.2541 cm-1

S	0.000000	0.000000	0.000000
N	-1.107403	0.658262	0.841342
C	-1.201343	-0.178400	1.995098
C	0.000000	0.000000	2.899374
C	1.326033	0.000000	2.192343
N	1.463913	-0.051540	0.922626
O	-0.057190	0.109956	4.097162
H	-2.131135	-0.050551	2.527337
H	2.211127	0.029618	2.808330
O	-1.176624	-1.560353	1.561838
O	-0.414113	-1.624556	0.265972

TS(4a^H-5a^H)2 CASPT2//CASSCF

CASSCF energy = -845.667913
Enthalpy 0K = -845.603349
Enthalpy 298K = -845.596169
Free energy 298K = -845.634296
CASPT2 energy = -846.850592
Low freq. = -481.4354 cm-1
Second freq. = 210.3276 cm-1

S	0.000000	0.000000	0.000000
N	-1.233395	0.398034	0.868198
C	-1.227006	-0.106177	2.109944
C	0.000000	0.000000	2.951555
C	1.293060	0.000000	2.213994
N	1.401541	-0.034363	0.939449
O	-0.033884	0.060829	4.154887
H	-2.156957	-0.062495	2.648929
H	2.195371	0.018327	2.802908
O	-0.923756	-1.878187	1.805901
O	-0.329020	-2.042239	0.576726

5a^H CASPT2//CASSCF

CASSCF energy = -845.829551
Enthalpy 0K = -845.760797
Enthalpy 298K = -845.753393
Free energy 298K = -845.792216
CASPT2 energy = -847.009821
Low freq. = 184.2835 cm-1
Second freq. = 216.2081 cm-1

S	0.000000	0.000000	0.000000
N	-0.843254	0.007602	1.515293
C	-2.244507	-0.052396	1.535499
C	0.000000	0.000000	2.622982
C	1.391576	0.000000	2.076080
N	1.514154	-0.012191	0.811357
O	-0.291690	0.007040	3.779404
H	-2.676183	-0.081711	0.540069
H	2.244558	0.004375	2.730969
O	-2.901586	-0.069665	2.529437
O	-0.188998	-1.303323	-0.657571

4a CASPT2//CASSCF

CASSCF energy = -1155.186421
Enthalpy 0K = -1154.951217
Enthalpy 298K = -1154.937923
Free energy 298K = -1154.990071
CASPT2 energy = -1157.6270690
Low freq. = 94.1489 cm-1
Second freq. = 127.4929 cm-1

S	0.000000	0.000000	0.000000
N	-1.331719	-0.103265	0.977331
C	-1.283887	-0.117351	2.261632
C	0.000000	0.000000	3.025506
C	1.289241	0.000000	2.261633
N	1.335590	0.018383	0.977332
O	-0.005708	0.125141	4.229544
C	-2.584213	-0.195556	2.995172
C	2.591286	0.040482	2.995170
C	3.627826	0.811567	2.478908
C	2.812659	-0.704149	4.148493
C	4.050275	-0.680246	4.768625
C	5.074478	0.095290	4.252576
C	4.860014	0.843109	3.106174
C	-3.686633	0.477987	2.478918
C	-4.916577	0.397238	3.106185
C	-5.062083	-0.367010	4.252579
C	-3.971540	-1.046110	4.768619
C	-2.736887	-0.957258	4.148486
H	2.026919	-1.303042	4.566349
H	4.210958	-1.266052	5.656074
H	6.032916	0.117528	4.740488
H	5.650243	1.449114	2.700031
H	3.464815	1.382922	1.584106
H	-1.899896	-1.482152	4.566334
H	-4.078234	-1.644120	5.656062
H	-6.018566	-0.432108	4.740492
H	-5.758685	0.928805	2.700049
H	-3.576306	1.061817	1.584122

TS(4a-5a)1 CASPT2//CASSCF

CASSCF energy = -1304.785335
Enthalpy 0K = -1304.543947
Enthalpy 298K = -1304.529242
Free energy 298K = -1304.584137
CASPT2 energy = -1307.526191
Low freq. = -432.3861 cm-1
Second freq. = 96.6215 cm-1

S	0.000000	0.000000	0.000000
N	-1.241109	0.355650	0.867950
C	-1.248883	-0.156257	2.107108
C	0.000000	0.000000	2.940685
C	1.315826	0.000000	2.202369
N	1.387535	-0.005149	0.918144
O	-0.039956	0.124038	4.137592
C	-2.568146	-0.190641	2.807019
C	2.597807	0.045818	2.960242
O	-0.695284	-1.932548	1.773216
O	-0.284813	-2.017532	0.470514
C	3.654175	0.792255	2.446831
H	3.518673	1.343525	1.535064
C	4.870744	0.827012	3.102811
H	5.677362	1.414380	2.702135
C	5.048008	0.103545	4.271139
H	5.994947	0.127047	4.780836
C	4.004532	-0.650109	4.781954
H	4.139762	-1.217772	5.684984
C	2.780895	-0.674875	4.135949
H	1.979140	-1.257398	4.544917
C	-2.778931	-1.039613	3.888249
H	-1.986654	-1.671837	4.240328
C	-4.013648	-1.082641	4.508102
H	-4.169507	-1.748083	5.338232
C	-5.045214	-0.270854	4.062824
H	-6.003714	-0.301818	4.550179
C	-4.839556	0.577369	2.988897
H	-5.636312	1.208395	2.637511
C	-3.607337	0.613529	2.357998
H	-3.451440	1.261861	1.517066

Int(4a-5a) CASPT2//CASSCF

CASSCF energy = -1304.801991
Enthalpy 0K = -1304.557809
Enthalpy 298K = -1304.543402
Free energy 298K = -1304.597690
CASPT2 energy = -1307.536664
Low freq. = 93.3540 cm-1
Second freq. = 113.6739 cm-1

S	0.000000	0.000000	0.000000
N	-1.145773	0.560742	0.857501
C	-1.202838	-0.296435	2.000332
C	0.000000	0.000000	2.899100
C	1.350435	0.000000	2.188882
N	1.444874	-0.023862	0.908274
O	-0.078371	0.244118	4.072972
C	-2.536830	-0.280287	2.707180
C	2.611121	0.049652	2.980680
O	-0.959537	-1.678539	1.547307
O	-0.335093	-1.646979	0.185931
C	3.703406	0.739750	2.461953
H	3.609052	1.246176	1.519775
C	4.900182	0.775645	3.152253
H	5.734731	1.319180	2.747007
C	5.022498	0.110384	4.362098
H	5.954418	0.135425	4.898643
C	3.943569	-0.585716	4.879796
H	4.035313	-1.106734	5.815769
C	2.739249	-0.611446	4.197829
H	1.910178	-1.148629	4.615060
C	-2.808461	-1.218686	3.696692
H	-2.069384	-1.952304	3.963696
C	-4.032243	-1.218849	4.337959
H	-4.237033	-1.951346	5.098173
C	-4.990982	-0.274184	4.003019
H	-5.942584	-0.271901	4.504758
C	-4.720687	0.663583	3.023249
H	-5.461224	1.397430	2.759083
C	-3.496301	0.660589	2.371989
H	-3.287087	1.378993	1.603600

TS(4a-5a)3 CASPT2//CASSCF

CASSCF energy = -1304.791013
Enthalpy 0K = -1304.551688
Enthalpy 298K = -1304.536174
Free energy 298K = -1304.593180
CASPT2 energy = -1307.516387
Low freq. = -557.2681 cm-1
Second freq. = 70.6267 cm-1

S	0.000000	0.000000	0.000000
N	-1.314722	0.222645	0.903524
C	-1.298419	-0.106870	2.225207
C	0.000000	0.000000	3.004005
C	1.301207	0.000000	2.253627
N	1.353790	-0.009596	0.971854
O	-0.018285	0.080695	4.208025
C	-2.567283	0.204624	2.962794
C	2.594276	0.040266	2.999357
O	-1.427112	-1.855812	1.948218
O	-0.334306	-2.370405	1.478048
C	3.626776	0.830470	2.505067
H	3.466544	1.414873	1.618043
C	4.852472	0.864524	3.145137
H	5.640587	1.484646	2.756809
C	5.062501	0.100273	4.281270
H	6.015771	0.124190	4.779043
C	4.041874	-0.695248	4.774364
H	4.201050	-1.295192	5.652479
C	2.811009	-0.722510	4.141827
H	2.028677	-1.340489	4.537545
C	-2.912342	-0.491361	4.116962
H	-2.264140	-1.255476	4.500043
C	-4.094937	-0.206411	4.773596
H	-4.355793	-0.754239	5.661290
C	-4.939260	0.781410	4.291268
H	-5.857041	1.004152	4.806211
C	-4.600013	1.477609	3.144705
H	-5.251881	2.243091	2.763355
C	-3.420942	1.186835	2.478542
H	-3.167348	1.717343	1.580710

Int(4a-5a)' CASPT2//CASSCF

CASSCF energy = -1304.798240
Enthalpy 0K = -1304.556562
Enthalpy 298K = -1304.541267
Free energy 298K = -1304.597495
CASPT2 energy = -1307.521760
Low freq. = 84.4184 cm-1
Second freq. = 102.3610 cm-1

S	0.000000	0.000000	0.000000
N	-1.314570	0.259755	0.888826
C	-1.321401	-0.176729	2.243213
C	0.000000	0.000000	3.012406
C	1.301363	0.000000	2.258108
N	1.353240	-0.020399	0.977367
O	-0.019959	0.127709	4.210446
C	-2.531831	0.342510	2.987208
C	2.593560	0.033272	3.005700
O	-1.560187	-1.645980	2.062231
O	-0.413403	-2.282200	1.754166
C	3.633471	0.812640	2.509881
H	3.479824	1.393938	1.619659
C	4.857972	0.839731	3.152647
H	5.652046	1.451428	2.763052
C	5.059214	0.079496	4.293047
H	6.011553	0.098025	4.792843
C	4.031018	-0.705246	4.787757
H	4.183361	-1.302303	5.669053
C	2.801549	-0.725616	4.152316
H	2.013781	-1.336623	4.548687
C	-2.990252	-0.319105	4.120907
H	-2.491108	-1.203938	4.468006
C	-4.092948	0.157221	4.804140
H	-4.445127	-0.363787	5.676269
C	-4.739820	1.303655	4.368343
H	-5.596476	1.674582	4.902738
C	-4.283378	1.966434	3.243638
H	-4.783396	2.853356	2.897804
C	-3.184372	1.484584	2.549755
H	-2.847180	1.990481	1.665380

TS(4a-5a)3' CASPT2//CASSCF

CASSCF energy = -1304.793047
Enthalpy 0K = -1304.550647
Enthalpy 298K = -1304.536413
Free energy 298K = -1304.590304
CASPT2 energy = -1307.535034
Low freq. = -153.2328 cm-1
Second freq. = 100.0366 cm-1

S	0.000000	0.000000	0.000000
N	-1.146364	0.642479	0.800139
C	-1.289345	0.029172	2.073917
C	0.000000	0.000000	2.910087
C	1.337856	0.000000	2.194159
N	1.424336	-0.035782	0.914734
O	-0.043501	-0.033589	4.113039
C	-2.504107	0.504545	2.831195
C	2.608766	0.060618	2.972731
O	-1.573438	-1.396022	1.661209
O	-0.447491	-1.852651	0.968948
C	3.643945	0.855272	2.489037
H	3.497348	1.424764	1.590160
C	4.853298	0.914419	3.156818
H	5.642743	1.538598	2.778129
C	5.045275	0.168248	4.308260
H	5.986422	0.210653	4.827359
C	4.023574	-0.633111	4.789424
H	4.170284	-1.219092	5.678928
C	2.806917	-0.683822	4.131280
H	2.023436	-1.304681	4.518201
C	-3.047278	-0.275994	3.845323
H	-2.601834	-1.220518	4.094429
C	-4.165287	0.158651	4.532325
H	-4.583671	-0.453710	5.310967
C	-4.742430	1.379972	4.220253
H	-5.610576	1.718350	4.757894
C	-4.201221	2.160455	3.214479
H	-4.646914	3.106913	2.965261
C	-3.086595	1.722648	2.516645
H	-2.677771	2.319396	1.724103

5a CASPT2//CASSCF

CASSCF energy = -1304.949432
Enthalpy 0K = -1304.704310
Enthalpy 298K = -1304.688950
Free energy 298K = -1304.745895
CASPT2 energy = -1307.682825
Low freq. = 58.5191 cm-1
Second freq. = 89.5602 cm-1

S	0.000000	0.000000	0.000000
N	-0.864015	0.064476	1.544949
C	-2.240891	-0.287560	1.611640
C	0.000000	0.000000	2.625142
C	1.410419	0.000000	2.082194
N	1.501399	-0.021770	0.787012
O	-0.311672	-0.005058	3.778850
C	-3.132092	0.328635	0.585778
C	2.625864	-0.013843	2.929169
O	-2.636825	-1.038958	2.454042
O	-0.215885	-1.306195	-0.581517
C	3.817249	-0.482736	2.376480
H	3.824535	-0.839576	1.363885
C	4.976054	-0.492613	3.126327
H	5.888643	-0.862615	2.694871
C	4.960502	-0.026878	4.433411
H	5.863918	-0.033902	5.017231
C	3.782986	0.443234	4.986315
H	3.767483	0.804618	5.998712
C	2.614438	0.447085	4.241588
H	1.705327	0.801096	4.684048

C	-4.237061	-0.398100	0.155182
H	-4.400632	-1.385797	0.543928
C	-5.108722	0.149923	-0.765887
H	-5.955591	-0.418360	-1.105779
C	-4.890969	1.431910	-1.249996
H	-5.572146	1.858324	-1.964819
C	-3.801348	2.164793	-0.812641
H	-3.638531	3.162729	-1.177840
C	-2.918709	1.612971	0.101546
H	-2.082369	2.191323	0.449315

4a^{H} CASPT2//UB3LYP

UB3LYP energy = -698.374298
Enthalpy 0K = -698.321498
Enthalpy 298K = -698.315046
Free energy 298K = -698.351356
CASPT2 energy = -696.962774
Low freq. = 118.8419 cm-1
Second freq. = 398.7267 cm-1

S	0.000000	0.000000	0.000000
N	-1.357422	-0.000980	0.961829
C	-1.256672	-0.000768	2.253762
C	0.000000	0.000000	3.034909
C	1.256574	0.000000	2.253660
N	1.356760	0.000093	0.961651
O	-0.000026	0.000668	4.261914
H	-2.183929	-0.000538	2.829014
H	2.183975	0.000298	2.828510

$^{1}O_2$ CASPT2//UB3LYP

UB3LYP energy = -150.254025
Enthalpy 0K = -150.250283
Enthalpy 298K = -150.246976
CASPT2 energy = -149.901954
Free energy 298K = -150.269226
Low freq. = 1642.3236 cm-1

O	1.215387	0.000000	0.000000
O	0.000000	0.000000	0.000000

TS(4a^{H}-5a^{H})1 CASPT2//UB3LYP

UB3LYP energy = -848.637987
Enthalpy 0K = -848.580768
Enthalpy 298K = -848.572059
Free energy 298K = -848.613966
CASPT2 energy = -846.844912
Low freq. = -389.1812 cm-1
Second freq. = 84.2009 cm-1

S	0.000000	0.000000	0.000000
N	-1.347141	0.097864	0.890229
C	-1.273133	-0.268169	2.217667
C	0.000000	0.000000	3.002652
C	1.268707	0.000000	2.237990
N	1.373714	-0.030910	0.945518
O	-0.024055	0.180393	4.208311
H	-2.174975	-0.043897	2.787163
H	2.186793	0.054445	2.823952
O	-1.359855	-1.937717	2.288733
O	-0.494767	-2.509364	1.533763

Int(4a^{H}-5a^{H})’ CASPT2//UB3LYP

UB3LYP energy = -848.639366
Enthalpy 0K = -848.580787
Enthalpy 298K = -848.571964
Free energy 298K = -848.613885
CASPT2 energy = -846.853208
Low freq. = 89.2277 cm-1
Second freq. = 120.8588 cm-1

S	0.000000	0.000000	0.000000
N	-1.346381	0.144065	0.867937
C	-1.295200	-0.282498	2.227436
C	0.000000	0.000000	3.000724
C	1.270621	0.000000	2.237912
N	1.379564	-0.030076	0.947073
O	-0.022769	0.197197	4.202632
H	-2.161792	0.080030	2.783180
H	2.186503	0.045874	2.828372
O	-1.498943	-1.772607	2.246161
O	-0.454421	-2.402833	1.751141

TS(4a^{H}-5a^{H})1’ CASPT2//UB3LYP

UB3LYP energy = -848.635398
Enthalpy 0K = -848.576173
Enthalpy 298K = -848.568450
Free energy 298K = -848.607798
CASPT2 energy = -846.866207
Low freq. = -257.9873 cm-1
Second freq. = 102.8369 cm-1

S	0.000000	0.000000	0.000000
N	-1.248946	0.511744	0.831964
C	-1.289884	-0.106306	2.137064
C	0.000000	0.000000	2.960907
C	1.303289	0.000000	2.218212
N	1.442218	-0.039008	0.942132
O	-0.022991	0.069136	4.172990
H	-2.153045	0.224046	2.715593
H	2.201613	0.023163	2.839547
O	-1.543626	-1.504255	1.791842
O	-0.438461	-2.005499	1.209025

Int(4a^{H}-5a^{H}) CASPT2//UB3LYP

UB3LYP energy = -848.643316
Enthalpy 0K = -848.583340
Enthalpy 298K = -848.575223
Free energy 298K = -848.615405
CASPT2 energy = -846.869760

Low freq. = 87.0322 cm-1
Second freq. = 156.1205 cm-1

S	0.000000	0.000000	0.000000
N	-1.174091	0.614109	0.835966
C	-1.203281	-0.213348	2.014675
C	0.000000	0.000000	2.930042
C	1.334232	0.000000	2.218897
N	1.507512	-0.047422	0.949724
O	-0.060761	0.100514	4.137003
H	-2.149707	-0.136971	2.554651
H	2.216609	0.054446	2.863721
O	-1.080324	-1.631776	1.591852
O	-0.360916	-1.679471	0.325052

TS(4a^H-5a^H)2 CASPT2//UB3LYP

UB3LYP energy = -848.631929
Enthalpy 0K = -848.573674
Enthalpy 298K = -848.565497
Free energy 298K = -848.605659
CASPT2 energy = -846.852458
Low freq. = -235.0686 cm-1
Second freq. = 127.2279 cm-1

S	0.000000	0.000000	0.000000
N	-1.309574	0.344387	0.866055
C	-1.246923	-0.078119	2.128348
C	0.000000	0.000000	2.969982
C	1.280467	0.000000	2.216358
N	1.406112	-0.006559	0.927578
O	-0.041087	0.054284	4.185235
H	-2.182150	-0.112532	2.684767
H	2.194450	0.025244	2.812639
O	-0.631511	-1.919886	1.826542
O	-0.295103	-2.004441	0.569508

TS(4a^H-5a^H)3 CASPT2//UB3LYP

UB3LYP energy = -848.618671
Enthalpy 0K = -848.560994
Enthalpy 298K = -848.553123
Free energy 298K = -848.592579
CASPT2 energy = -846.838923
Low freq. = -562.4601 cm-1
Second freq. = 112.3872 cm-1

S	0.000000	0.000000	0.000000
N	-1.085217	0.495385	1.026676
C	-1.130531	-0.608656	2.047041
C	0.000000	0.000000	2.946905
C	1.356371	0.000000	2.184370
N	1.580888	0.007480	0.934221
O	-0.112688	0.386015	4.074861
H	-2.137544	-0.651235	2.480304
H	2.223483	-0.039544	2.849796
O	-0.611342	-1.798265	1.646993
O	-0.215945	-1.546967	-0.140641

5a^H CASPT2//UB3LYP

UB3LYP energy = -848.791391
Enthalpy 0K = -848.731105
Enthalpy 298K = -848.721988
Free energy 298K = -848.764859
CASPT2 energy = -847.012800
Low freq. = 66.0048 cm-1
Second freq. = 120.9402 cm-1

S	0.000000	0.000000	0.000000
N	-0.869387	-0.035880	1.576117
C	-2.277164	-0.117176	1.614409
C	0.000000	0.000000	2.677824
C	1.398701	0.000000	2.103264
N	1.557382	-0.044107	0.839714
O	-0.272784	0.037191	3.852340
H	-2.707994	-0.154466	0.597879
H	2.251902	0.022715	2.781318
O	-2.931434	-0.149924	2.623362
O	-0.202418	-1.286765	-0.694285

4a CASPT2//UB3LYP(PCM)

UB3LYP energy = -1160.501750
Enthalpy 0K = -1160.286766
Enthalpy 298K = -1160.271080
Free energy 298K = -1160.330680
CASPT2 energy = -1157.632268
Low freq. = 21.2046 cm-1
Second freq. = 39.3897 cm-1

S	0.000000	0.000000	0.000000
N	-1.336787	-0.103654	0.945537
C	-1.280774	-0.115674	2.254350
C	0.000000	0.000000	3.020139
C	1.285988	0.000000	2.254349
N	1.340692	0.017020	0.945536
O	-0.005274	0.117019	4.246307
C	-2.582321	-0.198913	2.967685
C	2.589748	0.034227	2.967680
C	3.689942	0.638919	2.331070
C	2.780385	-0.557706	4.229441
C	4.040018	-0.546352	4.828306
C	5.122025	0.063668	4.191229
C	4.941660	0.658498	2.939130
C	-3.732471	0.304328	2.331087
C	-4.980867	0.211188	2.939158
C	-5.106964	-0.397463	4.191256
C	-3.974448	-0.907645	4.828322
C	-2.718908	-0.805604	4.229447
H	1.948629	-1.026362	4.737148
H	4.173437	-1.015189	5.798742
H	6.098315	0.078330	4.666905
H	5.776236	1.139712	2.437851
H	3.547926	1.096953	1.358235
H	-1.848349	-1.197511	4.737145

H	-4.065129	-1.386587	5.798757
H	-6.080608	-0.470713	4.666941
H	-5.855364	0.615353	2.437888
H	-3.632255	0.773288	1.358254

$^1O_2$ CASPT2//UB3LYP(PCM)

UB3LYP energy = -150.254547110
Enthalpy 0K = -150.250809
Enthalpy 298K = -150.247501
Free energy 298K = -150.269752
CASPT2 energy = -149.901954
Low freq. = 1640.9332 cm-1

O	1.215387	0.000000	0.000000
O	0.000000	0.000000	0.000000

TS(4a-5a)1 CASPT2//UB3LYP(PCM)

UB3LYP energy = -1310.765615
Enthalpy 0K = -1310.545307
Enthalpy 298K = -1310.527809
Free energy 298K = -1310.591146
CASPT2 energy = -1307.537536
Low freq. = -142.8845 cm-1
Second freq. = 20.3808 cm-1

S	0.000000	0.000000	0.000000
N	-1.290809	0.404387	0.846477
C	-1.277088	-0.038709	2.116130
C	0.000000	0.000000	2.947676
C	1.318740	0.000000	2.207977
N	1.392719	0.018605	0.899923
O	-0.051432	0.053361	4.165026
C	-2.585304	-0.088928	2.811208
C	2.599313	0.040312	2.943483
O	-0.610778	-1.898272	1.751988
O	-0.289110	-1.910667	0.460569
C	3.719931	0.609743	2.304544
H	3.599753	1.040056	1.316654
C	4.958608	0.632998	2.935151
H	5.809065	1.087380	2.436329
C	5.106094	0.073861	4.208473
H	6.073923	0.089831	4.700826
C	4.005910	-0.503386	4.846327
H	4.118214	-0.945818	5.831294
C	2.757318	-0.515982	4.228236
H	1.911666	-0.962079	4.732141
C	-2.781333	-0.882280	3.955036
H	-1.961934	-1.462282	4.359024
C	-4.034837	-0.938382	4.560371
H	-4.177939	-1.562256	5.437351
C	-5.101845	-0.198649	4.044395
H	-6.075181	-0.239884	4.524397
C	-4.914068	0.591332	2.907639
H	-5.739128	1.168098	2.500790
C	-3.667319	0.639785	2.288680
H	-3.518557	1.245834	1.402193

Int(4a-5a) CASPT2//UB3LYP(PCM)

UB3LYP energy = -1310.771684
Enthalpy 0K = -1310.550232
Enthalpy 298K = -1310.532725
Free energy 298K = -1310.595916
CASPT2 energy = -1307.542275
Low freq. = 24.1348 cm-1
Second freq. = 38.4833 cm-1

S	0.000000	0.000000	0.000000
N	-1.228533	0.471995	0.856292
C	-1.201558	-0.358553	2.025324
C	0.000000	0.000000	2.921726
C	1.360646	0.000000	2.205842
N	1.461085	-0.008848	0.909298
O	-0.097623	0.266637	4.100015
C	-2.526033	-0.459816	2.735605
C	2.607100	0.068645	2.993147
O	-0.797194	-1.772401	1.587644
O	-0.276968	-1.724850	0.239841
C	3.752874	0.633538	2.397764
H	3.675749	1.045647	1.397821
C	4.958551	0.674846	3.088191
H	5.830840	1.125057	2.624641
C	5.045080	0.139852	4.377903
H	5.987437	0.170779	4.916676
C	3.918552	-0.432281	4.973148
H	3.984951	-0.854758	5.970825
C	2.702995	-0.464232	4.293321
H	1.837524	-0.909969	4.765101
C	-2.681583	-1.364568	3.795632
H	-1.846716	-1.980666	4.113089
C	-3.911717	-1.480066	4.440469
H	-4.026774	-2.186483	5.257002
C	-4.990514	-0.686991	4.040171
H	-5.947068	-0.774481	4.547063
C	-4.835363	0.218193	2.988816
H	-5.670298	0.837260	2.674314
C	-3.607585	0.330803	2.334584
H	-3.480483	1.026051	1.512801

TS(4a-5a)3 CASPT2//UB3LYP(PCM)

UB3LYP energy = -1310.762716
Enthalpy 0K = -1310.543875
Enthalpy 298K = -1310.525904
Free energy 298K = -1310.590430
CASPT2 energy = -1307.520785
Low freq. = -270.0198 cm-1
Second freq. = 20.4200 cm-1

S	0.000000	0.000000	0.000000
N	-1.339617	0.200242	0.857740
C	-1.326049	-0.146019	2.206850
C	0.000000	0.000000	2.996488
C	1.300696	0.000000	2.243375

N	1.349154	-0.046392	0.932109
O	-0.036089	0.056100	4.213680
C	-2.564822	0.288084	2.941865
C	2.595658	0.008759	2.957339
O	-1.482652	-1.808491	2.208741
O	-0.501464	-2.399815	1.610915
C	3.712400	-0.568408	2.316704
H	3.579577	-1.028044	1.343795
C	4.960754	-0.566299	2.927720
H	5.806010	-1.028075	2.426553
C	5.125523	0.024168	4.185169
H	6.101200	0.027536	4.661998
C	4.030829	0.609468	4.824112
H	4.154281	1.078390	5.795553
C	2.773081	0.602039	4.223193
H	1.935582	1.063034	4.727296
C	-2.939810	-0.323249	4.148246
H	-2.335773	-1.120482	4.562995
C	-4.096781	0.087485	4.808734
H	-4.383305	-0.396033	5.737784
C	-4.882959	1.114563	4.280772
H	-5.781438	1.434343	4.800482
C	-4.511501	1.726478	3.082180
H	-5.118603	2.524121	2.664675
C	-3.359935	1.312992	2.412496
H	-3.071913	1.779569	1.477191

Int(4a-5a)' CASPT2//UB3LYP(PCM)

UB3LYP energy = -1310.763318
Enthalpy 0K = -1310.543468
Enthalpy 298K = -1310.525240
Free energy 298K = -1310.590235
CASPT2 energy = -1307.525495
Low freq. = 19.6856 cm-1
Second freq. = 40.6609 cm-1

S	0.000000	0.000000	0.000000
N	-1.326482	0.311490	0.834789
C	-1.348504	-0.077922	2.204423
C	0.000000	0.000000	2.996750
C	1.303177	0.000000	2.245608
N	1.351771	-0.066437	0.936910
O	-0.039165	-0.002466	4.213209
C	-2.517732	0.533284	2.940817
C	2.593758	0.007512	2.965438
O	-1.647194	-1.584329	2.166466
O	-0.602554	-2.267155	1.746893
C	3.714257	-0.575289	2.337088
H	3.587536	-1.043324	1.367375
C	4.958355	-0.566829	2.956638
H	5.807408	-1.032659	2.465817
C	5.114116	0.035747	4.209589
H	6.086575	0.043989	4.692856
C	4.015213	0.626855	4.836178
H	4.132289	1.104597	5.804047
C	2.761825	0.612890	4.226839
H	1.920748	1.078345	4.721426
C	-3.001588	-0.054367	4.118490
H	-2.543189	-0.958233	4.501123
C	-4.078985	0.519760	4.791966
H	-4.453497	0.054912	5.698970
C	-4.673057	1.686537	4.304417
H	-5.510235	2.132695	4.833259
C	-4.189999	2.275158	3.134378
H	-4.649012	3.180315	2.748276
C	-3.118217	1.699040	2.451434
H	-2.747326	2.146059	1.535760

TS(4a-5a)3' CASPT2//UB3LYP(PCM)

UB3LYP energy = -1310.762168
Enthalpy 0K = -1310.541840
Enthalpy 298K = -1310.524651
Free energy 298K = -1310.586950
CASPT2 energy = -1307.535290
Low freq. = -167.5776 cm-1
Second freq. = 25.9368 cm-1

S	0.000000	0.000000	0.000000
N	-1.222141	0.623795	0.797633
C	-1.331343	0.129373	2.143901
C	0.000000	0.000000	2.959012
C	1.329289	0.000000	2.227053
N	1.390533	-0.093803	0.929495
O	-0.058219	-0.176354	4.158314
C	-2.440567	0.803516	2.911512
C	2.597317	0.017768	2.981235
O	-1.745471	-1.280187	1.890968
O	-0.695550	-1.955902	1.397843
C	3.745003	-0.546201	2.387046
H	3.655820	-1.014247	1.413143
C	4.967411	-0.518296	3.047340
H	5.840103	-0.969284	2.585217
C	5.071374	0.086140	4.305098
H	6.027400	0.109458	4.819573
C	3.944183	0.658985	4.897497
H	4.022632	1.136412	5.869230
C	2.711920	0.623871	4.247762
H	1.847636	1.074963	4.716239
C	-3.043278	0.153276	3.997074
H	-2.719725	-0.841784	4.278626
C	-4.064246	0.784987	4.706215
H	-4.533677	0.273477	5.541069
C	-4.480804	2.069079	4.345911
H	-5.274167	2.559494	4.902378
C	-3.877708	2.718937	3.267344
H	-4.199705	3.715534	2.980714
C	-2.862450	2.087244	2.547960
H	-2.399378	2.581937	1.701191

TS(4a-5a)2 CASPT2//UB3LYP(PCM)

UB3LYP energy = -1310.752098
Enthalpy 0K = -1310.532726
Enthalpy 298K = -1310.515458

Free energy 298K = -1310.578407
CASPT2 energy = -1307.517498
Low freq. = -981.7913 cm-1
Second freq. = 22.7519 cm-1

S	0.000000	0.000000	0.000000
N	-1.120185	0.399655	1.043061
C	-1.110535	-0.729191	2.036670
C	0.000000	0.000000	2.892541
C	1.383710	0.000000	2.153273
N	1.530015	0.032599	0.870388
O	-0.180458	0.529802	3.955233
C	-2.460809	-0.949211	2.669313
C	2.592353	-0.059767	2.997256
O	-0.481271	-1.865095	1.579951
O	-0.177959	-1.572423	-0.155486
C	3.832017	0.313376	2.436915
H	3.861597	0.662110	1.411062
C	4.993271	0.250026	3.195832
H	5.939655	0.552682	2.758838
C	4.942244	-0.199843	4.520477
H	5.851533	-0.250920	5.111785
C	3.722562	-0.581150	5.081450
H	3.680296	-0.936160	6.106144
C	2.550296	-0.508883	4.331113
H	1.614156	-0.810812	4.782023
C	-2.754369	-2.196842	3.234001
H	-2.019087	-2.993527	3.182238
C	-3.988397	-2.415666	3.845771
H	-4.213185	-3.388018	4.273918
C	-4.930333	-1.385791	3.909008
H	-5.889767	-1.555058	4.389163
C	-4.635836	-0.137780	3.354198
H	-5.365986	0.664803	3.400485
C	-3.405613	0.081620	2.734216
H	-3.177279	1.043650	2.289294

5a CASPT2//UB3LYP(PCM)

UB3LYP energy = -1310.933416
Enthalpy 0K = -1310.710447
Enthalpy 298K = -1310.692246
Free energy 298K = -1310.758016
CASPT2 energy = -1307.690251
Low freq. = 23.2345 cm-1
Second freq. = 26.9678 cm-1

S	0.000000	0.000000	0.000000
N	-0.874191	-0.008951	1.578397
C	-2.273808	-0.296780	1.684518
C	0.000000	0.000000	2.662142
C	1.426104	0.000000	2.098513
N	1.516691	-0.061080	0.810202
O	-0.314178	0.053901	3.832428
C	-3.149522	0.290724	0.636067
C	2.626300	0.061099	2.941058
O	-2.686490	-0.995260	2.587752
O	-0.221113	-1.302236	-0.673056
C	3.868300	0.318794	2.323055
H	3.898825	0.483412	1.251777
C	5.032270	0.369182	3.078706
H	5.982000	0.573457	2.594358
C	4.978888	0.159827	4.461960
H	5.889884	0.199077	5.051788
C	3.755532	-0.098359	5.082480
H	3.712936	-0.264600	6.154291
C	2.580960	-0.147449	4.333353
H	1.634729	-0.347923	4.816563
C	-4.342247	-0.385739	0.331948
H	-4.559148	-1.320335	0.837860
C	-5.220261	0.138157	-0.611665
H	-6.133678	-0.395227	-0.855431
C	-4.926517	1.351785	-1.242489
H	-5.614966	1.762062	-1.975186
C	-3.753743	2.041065	-0.926989
H	-3.535070	2.992161	-1.402074
C	-2.861994	1.512437	0.006066
H	-1.966912	2.066387	0.267410

S4. Synthesis and Characterization

S4.1 Preparation of 4*H*-1,2,6-thiadiazine starting materials

3,5-Dimethoxy-4*H*-1,2,6-thiadiazin-4-one (**4r**),[40] 3,5-diphenoxy-4*H*-1,2,6-thiadiazin-4-one (**4t**),[40] 3-methoxy-5-phenyl-4*H*-1,2,6-thiadiazin-4-one (**4ab**),[41] 3,5-dichloro-4*H*-1,2,6-thiadiazin-4-one (**6a**),[40] 3-chloro-5-(phenylamino)-4*H*-1,2,6-thiadiazin-4-one (**6b**),[40] 3-chloro-5-phenoxy-4*H*-1,2,6-thiadiazin-4-one (**6c**),[40] 3-chloro-5-(phenylthio)-4*H*-1,2,6-thiadiazin-4-one (**6d**),[40] 3,5-diamino-4*H*-1,2,6-thiadiazin-4-one (**13a**),[40] 3,5-bis(phenyl-amino)-4*H*-1,2,6-thiadiazin-4-one (**13c**),[40] 3,5-bis(benzylamino)-4*H*-1,2,6-thiadiazin-4-one (**13b**),[26] 3,5-dimorpholino-4*H*-1,2,6-thiadiazin-4-one (**15a**),[26] 3,5-bis-[methyl(phenyl)amino]-4*H*-1,2,6-thiadiazin-4-one (**15b**),[40] 3-[(2-nitrophenyl)amino]-5-(phenylamino)-4*H*-1,2,6-thiadiazin-4-one (**13d**),[42] 3,5-diphenyl-4*H*-1,2,6-thiadiazine-4-thione (**19a**),[41] *N*,3,5-triphenyl-4*H*-1,2,6-thiadiazin-4-imine (**19b**),[43] 2-(3,5-diphenyl-4*H*-1,2,6-thiadiazin-4-ylidene)malononitrile (**19c**),[41] were prepared according to the reported procedures. 4-Spirocyclic thiadiazines **4u-4z** and 3',5'-bis(4-nitrophenyl)spiro[benzo[*d*][1,3]dioxole-2,4'-[1,2,6]thiadiazine] (**10**), were prepared according to the reported procedures.[44,45] 3,5-Bis(phenylethynyl)-4*H*-1,2,6-thiadiazin-4-one (**4q**) and symmetrical 3,5-diarylthiadiazinones (**4a-4p**) were prepared according to the reported procedure.[46]

S4.1.1 Preparation of symmetrical 3,5-diaryl-4*H*-1,2,6-thiadiazinones

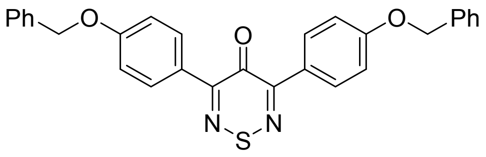

3,5-Bis[4-(benzyloxy)phenyl]-4*H*-1,2,6-thiadiazin-4-one (4k). A stirred solution of 3,5-dichloro-4*H*-1,2,6-thiadiazin-4-one (**6a**) (91.5 mg, 0.500 mmol), [4-(benzyloxy)phenyl]tributylstannane (520 mg, 1.10 mmol) and $Pd(Ph_3P)_2Cl_2$ (17.5 mg, 5 mol %) in PhMe (1 mL) was deaerated by bubbling of Ar through it for 5 min and then heated at *ca.* 110 °C under Ar until complete consumption of the starting thiadiazine (TLC, 1.5 h). The mixture was then allowed to cool to *ca.* 20 °C and the precipitate filtered to give the *title compound* **4k** (189 mg, 79%) as yellow plates, mp 214-215 °C (from PhMe); R_f 0.59 (*n*-hexane/DCM, 50:50); (found: C, 73.00; H, 4.37; N, 5.63. $C_{29}H_{22}N_2O_3S$ requires C, 72.78; H, 4.63; N, 5.85%); λ_{max}(DCM)/nm 255 (log ε 4.20), 270 inf (4.16), 382 (4.46); ν_{max}/cm^{-1} 3065w and 3051w (aryl C-H), 2947 (alkyl C-H), 1620s, 1597m, 1570w, 1558w, 1504m, 1468m, 1456m, 1412m, 1379m, 1337m, 1304m, 1240s, 1179s, 1138m, 1121w, 1084w, 1016m, 1005m, 991m, 928m, 866m, 839s, 785m, 756m, 743s; ^{1}H NMR ($CDCl_3$,) δ 8.08 (d, 4H, J = 8.1 Hz, Ar *H*), 7.49 (d, 4H, J = 7.5 Hz, Ar *H*), 7.42 (dd, 4H, J = 7.4, 7.4 Hz, Ar *H*), 7.36 (d, 2H, J = 9.0 Hz, Ar *H*), 7.13 (d, 4H, J = 7.1 Hz, Ar *H*), 5.21 (s, 4H, C*H*$_2$); ^{13}C NMR could not be recorded due to poor solubility; *m/z* (MALDI-TOF) calcd. for $C_{29}H_{23}N_2O_3S$ [M+H]$^+$: 479, Found: 479 (MH$^+$, 27%), 386 (32), 91 (100).

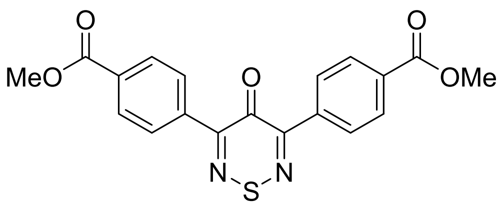

Dimethyl 4,4'-(4-oxo-4*H*-1,2,6-thiadiazine-3,5-diyl)di-benzoate (4l). To an intimate mixture of 3,5-dichloro-4*H*-1,2,6-thiadiazin-4-one (**6a**) (100 mg, 0.546 mmol), [4-(methoxycarbonyl)phenyl]-

boronic acid (216 mg, 1.20 mmol), $Pd(Ph_3P)_4$ (31.5 mg, 5 mol %), and Na_2CO_3 (115.7 mg, 1.092 mmol) was added dioxane (1 mL) and H_2O (0.6 mL) and the mixture heated to *ca.* 100 °C until complete consumption of the starting thiadiazine (TLC, 30 min). The mixture was then allowed to cool to *ca.* 20 °C, the precipitate filtered and washed with H_2O (5 mL), then EtOH (5 mL) and dried under vacuum to give the *title compound* **4l** (187 mg, 90%) as yellow needles, mp 234-235 °C (from DCE/*c*-hexane); R_f 0.76 (DCM); (found: C, 59.82; H, 3.67; N, 7.55. $C_{19}H_{14}N_2O_5S$ requires C, 59.68; H, 3.69; N, 7.33%); λ_{max}(DCM)/nm 258 (log ε 4.22), 356 (4.40); ν_{max}/cm^{-1} 1724s (C=O), 1616s, 1433m, 1400m, 1331w, 1307w, 1281s, 1259s, 1234m, 1188m, 1177m, 1107s, 1013m, 997w, 953m, 856m, 837w, 779m, 764m, 750s; ^1H NMR (CD_2Cl_2, 500 MHz) δ 8.23 (d, 4H, J = 8.6 Hz, Ar *H*), 8.12 (d, 4H, J = 8.6 Hz, Ar *H*), 3.93 (s, 6H, CH_3); δ_C(CD_2Cl_2, 125 MHz) 166.7 (*C*q), 165.4 (*C*q), 160.7 (*C*q), 138.6 (*C*q), 132.8 (*C*q), 129.7 (*C*H), 129.3 (*C*H), 52.6 (CH_3); *m/z* (MALDI-TOF) calcd. for $C_{19}H_{15}N_2O_5S$ $[M+H]^+$: 383, Found: 383 (MH^+, 86%), 381 (M^+-H, 100), 353 (61), 341 (36).

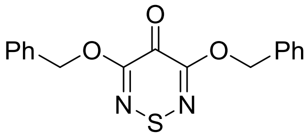

3,5-Bis(benzyloxy)-4*H*-1,2,6-thiadiazin-4-one (4s). To a stirred solution of 3,5-dichloro-4*H*-1,2,6-thiadiazin-4-one (**6a**) (183 mg, 1.00 mmol) in THF (5 mL), at *ca.* 20 °C, was added, in one portion, benzyl alcohol (227 μL, 2.00 mmol) and the mixture was cooled to *ca.* 0 °C. To this cooled mixture was added NaH 60% in paraffin oil (80 mg, 2.00 mmol) and the mixture was stirred at this temperature for 1 h, then allowed to warm to *ca.* 20 °C and stirred until complete consumption of the starting material (TLC, 24 h). The mixture was then adsorbed onto silica and chromatographed (*n*-hexane/DCM, 50:50) to give the *title compound* **4s** (301 mg, 92%) as colorless needles, mp 152-153 °C (from *c*-hexane); R_f 0.16 (*n*-hexane/DCM, 50:50); (found: C, 62.71; H, 4.36; N, 8.77. $C_{17}H_{14}N_2O_3S$ requires C, 62.56; H, 4.32; N, 8.58%); λ_{max}(DCM)/nm 271 (log ε 4.07), 359 (4.18); ν_{max}/cm^{-1} 3069w (aryl C-H), 2961 (alkyl C-H), 1639s, 1522m, 1454m, 1395w, 1319s, 1281m, 1221w, 1082s, 986s, 924w, 847w, 764m, 750m, 735s; ^1H NMR ($CDCl_3$, 500 MHz) δ 7.45-7.43 (m, 4H, Ar *H*), 7.38-7.31 (m, 6H, Ar *H*), 5.36 (s, 4H, CH_2); $^{13}C\{^1H\}$ NMR ($CDCl_3$, 125 MHz) δ 157.4 (*C*q), 155.7 (*C*q), 135.1 (*C*q), 128.54 (*C*H), 128.47 (*C*H), 128.43 (*C*H), 69.2 (CH_2); *m/z* (ES-API+) calcd. for $C_{17}H_{14}N_2O_3S$ $[M]^+$: 326, Found: 326 (M^+, 15%), 270 (M^+-H, 100), 118 (38).

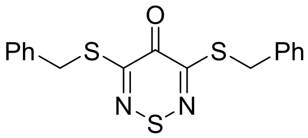

3,5-Bis(benzylthio)-4*H*-1,2,6-thiadiazin-4-one (9). To a stirred solution of 3,5-dichloro-4*H*-1,2,6-thiadiazin-4-one (**6a**) (183 mg, 1.00 mmol) in EtOH (5 mL), at *ca.* 20 °C, was added, in one portion, phenylmethanethiol (235 μL, 2.00 mmol) and the mixture was cooled to *ca.* 0 °C. To this cooled mixture was added NaH 60% in paraffin oil (80 mg, 2.00 mmol) and the mixture was stirred at this temperature for 1 h, then allowed to warm to *ca.* 20 °C and stirred until complete consumption of the starting material (TLC, 24 h). The mixture was then poured onto H_2O (10 mL), filtered and washed with *n*-hexane (5 mL) to give the *title compound* **9** (253 mg, 71%) as yellow needles, mp 91-92 °C (from *c*-hexane); R_f 0.19 (*n*-hexane/DCM, 70:30); (found: C, 56.80; H, 3.81; N, 7.73. $C_{17}H_{14}N_2OS_3$ requires C, 56.96; H, 3.94; N, 7.81%); λ_{max}(DCM)/nm 250 inf (log ε 4.30), 340 (4.01), 396 (4.07); ν_{max}/cm^{-1} 3055w and 3024w (aryl C-H), 2922w and 2853w (alkyl C-H), 1618s, 1493m, 1477w, 1452m, 1425w, 1391w, 1302m, 1242m, 1202w, 1074m, 1028w, 868w, 835w, 781m, 745s; ^1H NMR ($CDCl_3$, 500 MHz) δ 7.37 (d, 4H, J = 7.1 Hz, Ar *H*), 7.31 (dd, 4H, J = 7.0, 7.0 Hz, Ar *H*), 7.26 (dd, 2H, J = 6.4, 6.4 Hz, Ar

H), 4.20 (s, 4H, CH_2); $^{13}C\{^1H\}$ NMR ($CDCl_3$, 125 MHz) δ 160.7 (*C*q), 158.0 (*C*q), 136.0 (*C*q), 129.1 (*C*H), 128.6 (*C*H), 127.6 (*C*H), 33.8 (*C*H_2); *m/z* (MALDI-TOF) calcd. for $C_{17}H_{15}N_2OS_3$ $[M+H]^+$: 359, Found: 359 (MH^+, 29%), 358 (M^+, 42), 357 (M^+-H, 100), 180 (66), 153 (62), 90 (99).

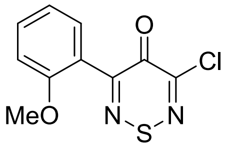

3-Chloro-5-(2-methoxyphenyl)-4*H*-1,2,6-thiadiazin-4-one (21). To an intimate mixture of 3,5-dichloro-4*H*-1,2,6-thiadiazin-4-one (**6a**) (100 mg, 0.546 mmol), (2-methoxyphenyl)boronic acid (83 mg, 0.546 mmol), $Pd(Ph_3P)_4$ (15.8 mg, 2.5 mol %), and Na_2CO_3 (57.9 mg, 0.546 mmol) was added dioxane (1 mL) and H_2O (0.6 mL) and the mixture heated to *ca*. 60 °C until complete consumption of the boronic acid (TLC, 30 min). The mixture was then allowed to cool to *ca*. 20 °C, adsorbed onto silica and chromatographed (*n*-hexane/DCM, 50:50) to give the *title compound* **21** (88.9 mg, 64%) as a yellow oil; R_f 0.18 (*n*-hexane/DCM, 50:50); (found: C, 47.28; H, 2.68; N, 10.97. $C_{10}H_7ClN_2O_2S$ requires C, 47.16; H, 2.77; N, 11.00%); λ_{max}(DCM)/nm 314 (log ε 4.16); ν_{max}/cm^{-1} 1655s, 1599m, 1497m, 1481m, 1437m, 1321m, 1300m, 1275m, 1250s, 1192m, 1182m, 1165m, 1117m, 1051m, 1022m, 1016s, 866m, 841m, 799s, 783m, 766s, 756s, 719s; ^1H NMR ($CDCl_3$, 500 MHz) δ 7.47 (ddd, 1H, J = 7.5, 7.5, 1.7 Hz, Ar *H*), 7.35 (dd, 1H, J = 7.6, 1.8 Hz, Ar *H*), 7.05 (ddd, 1H, J = 7.5, 7.5, 0.9 Hz, Ar *H*), 7.00 (d, 1H, J = 8.4 Hz, Ar *H*), 3.83 (s, 3H, CH_3); $^{13}C\{^1H\}$ NMR ($CDCl_3$, 125 MHz) δ 161.5 (*C*q), 159.9 (*C*q), 157.3 (*C*q), 151.8 (*C*q), 132.3 (*C*H), 130.0 (*C*H), 123.8 (*C*q), 120.8 (*C*H), 111.6 (*C*H), 55.9 (*C*H_3); *m/z* (MALDI-TOF) calcd. for $C_{10}H_8ClN_2O_2S$ $[M+H]^+$: 255, Found: 257 (MH^++2, 69%), 255 (MH^+, 100), 193 (40), 177 (34). A final elution (*n*-hexane/DCM, 50:50) gave 3,5-bis(2-methoxyphenyl)-4*H*-1,2,6-thiadiazin-4-one (**4e**) (25.5 mg, 14%) as yellow needles, mp 128-129 °C (from *c*-hexane) [lit. mp 128.5-129 °C],[46] R_f 0.47 (*n*-hexane/DCM, 50:50); ^1H NMR ($CDCl_3$, 500 MHz) δ 7.45-7.40 (4H, m, Ar *H*), 7.05 (2H, dd, *J* 7.5, 7.5, Ar *H*), 6.99 (2H, d, *J* 8.0, Ar *H*), 3.84 (6H, s, OCH_3), identical to an authentic sample.[46]

S4.1.2 Preparation of asymmetrical 3,5-diaryl-4*H*-1,2,6-thiadiazinones

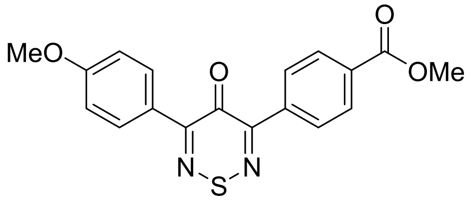

Methyl 4-[5-(4-methoxyphenyl)-4-oxo-4*H*-1,2,6-thiadiazin-3-yl]benzoate (4aa): (Typical procedure A, one pot). To an intimate mixture of 3,5-dichloro-4*H*-1,2,6-thiadiazin-4-one (**6a**) (100 mg, 0.546 mmol), [4-(methoxycarbonyl)phenyl]boronic acid (98.3 mg, 0.546 mmol), $Pd(Ph_3P)_4$ (15.8 mg, 2.5 mol %), and Na_2CO_3 (57.9 mg, 0.546 mmol) was added dioxane (1 mL) and water (0.6 mL) and the mixture heated at *ca.* 60 °C for 1 h. Then (4-methoxyphenyl)boronic acid (91.3 mg, 0.601 mmol), $Pd(Ph_3P)_4$ (15.8 mg, 2.5 mol %), and Na_2CO_3 (57.9 mg, 0.546 mmol) were added and the mixture heated at *ca.* 100 °C until complete consumption of the intermediate mono-arylthiadiazinone (TLC, 1 h). The mixture was allowed to cool to *ca*. 20 °C, adsorbed onto silica and chromatographed (*n*-hexane/DCM, 50:50) to give 3,5-bis(4-methoxyphenyl)-4*H*-1,2,6-thiadiazin-4-one (**4g**) (19.5 mg, 11%) as yellow needles, mp 169-171 °C (from *c*-hexane) [lit. mp 169-171 °C],[46] R_f 0.55 (*n*-hexane/DCM, 50:50); ^1H NMR ($CDCl_3$, 500 MHz) δ 8.22 (4H, d, J = 9.0 Hz, Ar *H*), 6.97 (4H, d, J = 8.5 Hz, Ar *H*), 3.87 (6H, s, OCH_3), identical to an authentic sample.[46] Further elution (*n*-hexane/DCM, 30:70) gave the *title compound* **4aa** (103.5 mg, 54%) as yellow plates, mp 186-187 °C (from

DCE/*c*-hexane); R_f 0.28 (*n*-hexane/DCM, 30:70); (found: C, 61.14; H, 3.83; N, 7.73. $C_{18}H_{14}N_2O_4S$ requires C, 61.01; H, 3.98; N, 7.91%); λ_{max}(DCM)/nm 251 (log ε 4.39), 376 (4.54); ν_{max}/cm^{-1} 2951w (alkyl C-H), 1715s, 1624s, 1601m, 1572w, 1564w, 1504m, 1452w, 1431m, 1412m, 1402m, 1331m, 1310m, 1283s, 1256s, 1177m, 1138w, 1109m, 1026m, 1016m, 997w, 961m, 870m, 843m, 808m, 766m, 743m; ^{1}H NMR ($CDCl_3$, 500 MHz) δ 8.26 (d, 2H, J = 8.9 Hz, Ar *H*), 8.22 (d, 2H, J = 8.6 Hz, Ar *H*), 8.12 (dd, 2H, J = 8.6 Hz, Ar *H*), 6.98 (d, 2H, J = 9.0 Hz, Ar *H*), 3.95 (s, 3H, CH_3), 3.89 (s, 3H, CH_3); ^{13}C{^{1}H} NMR ($CDCl_3$, 125 MHz) δ 166.5 (*C*q), 165.3 (*C*q), 162.2 (*C*q), 160.3 (*C*q), 159.2 (*C*q), 138.6 (*C*q), 131.8 (*C*q), 131.1 (*C*H), 129.4 (*C*H), 128.9 (*C*H), 127.0 (*C*q), 113.7 (*C*H), 55.4 (*C*H_3), 55.3 (*C*H_3); *m/z* (MALDI-TOF) calcd. for $C_{18}H_{15}N_2O_4S$ [M+H]$^+$: 355, Found: 355 (MH$^+$, 100%), 239 (65). A final elution (DCM) gave dimethyl 4,4'-(4-oxo-4*H*-1,2,6-thiadiazine-3,5-diyl)dibenzoate (**4l**) (37.1 mg, 18%), R_f 0.76 (DCM); δ_H(CD_2Cl_2, 500 MHz) 8.23 (4H, d, J = 8.6 Hz, Ar *H*), 8.12 (4H, d, J = 8.6 Hz, Ar *H*), 3.93 (6H, s, CH_3), identical to that described above.

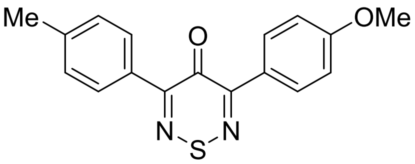

3-(4-Methoxyphenyl)-5-(p-tolyl)-4H-1,2,6-thiadiazin-4-one (4ai). Similar treatment (typical procedure A) of 3,5-dichloro-4*H*-1,2,6-thiadiazin-4-one (**6a**) (100 mg, 0.546 mmol) with *p*-tolyl-boronic acid (74.2 mg, 0.546 mmol) for 1 h, followed by (4-methoxyphenyl)boronic acid (91.3 mg, 0.601 mmol) for 1 h, gave after chromatography (*n*-hexane/DCM, 60:40) 3,5-di-*p*-tolyl-4*H*-1,2,6-thiadiazin-4-one (**4d**) (21.6 mg, 13%) as yellow needles, mp 201-203 °C (from *c*-hexane) [lit. mp 202-204 °C],[46] R_f 0.35 (*n*-hexane/DCM, 70:30); ^{1}H NMR ($CDCl_3$, 500 MHz) δ 8.11 (2H, d, *J* 8.0, Ar *H*), 7.29 (2H, d, *J* 8.5, Ar *H*), 2.44 (6H, s, CH_3), identical to an authentic sample.[46] Further elution (*n*-hexane/DCM, 60:40) gave the *title compound* **4ai** (92.3 mg, 55%) as yellow needles, mp 144-145 °C (from *c*-hexane/EtOH); R_f 0.30 (*n*-hexane/DCM, 60:40); (found: C, 65.90; H, 4.65; N, 9.17. $C_{17}H_{14}N_2O_2S$ requires C, 65.79; H, 4.55; N, 9.03%); λ_{max}(DCM)/nm 248 (log ε 4.16), 374 (4.40); ν_{max}/cm^{-1} 1628m, 1601s, 1571w, 1506m, 1460m, 1441w, 1410m, 1333m, 1304m, 1258s, 1177s, 1140m, 1117w, 1036m, 837s, 785m, 741m; ^{1}H NMR ($CDCl_3$, 500 MHz) δ 8.24 (d, 2H, J = 9.0 Hz, Ar *H*), 8.07 (d, 2H, J = 8.3 Hz, Ar *H*), 7.27 (d, 2H, J = 9.2 Hz, Ar *H*), 6.97 (d, 2H, J = 9.0 Hz, Ar *H*), 3.88 (s, 3H, CH_3), 2.42 (s, 3H, CH_3); ^{13}C{^{1}H} NMR ($CDCl_3$, 125 MHz) δ_C 165.6 (*C*q), 161.9 (*C*q), 160.2 (*C*q), 159.7 (*C*q), 141.4 (*C*q), 132.1 (*C*q), 130.9 (*C*H), 129.0 (*C*H), 128.9 (*C*H), 127.4 (*C*q), 113.6 (*C*H), 55.4 (O*C*H_3), 21.5 (*C*H_3); *m/z* (MALDI-TOF) calcd. for $C_{17}H_{15}N_2O_2S$ [M+H]$^+$: 311, Found: 311 (MH$^+$, 95%), 310 (M$^+$, 100), 290 (99), 279 (96), 214 (94), 165 (78), 135 (93), 119 (95). A final elution (*n*-hexane/DCM, 50:50) gave 3,5-bis(4-methoxyphenyl)-4*H*-1,2,6-thiadiazin-4-one (**4g**) (15.3 mg, 9%) as yellow needles, mp 169-171 °C (from *c*-hexane) [lit. mp 169-171 °C],[46] R_f 0.55 (*n*-hexane/DCM, 50:50); ^{1}H NMR ($CDCl_3$, 500 MHz) δ 8.22 (4H, d, J = 9.0 Hz, Ar *H*), 6.97 (4H, d, J = 8.5 Hz, Ar *H*), 3.87 (6H, s, OCH_3), identical to an authentic sample.[46]

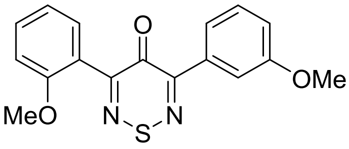

3-(2-Methoxyphenyl)-5-(3-methoxyphenyl)-4H-1,2,6-thiadiazin-4-one (4aj). Similar treatment (typical procedure B) of 3-chloro-5-(2-methoxyphenyl)-4*H*-1,2,6-thiadiazin-4-one (**21**) (50.9 mg, 0.200 mol) with (3-methoxyphenyl)-boronic acid (33.4 mg, 0.220 mol) for 30 min gave after chromatography (*n*-hexane/DCM, 50:50) the *title compound* **4aj** (55.6 mg, 85%) as yellow needles, mp 91-92

° C (from *c*-hexane); R_f 0.33 (*n*-hexane/DCM, 50:50); (found: C, 62.26; H, 4.10; N, 8.69. $C_{17}H_{14}N_2O_3S$ requires C, 62.56; H, 4.32; N, 8.58%); λ_{max}(DCM)/nm 239 (log ε 4.06), 342 (4.11); v_{max}/cm^{-1} 2940w and 2837w (alkyl C-H), 1643m, 1599m, 1574m, 1491m, 1464m, 1427m, 1341m, 1292m, 1287m, 1269s, 1250m, 1227m, 1179w, 1159w, 1134w, 1047s, 1024m, 1011m, 881m, 758s, 723m; ^{1}H NMR (Acetone-d_6, 500 MHz) δ 7.74 (ddd, 1H, J = 7.7, 1.5, 1.0 Hz, Ar *H*), 7.72 (dd, 1H, J = 2.6, 1.6 Hz, Ar *H*), 7.51-7.46 (m, 2H, Ar *H*), 7.42 (dd, 1H, J = 8.1, 8.1 Hz, Ar *H*), 7.14 (d, 1H, J = 8.3 Hz, Ar *H*), 7.12-7.07 (m, 2H, Ar *H*), 3.86 (s, 3H, C*H*$_3$), 3.85 (s, 3H, C*H*$_3$); ^{13}C{^{1}H} NMR (Acetone-d_6, 125 MHz) δ 165.6 (*C*q), 164.7 (*C*q), 160.4 (*C*q), 159.5 (*C*q), 157.9 (*C*q), 136.8 (*C*q), 132.4 (*C*H), 130.9 (*C*H), 130.2 (*C*H), 126.5 (*C*q), 121.8 (*C*H), 121.5 (*C*H), 117.7 (*C*H), 114.6 (*C*H), 112.4 (*C*H), 56.3 (*C*H$_3$), 55.7 (*C*H$_3$); *m/z* (MALDI-TOF) calcd. for $C_{17}H_{15}N_2O_3S$ [M+H]$^+$: 327, Found: 327 (MH$^+$, 54%), 285 (100), 151 (81), 135 (47).

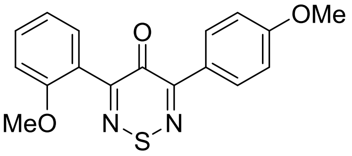

3-(2-Methoxyphenyl)-5-(4-methoxyphenyl)-4*H*-1,2,6-thiadiazin-4-one (4ak): (Typical procedure B, two-pot). To an intimate mixture of 3-chloro-5-(2-methoxyphenyl)-4*H*-1,2,6-thiadiazin-4-one (**21**) (50.9 mg, 0.200 mol), (4-methoxyphenyl)boronic acid (33.4 mg, 0.220 mol), Pd(Ph$_3$P)$_4$ (5.8 mg, 2.5 mol %), and Na_2CO_3 (21.2 mg, 0.200 mmol) was added dioxane (0.5 mL) and H_2O (0.3 mL) and the mixture heated to *ca*. 100 ° C until complete consumption of the starting thiadiazine (TLC, 1 h). The mixture was allowed to cool to *ca*. 20 ° C, adsorbed onto silica and chromatographed (*n*-hexane/DCM, 50:50) to give the *title compound* **4ak** (64.6 mg, 99%) as yellow plates, mp 134-135 ° C (from EtOH); R_f 0.26 (*n*-hexane/DCM, 50:50); (found: C, 62.78; H, 4.25; N, 8.31. $C_{17}H_{14}N_2O_3S$ requires C, 62.56; H, 4.32; N, 8.58%); λ_{max}(DCM)/nm 246 (log ε 4.11), 272 inf (3.96), 365 (4.33); v_{max}/cm^{-1} 3011w (aryl C-H), 2928w, 2930w and 2831w (alkyl C-H), 1616m, 1599s, 1572w, 1508m, 1497m, 1466m, 1456m, 1431m, 1414m, 1342m, 1314m, 1288m, 1258s, 1180s, 1165w, 1150m, 1113m, 1051m, 1030m, 1011w, 997m, 878w, 864w, 851m, 814m, 758s, 735m; ^{1}H NMR (Acetone-d_6, 500 MHz) δ 8.22 (d, 2H, J = 9.1 Hz, Ar *H*), 7.48 (ddd, 1H, J = 7.5, 7.5, 1.8 Hz, Ar *H*), 7.46 (dd, 1H, J = 7.3, 1.8 Hz, Ar *H*), 7.13 (d, 1H, J = 8.2 Hz, Ar *H*), 7.08 (ddd, 1H, J = 7.5, 7.5, 0.9 Hz, Ar *H*), 7.05 (d, 2H, J = 9.1 Hz, Ar *H*), 3.89 (s, 3H, C*H*$_3$), 3.83 (s, 3H, C*H*$_3$); ^{13}C{^{1}H} NMR (Acetone-d_6, 125 MHz) δ 165.8 (*C*q), 164.1 (*C*q), 163.1 (*C*q), 159.1 (*C*q), 157.9 (*C*q), 132.3 (*C*H), 131.4 (*C*H), 130.9 (*C*H), 128.1 (*C*q), 126.7 (*C*q), 121.5 (*C*H), 114.5 (*C*H), 112.3 (*C*H), 56.2 (*C*H$_3$), 55.0 (*C*H$_3$); *m/z* (MALDI-TOF) calcd. for $C_{17}H_{15}N_2O_3S$ [M+H]$^+$: 327, Found: 327 (MH$^+$, 54%), 326 (M$^+$, 92), 304 (86), 295 (68), 256 (100), 228 (95), 192 (63), 135 (89).

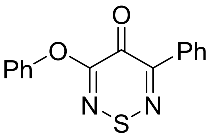

3-Phenoxy-5-phenyl-4*H*-1,2,6-thiadiazin-4-one (4ac). Similar treatment (typical procedure B) of 3-chloro-5-phenoxy-4*H*-1,2,6-thiadiazin-4-one (120 mg, 0.500 mol) with phenylboronic acid (67.0 mg, 0.550 mmol) for 2 h gave after chromatography (*n*-hexane/DCM, 60:40) the *title compound* **4ac** (78 mg, 55%) as yellow plates, mp 152-153 ° C (from *c*-hexane); R_f 0.17 (*n*-hexane/DCM, 60:40); (found: C, 63.75; H, 3.50; N, 9.95. $C_{15}H_{10}N_2O_2S$ requires C, 63.82; H, 3.57; N, 9.92%); λ_{max}(DCM)/nm 243 inf (log ε 3.77), 316 (4.16), 354 (4.17); v_{max}/cm^{-1} 3092w and 3065w (aryl C-H), 1632m, 1589w, 1524m, 1487m, 1454w, 1441m, 1342s, 1314m, 1279s, 1194s, 1180m, 1165m, 1121m, 1078w, 1022m, 1003w, 974w, 876m, 754s, 733s; ^{1}H NMR ($CDCl_3$, 500 MHz) δ 8.20-8.18 (m, 2H, Ar *H*),

7.50-7.45 (m, 5H, Ar *H*), 7.33 (ddt, 1H, J = 7.4, 7.4, 1.0 Hz, Ar *H*), 7.22-7.20 (m, 2H, Ar *H*); $^{13}C\{^1H\}$ NMR ($CDCl_3$, 125 MHz) δ 161.1 (*C*q), 158.9 (*C*q), 157.9 (*C*q), 151.5 (*C*q), 134.2 (*C*q), 130.9 (*C*H), 129.8 (*C*H), 128.8 (*C*H), 128.3 (*C*H), 126.4 (*C*H), 121.8 (*C*H); *m/z* (MALDI-TOF) calcd. for $C_{15}H_{11}N_2O_2S$ $[M+H]^+$: 283, Found: 283 (MH^+, 64%), 281 (M^+, 81), 279 (100), 272 (60), 256 (35), 180 (39).

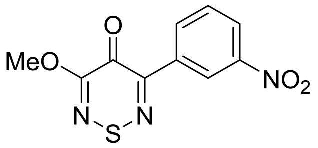

3-Methoxy-5-(3-nitrophenyl)-4*H*-1,2,6-thiadiazin-4-one (4ad). Similar treatment (typical procedure B) of 3-chloro-5-methoxy-4*H*-1,2,6-thiadiazin-4-one (89.3 mg, 0.500 mol) with (3-nitrophenyl)boronic acid (91.8 mg, 0.550 mol) for 15 min gave after chromatography (*n*-hexane/DCM, 40:60) the *title compound* **4ad** (72.5 mg, 55%) as yellow needles, mp 175-176 ° C (from DCE/*c*-hexane); R_f 0.29 (*n*-hexane/DCM, 40:60); (found: C, 45.00; H, 2.34; N, 15.76. $C_{10}H_7N_3O_4S$ requires C, 45.28; H, 2.66; N, 15.84%); λ_{max}(DCM)/nm 281 (log ε 3.99), 314 (4.00), 356 (4.12); ν_{max}/cm^{-1} 3123w, 3098w, 2945w (C-H), 1632s, 1535s, 1518s, 1477w, 1449w, 1427w, 1342s, 1281m, 1192m, 1179w, 1146m, 1092m, 1001m, 955m, 907m, 866m, 812m, 745m, 735m; ^1H NMR ($CDCl_3$, 500 MHz) δ 9.03 (dd, 1H, J = 2.0, 2.0 Hz, Ar *H*), 8.59 (ddd, 1H, J = 7.9, 1.1, 1.1 Hz, Ar *H*), 8.31 (ddd, 1H, J = 8.2, 2.2, 1.4 Hz, Ar *H*), 7.65 (dd, 1H, J = 8.0, 8.0 Hz, Ar *H*), 4.04 (s, 3H, CH_3); $^{13}C\{^1H\}$ NMR ($CDCl_3$, 125 MHz) δ 161.0 (*C*q), 159.2 (*C*q), 154.6 (*C*q), 148.2 (*C*q), 135.6 (*C*q), 134.4 (*C*H), 129.3 (*C*H), 124.9 (*C*H), 123.7 (*C*H), 55.1 (CH_3); *m/z* (MALDI-TOF) calcd. for $C_{10}H_8N_3O_4S$ $[M+H]^+$: 266, Found: 266 (MH^+, 100%).

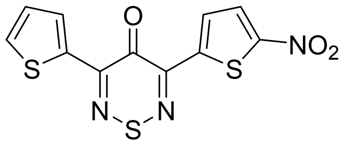

3-(5-Nitrothien-2-yl)-5-(thien-2-yl)-4*H*-1,2,6-thiadiazin-4-one (4ae). To a beaker cooled at -15 ° C using a salt/ice water bath, 15 mL of Ac_2O was added and allowed to cool. Then, concd HNO_3 (3.5 mL) was slowly added and mixed thoroughly. The resulting solution was then added to a 50 mL round bottom flask containing 3,5-di(thien-2-yl)-4*H*-1,2,6-thiadiazin-4-one (**4m**) (100 mg, 0.359 mmol) in DCM (10 mL) and the solution stirred until complete consumption of **4m** as monitored by TLC (*n*-hexane/DCM, 50:50). The solution was then placed in a separation funnel, and the organic phase was collected and washed with brine. The organic phase was then dried with Na_2SO_4, and the solvent was evaporated under vacuum. The resulting residue was then separated using column chromatography using an eluent mixture of *n*-hexane/DCM (50:50) to afford the *title compound* **4ae** (97.4 mg, 84%) as orange needles, mp (hot-stage) 206-207 ° C ($CHCl_3$); R_f 0.43 (*n*-hexane/DCM, 50:50); λ_{max}(DCM)/nm 287 (log ε 3.88), 436.5 (3.91); ν_{max}/cm^{-1} 3102m, 2922m, 1598s (C=O), 1522w, 1492s, 1408m, 1391m, 1325s, 1260m, 1221w, 1200w, 1150w, 1125w, 1056m, 1037m, 1000m, 873m, 844w, 820w, 844w, 820s, 789m, 731m, 713s; ^1H NMR ($CDCl_3$, 400 MHz) δ 8.37 (dd, 1H, J = 4.0, 1.2 Hz, Ar *H*), 8.04 (d, 1H, J = 4.4 Hz, Ar *H*), 7.96 (d, 1H, J = 4.8 Hz, Ar *H*), 7.74 (dd, 1H, J = 5.2, 1.2 Hz, Ar *H*), 7.24 (dd, 1H, J = 5.2, 4.0 Hz, Ar *H*); $^{13}C\{^1H\}$ NMR ($CDCl_3$, 100 MHz) δ 161.2, 155.1, 152.0, 139.8, 136.1, 135.0, 133.7, 129.4, 128.4, 127.7, one C resonance missing; *m/z* (ESI-) calcd. for $C_{11}H_5ClN_3O_3S_3$ $[M+Cl]^-$ 357.9187, Found: 357.9173.

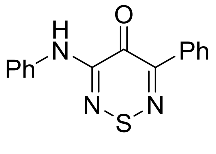

3-Phenyl-5-(phenylamino)-4*H*-1,2,6-thiadiazin-4-one (4af). Similar treatment (general procedure B) of 3-chloro-5-(phenylamino)-4*H*-1,2,6-thiadiazin-4-one (120 mg, 0.500

mol) with phenylboronic acid (67.0 mg, 0.550 mmol) for 2 h gave after chromatography (*n*-hexane/DCM, 70:30) the *title compound* **4af** (128.3 mg, 91%) as orange needles, mp 158-159 °C (from *c*-hexane); R_f 0.30 (*n*-hexane/DCM, 70:30); (found: C, 64.28; H, 3.64; N, 14.89. $C_{15}H_{11}N_3OS$ requires C, 64.04; H, 3.94; N, 14.94%); λ_{max}(DCM)/nm 253 inf (log ε 3.89), 346 (4.34), 411 (3.87); ν_{max}/cm^{-1} 3300w (N-H), 1605m, 1584s, 1551s, 1510w, 1497m, 1447m, 1342m, 1310w, 1229m, 1186w, 1030w, 893m, 872m, 750s; ^{1}H NMR ($CDCl_3$, 500 MHz) δ 9.02 (1H, br. s, N*H*), 8.18 (2H, dd, J = 8.2, 1.9 Hz, Ar *H*), 7.69 (2H, d, J = 7.8 Hz, Ar *H*), 7.49-7.45 (m, 3H, Ar *H*), 7.40 (dd, 2H, J = 7.8, 7.8 Hz, Ar *H*), 7.17 (dd, 1H, J = 7.4, 7.4 Hz, Ar *H*); ^{13}C{^{1}H} NMR ($CDCl_3$, 125 MHz) δ 159.1 (*C*q), 152.2 (*C*q), 151.9 (*C*q), 137.4 (*C*q), 135.2 (*C*q), 130.0 (*C*H), 129.2 (*C*H), 128.4 (*C*H), 128.2 (*C*H), 124.5 (*C*H), 119.7 (*C*H); *m/z* (APCI+) calcd. for $C_{15}H_{12}N_3OS$ [M+H]$^+$: 282, Found: 282 (MH$^+$, 100%), 153 (16), 130 (45), 124 (22).

S4.2 Preparation of 1,2,5-thiadiazole *S*-oxides from 4*H*-1,2,6-thiadiazines

It is noted that no R_f values are reported for the 1,2,5-thiadiazole S-oxide products as those were prone to decompose on silica. Moreover, it was observed that some of the derivatives, exhibited substantial fragmentation upon MS analysis, making the identification of their molecular peaks challenging. Since this scaffold has not been reported previously, a known fragmentation pattern is not available. In general, the isolated 1,2,5-thiadiazole *S*-oxides reported herein were obtained without subjecting them to further purification unless otherwise stated. In cases where the resulting ^{1}H NMR spectrum contained impurities the compound was triturated with 2 x 5 mL Et_2O and left to dry under vacuum.

S4.2.1 Symmetric derivatives

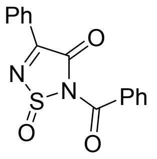

2-Benzoyl-4-phenyl-1,2,5-thiadiazol-3(2*H*)-one 1-oxide (5a).

Method A (batch): In a screw capped 5 mL glass vial, a stirred solution of 3,5-diphenyl-4*H*-1,2,6-thiadiazin-4-one (**4a**) (10.0 mg, 0.0375 mmol) in $CDCl_3$ (1 mL) at *ca.* 20 °C was irradiated with 420 nm light until it was consumed (monitored by TLC, $CHCl_3$/*n*-hexane, 50:50) over a period of 15 min. The resulting solution was then evaporated giving the *title compound* **5a** (11.2 mg, 100%) as colorless needles, mp (hot-stage) 171-172 °C ($CHCl_3$); (found: C, 60.04; H, 3.38; N, 9.45; S, 10.77. $C_{15}H_{10}N_2O_3S$ requires C, 60.39; H, 3.38; N, 9.39; S, 10.75%); λ_{max}(DCM)/nm 244.5 (log ε 4.32), 296 (4.26); ν_{max}/cm^{-1} 1733s (C=O), 1699s (C=O), 1600w, 1584m, 1563m, 1491w, 1447m, 1322w, 1309w, 1254m, 1208m, 1186s, 1177w, 1150s, 1066m (S=O), 1041m, 1019m, 1000w, 977w, 938w, 926w, 900w, 866w, 837w, 803m, 784w, 747m, 715m; ^{1}H NMR ($CDCl_3$, 300 MHz) δ 8.47-8.51 (m, 2H, Ar *H*), 7.81-7.85 (m, 2H, Ar *H*), 7.66-7.74 (m, 2H, Ar *H*), 7.50-7.57 (m, 4H, Ar *H*); ^{13}C{^{1}H} NMR ($CDCl_3$, 100 MHz) δ 167.9, 166.4, 157.8, 135.5, 134.5, 131.3, 131.1, 130.2, 129.3, 128.7, 128.4; *m/z* (ESI+) calcd. for $C_{15}H_{11}N_2O_3S$ [M+H]$^+$ 299.0490, Found: 299.0468.

Method B (flow): The contents of a 25 mL round bottom flask containing a solution of 3,5-diphenyl-4*H*-1,2,6-thiadiazin-4-one (**4a**) (10.0 mg, 0.0375 mmol) in $CDCl_3$ (5 mL) were pumped through a Vapourtec UV-150 photochemical reactor, equipped with a 420 nm LED lamp at 3.30 mL·min^{-1} (1 min residence time). After exiting the photoreactor, the solution was then passed through a back pressure regulator which kept the pressure

at 3 bar before exiting the flow machine and collected in a 25 mL round bottom flask. The resulting solution was then evaporated giving the *title compound* **5a** (11.2 mg, 100%) as colorless needles, mp (hot-stage) 171-172 °C ($CHCl_3$); identical to that described above.

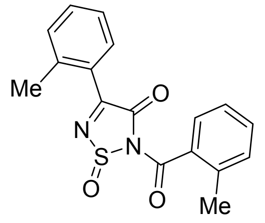

2-(2-Methylbenzoyl)-4-(*o*-tolyl)-1,2,5-thiadiazol-3(2*H*)-one 1-oxide (5b). Similar treatment (**Method A**) of 3,5-di-*o*-tolyl-4*H*-1,2,6-thiadiazin-4-one (**4b**) (11.0 mg, 0.0375 mmol) under 420 nm light for 30 min gave the *title compound* **5b** (12.2 mg, 100%) as colorless needles, mp (hot-stage) 175-176 °C ($CHCl_3$) decomp.; λ_{max}(DCM)/nm 247 (log ε 3.94), 308 (3.92); ν_{max}/cm^{-1} 2927w (alkyl C-H), 1735m (C=O), 1692m (C=O), 1586m, 1573m, 1487w, 1457m, 1381w, 1300w, 1242s, 1213s, 1197m, 1147s, 1056m, 1044m, 1021m, 905w, 868w, 824w, 777m, 762m, 747m, 734s, 700m; ^{1}H NMR ($CDCl_3$, 400 MHz) δ 8.00 (dd, 1H, J = 8.4, 1.6 Hz, Ar *H*), 7.44-7.52 (m, 3H, Ar *H*), 7.29-7.38 (m, 4H, Ar *H*), 2.63 (s, 3H, C*H*$_3$), 2.47 (s, 3H, C*H*$_3$); ^{13}C{^{1}H} NMR ($CDCl_3$, 100 MHz) δ 168.4, 168.3, 157.6, 141.3, 137.7, 133.7, 132.44, 132.38, 132.3, 131.9, 131.4, 128.6, 126.9, 126.2, 126.0, 22.0, 19.8; *m/z* (ESI+) calcd. for $C_{17}H_{15}N_2O_3S$ $[M+H]^+$ 327.0803, Found: 327.0783.

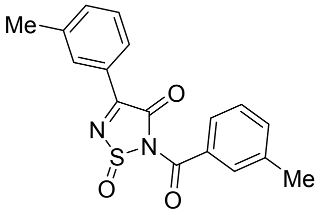

2-(3-Methylbenzoyl)-4-(*m*-tolyl)-1,2,5-thiadiazol-3(2*H*)-one 1-oxide (5c). Similar treatment (**Method B**) of 3,5-di-*m*-tolyl-4*H*-1,2,6-thiadiazin-4-one (**4c**) (11.0 mg, 0.0375 mmol) under 420 nm light for 2 min gave the *title compound* **5c** (11.8 mg, 97%) as colorless needles, mp (hot-stage) 143-144 °C ($CHCl_3$); λ_{max}(DCM)/nm 253 (log ε 4.91), 314 (4.86); ν_{max}/cm^{-1} 2923w (alkyl C-H), 1728s (C=O), 1695m (C=O), 1606w, 1581m, 1554m, 1479w, 1456w, 1425w, 1385w, 1276m, 1225m, 1193m, 1161s, 1085m, 1054m, 1000w, 939m, 932w, 890m, 826m, 797m, 760s, 739s, 716m; ^{1}H NMR ($CDCl_3$, 400 MHz) δ 8.29 (dd, 2H, J = 5.6, 0.4 Hz, Ar *H*), 7.59-7.62 (m, 2H, Ar *H*), 7.49 (dd, 2H, J = 8.0, 8.0 Hz, Ar *H*), 7.38-7.43 (m, 2H, Ar *H*), 2.43 (s, 3H, C*H*$_3$), 2.42 (s, 3H, C*H*$_3$); ^{13}C{^{1}H} NMR ($CDCl_3$, 100 MHz) δ 168.1, 166.4, 157.9, 139.2, 138.8, 136.4, 135.3, 131.4, 130.5, 129.2, 128.6, 128.41, 128.37, 127.3, 21.5, 21.4, one C resonance missing; *m/z* (ESI+) calcd. for $C_{17}H_{15}N_2O_3S$ $[M+H]^+$ 327.0803, found: 327.0809.

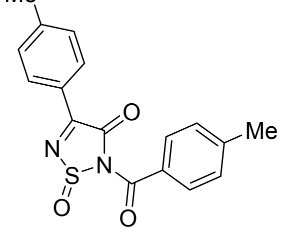

2-(4-Methylbenzoyl)-4-(*p*-tolyl)-1,2,5-thiadiazol-3(2*H*)-one 1-oxide (5d). Similar treatment (**Method A**) of 3,5-di-*p*-tolyl-4*H*-1,2,6-thiadiazin-4-one (**4d**) (11.0 mg, 0.0375 mmol) under 420 nm light for 15 min gave the *title compound* **5d** (12.2 mg, 100%) as colorless needles, mp (hot-stage) 169-170 °C ($CHCl_3$) decomp.; λ_{max}(DCM)/nm 250 (log ε 4.17), 326 (4.13); ν_{max}/cm^{-1} 2920w (alkyl C-H), 1728m (C=O), 1700m (C=O), 1606m, 1585m, 1551m, 1509w, 1447w, 1401w, 1364w, 1309w, 1254m, 1207m, 1171m, 1149s, 1063m, 1033w, 1017s, 968w, 906w, 833m, 826m, 796w, 777w, 758m, 740s; ^{1}H NMR ($CDCl_3$, 400 MHz) δ 8.40 (dd, 2H, J = 6.8, 1.6 Hz, Ar *H*), 7.74 (dd, 2H, J = 6.8, 1.6 Hz, Ar *H*), 7.31-7.34 (m, 4H, Ar *H*), 2.46 (s, 6H, C*H*$_3$); ^{13}C{^{1}H} NMR ($CDCl_3$, 100 MHz) δ 167.7, 166.0, 158.1, 147.2, 145.8, 131.2, 130.4, 130.1, 129.5, 128.6, 125.8, 22.2, 22.1; *m/z* (ESI+) calcd. for $C_{17}H_{15}N_2O_3S$ $[M+H]^+$ 327.0803, Found: 327.0790.

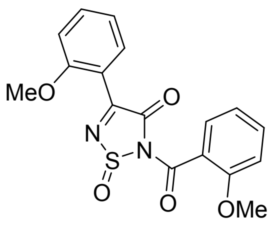

2-(2-Methoxybenzoyl)-4-(2-methoxyphenyl)-1,2,5-thiadiazol-3(2*H*)-one 1-oxide (5e). Similar treatment (**Method A**) of 3,5-bis(2-methoxyphenyl)-4*H*-1,2,6-thiadiazin-4-one (**4e**) (12.2 mg, 0.0375 mmol) under 420 nm light for 4 h gave the *title compound* **5e** (13.4 mg, 100%) as yellow needles, mp (hot-stage) 139-140 ° C ($CHCl_3$) decomp.; λ_{max}(DCM)/nm 262 (log ε 4.15), 297 (3.14), 370 inf (3.60); ν_{max}/cm^{-1} 2931w and 2836w (alkyl C-H), 1762m (C=O), 1664m (C=O), 1664m, 1598s, 1490m, 1455m, 1432m, 1297m, 1276m, 1243s, 1196w, 1162w, 1143s, 1124m, 1073w, 1052w, 1041w, 1019s, 936w, 907m, 851w, 816w, 747s, 708m; ^{1}H NMR ($CDCl_3$, 400 MHz) δ 7.74 (dd, 1H, J = 8.0, 1.6 Hz, Ar *H*), 7.50-7.57 (m, 3H, Ar *H*), 7.03-7.08 (m, 3H, Ar *H*), 6.96 (d, 1H, J = 8.4 Hz, Ar *H*), 3.88 (s, 3H, C*H*$_3$), 3.83 (s, 3H, C*H*$_3$); ^{13}C{^{1}H} NMR ($CDCl_3$, 100 MHz) δ 169.1, 166.5, 159.9, 157.7, 156.5, 135.2, 134.2, 132.2, 130.3, 122.3, 121.0, 120.9, 117.9, 112.3, 111.4, 56.3, 56.0; *m/z* (ESI+) calcd. for $C_{17}H_{15}N_2O_5S$ [M+H]$^+$ 359.0702, Found: 359.0711.

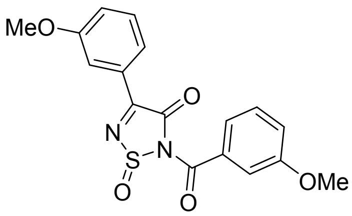

2-(3-Methoxybenzoyl)-4-(3-methoxyphenyl)-1,2,5-thiadiazol-3(2*H*)-one 1-oxide (5f). Similar treatment (**Method A**) of 3,5-bis(3-methoxyphenyl)-4*H*-1,2,6-thiadiazin-4-one (**4f**) (12.2 mg, 0.0375 mmol) under 420 nm light for 25 min gave the *title compound* **5f** (13.4 mg, 100%) as yellow needles, mp (hot-stage) 119-120 ° C ($CHCl_3$) decomp.; λ_{max}(DCM)/nm 260 (log ε 4.07), 310 (4.00), 379 inf (3.44); ν_{max}/cm^{-1} 3080w (aryl C-H) and 3003w (C-H), 2940w and 2830w (alkyl C-H), 1732s (C=O), 1688m (C=O), 1597m, 1557m, 1487m, 1425m, 1335w, 1291w, 1275m, 1247m, 1227m, 1197w, 1152s, 1083w, 1056w, 1029s, 994m, 943m, 890w, 857m, 822m, 790m, 756m, 740m; ^{1}H NMR ($CDCl_3$, 400 MHz) δ 8.14 (dt, 1H, J = 7.6, 1.2 Hz, Ar *H*), 7.97 (dd, 1H, J = 2.4, 1.6 Hz, Ar *H*), 7.38-7.46 (m, 3H, Ar *H*), 7.33 (dd, 1H, J = 2.4, 1.2 Hz, Ar *H*), 7.22-7.26 (m, 2H, Ar *H*), 3.88 (s, 3H, C*H*$_3$), 3.87 (s, 3H, C*H*$_3$); ^{13}C{^{1}H} NMR ($CDCl_3$, 100 MHz) δ 167.7, 166.1, 160.1, 159.8, 157.8, 132.6, 130.3, 129.8, 129.5, 124.0, 122.7, 122.5, 120.9, 114.6, 55.74, 55.72, one C resonance missing; *m/z* (ESI+) calcd. for $C_{17}H_{15}N_2O_5S$ [M+H]$^+$ 359.0702, Found: 359.0714.

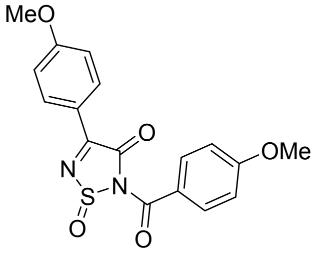

2-(4-Methoxybenzoyl)-4-(4-methoxyphenyl)-1,2,5-thiadiazol-3(2*H*)-one 1-oxide (5g). Similar treatment (**Method A**) of 3,5-bis(4-methoxyphenyl)-4*H*-1,2,6-thiadiazin-4-one (**4g**) (12.2 mg, 0.0375 mmol) under 420 nm light for 15 min gave the *title compound* **5g** (13.4 mg, 100%) as yellow needles, mp (hot-stage) 192-193 ° C ($CHCl_3$) decomp.; λ_{max}(DCM)/nm 257 (log ε 3.99), 281 (3.97), 359 (4.05); ν_{max}/cm^{-1} 2941w and 2846w (alkyl C-H), 1728m (C=O), 1692m (C=O), 1601s, 1577m, 1548m, 1511m, 1459w, 1445w, 1424w, 1317m, 1258s, 1182w, 1166m, 1152s, 1064m, 1018m, 907m 841m, 817w, 784w, 762m, 753m, 727w, 717w, 702m; ^{1}H NMR ($CDCl_3$, 400 MHz) δ 8.53-8.57 (m, 2H, Ar *H*), 7.83-7.87 (m, 2H, Ar *H*), 6.98-7.03 (m, 4H, Ar *H*), 3.92 (s, 3H, C*H*$_3$), 3.91 (s, 3H, C*H*$_3$); ^{13}C{^{1}H} NMR ($CDCl_3$, 100 MHz) δ 166.9, 165.8, 164.9, 164.8, 158.6, 133.8, 133.1, 123.6, 121.1, 114.9, 114.1, 55.9, 55.8; *m/z* (ESI+) calcd. for $C_{17}H_{15}N_2O_5S$ [M+H]$^+$ 359.0702, Found: 359.0683.

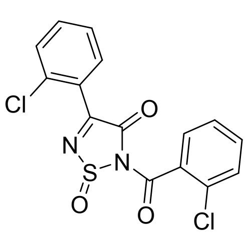

2-(2-Chlorobenzoyl)-4-(2-chlorophenyl)-1,2,5-thiadiazol-3(2*H*)-one 1-oxide (5h). Similar treatment (**Method A**) of 3,5-bis(2-chlorophenyl)-4*H*-1,2,6-thiadiazin-4-one (**4h**) (12.6 mg, 0.0375 mmol) under 420 nm light for 2 h gave the *title compound* **5h** (13.8 mg, 100%) as colorless plates, mp (hot-stage) 173-175 ° C ($CHCl_3$) decomp.; λ_{max}(DCM)/nm 241 (log ε 3.90), 296 (3.76); ν_{max}/cm^{-1} 3098w (C-H), 1743s (C=O), 1703s (C=O), 1589m, 1566w, 1473w, 1436m, 1291w, 1249s, 1210s, 1151s, 1094m, 1072w, 1031m, 1020m, 958w, 909w, 875w, 803m, 777m, 744s, 715w; ^{1}H NMR ($CDCl_3$, 400 MHz) δ 7.69-7.71 (m, 1H, Ar *H*), 7.53-7.55 (m, 2H, Ar *H*), 7.48-7.51 (m, 3H, Ar *H*) 7.39-7.43 (m, 2H, Ar *H*); ^{13}C{^{1}H} NMR ($CDCl_3$, 100 MHz) δ 169.0, 165.6, 156.1, 134.5, 134.1, 133.1, 132.29, 132.25, 131.5, 131.2, 130.2, 129.4, 127.4, 127.13, 127.08; *m/z* (ESI+) calcd. for $C_{15}H_9Cl_2N_2O_3S$ $[M+H]^+$ 366.9711, Found: 366.9743.

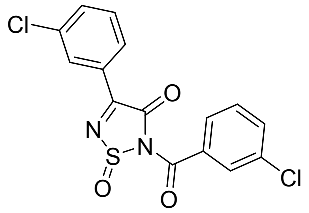

2-(3-Chlorobenzoyl)-4-(3-chlorophenyl)-1,2,5-thiadiazol-3(2*H*)-one 1-oxide (5i). Similar treatment (**Method A**) of 3,5-bis(3-chlorophenyl)-4*H*-1,2,6-thiadiazin-4-one (**4i**) (12.6 mg, 0.0375 mmol) under 420 nm light for 25 min gave the *title compound* **5i** (13.8 mg, 100%) as colorless needles, mp (hot-stage) 167-169 ° C ($CHCl_3$) decomp.; λ_{max}(DCM)/nm 244 (log ε 3.57), 300 (3.49); ν_{max}/cm^{-1} 3069w (aryl C-H), 1736m (C=O), 1692s (C=O), 1576m, 1551s, 1472w, 1423m, 1408m, 1284m, 1252s, 1197m, 1153s, 1089w, 1072m, 1032w, 998w, 928m, 914w, 889m, 830w, 800m, 792m, 769w, 740s, 712m; ^{1}H NMR ($CDCl_3$, 400 MHz) δ 8.47 (dd, 1H, J = 2.0, 2.0 Hz, Ar *H*), 8.38 (dt, 1H, J = 8.4, 1.2 Hz, Ar *H*), 7.78 (dd, 1H, J = 2.0, 2.0 Hz, Ar *H*) 7.65-7.70 (m, 3H, Ar *H*), 7.49 (dd, 2H, J = 15.2, 7.6 Hz, Ar *H*); ^{13}C{^{1}H} NMR ($CDCl_3$, 100 MHz) δ 166.7, 165.4, 157.3, 135.7, 135.6, 135.1, 134.5, 132.8, 130.8, 130.6, 130.1, 130.0, 129.8, 129.2, 128.1; *m/z* (ESI+) calcd. for $C_{15}H_9Cl_2N_2O_3SNa$ $[M+Na]^+$ 388.9530, Found: 388.9549.

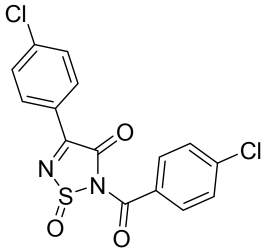

2-(4-Chlorobenzoyl)-4-(4-chlorophenyl)-1,2,5-thiadiazol-3(2*H*)-one 1-oxide (5j). Similar treatment (**Method A**) of 3,5-bis(4-chlorophenyl)-4*H*-1,2,6-thiadiazin-4-one (**4j**) (12.6 mg, 0.0375 mmol) under 420 nm light for 15 min gave the *title compound* **5j** (13.8 mg, 100%) as colorless needles, mp (hot-stage) 179-181 ° C ($CHCl_3$) decomp.; λ_{max}(DCM)/nm 250 (log ε 4.18), 325 (4.10); ν_{max}/cm^{-1} 3100w (aryl C-H), 1728m (C=O), 1673m (C=O), 1586s, 1488m, 1402m, 1304w, 1270m, 1210m, 1185w, 1151s, 1117w, 1090s, 1032w, 1011m, 958w, 901m, 840s, 790m, 763m, 743s, 715m; ^{1}H NMR ($CDCl_3$, 400 MHz) δ 8.45 (dd, 2H, J = 8.8, 2.4 Hz, Ar *H*), 7.77 (dd, 2H, J = 8.8, 2.4 Hz, Ar *H*), 7.50-7.55 (m, 4H, Ar *H*); ^{13}C{^{1}H} NMR ($CDCl_3$, 100 MHz) δ 166.9, 165.4, 157.6, 142.8, 141.4, 132.4, 131.6, 129.9, 129.5, 129.2, 126.7; *m/z* (ESI+) calcd. for $C_{15}H_9Cl_2N_2O_3SNa$ $[M+Na]^+$ 388.9530, Found: 388.9530.

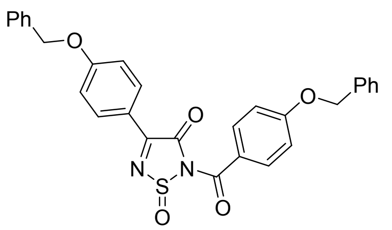

2-[4-(Benzyloxy)benzoyl]-4-[4-(benzyloxy)phenyl]-1,2,5-thiadiazol-3(2*H*)-one 1-oxide (5k). Similar treatment (**Method A**) of 3,5-bis[4-(benzyloxy)phenyl]-4*H*-1,2,6-thiadiazin-4-one (**4k**) (17.9 mg, 0.0375 mmol) under 420 nm light for 15 min gave the *title compound* **5k** (19.1 mg, 100%) as yellow plates, mp (hot-stage) 163-164 ° C ($CHCl_3$); λ_{max}(DCM)/nm 259 (log

ε 4.08), 283 (4.09), 361 (4.13); ν_{max}/cm^{-1} 3064w and 3037w (aryl C-H), 2873w (alkyl C-H), 1730m (C=O), 1690w (C=O), 1674m (C=O), 1602s, 1569m, 1542s, 1509s, 1466w, 1455m, 1423m, 1384m 1307m, 1274m, 1255s, 1210m, 1153s, 1123m, 1069m, 1033m, 988s, 917w, 905m, 835m, 812w, 776m, 750s, 742m; ^{1}H NMR (CD_2Cl_2, 400 MHz) δ 8.53 (dt, 2H, J = 9.2, 3.2 Hz, Ar *H*), 7.84 (dt, 2H, J = 9.2, 2.8 Hz, Ar *H*), 7.37-7.48 (m, 10H, Ar *H*), 7.08-7.13 (m, 4H, Ar *H*), 5.19 (d, 4H, J = 5.2 Hz, CH_2); ^{13}C{^{1}H} NMR (CD_2Cl_2, 100 MHz) δ 167.4, 165.44, 165.41, 164.5, 158.8, 136.6, 136.4, 134.1, 133.4, 129.3, 129.2, 129.0, 128.9, 128.2, 124.3, 121.8, 116.1, 115.3, 71.03, 70.97, one C resonance missing; *m/z* (ESI+) calcd. for $C_{29}H_{23}N_2O_5S$ $[M+H]^+$ 511.1322, Found: 511.1317.

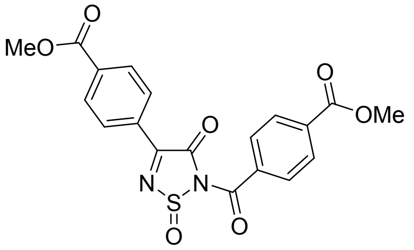

Methyl 4-{5-[4-(methoxycarbonyl)benzoyl]-1-oxido-4-oxo-4,5-dihydro-1,2,5-thiadiazol-3-yl}benzoate (5l). Similar treatment (**Method A**) of dimethyl 4,4'-(4-oxo-4*H*-1,2,6-thiadiazine-3,5-diyl)dibenzoate (**4l**) (14.2 mg, 0.0375 mmol) under 420 nm light for 15 min gave the *title compound* **5l** (15.5 mg, 100%) as off-yellow plates, mp (hot-stage) 182-183 °C ($CHCl_3$); λ_{max}(DCM)/nm 244 (log ε 4.35), 300 (4.24); ν_{max}/cm^{-1} 2964w (alkyl C-H), 1727s (C=O), 1698m (C=O), 1579m, 1555m, 1503w, 1433m, 1407m, 1394w, 1317w, 1266s, 1260s, 1206m, 1158s, 1107s, 1069m, 1031w, 1014m, 955m, 908m, 866m, 826m, 796w, 781m, 779m, 727s, 701m; ^{1}H NMR (CD_2Cl_2, 400 MHz) δ 8.50-8.53 (m, 2H, Ar *H*), 8.16-8.19 (m, 4H, Ar *H*), 7.86-7.88 (m, 2H, Ar *H*), 3.952 (s, 3H, CH_3), 3.946 (s, 3H, CH_3); ^{13}C{^{1}H} NMR (CD_2Cl_2, 100 MHz) δ 167.9, 166.6, 166.2, 157.8, 136.4, 135.6, 135.4, 132.2, 131.4, 130.5, 130.3, 130.1, 53.12, 53.08, one C resonance missing; *m/z* (ESI+) calcd. for $C_{19}H_{15}N_2O_7S$ $[M+H]^+$ 415.0594, Found: 415.0595.

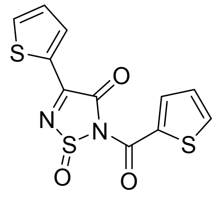

***4-(Thien-2-yl)-2-(thiophene-2-carbonyl)-1,2,5-thiadiazol-3(2*H*)-one 1-oxide* (5m).** Similar treatment (**Method A**) of 3,5-di(thien-2-yl)-4*H*-1,2,6-thiadiazin-4-one (**4m**) (10.4 mg, 0.0375 mmol) under 420 nm light for 15 min gave the *title compound* **5m** (11.6 mg, 100%) as beige-yellow needles, mp (hot-stage) 180-181 °C ($CHCl_3$) decomp.; λ_{max} (DCM)/nm 264 (log ε 4.03), 353.5 (4.03); ν_{max}/cm^{-1} 3105m and 3087m (aryl C-H), 1723s (C=O), 1648s (C=O), 1558s, 1515m, 1421m, 1406s, 1353m, 1262s, 1238s, 1160s, 1053s, 995m, 850m, 829w, 728s; ^{1}H NMR ($CDCl_3$, 300 MHz) δ 8.61 (dd, 1H, J = 4.2 Hz, 1.2, Ar *H*), 8.01 (dd, 1H, J = 3.9, 1.2 Hz, Ar *H*), 7.97 (dd, 1H, J = 5.1, 1.2 Hz, Ar *H*), 7.85 (dd, 1H, J = 4.8, 1.2 Hz, Ar *H*), 7.32 (dd, 1H, J = 5.1, 3.9 Hz, Ar *H*), 7.23 (dd, 1H, J = 5.1, 4.2 Hz, Ar *H*); ^{13}C{^{1}H} NMR ($CDCl_3$, 100 MHz) δ 160.9, 160.3, 157.2, 139.4, 138.6, 137.07, 137.06, 134.8, 131.1, 129.7, 128.5; *m/z* (ESI+) calcd. for $C_{11}H_7N_2O_3S_3$ $[M+H]^+$ 310.9619, Found: 310.9613.

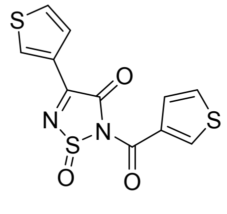

4-(Thien-3-yl)-2-(thiophene-3-carbonyl)-1,2,5-thiadiazol-3(2*H*)-one 1-oxide (5n). Similar treatment (**Method A**) of 3,5-di(thien-3-yl)-4*H*-1,2,6-thiadiazin-4-one (**4n**) (10.4 mg, 0.0375 mmol) under 420 nm light for 35 min gave the *title compound* **5n** (11.6 mg, 100%) as beige needles, mp (hot-stage) 138-140 °C ($CHCl_3$) decomp.; λ_{max} (DCM)/nm 259 (log ε 4.11), 319 (4.09); ν_{max}/cm^{-1} 3108m (aryl C-H), 1731s (C=O), 1664s (C=O),

1562s, 1511m, 1408w, 1385m, 1299m, 1255m, 1226m, 1200w, 1151s, 1069m, 1040m, 943m, 911m, 877m, 854m, 815s, 803s, 774w, 732s; ^{1}H NMR ($CDCl_3$, 400 MHz) δ 9.09 (dd, 1H, J = 3.2, 1.2 Hz, Ar *H*), 8.30 (dd, 1H, J = 3.2, 1.2 Hz, Ar *H*), 7.91 (dd, 1H, J = 5.2, 1.6 Hz, Ar *H*), 7.57 (dd, 1H, J = 5.2, 1.2 Hz, Ar *H*), 7.48 (dd, 1H, J = 5.2, 3.2 Hz, Ar *H*), 7.40 (dd, 1H, J = 5.2, 2.8 Hz, Ar *H*); $^{13}C\{^1H\}$ NMR ($CDCl_3$, 100 MHz) δ 161.4, 161.0, 157.8, 138.8, 136.7, 133.6, 130.7, 128.6, 128.2, 127.5, 126.5; *m/z* (ESI+) calcd. for $C_{11}H_7N_2O_3S_3$ $[M+H]^+$ 310.9619, Found: 310.9608.

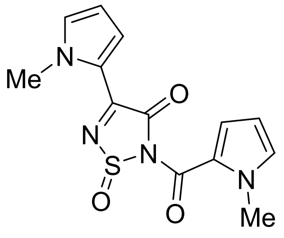

4-(1-Methyl-1*H*-pyrrol-2-yl)-2-(1-methyl-1*H*-pyrrole-2-carbonyl)-1,2,5-thiadiazol-3(2*H*)-one 1-oxide (5o). Similar treatment (**Method A**) of 3,5-bis(1-methyl-1*H*-pyrrol-2-yl)-4*H*-1,2,6-thiadiazin-4-one (**4o**) (10.2 mg, 0.0375 mmol) under 420 nm light for 90 min gave the *title compound* **5o** (11.4 mg, 100%) as brown plates, mp (hot-stage) 170-180 °C (Et_2O) decomp.; λ_{max} (DCM)/nm 296 (log ε 3.94), 376 (4.10); ν_{max}/cm^{-1} 3121w (aryl C-H), 1729m (C=O), 1664m (C=O), 1561m, 1512w, 1406m, 1389m, 1330m, 1246m, 1144m, 1064s, 1007m, 758s; ^{1}H NMR ($CDCl_3$, 400 MHz) δ 8.09 (dd, 1H, J = 4.4, 1.6 Hz, Ar *H*), 7.17 (dd, 1H, J = 2.0, 2.0 Hz, Ar *H*), 7.05 (dd, 1H, J = 4.4, 1.6 Hz, Ar *H*), 7.00 (dd, 1H, J = 2.0, 2.0 Hz, Ar *H*), 6.35 (dd, 1H, J = 4.4, 2.4 Hz, Ar *H*), 6.24 (dd, 1H, J = 4.4, 2.8 Hz, Ar *H*), 4.01 (s, 3H, C*H*$_3$), 3.98 (s, 3H, C*H*$_3$); $^{13}C\{^1H\}$ NMR ($CDCl_3$, 100 MHz) δ 159.2, 157.8, 155.6, 136.8, 134.0, 126.7, 124.4, 124.1, 122.7, 111.7, 109.6, 39.0, 37.4; *m/z* (ESI+) calcd. for $C_{13}H_{13}N_4O_3S$ $[M+H]^+$ 305.0708, Found: 305.0711.

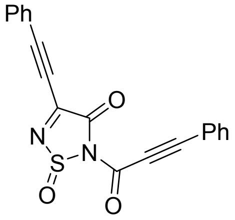

4-(Phenylethynyl)-2-(3-phenylpropioloyl)-1,2,5-thiadiazol-3(2*H*)-one 1-oxide (5q). Similar treatment (**Method A**) of 3,5-bis(phenylethynyl)-4*H*-1,2,6-thiadiazin-4-one (**4q**) (11.8 mg, 0.0375 mmol) under 420 nm light for 4 h gave the *title compound* **5q** (13.0 mg, 100%) as orange plates, mp (hot-stage) 126-127 °C ($CHCl_3$); λ_{max}(DCM)/nm 345 (log ε 4.20); ν_{max}/cm^{-1} 2197s (C≡C), 1756m (C=O), 1669m (C=O), 1595w, 1563m, 1489m, 1443m, 1290m, 1232m, 1158s, 1111s, 1069m, 1025m, 998m 959w, 900m, 812m, 755s; ^{1}H NMR ($CDCl_3$, 300 MHz) δ 7.74-7.78 (m, 3H, Ar *H*), 7.37-7.62 (m, 7H, Ar *H*); $^{13}C\{^1H\}$ NMR ($CDCl_3$, 100 MHz) δ 157.4, 154.8, 148.9, 134.1, 134.0, 132.7, 132.2, 129.1, 129.0, 119.1, 118.7, 110.2, 97.6, 81.0, 80.8; *m/z* (ESI+) calcd. for $C_{19}H_{11}N_2O_3S$ $[M+H]^+$ 347.0485, Found: 347.0465.

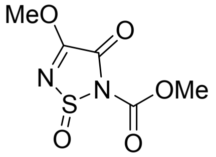

Methyl 4-methoxy-3-oxo-1,2,5-thiadiazole-2(3*H*)-carboxylate 1-oxide (5r). Similar treatment (**Method A**) of 3,5-dimethoxy-4*H*-1,2,6-thiadiazin-4-one (**4r**) (6.5 mg, 0.0375 mmol) under 420 nm light for 15 min gave the *title compound* **5r** (7.7 mg, 100%) as colorless plates, mp (hot-stage) 98-100 °C ($CHCl_3$); λ_{max}(DCM)/nm no absorption above 235 nm; ν_{max}/cm^{-1} 2961w (alkyl C-H), 1790s (C=O), 1729m (C=O), 1612s, 1441m, 1370w, 1265s, 1228s, 1157s, 1033s, 1011s, 945m, 894s, 846w, 894s, 846w, 787w, 760m, 741m; ^{1}H NMR ($CDCl_3$, 300 MHz) δ 4.21 (s, 3H, C*H*$_3$), 4.03 (s, 3H, C*H*$_3$); $^{13}C\{^1H\}$ NMR ($CDCl_3$, 100 MHz) δ 164.8, 151.6, 148.7, 59.1, 55.2; *m/z* (ESI-) calcd. for $C_5H_4N_2O_5SNa$ $[M+Na-2H]^-$ 226.9739, Found: 226.9780.

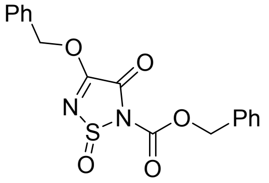

Benzyl 4-(benzyloxy)-3-oxo-1,2,5-thiadiazole-2(3*H*)-carboxylate 1-oxide (5s). Similar treatment (**Method A**) of 3,5-bis(benzyloxy)-4*H*-1,2,6-thiadiazin-4-one (**4s**) (12.6 mg, 0.0375 mmol) under 420 nm light for 15 min gave the *title compound* **5s** (13.4 mg, 100%) as colorless plates, mp (hot-stage) 79-81 °C ($CHCl_3$); λ_{max}(MeOH)/nm 232 (log ε 3.72); ν_{max}/cm^{-1} 3186w, 3061w and 3033w (aryl C-H), 1775m (C=O), 1745s (C=O), 1704w, 1683w, 1535w, 1495m, 1454m, 1405w, 1301s, 1227m, 1186s, 1079w, 1047s, 1018m, 943m, 906m, 770m, 744w, 728s; ^{1}H NMR ($CDCl_3$, 400 MHz) δ 7.37-7.48 (m, 10H, Ar *H*), 5.53 (s, 2H, CH_2), 5.42 (q, 2H, J = 12.0 Hz, CH_2); $^{13}C\{^1H\}$ NMR ($CDCl_3$, 100 MHz) δ 163.9, 151.8, 148.2, 133.9, 132.6, 129.8, 129.5, 129.2, 129.1, 129.0, 128.7, 74.6, 70.1; *m/z* (ESI+) calcd. for $C_{18}H_{19}N_2O_6S$ $[M+CH_3OH+H]^+$ 391.0964, Found: 391.0948.

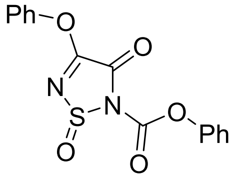

Phenyl 3-oxo-4-phenoxy-1,2,5-thiadiazole-2(3*H*)-carboxylate 1-oxide (5t). Similar treatment (**Method B**) of 3,5-diphenoxy-4*H*-1,2,6-thiadiazin-4-one (**4t**) (11.2 mg, 0.0375 mmol) under 420 nm light for 2 min gave the *title compound* **5t** (11.5 mg, 93%) as colorless plates, mp (hot-stage) 163-164 °C (Et_2O) decomp.; λ_{max}(DCM)/nm 258 (log ε 3.99); ν_{max}/cm^{-1} 1784m (C=O), 1732s (C=O), 1622m, 1583m, 1487s, 1457w, 1373m, 1277s, 1217m, 1187m, 1168s, 1157s, 1068w, 1026w, 1005m, 974w, 931m, 915w, 894m, 838m, 757s, 726s; ^{1}H NMR (Acetone-d_6, 400 MHz) δ 7.46-7.60 (m, 6H, Ar *H*), 7.42-7.46 (m, 1H, Ar *H*), 7.39 (tt, 1H, J = 7.6, 1.2 Hz, Ar *H*), 7.32-7.34 (m, 2H, Ar *H*); $^{13}C\{^1H\}$ NMR (Acetone-d_6, 100 MHz) δ 165.0, 153.1, 153.0, 150.8, 148.2, 131.0, 130.8, 128.3, 127.9, 122.2, 121.5; *m/z* (ESI+) calcd. for $C_{15}H_{11}N_2O_5S$ $[M+H]^+$ 331.0389, Found: 331.0388.

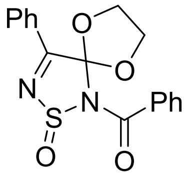

(2-Oxido-4-phenyl-6,9-dioxa-2-thia-1,3-diazaspiro[4.4]non-3-en-1-yl)(phenyl)methanone (5u). Similar treatment (**Method B**) of 6,10-diphenyl-1,4-dioxa-8-thia-7,9-diazaspiro[4.5]deca-6,9-diene (**4u**) (11.6 mg, 0.0375 mmol) under 420 nm light for 10 min gave the *title compound* **5u** (11.7 mg, 91%) as colorless needles, mp (hot-stage) 100-101 °C ($CHCl_3$) decomp.; λ_{max} (DCM)/nm 268.5 (log ε 5.02); ν_{max}/cm^{-1} 2916m (alkyl C-H), 1681s (C=O), 1596m, 1570m, 1489w, 1446m, 1361w, 1289s, 1206m, 1125m, 1051s, 977w, 948w, 896m, 849w, 794m, 746m 722m; ^{1}H NMR ($CDCl_3$, 400 MHz) δ 7.95-7.98 (m, 2H, Ar *H*), 7.66-7.71 (m, 2H, Ar *H*), 7.63 (tt, 1H, J = 6.2, 1.3 Hz, Ar *H*), 7.57 (tt, 1H, J = 7.5, 1.4 Hz, Ar *H*), 7.48-7.53 (m, 4H, Ar *H*), 4.74-4.79 (m, 1H, CH_2), 4.62-4.67 (m, 1H, CH_2), 4.28-4.38 (m, 2H, CH_2); $^{13}C\{^1H\}$ NMR ($CDCl_3$, 100 MHz) δ 176.7, 168.6, 135.0, 133.7, 132.2, 129.9, 129.5, 129.0, 128.9, 128.4, 119.6, 69.0; *m/z* (ESI+) calcd. for $C_{17}H_{15}N_2O_4S$ $[M+H]^+$ 343.0753, Found: 343.0770.

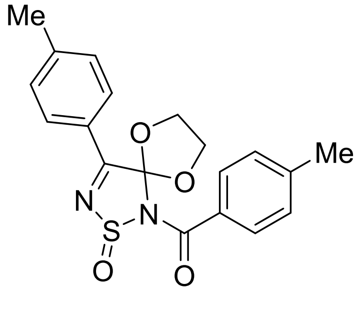

[2-Oxido-4-(p-tolyl)-6,9-dioxa-2-thia-1,3-diazaspiro[4.4]non-3-en-1-yl](p-tolyl)methanone (5v). Similar treatment (**Method B**) of 6,10-di-*p*-tolyl-1,4-dioxa-8-thia-7,9-diazaspiro[4.5]deca-6,9-diene (**4v**) (12.7 mg, 0.0375 mmol) under 420 nm light for 14 min gave the *title compound* **5v** (13.1 mg, 94%) as colorless needles, mp (hot-stage) 169-170 °C (Et_2O); λ_{max} (DCM)/nm 287

(log ε 4.12); ν_{max}/cm^{-1} 1677s (C=O), 1588s, 1557m, 1510w, 1485w, 1297s, 1183m, 1158m, 1127m, 1051s, 1017m, 977w, 947m, 900m, 858m, 823m, 790m, 752s, 702m; ^{1}H NMR (CDCl$_3$, 400 MHz) δ 7.87 (d, 2H, J = 8.4 Hz, Ar *H*), 7.60 (d, 2H, J = 8.4 Hz, Ar *H*), 7.30 (dd, 4H, J = 8.0, 0.4 Hz, Ar *H*), 4.65-4.83 (m, 2H, C*H*$_2$), 4.30-4.40 (m, 2H, C*H*$_2$), 2.45 (s, 3H, C*H*$_3$), 2.42 (s, 3H, C*H*$_3$); ^{13}C{^{1}H} NMR (CDCl$_3$, 100 MHz) δ 176.5, 168.7, 144.9, 142.8, 132.2, 129.8, 129.6, 129.5, 128.5, 127.0, 119.6, 69.03, 68.97, 21.9, 21.8; *m/z* (ESI+) calcd. for $C_{19}H_{19}N_2O_4S$ [M+H]$^+$ 371.1066, Found: 371.1071.

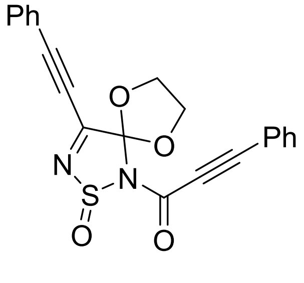

[2-Oxido-4-(phenylethynyl)-6,9-dioxa-2-thia-1,3-diazaspiro[4.4]non-3-en-1-yl]-3-phenylprop-2-yn-1-one (5w). Similar treatment (**Method B**) of 6,10-bis(phenylethynyl)-1,4-dioxa-8-thia-7,9-diazaspiro[4.5]deca-6,9-diene (**4w**) (13.4 mg, 0.0375 mmol) under 420 nm light for 60 min gave the *title compound* **5w** (13.1 mg, 90%) as beige plates, mp (hot-stage) 115-117 ° C (Et$_2$O); λ_{max} (DCM)/nm 277 (log ε 4.10), 286 (4.09), 320 inf (3.92); ν_{max}/cm^{-1} 2203s (C≡C), 1676s (C=O), 1601w, 1580s, 1490w, 1443w, 1393w, 1306s, 1231m, 1190s, 1150s, 1045s, 929m, 887m, 820m, 783w, 757s, 722m; ^{1}H NMR (CDCl$_3$, 400 MHz) δ 7.67 (d, 2H, J = 7.2 Hz, Ar *H*), 7.60-7.63 (m, 2H, Ar *H*), 7.46-7.54 (m, 2H, Ar *H*), 7.40-7.45 (m, 4H, Ar *H*), 4.63-4.70 (m, 2H, C*H*$_2$), 4.40-4.47 (m, 2H, C*H*$_2$); ^{13}C{^{1}H} NMR (CDCl$_3$, 100 MHz) δ 133.3, 133.2, 131.9, 131.5, 129.1, 128.9, 119.5, 119.0, 80.6, 69.4, 68.8. Five of the C resonances belonging to alkyne and phenyl groups are overlapping; *m/z* (ESI+) calcd. for $C_{21}H_{15}N_2O_4S$ [M+H]$^+$ 391.0753, Found: 391.0753.

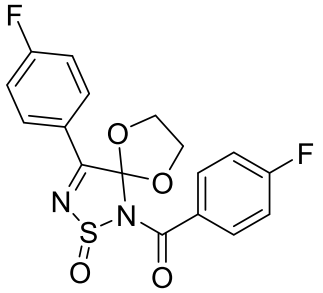

(4-Fluorophenyl)[4-(4-fluorophenyl)-2-oxido-6,9-dioxa-2-thia-1,3-diazaspiro[4.4]non-3-en-1-yl]methanone (5x). Similar treatment (**Method B**) of 6,10-bis(4-fluorophenyl)-1,4-dioxa-8-thia-7,9-diazaspiro[4.5]deca-6,9-diene (**4x**) (13.0 mg, 0.0375 mmol) under 420 nm light for 60 min gave the *title compound* **5x** (13.5 mg, 95%) as colorless needles, mp (hot-stage) 160-161 ° C (Et$_2$O); λ_{max} (DCM)/nm 276 (log ε 4.13); ν_{max}/cm^{-1} 2986w and 2908w (alkyl C-H), 1688s (C=O), 1600s, 1580s, 1507m, 1484w, 1408w, 1303s, 1240m, 1230m, 1208m, 1165w, 1157m, 1139s, 1123s, 1104w, 1052s, 1005s, 981m, 959m, 945s, 900s, 852m, 844s, 822w, 799w, 758s, 749s, 719w, 701w; ^{1}H NMR (CDCl$_3$, 400 MHz) δ 7.99-8.03 (m, 2H, Ar *H*), 7.71-7.75 (m, 2H, Ar *H*), 7.17-7.23 (m, 4H, Ar *H*), 4.69-4.82 (m, 2H, C*H*$_2$), 4.31-4.40 (m, 2H, C*H*$_2$); ^{13}C{^{1}H} NMR (CDCl$_3$, 100 MHz,) δ 174.5, 167.5, 166.3 (d, $^1J_{CF}$ = 255.0 Hz), 165.0 (d, $^1J_{CF}$ = 252.0 Hz), 132.2 (d, $^3J_{CF}$ = 9.0 Hz), 131.1 (d, $^3J_{CF}$ = 9.0 Hz), 125.9 (d, $^4J_{CF}$ = 3.0 Hz), 119.5, 116.5 (d, $^2J_{CF}$ = 22.0 Hz), 116.2 (d, $^2J_{CF}$ = 22.0 Hz), 69.1. One C resonance is missing; *m/z* (ESI+) calcd. for $C_{17}H_{13}F_2N_2O_4S$ [M+H]$^+$ 379.0564, Found: 379.0564.

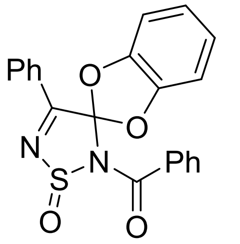

(1'-Oxido-4'-phenyl-2'H-spiro[benzo[d][1,3]dioxole-2,3'-[1,2,5]thiadiazol]-2'-yl)(phenyl)methanone (5y). Similar treatment (**Method B**) of 3',5'-diphenylspiro[benzo[*d*][1,3]dioxole-2,4'-[1,2,6]thiadiazine (**4y**) (13.4 mg, 0.0375 mmol) under 420 nm light for 10 min gave the *title compound* **5y** (14.0 mg, 96%) as a colorless needles, mp (hot-stage) 155-160 ° C (Et$_2$O) decomp.; λ_{max} (DCM)/nm 277 (log ε

3.81); ν_{max}/cm^{-1} 1698s (C=O), 1605m, 1595m, 1572w, 1482s, 1445m, 1362w, 1279s, 1261m, 1228s, 1197w, 1180w, 1129s, 1098m, 1063s, 1024m, 1001m, 980w, 961m, 918w, 796s, 739s, 717s; ^{1}H NMR ($CDCl_3$, 300 MHz) δ 7.86-7.90 (m, 2H, Ar *H*), 7.66-7.69 (m, 2H, Ar *H*), 7.59 (tt, 1H, J = 7.5, 1.2 Hz, Ar *H*), 7.52 (tt, 1H, J = 7.5, 1.5 Hz, Ar *H*), 7.36-7.45 (m, 4H, Ar *H*), 6.98-7.06 (m, 3H, Ar *H*), 6.89-6.91 (m, 1H, Ar *H*); ^{13}C{^{1}H} NMR ($CDCl_3$, 100 MHz) δ 172.3, 168.3, 146.1, 146.0, 134.7, 134.0, 132.5, 129.9, 129.3, 128.8, 128.5, 128.3, 123.4, 123.2, 121.0, 109.1, 108.7; *m/z* (ESI+) calcd. for $C_{21}H_{15}N_2O_4S$ [M+H]$^+$ 391.0753, Found: 391.0752.

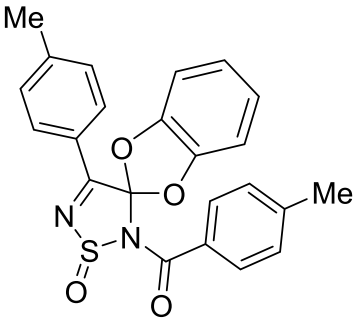

[1'-Oxido-4'-(*p*-tolyl)-2'*H*-spiro(benzo[*d*][1,3]dioxole-2,3'-[1,2,5]thiadiazol)-2'-yl](*p*-tolyl)methanone (5z). Similar treatment (**Method B**) of 3',5'-di-*p*-tolylspiro(benzo[*d*][1,3]dioxole-2,4'-[1,2,6]thiadiazine) (**4z**) (14.5 mg, 0.0375 mmol) under 420 nm light for 10 min gave the *title compound* **5z** (13.8 mg, 88%) as colorless needles, mp (hot-stage) 166-167 °C (Et_2O) decomp.; λ_{max}(DCM)/nm 284 (log ε 4.17), 297 inf (4.13); ν_{max}/cm^{-1} 1696m (C=O), 1591m, 1560w, 1481s, 1278s, 1229w, 1183w, 1136m, 1063w, 1018w, 961w, 910w, 826w, 804w, 780w, 749s; ^{1}H NMR ($CDCl_3$, 400 MHz) δ 7.77 (d, 2H, J = 8.4 Hz, Ar *H*), 7.58 (d, 2H, J = 8.4 Hz, Ar *H*), 7.21 (dd, 4H, J = 12.8, 7.6 Hz, Ar *H*), 6.98-7.05 (m, 3H, Ar *H*), 6.89-6.91 (m, 1H, Ar *H*), 2.38 (s, 6H, C*H*$_3$); ^{13}C{^{1}H} NMR ($CDCl_3$, 100 MHz) δ 172.2, 168.2, 146.22, 146.15, 143.2, 131.3, 130.1, 130.0, 129.5, 129.4, 128.7, 125.6, 123.3, 123.0, 121.0, 109.0, 108.6, 22.0, 21.8; *m/z* (ESI+) calcd. for $C_{23}H_{19}N_2O_4S$ [M+H]$^+$ 419.1066, Found: 419.1063.

S4.2.2 Asymmetric derivatives

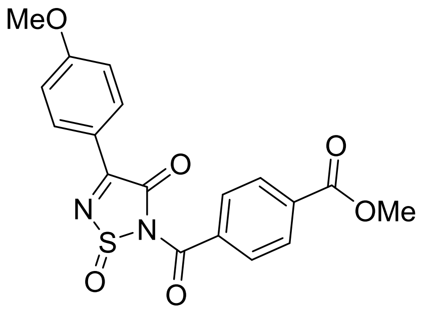

Methyl 4-[4-(4-methoxyphenyl)-1-oxido-3-oxo-2,3-dihydro-1,2,5-thiadiazole-2-carbonyl]benzoate (5aa). Similar treatment (**Method A**) of methyl 4-[5-(4-methoxyphenyl)-4-oxo-4*H*-1,2,6-thiadiazin-3-yl]benzoate (**4aa**) (13.3 mg, 0.0375 mmol) under 420 nm light for 10 min gave the *title compound* **5aa** (14.5 mg, 100%) as pale yellow needles, mp (hot-stage) 159-160 °C ($CHCl_3$) decomp.; λ_{max}(DCM)/nm 247 (log ε 4.58), 365 (4.40); ν_{max}/cm^{-1} 2958w, 2925w and 2844w (alkyl C-H), 1732m (C=O), 1719s (C=O), 1690s (C=O), 1603s, 1568s, 1529s, 1504s, 1487w, 1464m, 1437m, 1424m, 1405m, 1339w, 1312m, 1268s, 1254s, 1247m, 1212m, 1188w, 1166s, 1104s, 1069m, 1031m, 1007s, 959m, 910m, 844s, 791s, 776s, 736m, 715s; ^{1}H NMR ($CDCl_3$, 400 MHz) δ 8.51-8.54 (m, 2H, Ar *H*), 8.17-8.20 (m, 2H, Ar *H*), 7.84-7.87 (m, 2H, Ar *H*), 6.99-7.03 (m, 2H, Ar *H*), 3.97 (s, 3H, C*H*$_3$), 3.93 (s, 3H, C*H*$_3$); ^{13}C{^{1}H} NMR ($CDCl_3$, 100 MHz) δ 167.5, 166.1, 166.0, 164.8, 158.4, 135.4, 134.9, 133.8, 129.8, 121.0, 115.0, 55.9, 52.7, one C resonance missing; *m/z* (ESI+) calcd. for $C_{18}H_{15}N_2O_6S$ [M+H]$^+$ 387.0645, Found: 387.0628.

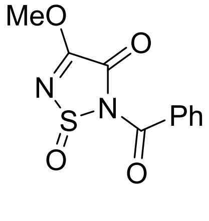

2-Benzoyl-4-methoxy-1,2,5-thiadiazol-3(2*H*)-one 1-oxide (5ab). Similar treatment (**Method A**) of 3-methoxy-5-phenyl-4*H*-1,2,6-thiadiazin-4-one (**4ab**) (8.1 mg, 0.0375

mmol) under 420 nm light for 15 min gave the *title compound* **5ab** (9.3 mg, 100%) as off white plates, mp (hot-stage) 79-81 °C ($CHCl_3$); λ_{max}(MeOH)/nm 268 inf (log ε 3.45), 339 (3.07); ν_{max}/cm^{-1} 3062w (alkyl C-H), 1747m (C=O), 1686 (C=O), 1600w, 1574w, 1502w, 1478m, 1438m, 1352w, 1305w, 1202s, 1155s, 1053m, 1025w, 1000w, 971w, 907m, 800m, 705s; ^{1}H NMR ($CDCl_3$, 400 MHz) δ 7.78 (d, 2H, J = 7.2 Hz, Ar *H*), 7.67 (dd, 1H, J = 8.0, 8.0 Hz, Ar *H*), 7.51 (dd, 2H, J = 7.6, 7.6 Hz, Ar *H*), 4.24 (s, 3H, CH_3); ^{13}C{^{1}H} NMR ($CDCl_3$, 100 MHz) δ 167.3, 164.6, 152.2, 134.7, 130.8, 130.3, 128.7, 59.1; HRMS analysis did not show peaks corresponding to any of the anticipated molecular adducts.

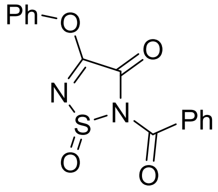

2-Benzoyl-4-phenoxy-1,2,5-thiadiazol-3(2*H*)-one 1-oxide (5ac). Similar treatment (**Method B**) of 3-phenoxy-5-phenyl-4*H*-1,2,6-thiadiazin-4-one (**4ac**) (10.6 mg, 0.0375 mmol) under 420 nm light for 5 min gave the *title compound* **5ac** (4.6 mg, 39%) as colorless needles, mp (hot-stage) >129 °C (Et_2O) decomp.; λ_{max}(DCM)/nm 256 (log ε 4.18), 283 inf (3.95); ν_{max}/cm^{-1} 2971w (aryl C-H), 1766s (C=O), 1691m (C=O), 1620s, 1581s, 1486s, 1364m, 1259s, 1253s, 1166m, 1050m, 1021m, 918w, 881m, 832w, 756m, 713m; ^{1}H NMR (Acetone-d_6, 400 MHz) δ 7.94-7.96 (m, 2H, Ar *H*), 7.73-7.77 (m, 1H, Ar *H*), 7.54-7.61 (m, 4H, Ar *H*), 7.40-7.46 (m, 3H, Ar *H*); Compound **5ac** is too insoluble to record a ^{13}C NMR spectrum; *m/z* (ESI+) calcd. for $C_{15}H_{10}N_2O_4SNa$ $[M+Na]^+$ 337.0259, Found: 337.0248.

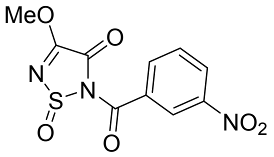

4-Methoxy-2-(3-nitrobenzoyl)-1,2,5-thiadiazol-3(2*H*)-one (5ad). Similar treatment (**Method B**) of 3-methoxy-5-(3-nitrophenyl)-4*H*-1,2,6-thiadiazin-4-one (**4ad**) (9.9 mg, 0.0375 mmol) under 420 nm light for 5 min gave the *title compound* **5ad** (9.1 mg, 82%) as off white plates, mp (hot-stage) >86 °C ($CHCl_3$) decomp.; λ_{max}(DCM)/nm 243 inf (log ε 3.70); ν_{max}/cm^{-1} 3088w (aryl C-H), 2954w (alkyl C-H), 1761s (C=O), 1694s (C=O), 1604s, 1583w, 1532s, 1478m, 1441m, 1351s, 1296m, 1259s, 1227m, 1180s, 1168s, 1103m, 1056m, 1010m, 930w, 913w, 889m, 868m, 817w, 799m, 784w, 758m, 718s, 706s; ^{1}H NMR ($CDCl_3$, 400 MHz) δ 8.57-8.62 (m, 1H, Ar *H*), 8.51 (ddd, 1H, J = 8.3, 2.3, 1.1 Hz, Ar *H*), 8.07 (ddd, 1H, J = 7.8, 1.7, 1.1 Hz, Ar *H*), 7.73 (ddd, 1H, J = 8.3, 7.9, 0.5 Hz, Ar *H*), 4.27 (s, 3H, CH_3); ^{13}C{^{1}H} NMR ($CDCl_3$, 100 MHz) δ 165.5, 164.4, 152.2, 148.2, 135.5, 132.4, 130.0, 128.6, 125.1, 59.4; *m/z* (ESI+) calcd. for $C_{10}H_8N_3O_5S$ $[M+H]^+$ 298.01, Found: 298.01. HRMS analysis did not show peaks corresponding to any of the anticipated molecular adducts. However, it has been reported that other nitro-thiadiazole 1-oxide compounds can produce specific signals for loss of oxygen and nitro groups.[47] After further analysis, the following fragment was found: (ESI+) calcd. for $C_{10}H_7N_2O_2$ $[M-NO_2-O]^+$ 235.0177, Found: 235.0182.

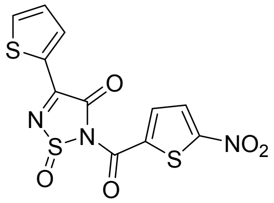

2-(5-Nitrothiophene-2-carbonyl)-4-(thien-2-yl)-1,2,5-thiadiazol-3(2*H*)-one 1-oxide (5ae). Similar treatment (**Method A**) of 3-(5-nitrothien-2-yl)-5-(thien-2-yl)-4*H*-1,2,6-thiadiazin-4-one (**4ae**) (12.1 mg, 0.0375 mmol) under 420 nm light for 60 min gave the *title compound* **5ae** (13.3 mg 100%) as orange needles, mp (hot-stage) 184-185 °C ($CHCl_3$); λ_{max}(DCM)/nm 322.5 (log ε 3.81), 370 inf (log ε 3.64); ν_{max}/cm^{-1} 3112m (aryl C-H), 2923m (aryl C-H), 1743m (C=O), 1662m (C=O), 1638w, 1553m, 1537m, 1508s, 1435m,

1354w, 1260m, 1193m, 1159s, 1122m, 1081m, 1046m, 992w, 863m, 815w, 767m, 729s; ^{1}H NMR ($CDCl_3$, 300 MHz) δ 8.61 (d, 1H, J = 3.6 Hz, Ar *H*), 8.01 (d, 1H, J = 4.8 Hz, Ar *H*), 7.93 (s, 2H, Ar *H*), 7.34 (t, 1H, J = 4.2, 4.2 Hz, Ar *H*); ^{13}C{^{1}H} NMR (DMSO-d_6, 100 MHz) δ 163.2, 157.6, 152.1, 150.4, 134.1, 133.6, 131.6, 129.8, 128.8, 124.9, 99.2; *m/z* (ESI+) calcd. for $C_{11}H_6N_3O_5S_3$ $[M+H]^+$ 355.9464, Found: 355.9453.

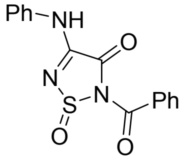

2-Benzoyl-4-(phenylamino)-1,2,5-thiadiazol-3(2*H*)-one 1-oxide (5af). Similar treatment (**Method B**) of 3-phenyl-5-(phenylamino)-4*H*-1,2,6-thiadiazin-4-one (**4af**) (10.5 mg, 0.0375 mmol) under 420 nm light for 5 min gave the *title compound* **5af** (11.0 mg, 94%) as off white needles, mp (hot-stage) 86-90 °C ($CHCl_3$) decomp.; λ_{max}(DCM)/nm 258 inf (log ε 3.95), 326 (3.76); ν_{max}/cm^{-1} 3297m (N-H), 3249br (N-H), 3140m and 3089m (aryl C-H), 1751s (C=O), 1721w, 1683s (C=O), 1624s, 1582s, 1495s, 1450s, 1267s, 1202m, 1178m, 1137s, 1084w, 1055m, 1020m, 1006m, 969w, 926w, 898s, 843m, 779m, 757s, 715m; ^{1}H NMR ($CDCl_3$, 400 MHz) δ 8.14 (s, 1H, N*H*), 7.80-7.85 (m, 2H, Ar *H*), 7.74-7.80 (m, 2H, Ar *H*), 7.63-7.72 (m, 1H, Ar *H*), 7.50-7.56 (m, 2H, Ar *H*), 7.42-7.49 (m, 2H, Ar *H*), 7.27-7.32 (m, 1H, Ar *H*); ^{13}C{^{1}H} NMR ($CDCl_3$, 100 MHz) δ 167.2, 156.4, 152.8, 135.5, 134.5, 131.5, 130.2, 129.8, 128.8, 126.8, 120.5; *m/z* (ESI+) calcd. for $C_{15}H_{12}N_3O_3S$ $[M+H]^+$ 314.0599, Found: 314.0582.

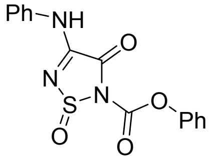

Phenyl 3-oxo-4-(phenylamino)-1,2,5-thiadiazole-2(3*H*)-carboxylate 1-oxide (5ag). Similar treatment (**Method B**) of 3-phenoxy-5-(phenylamino)-4*H*-1,2,6-thiadiazin-4-one (**4ag**) (11.2 mg, 0.0375 mmol) under 420 nm light for 2 min gave the *title compound* **5ag** (10.7 mg, 86%) as beige needles, mp (hot-stage) 155-165 °C (Et_2O) decomp.; λ_{max}(DCM)/nm 272 (log ε 3.48), 277 inf (3.46), 313 (3.36), 410 inf (2.02); ν_{max}/cm^{-1} 3262m (N-H), 3145w, 3078w (aryl C-H), 1801s (C=O), 1734m (C=O), 1629s, 1584s, 1492s, 1451m, 1331w, 1293s, 1263w, 1230m, 1186s, 1137s, 1073w, 1019s, 901s, 836w, 757s, 743s, 730s; ^{1}H NMR ($CDCl_3$, 400 MHz) δ 8.31 (s, 1H, N*H*), 7.80-7.82 (m, 2H, Ar *H*), 7.43-7.49 (m, 4H, Ar *H*), 7.28-7.34 (m, 4H, Ar *H*); ^{13}C{^{1}H} NMR ($CDCl_3$, 100 MHz) δ 156.2, 152.6, 149.8, 147.0, 135.4, 129.9, 129.8, 127.2, 126.9, 121.3, 120.7; *m/z* (ESI+) calcd. for $C_{15}H_{12}N_3O_4S$ $[M+H]^+$ 330.0549, Found: 330.0568.

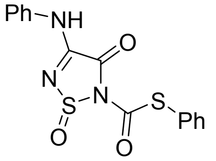

S*-Phenyl 3-oxo-4-(phenylamino)-1,2,5-thiadiazole-2(3*H*)-carbothioate 1-oxide (5ah).* Similar treatment (**Method B**) of 3-(phenylamino)-5-(phenylthio)-4*H*-1,2,6-thiadiazin-4-one (**4ah**) (11.8 mg, 0.0375 mmol) under 420 nm light for 2 min gave the *title compound* **5ah** (11.8 mg, 91%) as beige needles, mp (hot-stage) 155-165 °C (Et_2O) decomp.; λ_{max}(DCM)/nm 330 (log ε 3.94); ν_{max}/cm^{-1} 3275m (N-H), 3075w (aryl C-H), 1742s (C=O), 1678s (C=O), 1630s, 1589s, 1494m, 1475m, 1452m, 1441m, 1342w, 1328w, 1284s, 1256m, 1208m, 1153s, 1096m, 1073s, 1023m, 1009m, 967m, 896s, 838m, 775w, 758s, 747s; ^{1}H NMR ($CDCl_3$, 400 MHz) δ 8.20 (s, 1H, N*H*), 7.77-7.79 (m, 2H, Ar *H*), 7.55-7.57 (m, 2H, Ar *H*), 7.45-7.52 (m, 4H, Ar *H*), 7.28-7.44 (m, 2H, Ar *H*); ^{13}C{^{1}H} NMR ($CDCl_3$, 100 MHz) δ 171.0, 166.4, 156.4, 152.6, 135.5, 135.4, 130.7, 129.8, 129.7, 129.2, 127.7, 126.9, 120.6; *m/z* (ESI+) calcd. for $C_{15}H_{12}N_3O_3S_2$ $[M+H]^+$ 346.0320, Found: 346.0326.

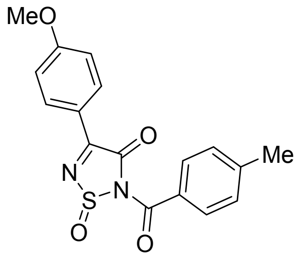

5ai (major)

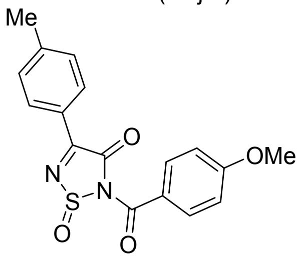

5al (minor)

4-(4-Methoxyphenyl)-2-(4-methylbenzoyl)-1,2,5-thiadiazol-3(2*H*)-one 1-oxide (5ai) and 2-(4-methoxybenzoyl)-4-(p-tolyl)-1,2,5-thiadiazol-3(2*H*)-one 1-oxide (5al). Similar treatment (**Method A**) of 3-(4-methoxyphenyl)-5-(*p*-tolyl)-4*H*-1,2,6-thiadiazin-4-one (**4ai**) (11.6 mg, 0.0375 mmol) under 420 nm light for 15 min gave the *title compounds* **5ai** and **5al** as an inseparable mixture (molar ratio **5ai**/**5al** 69:31 as judged by the integrations of the peaks at δ 8.53-8.56 and 8.41 in the ^{1}H NMR spectrum) (12.8 mg, 100%) as beige needles, mp (hot-stage) 159-166 °C ($CHCl_3$) decomp.; λ_{max}(DCM)/nm 255 (log ε 3.94), 357 (3.86); ν_{max}/cm^{-1} 2940w (alkyl C-H), 1732s (C=O), 1698s (C=O), 1604s, 1577m, 1547s, 1511s, 1456w, 1425m, 1383w, 1321w, 1259s, 1210m, 1183w, 1168s, 1150s, 1062s, 1015s, 906m, 846m, 826m, 798w, 763s, 742s, 717w; ^{1}H NMR ($CDCl_3$, 400 MHz) crude after completion of the reaction containing **5ai** and **5al**: δ 8.53-8.56 (m, 2.04H, Ar *H*), 8.41 (d, 0.90H, J = 8.4 Hz, Ar *H*), 7.83-7.87 (m, 0.94H, Ar *H*), 7.74 (dd, 2.06H, J = 6.4, 1.6 Hz, Ar *H*), 7.31-7.35 (m, 3.03H, Ar *H*), 6.99-7.01 (m, 3.06H, Ar *H*), 3.92 (s, 3H, CH_3O), 3.91 (s, 1.40H, CH_3O), 2.47 (s, 1.35H, CH_3), 2.46 (s, 3.03H, CH_3); $^{13}C\{^1H\}$ NMR ($CDCl_3$, 100 MHz) crude after completion of the reaction containing **5ai** and **5al**: δ 167.8, 166.9, 166.0, 165.9, 165.0, 164.8, 158.5, 158.2, 147.1, 145.8, 133.8, 133.2, 131.2, 130.4, 130.1, 129.5, 128.8, 125.9, 123.5, 121.1, 114.9, 114.2, 55.9, 55.8, 22.2, 22.1; *m/z* (ESI+) calcd. for $C_{17}H_{15}N_2O_4S$ [M+H]$^+$ 343.0753, Found: 343.0736. The assignment of the structures for **5ai** and **5al** is tentative and was based on comparison of the ^{1}H and ^{13}C NMR spectra of the mixture of the two compounds (focusing on the peaks of the CH_3O and CH_3 groups) with that of **5b** and **5e**.

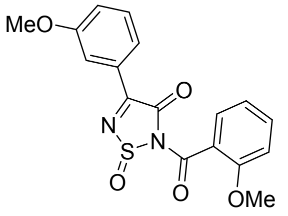

5aj (major)

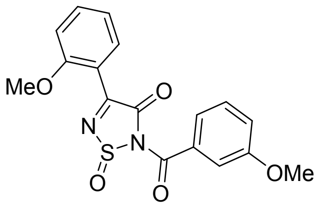

5am (minor)

2-(2-Methoxybenzoyl)-4-(3-methoxyphenyl)-1,2,5-thiadiazol-3(2*H*)-one 1-oxide (5aj)* and *2-(3-methoxybenzoyl)-4-(2-methoxyphenyl)-1,2,5-thiadiazol-3(2*H*)-one 1-oxide (5am). Similar treatment (**Method A**) of 3-(2-methoxyphenyl)-5-(3-methoxyphenyl)-4*H*-1,2,6-thiadiazin-4-one (**4aj**) (12.2 mg, 0.0375 mmol) under 420 nm light for 10 min gave the *title compounds* **5aj** and **5am** as an inseparable mixture (molar ratio **5aj**/**5am** 60:40 as judged by the integrations of the peaks at δ 3.91 and 3.80 in the ^{1}H NMR spectrum) (13.4 mg, 100%) as yellow needles, mp (hot-stage) 115-127 °C ($CHCl_3$) decomp.; λ_{max}(DCM)/nm 262 (log ε 3.73), 306 (3.58), 381 inf (3.12); ν_{max}/cm^{-1} 2942w and 2837w (alkyl C-H), 1746s (C=O), 1700s (C=O), 1599s, 1585s, 1489s, 1463s, 1427m, 1333m, 1299w, 1276m, 1230s, 1196w, 1149s, 1121w, 1067m, 1033s, 1019s, 984w, 924m, 897w, 882m, 842w, 809w, 798w, 788w, 753s, 740s, 715s; ^{1}H NMR ($CDCl_3$, 400 MHz) crude after completion of the reaction containing **5aj** and **5am**: δ 8.11 (ddd, 0.67H, J = 8.0, 1.6, 1.2 Hz, Ar *H*), 7.93 (dd, 0.65H, J = 2.4, 1.6 Hz, Ar *H*), 7.76 (ddd, 1H, J = 7.6, 1.6, 0.4 Hz, Ar *H*), 7.53-7.61 (m, 2.42H, Ar *H*), 7.40-

7.44 (m, 2.81H, Ar *H*), 7.33-7.34 (m, 1H, Ar *H*), 7.19-7.21 (m, 1.77H, Ar *H*), 7.01-7.11 (m, 2.80H, Ar *H*), 6.99 (d, 0.80H, J = 8.4 Hz, Ar *H*), 3.91 (s, 3H, C*H*$_3$O), 3.86 (s, 5.04H, C*H*$_3$O), 3.80 (s, 1.98H, C*H*$_3$O); $^{13}C\{^1H\}$ NMR ($CDCl_3$, 100 MHz) crude after completion of the reaction containing **5aj** and **5am**: δ 169.5, 167.8, 166.2, 160.0, 159.7, 157.7, 157.2, 157.0, 135.5, 134.4, 132.6, 132.0, 130.5, 130.3, 129.73, 129.68, 123.8, 122.6, 122.4, 122.2, 121.1, 120.7, 117.8, 114.9, 114.5, 112.4, 111.5, 56.3, 56.0, 55.72, 55.70; *m/z* (ESI+) calcd. for $C_{17}H_{15}N_2O_5S$ $[M+H]^+$ 359.0702, Found: 359.0696. The assignment of the structures for **5aj** and **5am** is tentative and was based on comparison of the ^{1}H and ^{13}C NMR spectra of the mixture of the two compounds (focusing on the peaks of the CH_3O groups) with that of **5e** and **5f**.

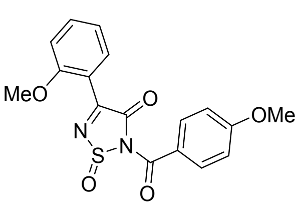

5ak (major)

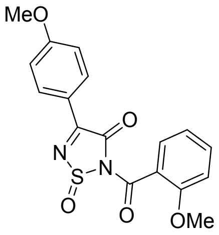

5an (minor)

2-(4-Methoxybenzoyl)-4-(2-methoxyphenyl)-1,2,5-thiadiazol-3(2*H*)-one 1-oxide (5ak) and 2-(2-methoxybenzoyl)-4-(4-methoxyphenyl)-1,2,5-thiadiazol-3(2*H*)-one 1-oxide (5an). Similar treatment (**Method A**) of 3-(2-methoxyphenyl)-5-(4-methoxyphenyl)-4*H*-1,2,6-thiadiazin-4-one (**4ak**) (12.2 mg, 0.0375 mmol) under 420 nm light for 10 min gave the *title compounds* **5ak** and **5an** as an inseparable mixture (molar ratio **5ak**/**5an** 60:40 as judged by the integrations of the peaks at δ 3.90 and 3.80 in the ^{1}H NMR spectrum) (13.4 mg, 100%) as yellow needles, mp (hot-stage) 159-176 °C ($CHCl_3$) decomp.; λ_{max}(DCM)/nm 257 (log ε 3.51), 283 inf (3.36), 362 (3.43); ν_{max}/cm^{-1} 2941w and 2841w (alkyl C-H), 1749s (C=O), 1687s (C=O), 1601s, 1532w, 1513m, 1489s, 1461m, 1437m, 1332w, 1308w, 1260s, 1201m, 1173s, 1165s, 1153s, 1120m, 1069m, 1046m, 1020s, 935w, 906m, 847s, 815m, 788w, 776w, 759s, 746s, 730m, 712s; ^{1}H NMR ($CDCl_3$, 400 MHz) crude after completion of the reaction containing **5ak** and **5an**: δ 8.49-8.52 (m, 1.99H, Ar *H*), 7.84-7.86 (m, 1.32H, Ar *H*), 7.77 (ddd, 0.66H, J = 7.6, 2.0, 0.4 Hz, Ar *H*), 7.54-7.62 (m, 1.73H, Ar *H*), 7.52 (ddd, 0.98H, J = 7.6, 1.6, 0.4 Hz, Ar *H*), 7.06-7.11 (m, 2.38H, Ar *H*), 6.97-7.00 (m, 4.45H, Ar *H*), 3.913 (s, 2.10H, C*H*$_3$O), 3.910 (s, 2.91H, C*H*$_3$O), 3.90 (s, 1.98H, C*H*$_3$O), 3.80 (s, 3H, C*H*$_3$O); $^{13}C\{^1H\}$ NMR ($CDCl_3$, 100 MHz) crude after completion of the reaction containing **5ak** and **5an**: δ 169.6, 166.9, 166.3, 165.8, 165.1, 165.0, 160.1, 157.80, 157.76, 157.4, 135.3, 134.2, 133.6, 133.2, 132.0, 130.4, 121.4, 121.1, 114.9, 114.1, 112.5, 111.6, 56.4, 56.1, 55.84, 55.77; *m/z* (ESI+) calcd. for $C_{17}H_{15}N_2O_5S$ $[M+H]^+$ 359.0702, Found: 359.0699. The assignment of the structures for **5ak** and **5an** is tentative and was based on comparison of the ^{1}H and ^{13}C NMR spectra of the mixture of the two compounds (focusing on the peaks of the CH_3O groups) with that of **5e** and **5g**.

S4.3 Characterization of isolated side-products and reference compounds

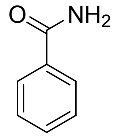

Benzamide (22). To a stirred solution of 2-benzoyl-4-phenyl-1,2,5-thiadiazol-3(2*H*)-one 1-oxide (**5a**) (10.0 mg, 0.034 mmol) in dry DCM (5 mL) was added Ph_3P (0.068 mmol, 2equiv.) and CCl_4 (10 equiv.). The resulting solution was heated to 40 °C (reflux) and stirred for 24 h, after which the thiadiazole **5a** was consumed (monitored by ^{1}H NMR). The resulting solution was then concentrated under reduced pressure, and the title compound **22** was isolated after chromatography ($CHCl_3$/*n*-hexane, 50:50) as a colorless solid (1.5 mg, 37%); ^{1}H NMR ($CDCl_3$, 400 MHz) δ 7.81-7.83 (m, 2H, Ar *H*),

7.54 (tt, 1H, J = 7.2, 1.6 Hz, Ar *H*), 7.43-7.47 (m, 2H, Ar *H*), 6.11 (s, 1H, NH_2), 5.82 (s, 1H, NH_2); $^{13}C\{^1H\}$ NMR ($CDCl_3$, 100 MHz) δ 169.5, 133.5, 132.2, 128.8, 127.5; LC (ret. time): 4.040 min; *m/z* (ESI+) calcd. for $C_{12}H_{15}N_2$ $[2M+H]^+$ 243.11, Found: 243.15. Identical to spectra obtained from commercially available sample.

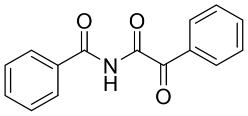

N-*(2-Oxo-2-phenylacetyl)benzamide (23).* The title compound was synthesized according to known procedures[48] and was obtained as a yellow solid (0.88 g , 59%); ^1H NMR ($CDCl_3$, 300 MHz) δ 9.78 (s, 1H, N*H*), 8.12 (dd, 2H, J = 8.7, 1.5 Hz, Ar *H*), 7.88-7.93 (m, 2H, Ar *H*), 7.61-7.69 (m, 2H, Ar *H*), 7.51-7.55 (m, 4H, Ar *H*); $^{13}C\{^1H\}$ NMR ($CDCl_3$, 75 MHz) δ 186.7, 165.4, 161.0, 134.9, 134.1, 132.5, 131.2, 130.4, 129.30, 129.1, 128.2; LC (ret. time): 5.581 min; *m/z* (ESI+) calcd. for $C_{15}H_{12}NO_3$ $[M+H]^+$ 254.08, Found: 254.00. Identical to published spectra.[48,49]

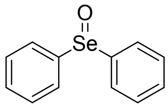

Seleninyldibenzene (24). The title compound was synthesized according to known procedures[38] and was obtained as a colorless solid (0.20 g , 10%); ^1H NMR ($CDCl_3$, 400 MHz) δ 7.69-7.71 (4H, m, Ar *H*), 7.46-7.48 (6H, m, Ar *H*); $^{13}C\{^1H\}$ NMR ($CDCl_3$, 100 MHz) δ 142.7, 131.5, 129.9, 126.4; LC (ret. time): 4.737 min; *m/z* (ESI+) calcd. for $C_{12}H_{11}OSe$ $[M+H]^+$ 251.00, Found: 251.05. Identical to published spectra.[38]

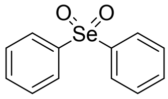

Selenonyldibenzene (25). The title compound was synthesized according to known procedures[50] and was obtained as a colorless solid (0.19 g , 72%); ^1H NMR (CD_2Cl_2, 400 MHz) δ 7.96-7.98 (m, 4H, Ar *H*), 7.67-7.69 (m, 2H, Ar *H*), 7.61-7.65 (m, 4H, Ar *H*); $^{13}C\{^1H\}$ NMR (CD_2Cl_2, 100 MHz) δ 142.9, 134.1, 130.3, 126.9; LC (ret. time): 4.740 min; *m/z* (ESI+) calcd. for $C_{12}H_{11}O_2Se$ $[M+H]^+$ 266.99, Found: 267.05. Identical to published spectra.[51]

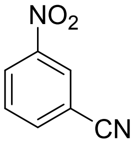

3-Nitrobenzonitrile (26). Similar treatment (**Method A**) of 3-methoxy-5-(3-nitrophenyl)-4*H*-1,2,6-thiadiazin-4-one (**4ad**) (9.9 mg, 0.0375 mmol) under 420 nm light for 24 h led to its consumption (by ^1H NMR) and the concomitant appearance of multiple new spots on TLC ($CHCl_3$/EtOAc, 90:10). The reaction mixture was subjected to column chromatography ($CHCl_3$/EtOAc, 90:10), and the major band was isolated. After the solvent was removed, the title compound **26** was isolated (0.5 mg, 9%) as colorless needles, mp (hot-stage) 115-116 °C ($CHCl_3$), lit. mp[52] 115-117 °C (EtOH); ν_{max}/cm^{-1} 3080m (aryl C-H), 2237m (C≡N), 1618m, 1535s, 1474m, 1434w, 1335s, 1202w, 1102m, 1080w, 921m, 904m, 817m, 789m, 735m; ^1H NMR ($CDCl_3$, 400 MHz) δ 8.47 (t, 1H, J = 1.7 Hz, Ar *H*), 8.41 (ddd, 1H, J = 8.4, 2.3, 1.1 Hz, Ar *H*), 7.89-7.96 (m, 1H, Ar *H*), 7.61-7.71 (m, 1H, Ar *H*); $^{13}C\{^1H\}$ NMR ($CDCl_3$, 100 MHz) δ 148.4, 137.7, 130.8, 127.7, 127.4, 116.7, 114.3. Identical to published spectra.[52]

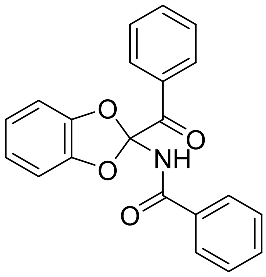

N-*(2-Benzoylbenzo[*d*][1,3]dioxol-2-yl)benzamide (27).* Similar treatment (**Method A**) of 3',5'-diphenylspiro[benzo[*d*][1,3]dioxole-2,4'-[1,2,6]thiadiazine] (**4z**) (13.4 mg, 0.0375 mmol) under 420 nm light in $CDCl_3$ (1 mL) at *ca.* 20 °C was irradiated with 420 nm light until it was consumed (TLC, $CHCl_3$/*n*-hexane, 50:50) over a period of

3 days. The resulting solution turned cloudy, and TLC analysis revealed the formation of multiple spots, indicative of decomposition by-products. After removal of the solvent under reduced pressure, the crude mixture was subjected to a preparative TLC separation (DCM/*n*-hexane, 50:50). The major band was isolated, extracted with $CHCl_3$, and was then dried under reduced pressure to afford the *title compound* **27** (2.5 mg, 19%) as colorless needles, mp (hot-stage) 120-123 °C ($CHCl_3$); R_f 0.66 ($CHCl_3$/EtOAc, 95:5); λ_{max}(DCM)/nm 254 inf (log ε 4.05), 273 inf (3.86); ν_{max}/cm^{-1} 3258br (N-H), 1708m (C=O), 1666s (C=O), 1600w, 1580w, 1516m, 1478s, 1464w, 1359m, 1314w, 1260w, 1233s, 1179w, 1099s, 1026w, 1003m, 909s, 901m, 801w, 729s, 709s; ^{1}H NMR ($CDCl_3$, 400 MHz) δ 8.32 (s, 1H, N*H*), 8.00-8.02 (m, 2H, Ar *H*), 7.82-7.84 (m, 2H, Ar *H*), 7.52-7.59 (m, 2H, Ar *H*), 7.38-7.47 (m, 4H, Ar *H*), 6.97-7.02 (m, 4H, Ar *H*); ^{13}C{^{1}H} NMR (Acetone-d_6, 100 MHz) δ 185.6, 167.4, 146.7, 134.6, 133.9, 133.5, 133.3, 130.0, 129.5, 129.2, 128.6, 123.2, two C resonances missing; *m/z* (ESI+) calcd. for $C_{21}H_{15}NO_4$ $[M]^+$ 345.10, Found: 345.09.

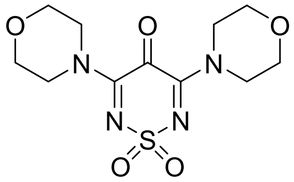

3,5-Dimorpholino-4*H*-1,2,6-thiadiazin-4-one 1,1-dioxide (18a). Similar treatment (**Method A**) of 3,5-dimorpholino-4*H*-1,2,6-thiadiazin-4-one (**15a**) (10.7 mg, 0.0375 mmol) under 420 nm light for 24 h led to its consumption (by TLC) and the concomitant appearance of multiple new spots on TLC (EtOAc). The reaction mixture was subjected to column chromatography (EtOAc), and the major band was isolated. After the solvent was removed, the *title compound* **18a** was isolated (7.4 mg, 62%) as yellow needles, mp (hot-stage) 252-254 °C ($CHCl_3$); R_f 0.19 (EtOAc); ^{1}H NMR (Acetone-d_6, 300 MHz) δ 3.70-3.65 (m, 8H, C*H$_2$*), 3.62-3.66 (m, 8H, *CH$_2$*); ^{13}C{^{1}H} NMR (Acetone-d_6, 75 MHz) δ 176.8, 157.1, 67.0, 66.5, 49.0, 44.9; *m/z* (ESI-) calcd. for $C_{11}H_{16}N_4O_5S$ $[M]^-$ 316.08, Found: 316.10. Identical to published spectra.[26]

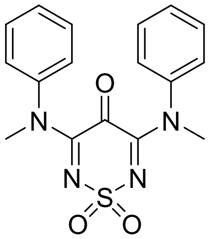

3,5-Bis[methyl(phenyl)amino]-4*H*-1,2,6-thiadiazin-4-one 1,1-dioxide (18b). Similar treatment (**Method A**) of 3,5-bis(methyl(phenyl)amino)-4H-1,2,6-thiadiazin-4-one (**15b**) (12.2 mg, 0.0375 mmol) under 420 nm light for 24 h led to its consumption (by ^{1}H NMR) and the concomitant appearance of multiple new spots on TLC ($CHCl_3$/EtOAc, 90:10). The reaction mixture was subjected to column chromatography ($CHCl_3$/EtOAc, 90:10), and the major band was isolated. After the solvent was removed, the *title compound* **18b** was isolated (5.9 mg, 44%) as orange needles, R_f 0.80 (EtOAc); mp (hot-stage) 260-262 °C ($CHCl_3$); ^{1}H NMR (500 MHz, DMSO-d_6) δ 7.20-7.42 (m, 10H, Ar *H*), 3.32 (s, 6H, *CH$_3$*); ^{13}C{^{1}H} NMR (DMSO-d_6, 125 MHz) δ 155.4, 142.3, 129.4, 127.8, 126.0, 40.5; *m/z* (ESI-) calcd. for $C_{17}H_{15}N_4O_3S$ $[M-H]^-$ 355.08, Found: 355.05. Identical to published spectra.[26]

S5. Single Crystal X-ray Diffraction Data Collection and Refinement Details and Discussion

Single crystals were grown by slow evaporation or solvent diffusion with details of each given below. Single crystals of **4m**, **5a**, **5u**, **5m**, **5ah**, **5ah**, **5ac**, **5aa**, **23** and **27**, were grown by slow evaporation of Chloroform Single crystals of **5b**, **5i**, **5l** and **5n** were grown by slow evaporation of acetonitrile. Single crystals of **5x**, and **18b** were grown by slow evaporation of diethyl ether. Single crystals of **5af** were grown by slow evaporation of acetone. Single crystals of **4a** were grown by slow evaporation of dichloromethane. Single crystals of **18a**, **17a** and **17b** were grown by solvent diffusion of chloroform and acetone.
Each crystal was coated in paraffin oil and mounted on a Molecular Dimensions Litholoop and placed directly into the cold stream of the Bruker D8 Venture diffractometer. Single crystal x-ray diffraction data were collected using either a Cu-K_{α} (λ = 1.5418 Å) IμS 3.0 microfocus source, or a Mo sealed tube with Triumph monochromator, using Bruker's APEX3 program suite, with the crystal kept at 100.0 K during data collection. The structures were solved using Olex2, using the SHELXT structure solution program using Intrinsic Phasing and refined with the SHELXL refinement package using Least Squares minimisation.[1–2]
X-ray structures were determined for **4a**, **4m**, **5a**, **5b**, **5i**, **5l**, **5m**, **5n**, **5u**, **5x**, **5aa**, **5ac**, **5af**, **5ah**, **17a**, **17b**, **18a**, **18b**, **23** and **27**.

Compounds **4a**, **5u**, **5ad** and **27**, were refined as two component twins with hklf 4 data with the twin law (-1 0 0, 0 -1 0, 0 0 -1), *i.e.*, *hkl* data from one domain only.
The crystal structure of thiadiazole **5a** contains notably short intermolecular S···O contacts [2.888(3) Å] where the S-O bonds stack on top of each other parallel to the *b*-axis. This may have implications for the reactivity of **5a**. The structures of compounds **5a**, **5b**, **5i**, **5m** and **5x** were confirmed by single crystal XRD as racemates. All crystallized in centrosymmetric space groups despite the chiral sulfur atom. However, compound **5u** crystallized in the chiral space group $P2_12_12_1$, albeit as a two-component twin and compound **5n** crystallized in the polar space group $Pna2_1$ where the S lone pair faces in one direction parallel to the *c* axis. Close intermolecular S···O contacts were observed in thiadiazole **5i** [2.782(3) Å], running parallel to the *a*-axis. These close contacts were also observed in the structure of thiadiazole **5a** (*vide supra*) which differs only in the phenyl meta substituents (H *cf.* Cl). The structure of compound **5l** was supported by X-ray diffraction and crystallized in the polar space group Cc with molecules packing in such a way that the S lone pair faces in one direction along the *a*-axis. The structures of compounds **5aa**, **5af** and **5ah** were supported by single crystal XRD as racemates in P21/c while compound **5ac** crystallized in the chiral space group $P2_12_12_1$, albeit as a two-component twin**.**
In **4m**, **5m** and **5n** there was substitutional disorder in the five-membered rings between carbon and sulfur atoms. The coordinates and displacement parameters were constrained to be the same at the disordered positions with the commands EXYZ and EADP and the relative occupancies tied so that the occupancies of the positions and constituent elements summed to 100%.
In **5b** there is 2-fold rotational disorder in the phenyl ring along the Cipso-Me axis. The occupancies were fixed at 50:50 and the displacement parameters in the two conformations are the same.

The structure of **23** only solved in P1. A Flack parameter was obtained 0.3(2) There are warnings that this could be P-1 however all the largest correlation matrix elements are between atoms within the same molecule. There are 8 molecules on the asymmetric unit. The data were refined against *hklf* 5 data which were compiled from the OLEX2 twinning processing procedure. There is a mixture of isotropic and anisotropic refinement chosen on the basis of atomic parameter refinement stability and standard deviation on the Flack parameter. RIGU (rigid bond restraint) and isotropic restraints were applied.

CCDC deposition numbers and structure codes

Paper Code	Deposition Number
**4a**	2168267
**4m**	2168266
**5a**	2167996
**5b**	2168000
**5i**	2168157
**5l**	2168147
**5m**	2168187
**5n**	2168217
**5u**	2167997
**5x**	2167999
**5aa**	2168224
**5ac**	2168220
**5af**	2168218
**5ah**	2168219
**17a**	2173994
**17b**	2173995
**18a**	2168263
**18b**	2168264
**23**	2168225[a]
**27**	2168226

a) This was recorded at 100 K, albeit this compound's crystal structure was previously reported at 293 K. Hoong-Kun Fun, Jia Hao Goh, Dongdong Wu, Yan Zhang (2011) Acta Crystallogr., Sect. E: Struct. Rep. Online ,67, o197; CCDC Ref code QAKREB

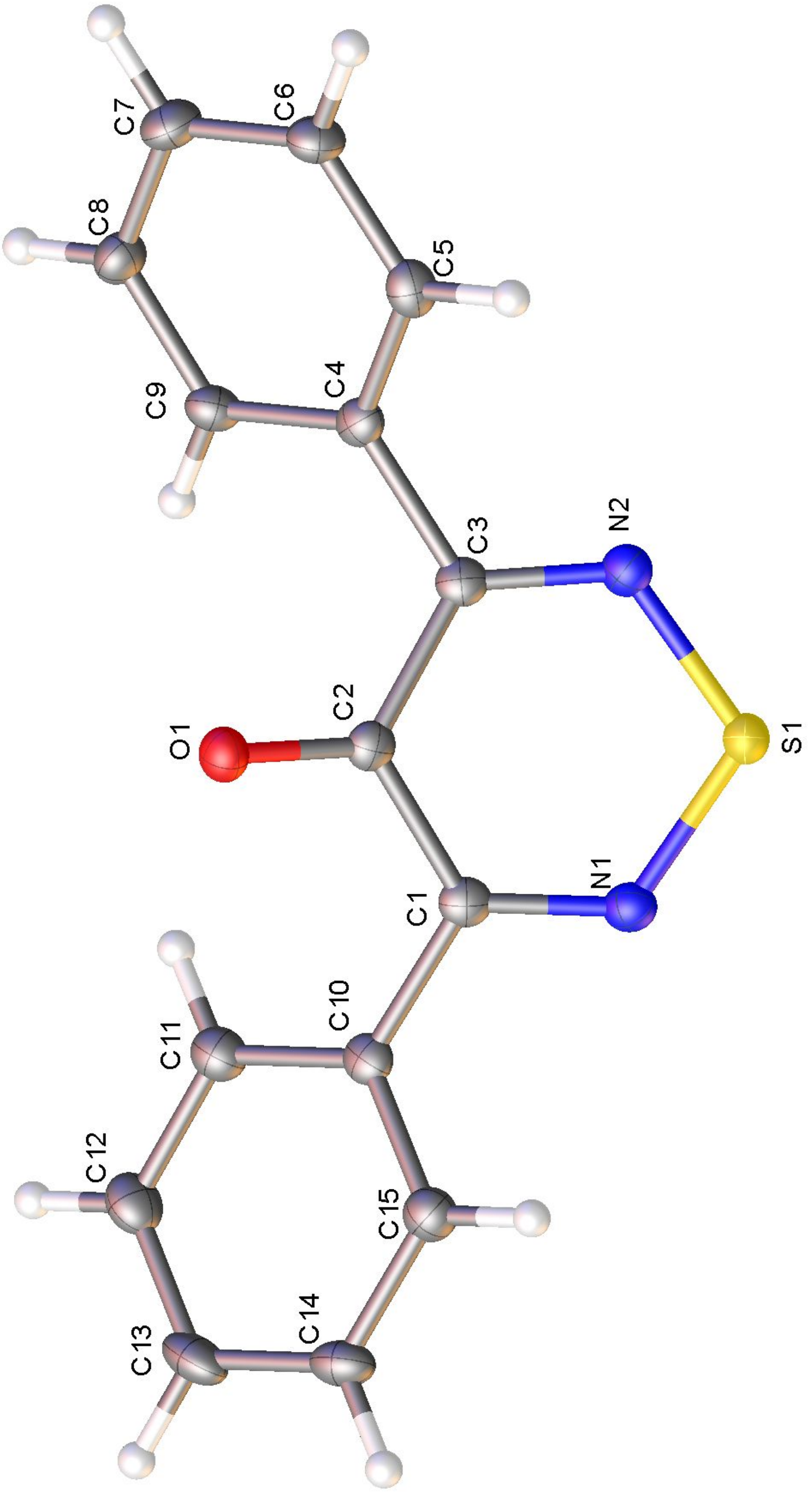

Perspective view of **4a** with displacement ellipsoids drawn at the 50% probability level

Crystal data and structure refinement for **4a**

Identification code	4a
Empirical formula	$C_{15}H_{10}N_2OS$
Formula weight	266.31
Temperature/K	100.0
Crystal system	Trigonal
Space group	$P3_1$
a/Å	16.6569(10)
b/Å	16.6569(10)
c/Å	3.7166(3)
α/°	90
β/°	90
γ/°	120
Volume/Å^3	893.03(13)
Z	3
ρ_{calc}g/cm^3	1.486
μ/mm^{-1}	2.343
F(000)	414.0
Crystal size/mm^3	0.24 × 0.04 × 0.03
Radiation	CuKα (λ = 1.54178)
2Θ range for data collection/°	6.126 to 148.926
Index ranges	$-20 \le h \le 17$, $-20 \le k \le 20$, $-4 \le l \le 4$
Reflections collected	13130
Independent reflections	2410 [R_{int} = 0.0621, R_{sigma} = 0.0380]
Data/restraints/parameters	2410/148/173
Goodness-of-fit on F^2	1.059
Final R indexes [I>=2σ (I)]	R_1 = 0.0279, wR_2 = 0.0684
Final R indexes [all data]	R_1 = 0.0294, wR_2 = 0.0691
Largest diff. peak/hole / e Å^{-3}	0.20/-0.21
Flack parameter	0.046(12)

Crystal data and structure refinement for **4m**

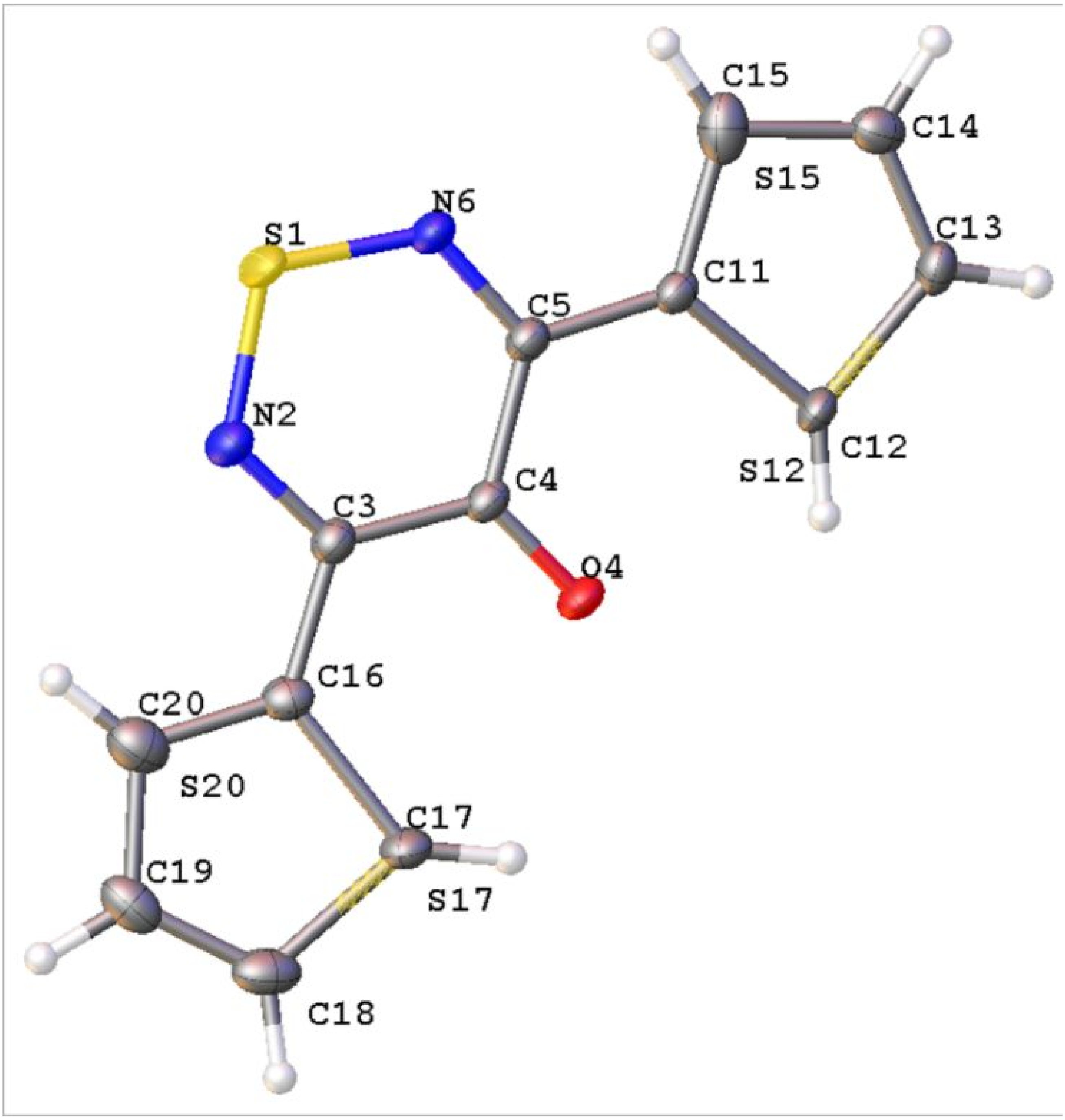

Perspective view of **4m** with displacement ellipsoids drawn at the 50% probability level

Identification code	4m
Empirical formula	$C_{11}H_6N_2OS_3$
Formula weight	278.36
Temperature/K	100.0
Crystal system	monoclinic
Space group	$P2_1/n$
a/Å	14.0787(8)
b/Å	4.9634(3)
c/Å	16.2584(10)
α/°	90
β/°	104.691(2)

γ/°	90
Volume/Å^3	1098.97(11)
Z	4
ρ_{calc}g/cm^3	1.682
μ/mm^{-1}	0.654
F(000)	568.0
Crystal size/mm^3	0.46 × 0.08 × 0.04
Radiation	MoKα (λ = 0.71073)
2Θ range for data collection/°	5.984 to 57.548
Index ranges	$-19 \leq h \leq 19$, $-6 \leq k \leq 6$, $-21 \leq l \leq 22$
Reflections collected	45729
Independent reflections	2855 [R_{int} = 0.0841, R_{sigma} = 0.0295]
Data/restraints/parameters	2855/0/156
Goodness-of-fit on F^2	1.067
Final R indexes [I>=2σ (I)]	R_1 = 0.0478, wR_2 = 0.1043
Final R indexes [all data]	R_1 = 0.0663, wR_2 = 0.1144
Largest diff. peak/hole / e Å^{-3}	0.41/-0.48

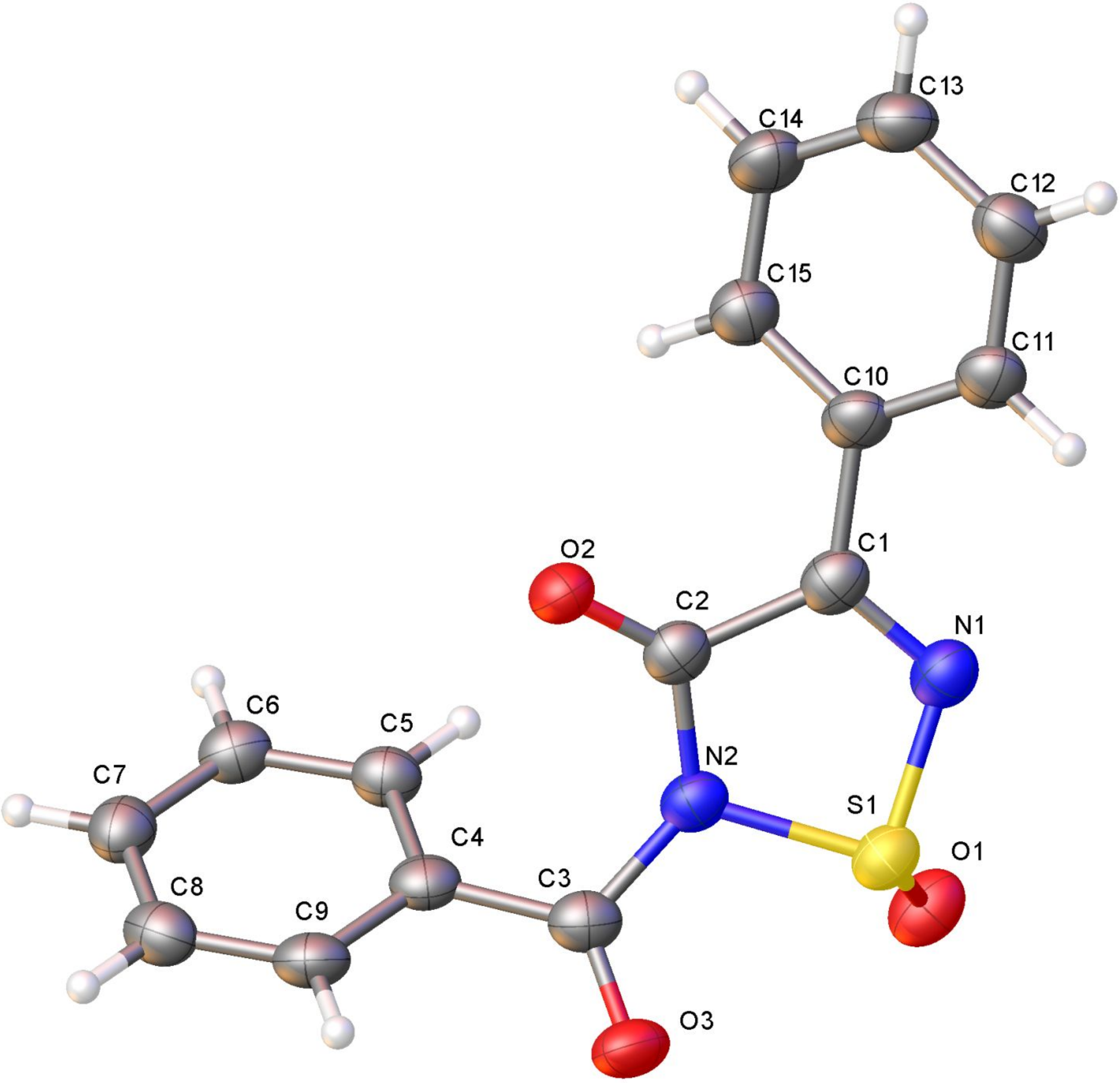

Perspective view of **5a** with displacement ellipsoids drawn at the 50% probability level

Crystal data and structure refinement for **5a**

Identification code	5a
Empirical formula	$C_{15}H_{10}N_2O_3S$
Formula weight	298.31
Temperature/K	100.0
Crystal system	monoclinic
Space group	$P2_1/c$
a/Å	9.7612(5)
b/Å	4.3248(2)
c/Å	31.9875(17)
α/°	90
β/°	93.392(3)
γ/°	90
Volume/Å^3	1347.99(12)
Z	4

ρ_{calc}g/cm^3	1.470
μ/mm^{-1}	2.250
F(000)	616.0
Crystal size/mm^3	0.22 × 0.06 × 0.03
Radiation	CuKα (λ = 1.54178)
2Θ range for data collection/°	5.536 to 149.262
Index ranges	$-12 \leq h \leq 12$, $-5 \leq k \leq 5$, $-40 \leq l \leq 40$
Reflections collected	35241
Independent reflections	2747 [R_{int} = 0.0790, R_{sigma} = 0.0303]
Data/restraints/parameters	2747/0/190
Goodness-of-fit on F^2	1.139
Final R indexes [I>=2σ (I)]	R_1 = 0.0670, wR_2 = 0.1749
Final R indexes [all data]	R_1 = 0.0746, wR_2 = 0.1788
Largest diff. peak/hole / e Å^{-3}	0.83/-0.40

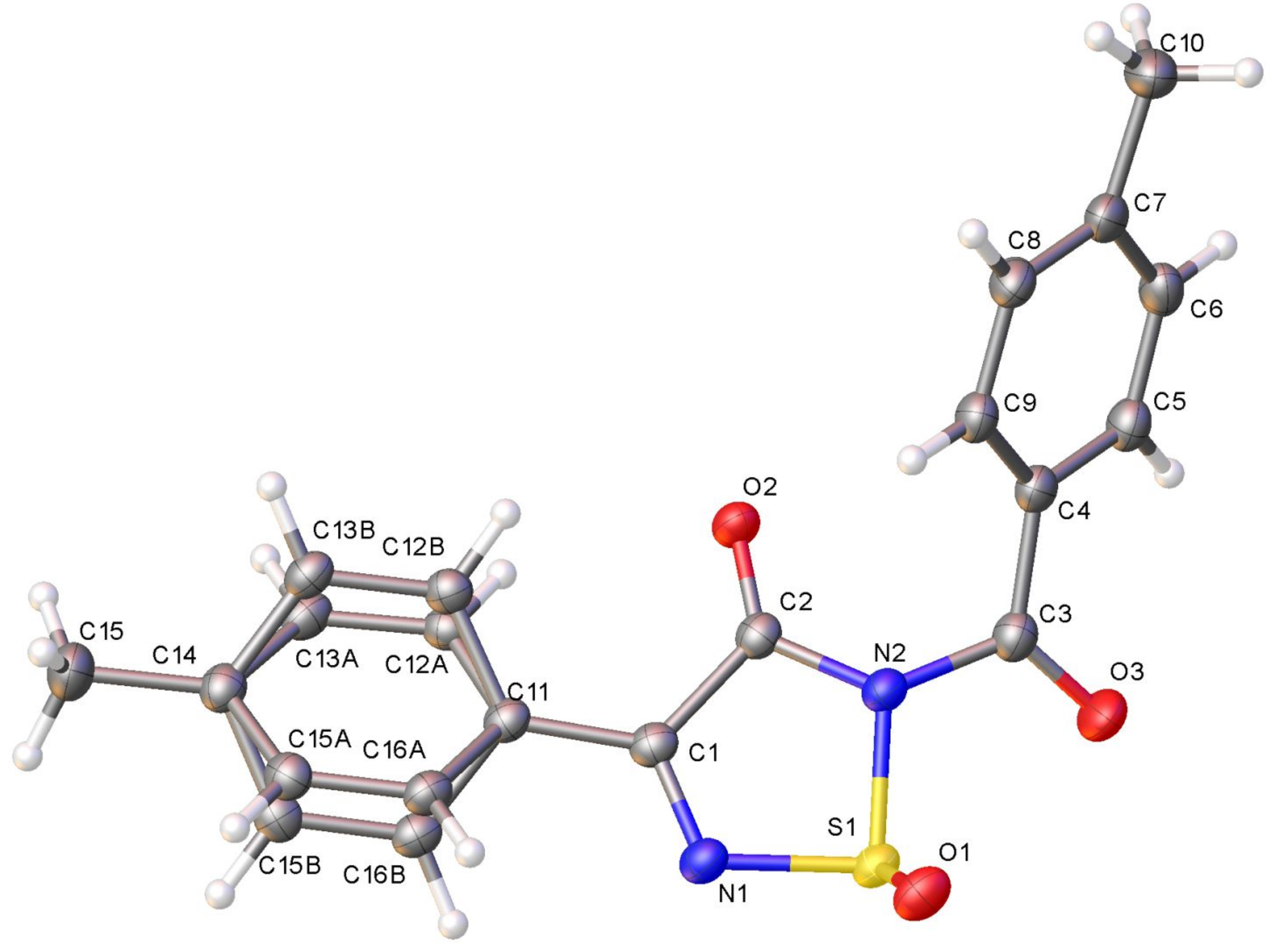

Perspective view of **5b** with displacement ellipsoids drawn at the 50% probability level. The phenyl rig is disordered over two positions

Crystal data and structure refinement for **5b**

Identification code	5b
Empirical formula	$C_{17}H_{14}N_2O_3S$
Formula weight	326.36
Temperature/K	100.0
Crystal system	monoclinic
Space group	C2/c
a/Å	18.1894(8)
b/Å	4.4604(2)
c/Å	37.7102(17)
α/°	90
β/°	91.048(2)
γ/°	90
Volume/Å^3	3059.0(2)
Z	8
ρ_{calc}g/cm^3	1.417
μ/mm^{-1}	2.031
F(000)	1360.0
Crystal size/mm^3	0.46 × 0.1 × 0.04
Radiation	CuKα (λ = 1.54178)
2Θ range for data collection/°	4.688 to 149.22

Index ranges	$-22 \le h \le 22$, $-5 \le k \le 5$, $-47 \le l \le 46$
Reflections collected	33296
Independent reflections	3139 [R_{int} = 0.0617, R_{sigma} = 0.0297]
Data/restraints/parameters	3139/0/246
Goodness-of-fit on F^2	1.037
Final R indexes [I>=2σ (I)]	R_1 = 0.0389, wR_2 = 0.1020
Final R indexes [all data]	R_1 = 0.0435, wR_2 = 0.1082
Largest diff. peak/hole / e Å^{-3}	0.36/-0.40

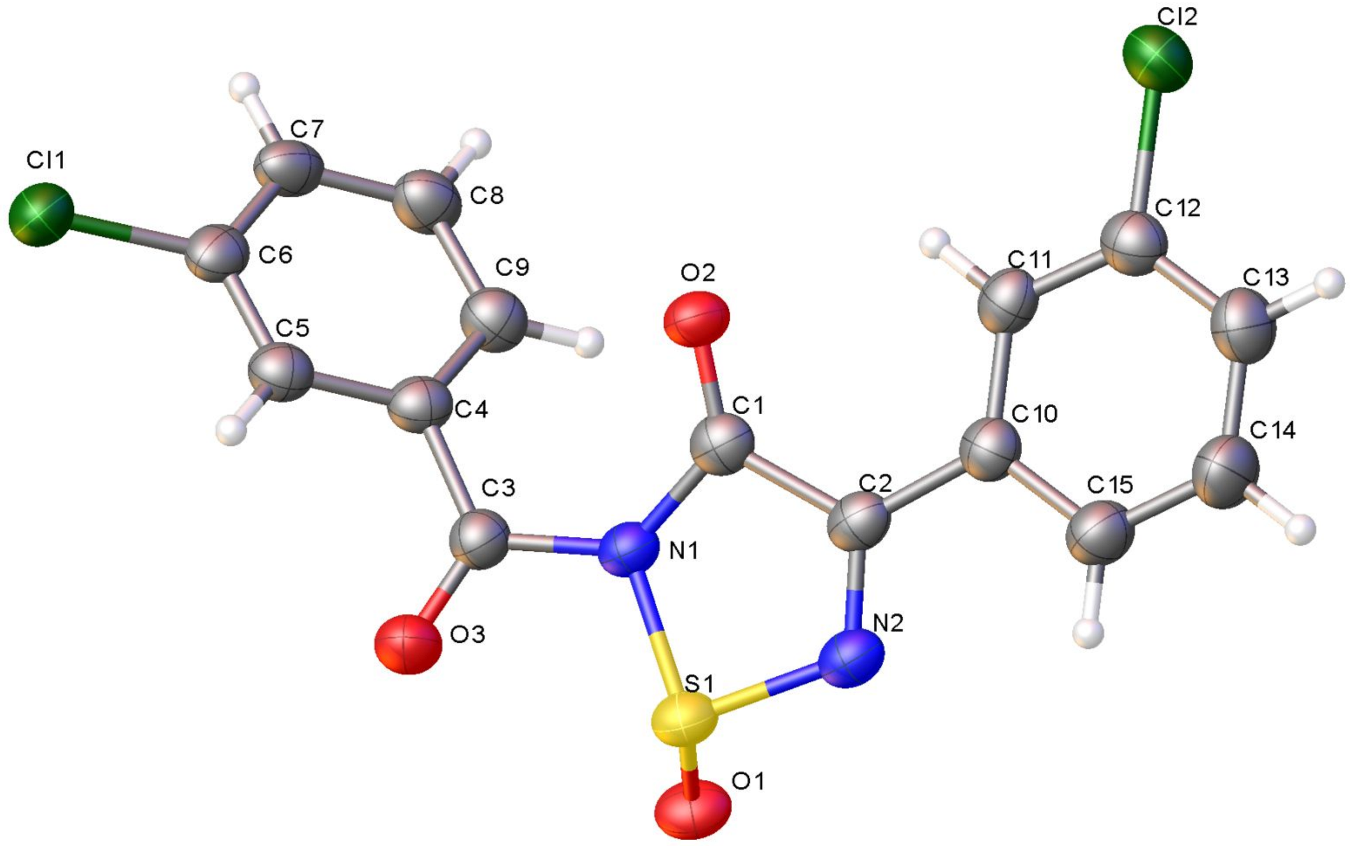

Perspective view of **5i** with displacement ellipsoids drawn at the 50% probability level

Crystal data and structure refinement for **5i**

Identification code	5i
Empirical formula	$C_{15}H_8Cl_2N_2O_3S$
Formula weight	367.19
Temperature/K	100.0
Crystal system	monoclinic
Space group	$P2_1/c$
a/Å	4.228(2)
b/Å	18.934(10)
c/Å	18.988(10)
α/°	90
β/°	95.597(18)
γ/°	90
Volume/Å^3	1512.8(14)
Z	4
ρ_{calc}g/cm^3	1.612
μ/mm^{-1}	5.305
F(000)	744.0
Crystal size/mm^3	0.26 × 0.04 × 0.04
Radiation	CuKα (λ = 1.54178)
2Θ range for data collection/°	6.608 to 117.892
Index ranges	$-4 \le h \le 4$, $-20 \le k \le 20$, $-20 \le l \le 20$
Reflections collected	16161
Independent reflections	2162 [R_{int} = 0.0662, R_{sigma} = 0.0339]
Data/restraints/parameters	2162/0/208

Goodness-of-fit on F^2	1.070
Final R indexes [I>=2σ (I)]	R_1 = 0.0488, wR_2 = 0.1293
Final R indexes [all data]	R_1 = 0.0547, wR_2 = 0.1341
Largest diff. peak/hole / e Å^{-3}	0.35/-0.54

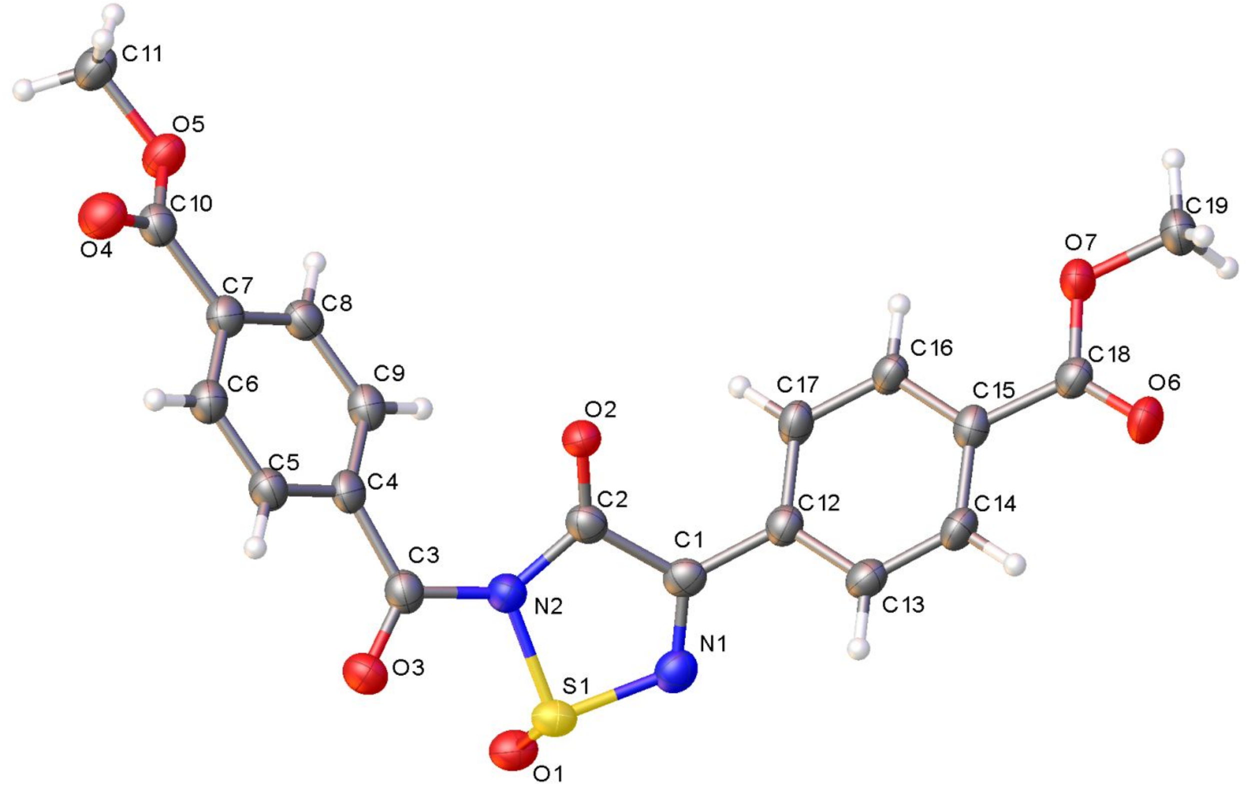

Perspective view of **5l** with displacement ellipsoids drawn at the 50% probability level

Crystal data and structure refinement for **5l**	
Identification code	5l
Empirical formula	$C_{19}H_{14}N_2O_7S$
Formula weight	414.38
Temperature/K	100.0
Crystal system	monoclinic
Space group	Cc
a/Å	8.9745(3)
b/Å	32.8200(11)
c/Å	6.9065(2)
α/°	90
β/°	119.452(2)
γ/°	90
Volume/Å^3	1771.37(10)
Z	4
ρ_{calc}g/cm^3	1.554
μ/mm^{-1}	2.069
F(000)	856.0
Crystal size/mm^3	0.22 × 0.18 × 0.04
Radiation	CuKα (λ = 1.54178)
2Θ range for data collection/°	5.386 to 149.056
Index ranges	$-11 \leq h \leq 11$, $-40 \leq k \leq 40$, $-8 \leq l \leq 8$
Reflections collected	22913
Independent reflections	3591 [R_{int} = 0.0625, R_{sigma} = 0.0388]
Data/restraints/parameters	3591/2/265
Goodness-of-fit on F^2	1.049
Final R indexes [I>=2σ (I)]	R_1 = 0.0404, wR_2 = 0.0999

Final R indexes [all data]	R_1 = 0.0437, wR_2 = 0.1031
Largest diff. peak/hole / e Å^{-3}	0.36/-0.25
Flack parameter	0.11(3)

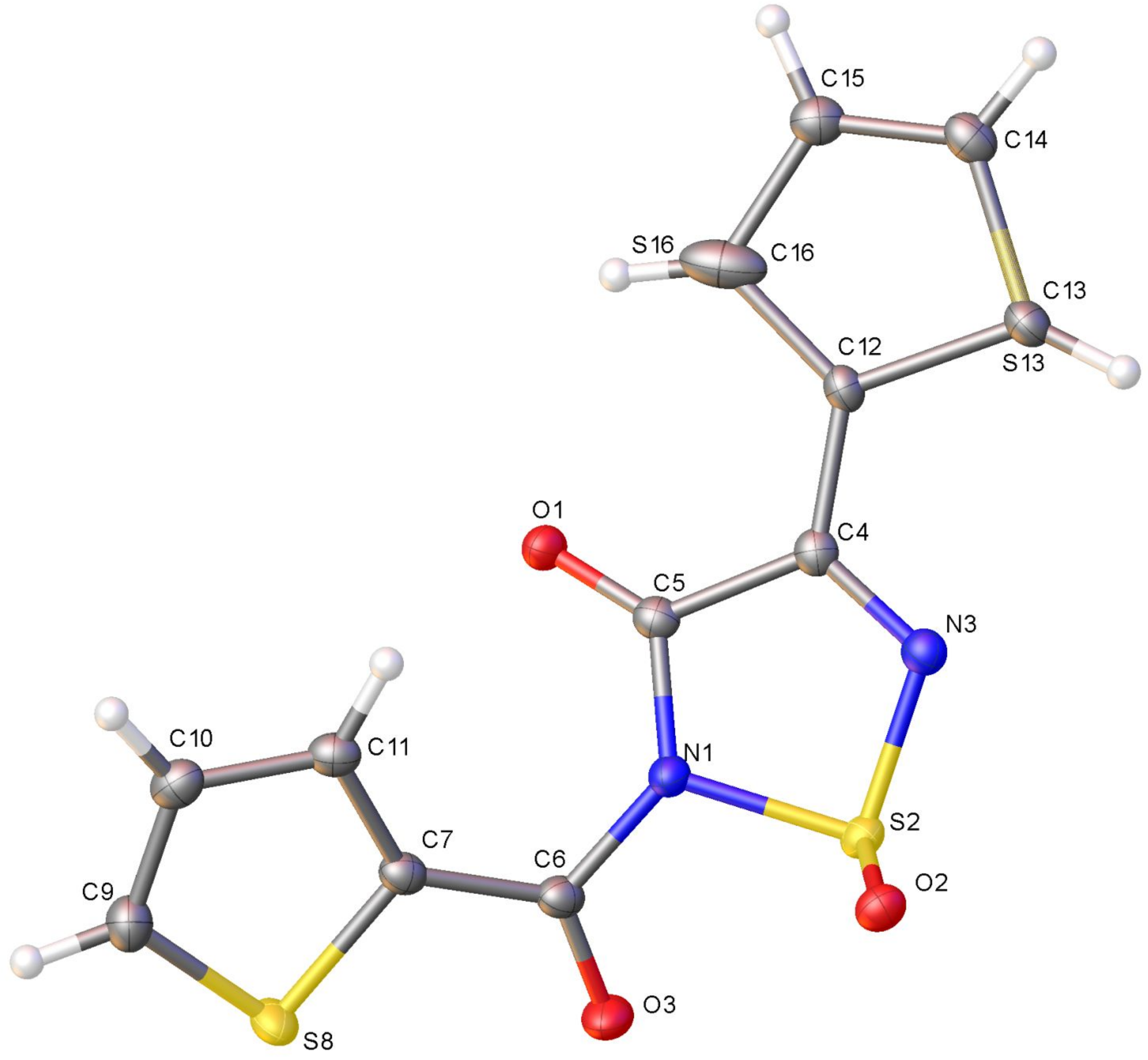

Perspective view of **5m** with displacement ellipsoids drawn at the 50% probability level

Crystal data and structure refinement for **5m**

Identification code	5m
Empirical formula	$C_{11}H_6N_2O_3S_3$
Formula weight	310.36
Temperature/K	100.0
Crystal system	monoclinic
Space group	$P2_1/c$
a/Å	6.79830(10)
b/Å	19.5591(4)
c/Å	8.9836(2)
α/°	90
β/°	95.2280(10)
γ/°	90
Volume/Å^3	1189.57(4)
Z	4
ρ_{calc}g/cm^3	1.733
μ/mm^{-1}	5.770

F(000)	632.0
Crystal size/mm^3	0.24 × 0.04 × 0.02
Radiation	CuKα (λ = 1.54178)
2Θ range for data collection/°	9.042 to 148.836
Index ranges	$-8 \leq h \leq 8$, $-24 \leq k \leq 24$, $-11 \leq l \leq 11$
Reflections collected	43523
Independent reflections	2437 [R_{int} = 0.0422, R_{sigma} = 0.0154]
Data/restraints/parameters	2437/0/173
Goodness-of-fit on F^2	1.080
Final R indexes [I>=2σ (I)]	R_1 = 0.0259, wR_2 = 0.0691
Final R indexes [all data]	R_1 = 0.0272, wR_2 = 0.0700
Largest diff. peak/hole / e Å^{-3}	0.52/-0.33

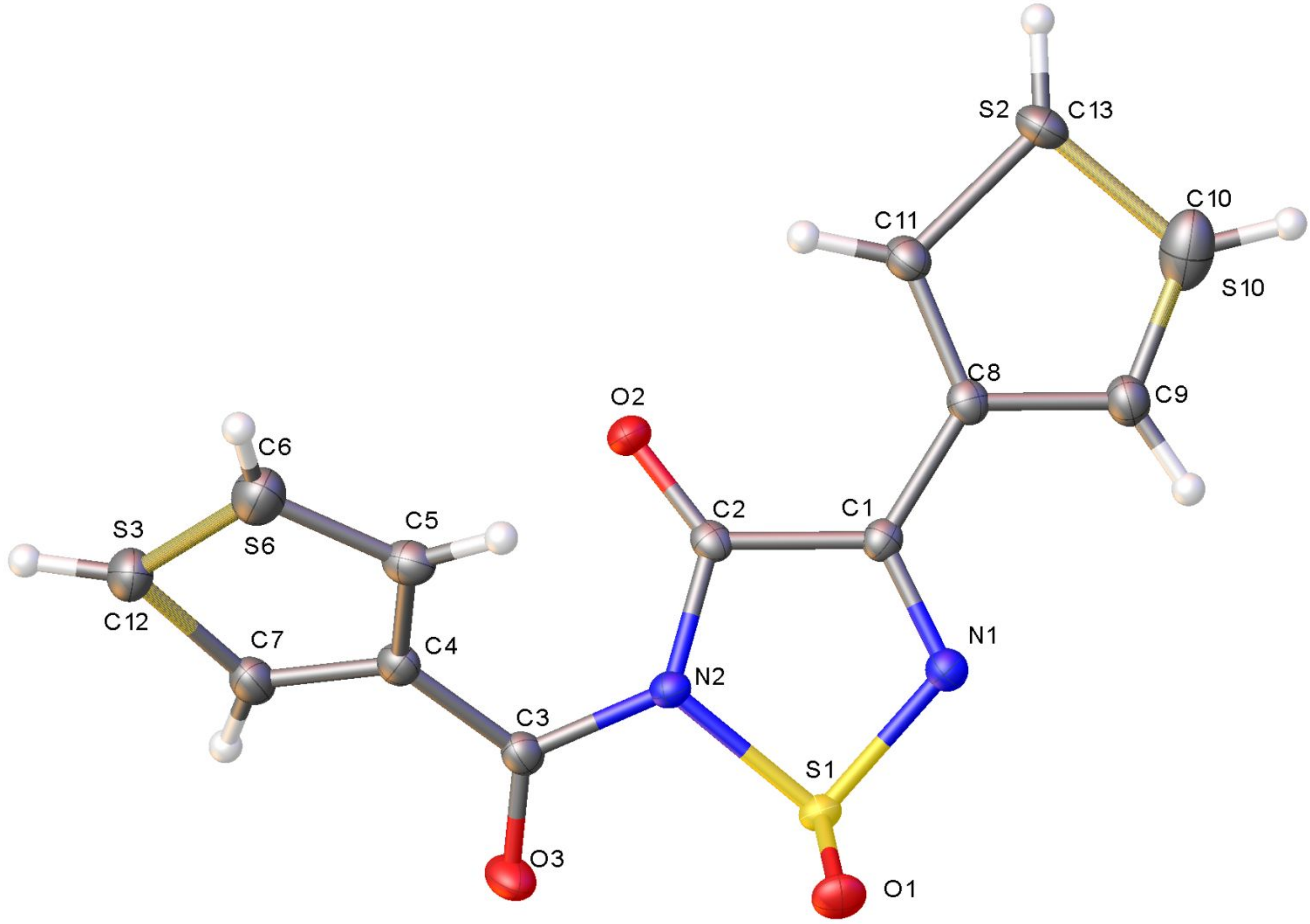

Perspective view of **5n** with displacement ellipsoids drawn at the 50% probability level showing substitutional carbon sulfur disorder at S3/C12, S6/C6 S2/C13 C10/S10.

Crystal data and structure refinement for **5n**	
Identification code	5n
Empirical formula	$C_{11}H_6N_2O_3S_3$
Formula weight	310.36
Temperature/K	100.0
Crystal system	orthorhombic
Space group	$Pna2_1$
a/Å	16.2994(7)
b/Å	17.9235(8)
c/Å	4.3228(2)
α/°	90
β/°	90
γ/°	90
Volume/Å^3	1262.87(10)
Z	4
ρ_{calc}g/cm^3	1.632
μ/mm^{-1}	5.435
F(000)	632.0
Crystal size/mm^3	0.44 × 0.1 × 0.04
Radiation	CuKα (λ = 1.54178)
2Θ range for data collection/°	7.33 to 144.636
Index ranges	$-20 \le h \le 20$, $-21 \le k \le 22$, $-5 \le l \le 5$

Reflections collected	14359
Independent reflections	2438 [R_{int} = 0.0369, R_{sigma} = 0.0263]
Data/restraints/parameters	2438/1/174
Goodness-of-fit on F^2	1.053
Final R indexes [I>=2σ (I)]	R_1 = 0.0239, wR_2 = 0.0628
Final R indexes [all data]	R_1 = 0.0242, wR_2 = 0.0631
Largest diff. peak/hole / e $Å^{-3}$	0.23/-0.23
Flack parameter	-0.005(11)

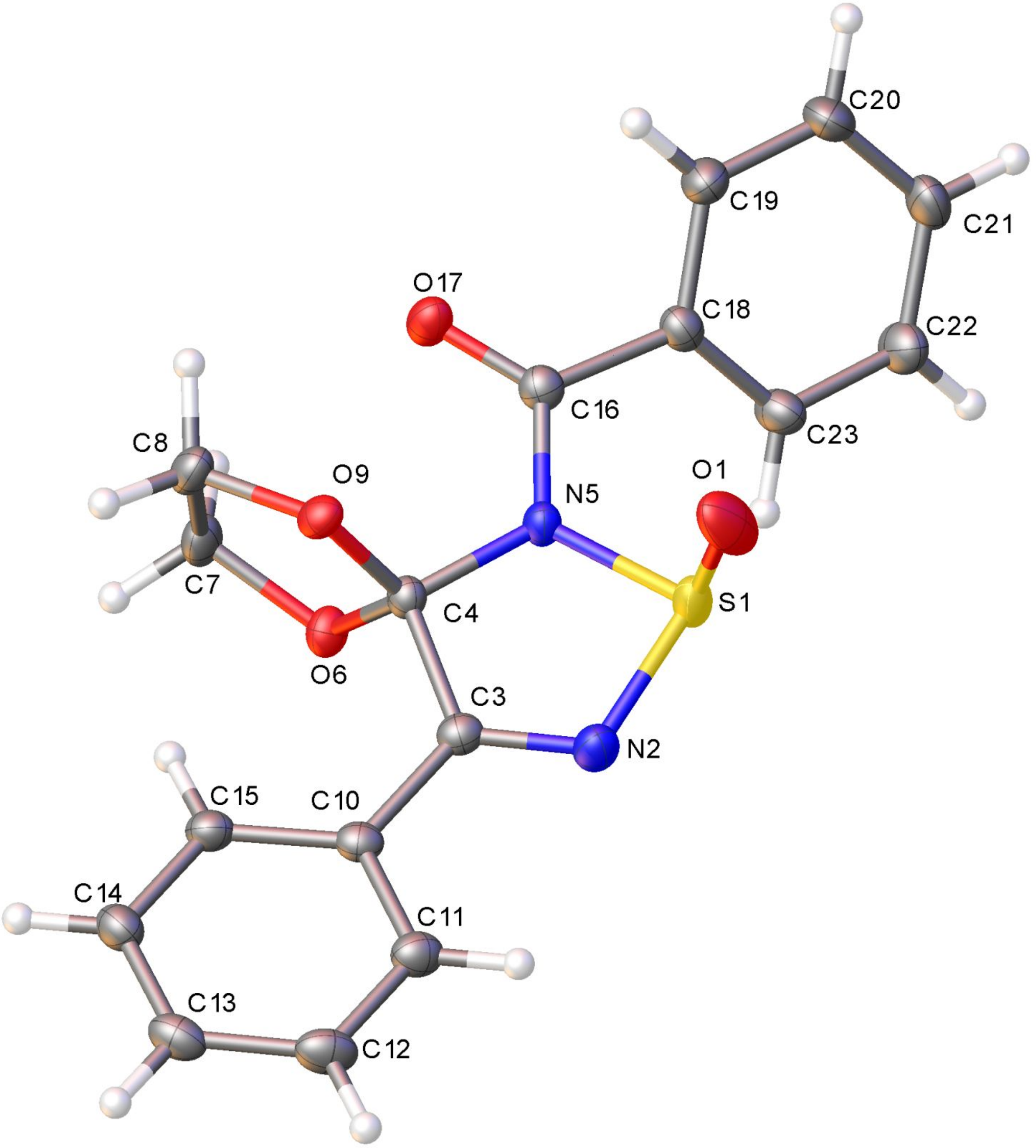

Perspective view of **5u** with displacement ellipsoids drawn at the 50% probability level

Crystal data and structure refinement for **5u**

Identification code	5u
Empirical formula	$C_{17}H_{14}N_2O_4S$
Formula weight	342.36
Temperature/K	100.0
Crystal system	orthorhombic
Space group	$P2_12_12_1$
a/Å	8.4196(7)

b/Å	13.2151(12)
c/Å	14.0737(10)
α/°	90
β/°	90
γ/°	90
Volume/Å^3	1565.9(2)
Z	4
ρ_{calc}g/cm^3	1.452
μ/mm^{-1}	2.061
F(000)	712.0
Crystal size/mm^3	0.2 × 0.2 × 0.1
Radiation	CuKα (λ = 1.54178)
2Θ range for data collection/°	12.464 to 159.958
Index ranges	-10 ≤ h ≤ 10, -16 ≤ k ≤ 16, -17 ≤ l ≤ 17
Reflections collected	53271
Independent reflections	3362 [R_{int} = 0.0259, R_{sigma} = 0.0103]
Data/restraints/parameters	3362/0/218
Goodness-of-fit on F^2	1.034
Final R indexes [I>=2σ (I)]	R_1 = 0.0226, wR_2 = 0.0616
Final R indexes [all data]	R_1 = 0.0226, wR_2 = 0.0616
Largest diff. peak/hole / e Å^{-3}	0.23/-0.24
Flack parameter	0.483(14)

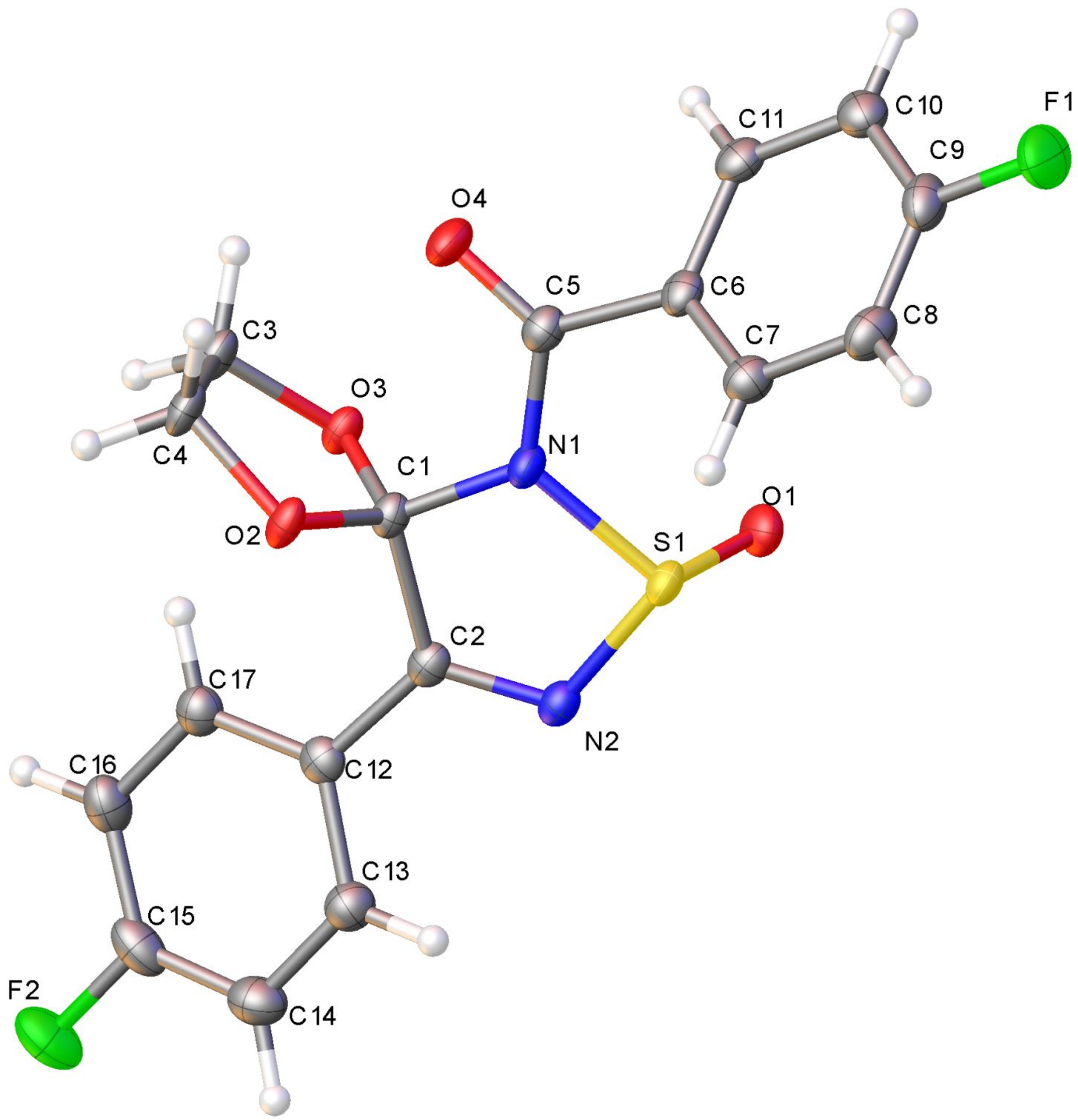

Perspective view of **5x** with displacement ellipsoids drawn at the 50% probability level

Crystal data and structure refinement for **5x**

Identification code	5x
Empirical formula	$C_{17}H_{12}F_2N_2O_4S$
Formula weight	378.35
Temperature/K	100.0
Crystal system	monoclinic
Space group	$P2_1/c$
a/Å	13.153(3)
b/Å	15.142(4)
c/Å	8.3150(15)

α/°	90
β/°	104.933(6)
γ/°	90
Volume/Å^3	1600.1(6)
Z	4
ρ_{calc}g/cm^3	1.571
μ/mm^{-1}	2.266
F(000)	776.0
Crystal size/mm^3	0.42 × 0.18 × 0.08
Radiation	CuKα (λ = 1.54178)
2Θ range for data collection/°	6.956 to 144.32
Index ranges	$-16 \leq h \leq 16$, $-18 \leq k \leq 16$, $-9 \leq l \leq 10$
Reflections collected	21101
Independent reflections	3156 [R_{int} = 0.0536, R_{sigma} = 0.0319]
Data/restraints/parameters	3156/0/235
Goodness-of-fit on F^2	1.070
Final R indexes [I>=2σ (I)]	R_1 = 0.0321, wR_2 = 0.0873
Final R indexes [all data]	R_1 = 0.0353, wR_2 = 0.0894
Largest diff. peak/hole / e Å^{-3}	0.32/-0.33

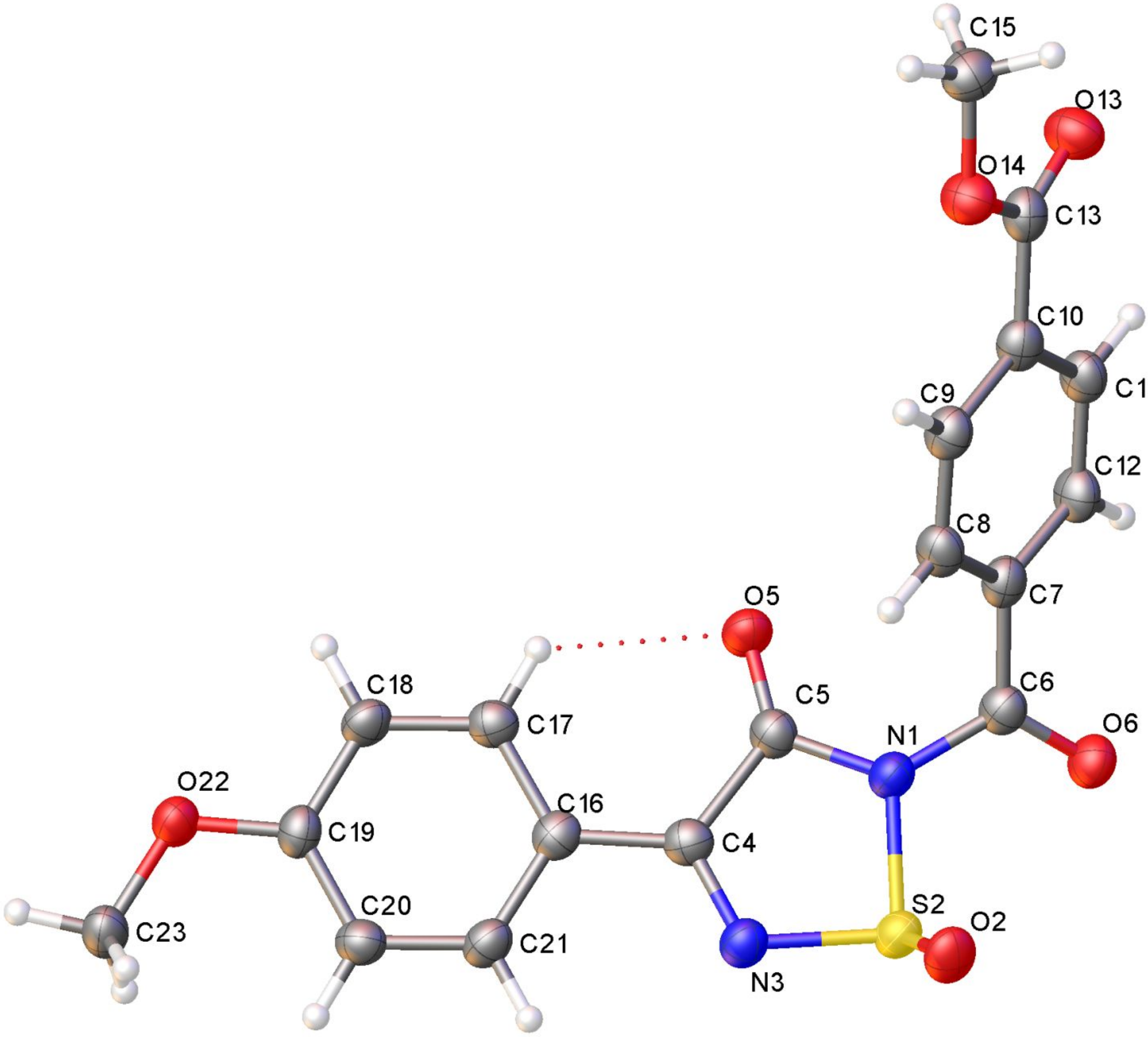

Perspective view of **5aa** with displacement ellipsoids drawn at the 50% probability level

Crystal data and structure refinement for **5aa**

Identification code	5aa
Empirical formula	$C_{18}H_{14}N_2O_6S$
Formula weight	386.37
Temperature/K	100.0
Crystal system	monoclinic
Space group	$P2_1/n$
a/Å	15.333(7)
b/Å	5.838(2)
c/Å	18.736(8)
α/°	90
β/°	91.048(18)
γ/°	90
Volume/Å^3	1676.8(12)
Z	4
ρ_{calc}g/cm^3	1.531
μ/mm^{-1}	2.091

F(000)	800.0
Crystal size/mm^3	0.36 × 0.04 × 0.02
Radiation	CuKα (λ = 1.54178)
2Θ range for data collection/°	7.384 to 150.274
Index ranges	$-13 \le h \le 19$, $-7 \le k \le 7$, $-23 \le l \le 23$
Reflections collected	21525
Independent reflections	3427 [R_{int} = 0.0983, R_{sigma} = 0.0616]
Data/restraints/parameters	3427/0/246
Goodness-of-fit on F^2	1.023
Final R indexes [I>=2σ (I)]	R_1 = 0.0625, wR_2 = 0.1625
Final R indexes [all data]	R_1 = 0.0799, wR_2 = 0.1773
Largest diff. peak/hole / e Å^{-3}	0.38/-0.60

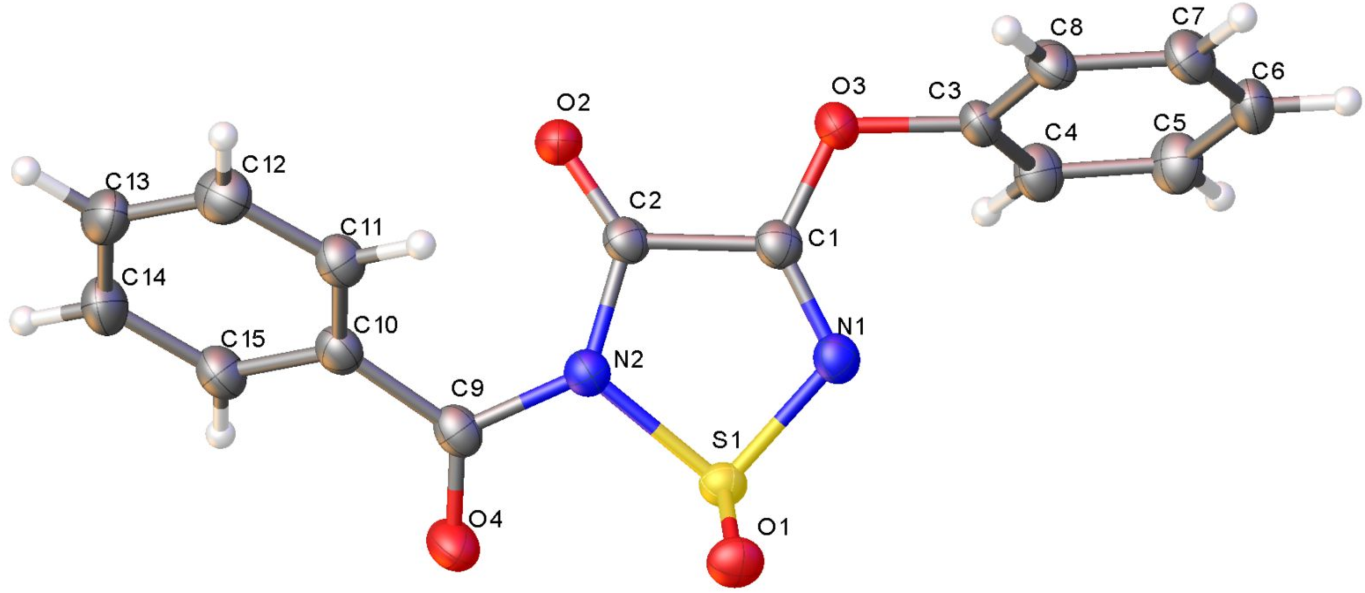

Perspective view of **5ac** with displacement ellipsoids drawn at the 50% probability level

Crystal data and structure refinement for **5ac**

Identification code	5ac
Empirical formula	$C_{15}H_{10}N_2O_4S$
Formula weight	314.31
Temperature/K	100.0
Crystal system	orthorhombic
Space group	$P2_12_12_1$
a/Å	4.34330(10)
b/Å	17.9288(6)
c/Å	18.4384(6)
α/°	90
β/°	90
γ/°	90
Volume/Å^3	1435.80(7)
Z	4
ρ_{calc}g/cm^3	1.454
μ/mm^{-1}	2.197
F(000)	648.0
Crystal size/mm^3	0.22 × 0.08 × 0.04
Radiation	CuKα (λ = 1.54178)
2Θ range for data collection/°	6.876 to 149.246
Index ranges	$-5 \le h \le 5$, $-21 \le k \le 22$, $-22 \le l \le 23$
Reflections collected	17077
Independent reflections	2932 [R_{int} = 0.0585, R_{sigma} = 0.0358]
Data/restraints/parameters	2932/0/200
Goodness-of-fit on F^2	1.058
Final R indexes [I>=2σ (I)]	R_1 = 0.0332, wR_2 = 0.0807
Final R indexes [all data]	R_1 = 0.0364, wR_2 = 0.0827
Largest diff. peak/hole / e Å^{-3}	0.28/-0.18
Flack parameter	0.44(2)

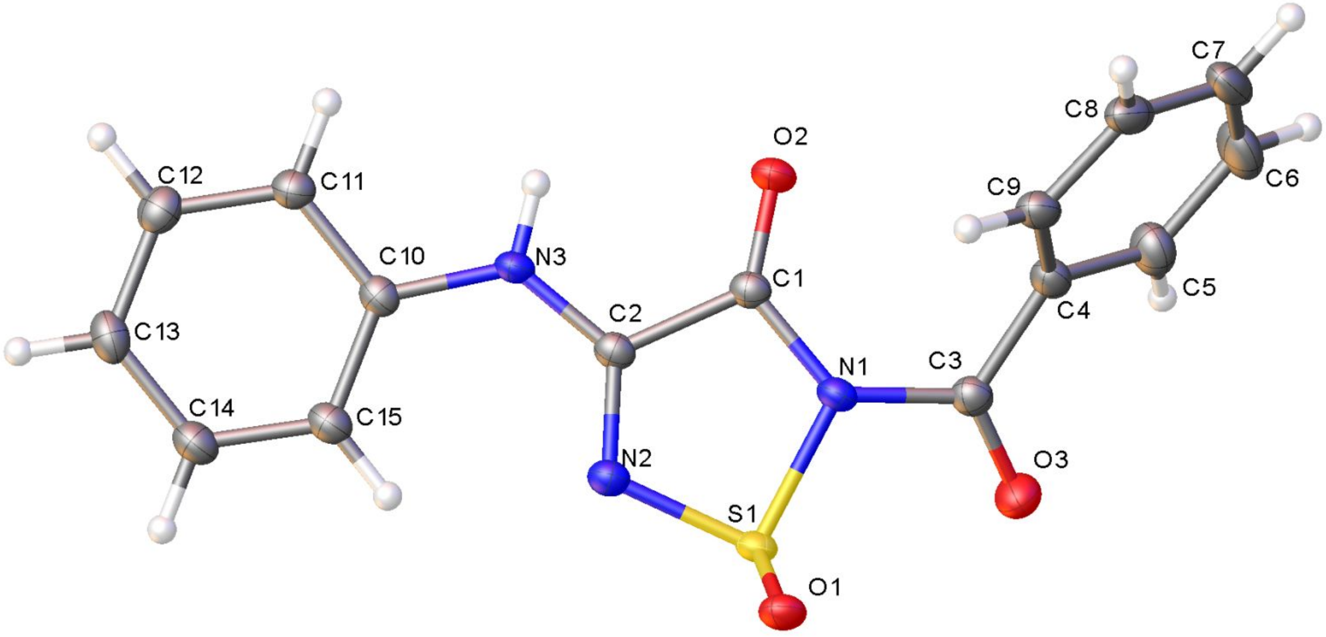

Perspective view of **5af** with displacement ellipsoids drawn at the 50% probability level

Crystal data and structure refinement for **5af**	
Identification code	5af
Empirical formula	$C_{15}H_{11}N_3O_3S$
Formula weight	313.33
Temperature/K	100.0
Crystal system	Monoclinic
Space group	$P2_1/c$
a/Å	14.7665(5)
b/Å	13.0019(5)
c/Å	7.1500(3)
α/°	90
β/°	90.591(2)
γ/°	90
Volume/Å^3	1372.67(9)
Z	4
ρ_{calc}g/cm^3	1.516
μ/mm^{-1}	2.260
F(000)	648.0
Crystal size/mm^3	0.42 × 0.28 × 0.04
Radiation	CuKα (λ = 1.54178)
2Θ range for data collection/°	5.986 to 140.424
Index ranges	$-18 \leq h \leq 16$, $-15 \leq k \leq 15$, $-8 \leq l \leq 8$
Reflections collected	17655
Independent reflections	2613 [R_{int} = 0.1543, R_{sigma} = 0.0821]
Data/restraints/parameters	2613/0/199
Goodness-of-fit on F^2	1.054
Final R indexes [I>=2σ (I)]	R_1 = 0.0411, wR_2 = 0.1053
Final R indexes [all data]	R_1 = 0.0658, wR_2 = 0.1129
Largest diff. peak/hole / e Å^{-3}	0.49/-0.38

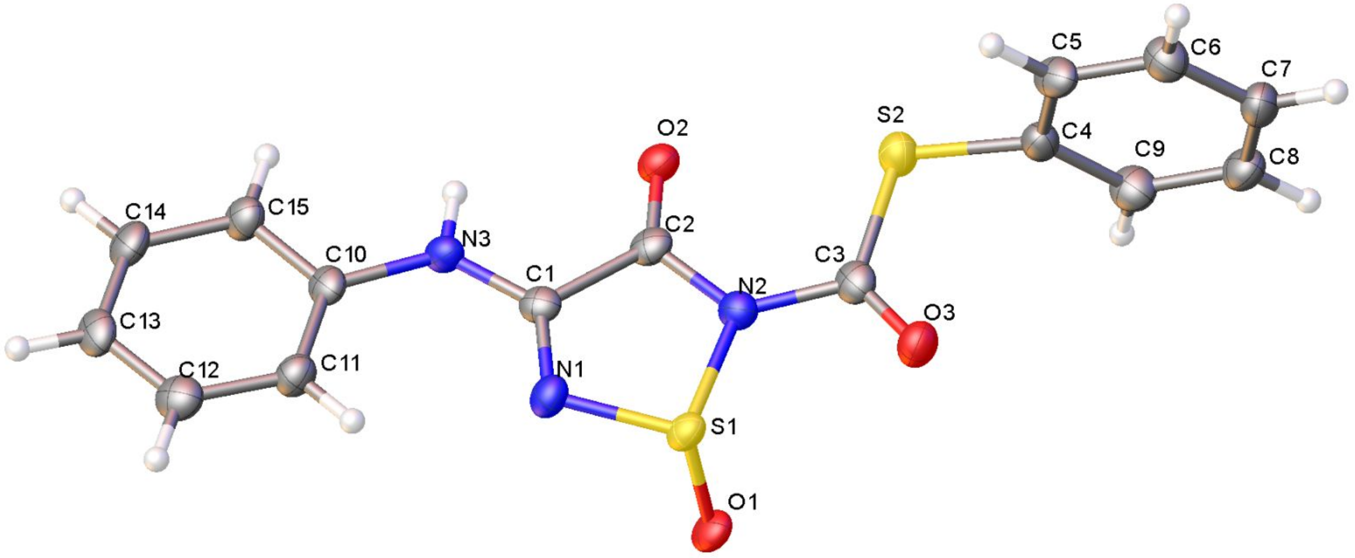

Perspective view of **5ah** with displacement ellipsoids drawn at the 50% probability level

Crystal data and structure refinement for **5ah**

Identification code	5ah
Empirical formula	$C_{15}H_{11}N_3O_3S_2$
Formula weight	345.39
Temperature/K	100.0
Crystal system	triclinic
Space group	P-1
a/Å	6.9681(4)
b/Å	7.5359(4)
c/Å	15.4390(9)
α/°	79.625(4)
β/°	88.507(4)
γ/°	67.320(4)
Volume/Å^3	734.96(7)
Z	2
ρ_{calc}g/cm^3	1.561
μ/mm^{-1}	3.463
F(000)	356.0
Crystal size/mm^3	0.12 × 0.04 × 0.02
Radiation	CuKα (λ = 1.54178)
2Θ range for data collection/°	5.826 to 127.45
Index ranges	$-8 \le h \le 8$, $-8 \le k \le 8$, $0 \le l \le 17$
Reflections collected	21852
Independent reflections	2350 [R_{int} = 0.1070, R_{sigma} = 0.0645]
Data/restraints/parameters	2350/0/209
Goodness-of-fit on F^2	1.110
Final R indexes [I>=2σ (I)]	R_1 = 0.0715, wR_2 = 0.1827
Final R indexes [all data]	R_1 = 0.0861, wR_2 = 0.1942
Largest diff. peak/hole / e Å^{-3}	0.45/-0.49

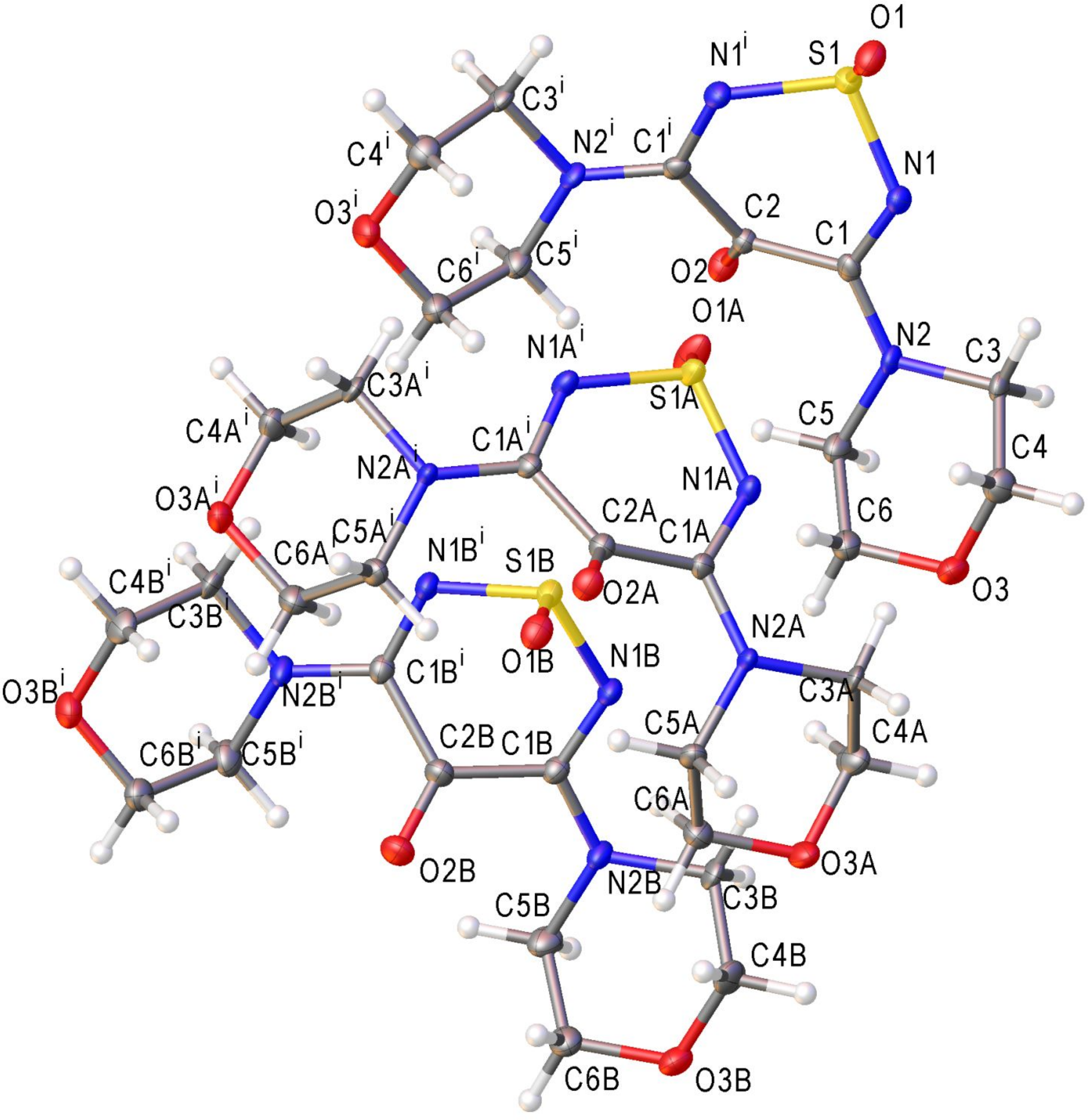

Perspective view of **17a** with displacement ellipsoids drawn at the 50% probability level.The superscript i denotes the symmetry operation $1 +X,1-Y,+Z

Crystal data and structure refinement **17a**

Identification code	17a
Empirical formula	$C_{11}H_{16}N_4O_4S$
Formula weight	300.34
Temperature/K	100.0
Crystal system	monoclinic
Space group	Cm
a/Å	7.98980(10)
b/Å	21.7995(3)
c/Å	11.6915(2)
α/°	90

β/°	109.9253(8)
γ/°	90
Volume/Å3	1914.45(5)
Z	6
ρcalcg/cm3	1.563
μ/mm-1	2.469
F(000)	948.0
Crystal size/mm3	0.2 × 0.14 × 0.03
Radiation	CuKα (λ = 1.54178)
2Θ range for data collection/°	8.044 to 144.458
Index ranges	-9 ≤ h ≤ 9, -26 ≤ k ≤ 26, -14 ≤ l ≤ 14
Reflections collected	24106
Independent reflections	3705 [Rint = 0.0495, Rsigma = 0.0301]
Data/restraints/parameters	3705/2/290
Goodness-of-fit on F2	1.069
Final R indexes [I>=2σ (I)]	R1 = 0.0373, wR2 = 0.0990
Final R indexes [all data]	R1 = 0.0380, wR2 = 0.1001
Largest diff. peak/hole / e $Å^{-3}$	0.64/-0.23
Flack parameter	-0.059(15)

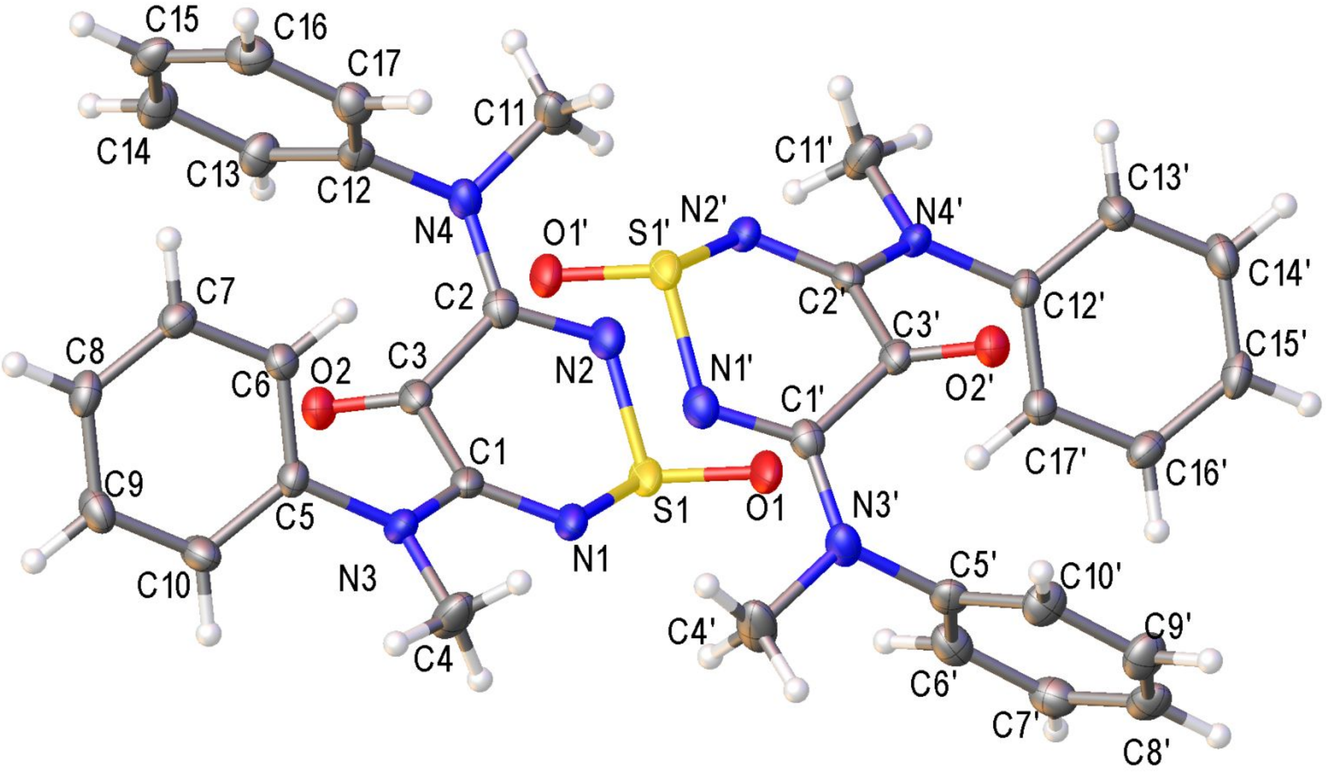

Perspective view of **17b** with displacement ellipsoids drawn at the 50% probability level. There are two molecules in the asymmetric unit.

Crystal data and structure refinement for **17b**	
Identification code	17b
Empirical formula	$C_{17}H_{16}N_4O_2S$
Formula weight	340.40
Temperature/K	100.0
Crystal system	triclinic
Space group	P-1
a/Å	10.7889(3)
b/Å	11.1624(3)
c/Å	14.1533(3)
α/°	81.1030(10)
β/°	78.8280(10)
γ/°	79.6240(10)
Volume/Å^3	1632.12(7)
Z	4
ρ_{calc}g/cm^3	1.385
μ/mm^{-1}	1.914
F(000)	712.0
Crystal size/mm^3	0.55 × 0.4 × 0.25
Radiation	CuKα (λ = 1.54178)
2Θ range for data collection/°	6.414 to 149.53
Index ranges	$-13 \le h \le 13$, $-13 \le k \le 13$, $-17 \le l \le 17$
Reflections collected	36321
Independent reflections	6612 [R_{int} = 0.0413, R_{sigma} = 0.0287]

Data/restraints/parameters	6612/0/438
Goodness-of-fit on F^2	1.133
Final R indexes [I>=2σ (I)]	R_1 = 0.0504, wR_2 = 0.1317
Final R indexes [all data]	R_1 = 0.0513, wR_2 = 0.1322
Largest diff. peak/hole / e Å^{-3}	1.16/-0.55

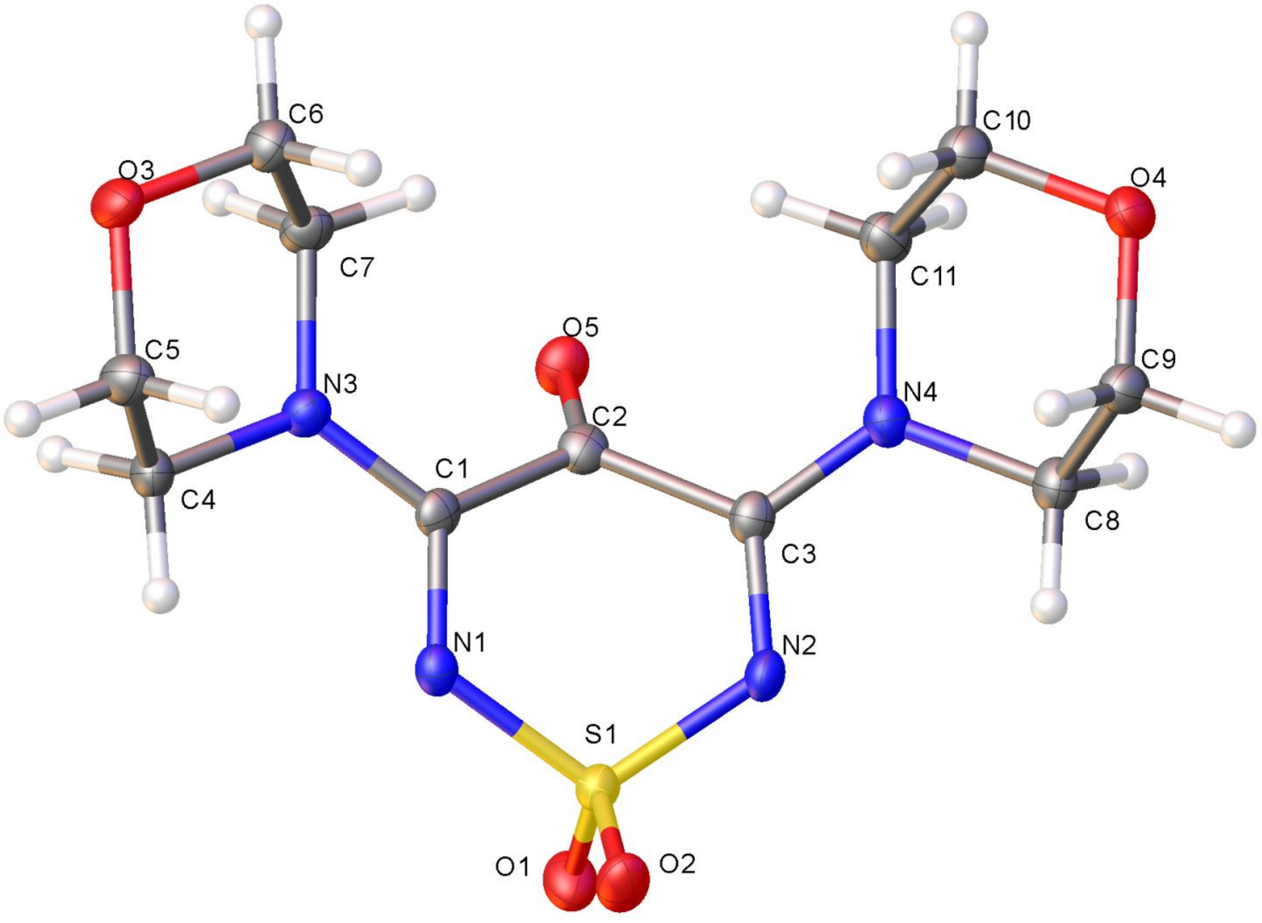

Perspective view of**18a** with displacement ellipsoids drawn at the 50% probability level.

Crystal data and structure refinement for **18a**

Identification code	18a
Empirical formula	$C_{11}H_{16}N_4O_5S$
Formula weight	316.34
Temperature/K	103.0
Crystal system	monoclinic
Space group	$P2_1/n$
a/Å	9.2026(18)
b/Å	7.6788(16)
c/Å	19.314(5)
α/°	90
β/°	100.157(9)
γ/°	90

Volume/Å^3	1343.4(5)
Z	4
ρ_{calc}g/cm^3	1.564
μ/mm^{-1}	2.436
F(000)	664.0
Crystal size/mm^3	0.36 × 0.03 × 0.02
Radiation	CuKα (λ = 1.54178)
2Θ range for data collection/°	9.304 to 137.09
Index ranges	$-11 \leq h \leq 11$, $-9 \leq k \leq 9$, $-22 \leq l \leq 23$
Reflections collected	20332
Independent reflections	2472 [R_{int} = 0.0773, R_{sigma} = 0.0372]
Data/restraints/parameters	2472/0/190
Goodness-of-fit on F^2	1.054
Final R indexes [I>=2σ (I)]	R_1 = 0.0477, wR_2 = 0.1227
Final R indexes [all data]	R_1 = 0.0630, wR_2 = 0.1329
Largest diff. peak/hole / e Å^{-3}	0.83/-0.57

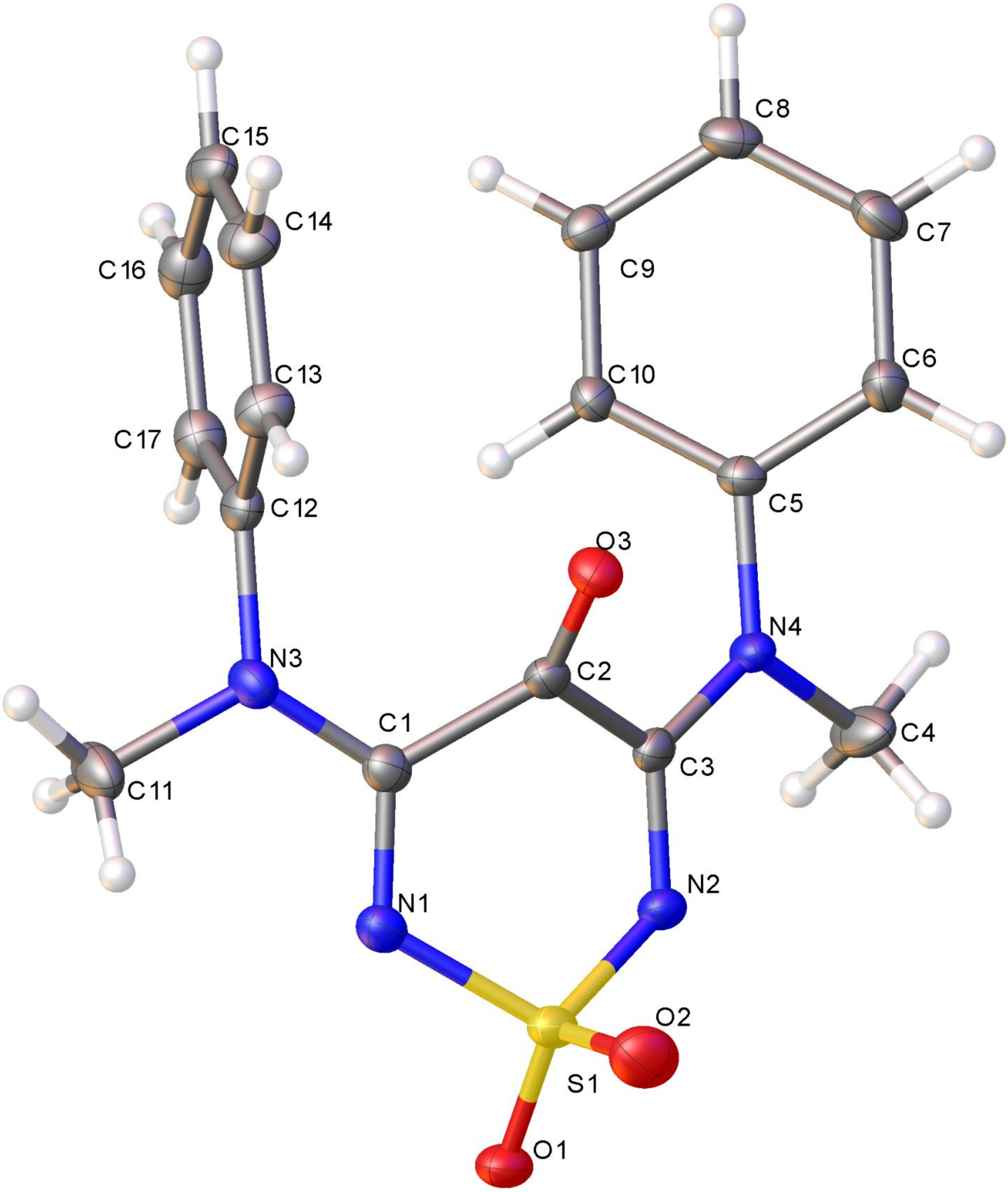

Perspective view of **18b** with displacement ellipsoids drawn at the 50% probability level.

Crystal data and structure refinement for **18b**	
Identification code	18b
Empirical formula	$C_{17}H_{16}N_4O_{2.75}S$
Formula weight	352.40
Temperature/K	100
Crystal system	triclinic
Space group	P-1
a/Å	9.7663(3)

b/Å	9.9019(3)
c/Å	10.1948(3)
α/°	88.9643(11)
β/°	65.4274(11)
γ/°	68.8774(10)
Volume/Å^3	826.21(4)
Z	2
ρ_{calc}g/cm^3	1.417
μ/mm^{-1}	0.219
F(000)	368.0
Crystal size/mm^3	0.24 × 0.2 × 0.12
Radiation	MoKα (λ = 0.71073)
2Θ range for data collection/°	5.012 to 56.574
Index ranges	-13 ≤ h ≤ 13, -13 ≤ k ≤ 13, -13 ≤ l ≤ 13
Reflections collected	25337
Independent reflections	4080 [R_{int} = 0.0382, R_{sigma} = 0.0244]
Data/restraints/parameters	4080/0/228
Goodness-of-fit on F^2	1.038
Final R indexes [I>=2σ (I)]	R_1 = 0.0378, wR_2 = 0.0895
Final R indexes [all data]	R_1 = 0.0475, wR_2 = 0.0951
Largest diff. peak/hole / e Å^{-3}	0.33/-0.49

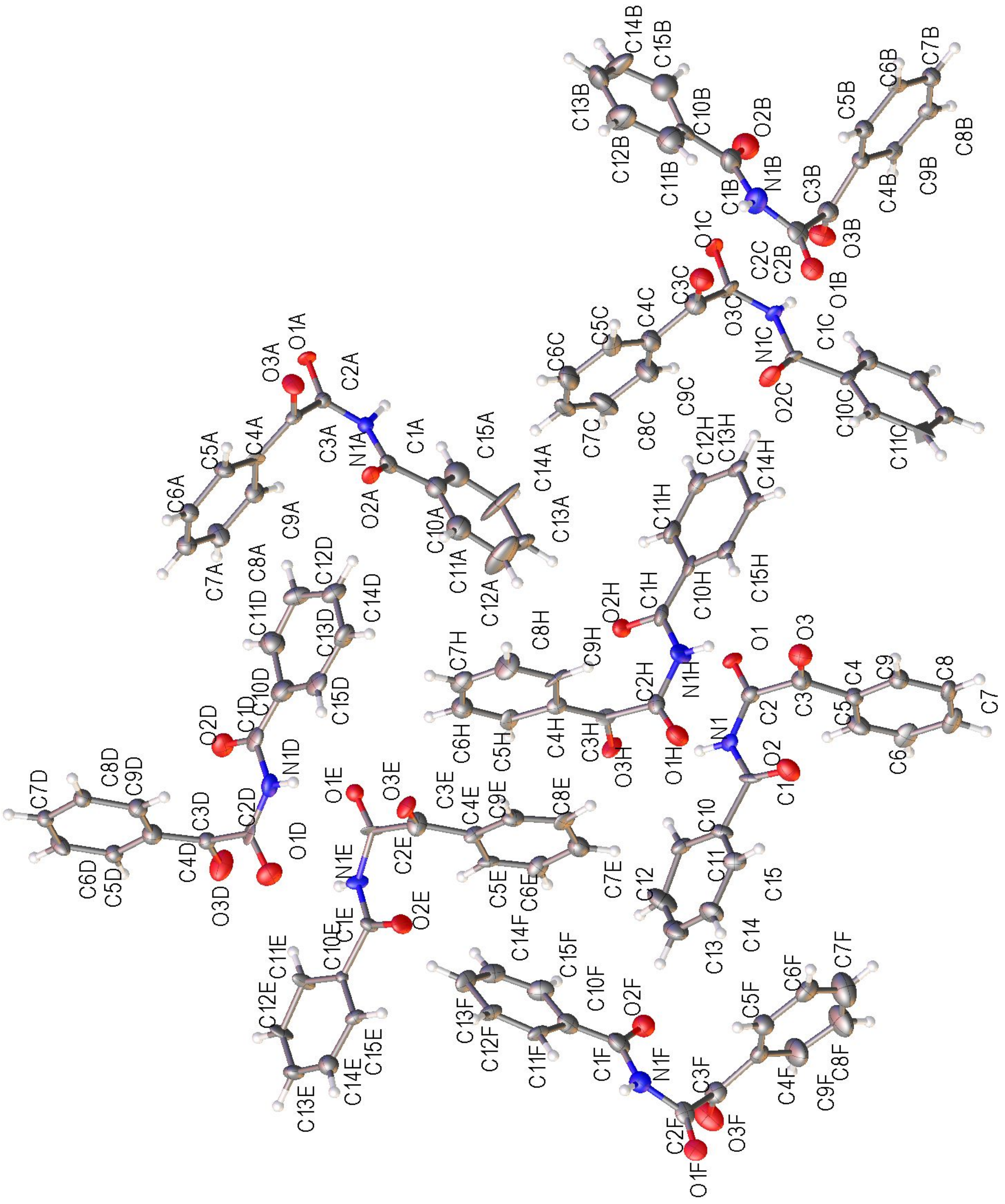

Perspective view of **23** with displacement ellipsoids drawn at the 50% probability level.
Crystal data and structure refinement for **23**

Identification code	**23**
Empirical formula	$C_{15}H_{11}NO_3$
Formula weight	253.25
Temperature/K	100.0
Crystal system	Triclinic

Space group	P1
a/Å	5.449(3)
b/Å	21.007(9)
c/Å	21.065(9)
α/°	88.01(2)
β/°	82.915(15)
γ/°	84.80(3)
Volume/Å^3	2382.2(18)
Z	8
ρ_{calc}g/cm^3	1.412
μ/mm^{-1}	0.820
F(000)	1056.0
Crystal size/mm^3	0.54 × 0.04 × 0.02
Radiation	CuKα (λ = 1.54178)
2Θ range for data collection/°	4.224 to 158.088
Index ranges	-6 ≤ h ≤ 6, -26 ≤ k ≤ 26, -26 ≤ l ≤ 26
Reflections collected	105763
Independent reflections	17524 [R_{int} 0.0731, R_{sigma} = 0.0575]
Data/restraints/parameters	17524/1230/1333
Goodness-of-fit on F^2	1.068
Final R indexes [I>=2σ (I)]	R_1 = 0.1327, wR_2 = 0.3894
Final R indexes [all data]	R_1 = 0.1444, wR_2 = 0.3971
Largest diff. peak/hole / e Å^{-3}	1.06/-0.97
Flack parameter	0.28(19)

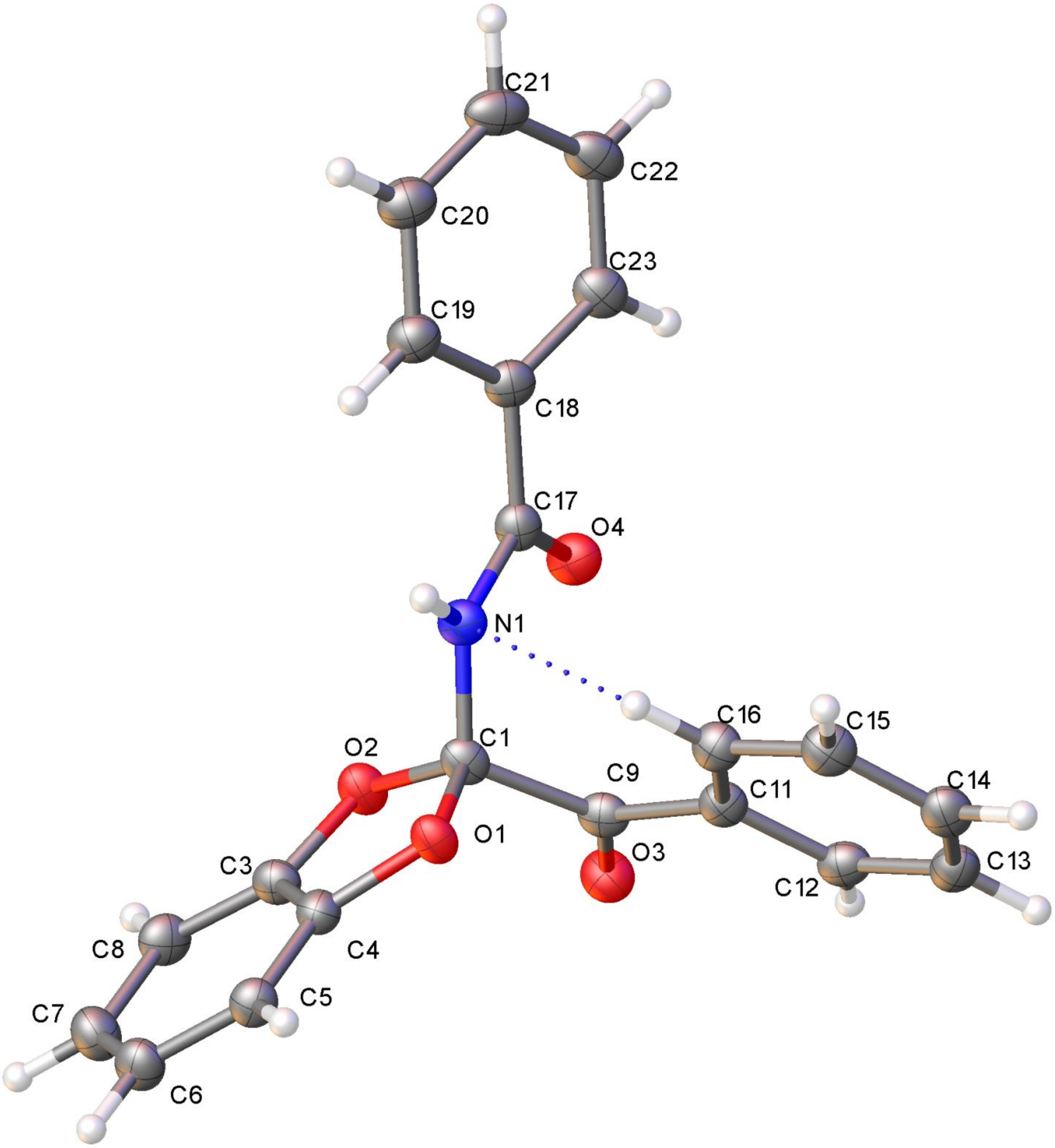

Perspective view of **27** with displacement ellipsoids drawn at the 50% probability level

Crystal data and structure refinement for **27**

Identification code	27
Empirical formula	$C_{21}H_{15}NO_4$
Formula weight	345.34
Temperature/K	100.0
Crystal system	Monoclinic
Space group	Pn
a/Å	9.237(4)
b/Å	9.516(4)
c/Å	9.930(5)
α/°	90
β/°	113.65(3)
γ/°	90
Volume/Å^3	799.5(6)
Z	2
ρ_{calc}g/cm^3	1.435
μ/mm^{-1}	0.822
F(000)	360.0
Crystal size/mm^3	0.28 × 0.2 × 0.04
Radiation	CuKα (λ = 1.54178)
2Θ range for data collection/°	9.294 to 150.82
Index ranges	$-11 \leq h \leq 11$, $-11 \leq k \leq 11$, $-12 \leq l \leq 12$
Reflections collected	26084
Independent reflections	3244 [R_{int} = 0.0697, R_{sigma} = 0.0339]
Data/restraints/parameters	3244/2/237
Goodness-of-fit on F^2	1.074
Final R indexes [I>=2σ (I)]	R_1 = 0.0350, wR_2 = 0.0875
Final R indexes [all data]	R_1 = 0.0375, wR_2 = 0.0895
Largest diff. peak/hole / e Å^{-3}	0.19/-0.20
Flack parameter	0.1(2)

S6. References

(1) Dolomanov, O. V; Bourhis, L. J.; Gildea, R. J.; Howard, J. A. K.; Puschmann, H. OLEX2: A Complete Structure Solution, Refinement and Analysis Program. *J. Appl. Crystallogr.* **2009**, *42*, 339–341. https://doi.org/10.1107/S0021889808042726

(2) Sheldrick, G. M. SHELXT: Integrated Space-Group and Crystal-Structure Determination. *Acta Crystallogr., Sect. A* **2015**, *71*, 3–8. https://doi.org/10.1107/S2053273314026370

(3) Long, C. A.; Kearns, D. R. Radiationless decay of singlet molecular oxygen in solution. II. Temperature dependence and solvent effects. *J. Am. Chem. Soc.* **1975**, *97*, 2018–2020. https://doi.org/10.1021/ja00841a004

(4) Bregnhøj, M.; Westberg, M.; Jensen, F.; Ogilby, P. R. Solvent-Dependent Singlet Oxygen Lifetimes: Temperature Effects Implicate Tunneling and Charge-Transfer Interactions. *Phys. Chem. Chem. Phys.* **2016**, *18*, 22946–22961. https://doi.org/10.1039/c6cp01635a

(5) Hurst, J. R.; McDonald, J. D.; Schuster, G. B. Lifetime of singlet oxygen in solution directly determined by laser spectroscopy. *J. Am. Chem. Soc.* **1982**, *104*, 2065–2067. https://doi.org/10.1021/ja00371a065

(6) Merkel, P. B.; Nilsson, R.; Kearns, D. R. Deuterium effects on singlet oxygen lifetimes in solutions. New test of singlet oxygen reactions. *J. Am. Chem. Soc.* **1972**, *94*, 1030–1031. https://doi.org/10.1021/ja00758a072

(7) Ogilby, P. R.; Foote, C. S. Chemistry of singlet oxygen. 34. Unexpected solvent deuterium isotope effects on the lifetime of singlet molecular oxygen (1.DELTA.g). *J. Am. Chem. Soc.* **1981**, *103*, 1219–1221. https://doi.org/10.1021/ja00395a041

(8) Alder, C. M.; Hayler, J. D.; Henderson, R. K.; Redman, A. M.; Shukla, L.; Shuster, L. E.; Sneddon, H. F. Updating and further expanding GSK's solvent sustainability guide. *Green Chem.* **2016**, *18*, 3879–3890. https://doi.org/10.1039/C6GC00611F

(9) Lutkus, L. V.; Rickenbach, S. S.; McCormick, T. M. Singlet Oxygen Quantum Yields Determined by Oxygen Consumption. *J. Photochem. Photobiol., A* **2019**, *378*, 131–135. https://doi.org/10.1016/j.jphotochem.2019.04.029

(10) Thomson, C. G.; Jones, C. M. S.; Rosair, G.; Ellis, D.; Marques-Hueso, J.; Lee, A.-L.; Vilela, F. Continuous-flow synthesis and application of polymer-supported BODIPY Photosensitisers for the generation of singlet oxygen; process optimised by in-line NMR spectroscopy. *J. Flow Chem.* **2020**, *10*, 327–345. https://doi.org/10.1007/s41981-019-00067-4

(11) Zhang, D.; Ye, B.; Ho, D. G.; Gao, R.; Selke, M. Chemistry of singlet oxygen with arylphosphines. *Tetrahedron* **2006**, *62*, 10729–10733. https://doi.org/10.1016/j.tet.2006.07.112

(12) Thomson, C. G.; Lee, A.-L.; Vilela, F. Heterogeneous photocatalysis in flow chemical reactors. *Beilstein J. Org. Chem.* **2020**, *16*, 1495–1549. https://doi.org/10.3762/bjoc.16.125

(13) Kollmann, J.; Zhang, Y.; Schilling, W.; Zhang, T.; Riemer, D.; Das, S. A Simple Ketone as an Efficient Metal-Free Catalyst for Visible-Light-Mediated Diels-Alder and Aza-Diels-Alder Reactions. *Green Chem.* **2019**, *21*, 1916–1920. https://doi.org/10.1039/c9gc00485h

(14) Fatima, K.; Masood, N.; Luqman, S. Quenching of Singlet Oxygen by Natural and Synthetic Antioxidants and Assessment of Electronic UV/Visible Absorption Spectra for Alleviating or Enhancing the Efficacy of Photodynamic Therapy. *Biomed. Res. Ther.* **2016**, *3*, 514-527. https://doi.org/10.15419/bmrat.v3i02.82

(15) Mendoza, C.; Emmanuel, N.; Páez, C. A.; Dreesen, L.; Monbaliu, J.-C. M.; Heinrichs, B. Improving Continuous Flow Singlet Oxygen Photooxygenation Reactions with Functionalized Mesoporous Silica Nanoparticles. *ChemPhotoChem* **2018**, *2*, 890–897. https://doi.org/10.1002/cptc.201800148

(16) Li, X.; Li, J.; Bai, J.; Dong, Y.; Li, L.; Zhou, B. The Inhibition Effect of Tert-Butyl Alcohol on the TiO2 Nano Assays Photoelectrocatalytic Degradation of Different Organics and Its Mechanism. *Nano-Micro Lett.* **2016**, *8*, 221–231. https://doi.org/10.1007/s40820-015-0080-2

(17) Hayyan, M.; Hashim, M. A.; AlNashef, I. M. Superoxide Ion: Generation and Chemical Implications. *Chem. Rev.* **2016**, *116*, 3029–3085. https://doi.org/10.1021/acs.chemrev.5b00407

(18) Bilski, P.; Reszka, K.; Bilska, M.; Chignell, C. F. Oxidation of the Spin Trap 5,5-Dimethyl-1-pyrroline *N*-Oxide by Singlet Oxygen in Aqueous Solution. *J. Am. Chem. Soc.* **1996**, *118*, 1330–1338. https://doi.org/10.1021/ja952140s

(19) Skolia, E.; Gkizis, P. L.; Kokotos, C. G. Aerobic Photocatalysis: Oxidation of Sulfides to Sulfoxides. *ChemPlusChem* **2022**, *87*, e202200008. https://doi.org/10.1002/cplu.202200008

(20) Sofikiti, N.; Rabalakos, C.; Stratakis, M. Efficient Trapping of the Intermediates in the Photooxygenation of Sulfides by Aryl Selenides and Selenoxides. *Tetrahedron Lett.* **2004**, *45*, 1335–1337. https://doi.org/10.1016/j.tetlet.2003.12.065

(21) Pierlot, C.; Nardello, V.; Schrive, J.; Mabille, C.; Barbillat, J.; Sombret, B.; Aubry, J. M. Calcium Peroxide Diperoxohydrate as a Storable Chemical Generator of Singlet Oxygen for Organic Synthesis. *J. Org. Chem.* **2002**, *67*, 2418–2423. https://doi.org/10.1021/jo010766x

(22) You, Y. Chemical Tools for the Generation and Detection of Singlet Oxygen. *Org. Biomol. Chem.* **2018**, *16*, 4044–4060. https://doi.org/10.1039/C8OB00504D

(23) Wilkinson, F.; Helman, W. P.; Ross, A. B. Rate Constants for the Decay and Reactions of the Lowest Electronically Excited Singlet State of Molecular Oxygen in Solution. An Expanded and Revised Compilation. *J. Phys. Chem. Ref. Data* **1995**, *24*, 663–677. https://doi.org/10.1063/1.555965

(24) Varchola, J.; Želonková, K.; Chorvat, D.; Jancura, D.; Miskovsky, P.; Bánó, G. Singlet Oxygen Produced by Quasi-Continuous Photo-Excitation of Hypericin in Dimethyl-Sulfoxide. *J. Lumin.* **2016**, *177*, 17–21. https://doi.org/10.1016/j.jlumin.2016.04.020

(25) Staicu, A.; Pascu, A.; Boni, M.; Pascu, M. L.; Enescu, M. Photophysical Study of Zn Phthalocyanine in Binary Solvent Mixtures. *J. Mol. Struct*. **2013**, *1044*, 188–193. https://doi.org/10.1016/j.molstruc.2012.12.010.

(26) Kalogirou, A. S.; Kourtellaris, A.; Koutentis, P. A. Oxidations of 4*H*-1,2,6-thiadiazines. *ChemistrySelect* **2022**, *7*, e202204204. https://doi.org/10.1002/slct.202204204

(27) Samuel, D.; Norrell, K.; Hilmey, D. G. Novel ring chemistry of vitamin B6 with singlet oxygen and an activated ene: isolated products and identified intermediates suggesting an operable [3 + 2] cycloaddition. Org. Biomol. Chem. 2012, 10, 7278-7281. https://doi.org/10.1039/C2OB26067K

(28) Frisch, M. J.; Trucks, G. W.; Schlegel, H. B.; Scuseria, G. E.; Robb, M. A.; Cheeseman, J. R.; Scalmani, G.; Barone, V.; Petersson, G. A.; Nakatsuji, H.; Li, X.; Caricato, M.; Marenich, A. V.; Bloino, J.; Janesko, B. G.; Gomperts, R.; Mennucci, B.; Hratchian, H. P.; Ortiz, J. V.; Izmaylov, A. F.; Sonnenberg, J. L.; Williams-Young, D.; F. Ding, F. L.; F. Egidi, J. G.; B. Peng, A. P.; Henderson, T.; Ranasinghe, D.; Zakrzewski, V. G.; Gao, J.; Rega, N.; Zheng, G.; Liang, W.; Hada, M.; Ehara, M.; Toyota, K.; Fukuda, R.; Hasegawa, J.; Ishida, M.; Nakajima, T.; Honda, Y.; Kitao, O.; Nakai, H.; Vreven, T.; Throssell, K.; J. A. Montgomery, J.; Peralta, J. E.; Ogliaro, F.; Bearpark, M. J.; Heyd, J. J.; Brothers, E. N.; Kudin, K. N.; Staroverov, V. N.; Keith, T. A.; Kobayashi, R.; Normand, J.; Raghavachari, K.; Rendell, A. P.; Burant, J. C.; Iyengar, S. S.; Tomasi, J.; Cossi, M.; Millam, J. M.; Klene, M.; Adamo, C.; Cammi, R.; Ochterski, J. W.; Martin, R. L.; Morokuma, K.; Farkas, O.; Foresman, J. B.; Fox, D. J. *Gaussian 16, Revision A.03* Gaussian Inc.: Wallingford CT, 2016.

(29) Siegbahn, P. E. M.; Almlöf, J.; Heiberg, A.; Roos, B. O. The complete active space SCF (CASSCF) method in a Newton–Raphson formulation with application to the HNO molecule. *J. Chem. Phys.* **1981**, *74* (4), 2384-2396. https://doi.org/10.1063/1.441359.

(30) Roos, B. O.; Taylor, P. R.; Siegbahn, P. E. M. A complete active space SCF method (CASSCF) using a density matrix formulated super-CI approach. *Chem. Phys.* **1980**, *48*, 157-173. https://doi.org/10.1016/0301-0104(80)80045-0

(31) Hehre, W. J.; Ditchfield, R.; Pople, J. A. Self-Consistent Molecular Orbital Methods. XII. Further Extensions of Gaussian-Type Basis Sets for Use in Molecular Orbital Studies of Organic Molecules. *J. Chem. Phys.* **1972**, *56* (5), 2257-2261. https://doi.org/10.1063/1.1677527.

(32) Hariharan, P. C.; Pople, J. A. The influence of polarization functions on molecular orbital hydrogenation energies. *Theor. Chim. Acta* **1973**, *28* (3), 213-222. https://doi.org/10.1007/BF00533485

(33) Becke, A. D. Density-functional thermochemistry. III. The role of exact exchange. *J. Chem. Phys.* **1993**, *98* (7), 5648-5652. https://doi.org/10.1063/1.464913

(34) Andersson, K.; Malmqvist, P. Å.; Roos, B. O. Second-order perturbation theory with a complete active space self-consistent field reference function. *J. Chem. Phys.* **1992**, *96* (2), 1218-1226. https://doi.org/10.1063/1.462209.
(35) Andersson, K.; Malmqvist, P. Å.; Roos, B. O.; Sadlej, A. J.; Wolinsk, K. Second-Order Perturbation Theory with a CASSCF Reference Function. *J. Phys. Chem.* **1990**, *94*, 5483-5488. https://doi.org/10.1021/j100377a012
(36) Werner, H.-J.; Knowles, P. J.; Knizia, G.; Manby, F. R.; Schütz, M.; Celani, P.; Györffy, W.; Kats, D.; Korona, T.; Lindh, R.; Mitrushenkov, A.; Rauhut, G.; Shamasundar, K. R.; Adler, T. B.; Amos, R. D.; Bennie, S. J.; Bernhardsson, A.; Berning, A.; Cooper, D. L.; Deegan, M. J. O.; Dobbyn, A. J.; Eckert, F.; Goll, E.; Hampel, C.; Hesselmann, A.; Hetzer, G.; Hrenar, T.; Jansen, G.; Köppl, C.; Lee, S. J. R.; Liu, Y.; Lloyd, A. W.; Ma, Q.; Mata, R. A.; May, A. J.; McNicholas, S. J.; Meyer, W.; III, T. F. M.; Mura, M. E.; Nicklass, A.; O'Neill, D. P.; Palmieri, P.; Peng, D.; Petrenko, T.; Pflüger, K.; Pitzer, R.; Reiher, M.; Shiozaki, T.; Stoll, H.; Stone, A. J.; Tarroni, R.; Thorsteinsson, T.; Wang, M.; Welborn, M. *MOLPRO, 2012.1, a package of ab initio programs*; see https://www.molpro.net: 2012.
(37) Celani, P.; Werner, H. J. Multireference perturbation theory for large restricted and selected active space reference wave functions. *J. Chem. Phys.* **2000**, *112*, 5546-5557. https://doi.org/10.1063/1.481132
(38) Ghigo, G.; Roos, B. O.; Malmqvist, P.-Å. A modified definition of the zeroth-order Hamiltonian in multiconfigurational perturbation theory (CASPT2). *Chem. Phys. Lett.* **2004**, *396* (1-3), 142-149. https://doi.org/10.1016/j.cplett.2004.08.032
(39) Tomasi, J.; Mennucci, B.; Cammi, R. Quantum mechanical continuum solvation models. *Chem. Rev.* **2005**, *105* (8), 2999-3093. https://doi.org/10.1021/cr9904009
(40) Geevers, J.; Trompen, W. P. Synthesis and Reactions of 3,5-Dichloro-4*H*-1,2,6-thiadiazin-4-one. *Rec. Trav. Chim. Pays-Bas* **1974**, *93*, 270–272. https://doi.org/10.1002/recl.19740930911.
(41) Ioannidou, H. A.; Koutentis, P. A. Synthesis of Asymmetric 3,5-Diaryl-4*H*-1,2,6-thiadiazin-4-ones *via* Suzuki–Miyaura and Stille Coupling Reactions. *Tetrahedron* **2012**, *68*, 7380–7385. https://doi.org/10.1016/j.tet.2012.06.079
(42) Kalogirou, A. S.; Koutentis, P. A. Pd-catalyzed C-N Coupling of Primary (Het)arylamines with 5-Substituted 3-Chloro-4*H*-1,2,6-thiadiazin-4-ones. *Tetrahedron Lett.* **2018**, *59*, 2653‑2656. https://doi.org/10.1016/j.tetlet.2018.05.068
(43) Kalogirou, A. S.; Manoli, M.; Koutentis, P. A. Synthesis of *N*-Aryl-3,5-dichloro-4*H*-1,2,6-thiadiazin-4-imines from 3,4,4,5-Tetrachloro-4*H*-1,2,6-thiadiazine. *Org. Lett.* **2015**, *17*, 4118–4121. https://doi.org/10.1021/acs.orglett.5b02237.
(44) Kalogirou, A. S.; Koutentis, P. A. Regioselective Geminal Dichloride Reactivity of 3,4,4,5-Tetrachloro-4*H*-1,2,6-thiadiazine: Access to 4,4-Dioxo- and Dithio-Ketals. *Tetrahedron Lett.* **2016**, *57*, 203–205. https://doi.org/10.1016/j.tetlet.2015.11.100
(45) Kalogirou, A. S.; Kourtellaris, A.; Koutentis, P. A. The Acid and/or Thermal Mediated Ring Contraction of 4*H*-1,2,6-Thiadiazines To Afford 1,2,5-Thiadiazoles. *Org. Lett.* **2016**, *18*, 4056–4059. https://doi.org/10.1021/acs.orglett.6b01929
(46) Ioannidou, H. A.; Kizas, C.; Koutentis, P. A. Palladium Catalyzed C-C Coupling Reactions of 3,5-Dichloro-4*H*-1,2,6-thiadiazin-4-one. *Org. Lett.* **2011**, *13*, 3466–3469. https://doi.org/10.1021/ol201212b
(47) Konstantinova, L. S.; Knyazeva, E. A.; Gatilov, Yu. V; Zlotin, S. G.; Rakitin, O. A. Nitro Derivatives of 2,1,3-Benzothiadiazole 1-Oxides: Synthesis, Structural Study, and NO Release. *Russ. Chem. Bull.* **2018**, *67*, 95–101. https://doi.org/10.1007/s11172-018-2042-6
(48) Kalmode, H. P.; Vadagaonkar, K. S.; Chaskar, A. C. The Oxidative Cross-Coupling of Benzonitriles with Multiform Substrates: A Domino Strategy Inspired Easy Access to α-Keto-Imides. *Synthesis (Stuttg)* **2015**, *47*, 429–438. https://doi.org/10.1055/s-0034-1379955
(49) Kalmode, H. P.; Vadagaonkar, K. S.; Chaskar, A. C. Metal-Free in Situ *sp*^{3}, *sp*^{2}, and *sp* C-H Functionalization and Oxidative Cross Coupling with Benzamidines Hydrochloride: A Promising Approach for the Synthesis of α-Ketoimides. *RSC Adv.* **2014**, *4*, 60316–60326. https://doi.org/10.1039/c4ra07556k

(50) McCulla, R. D.; Jenks, W. S. Deoxygenation and Other Photochemical Reactions of Aromatic Selenoxides. *J. Am. Chem. Soc.* **2004**, *126*, 16058–16065. https://doi.org/10.1021/ja045935k

(51) Weilbeer, C.; Selent, D.; Dyballa, K. M.; Franke, R.; Spannenberg, A.; Börner, A. Evaluation of Organoselenium Based Compounds as Co-Catalysts in Rhodium-Catalyzed Hydroformylation. *ChemistrySelect* **2016**, *1*, 5421–5429. https://doi.org/10.1002/slct.201601215

(52) Rezaei, R.; Mohammadi, M. K.; Rastin, N. Microwave Assisted, Solvent Free One Pot Synthesis of Nitriles from Aryl Aldehydes on Melamin Formaldehyde as Solid Support. *Chin. J. Chem.* **2010**, *28*, 993–996. https://doi.org/10.1002/cjoc.201090184

S7. ^{1}H and ^{13}C NMR Spectra of New Compounds

DMSO-d6

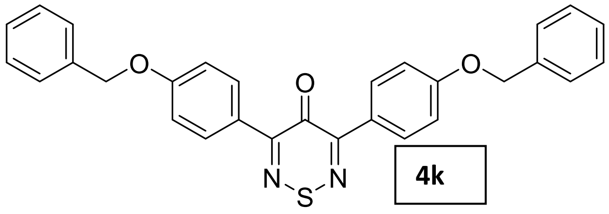

4k

500 MHz

3,5-Bis[4-(benzyloxy)phenyl]-4H-1,2,6-thiadiazin-4-one (**34a**)

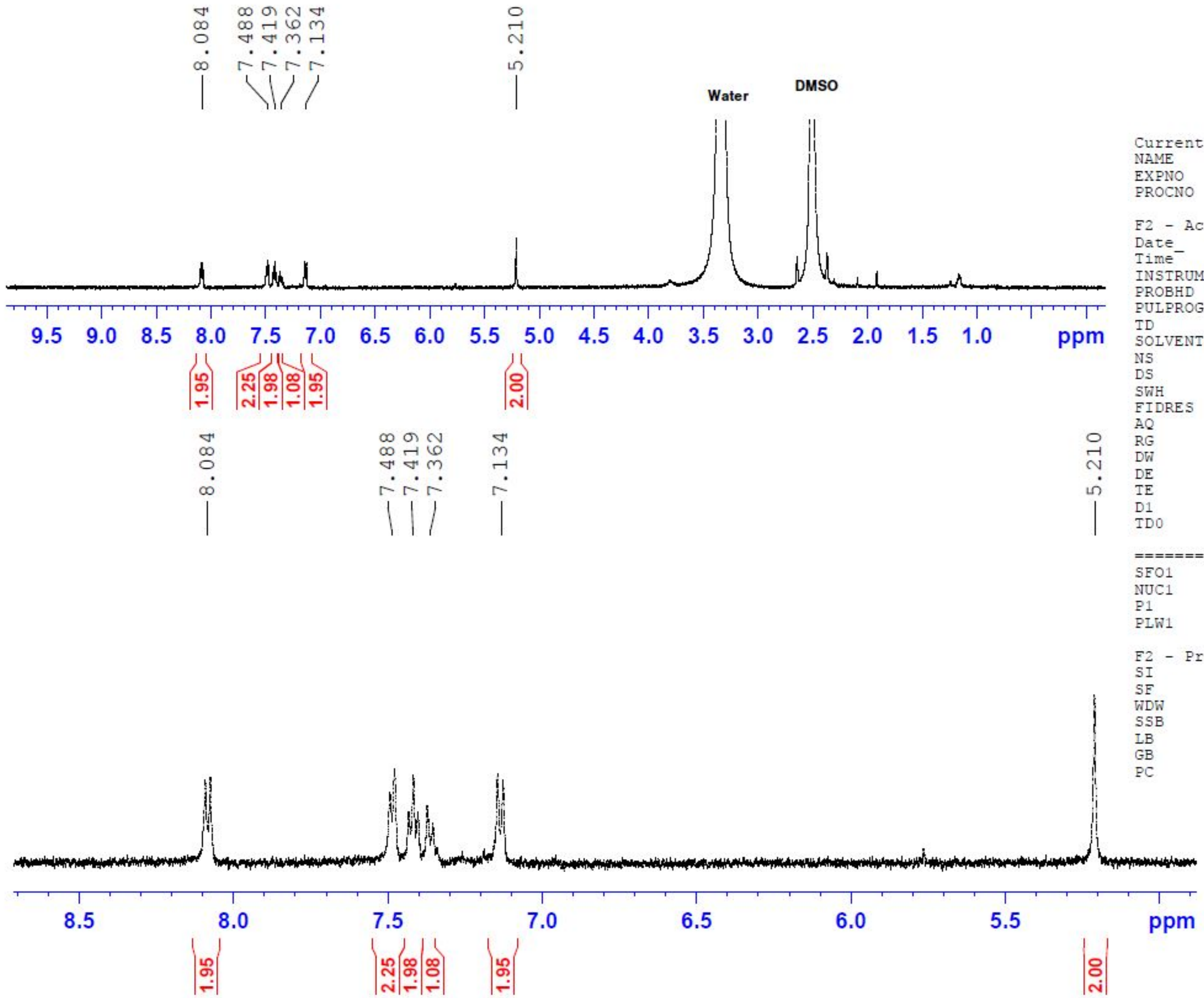

Current Data Parameters
NAME Andreas Kalogirou
EXPNO 1061
PROCNO 1

F2 - Acquisition Parameters
Date_ 20150501
Time 21.44
INSTRUM spect
PROBHD 5 mm PABBO BB-
PULPROG zg30
TD 65536
SOLVENT DMSO
NS 16
DS 2
SWH 10000.000 Hz
FIDRES 0.152588 Hz
AQ 3.2767999 sec
RG 161
DW 50.000 usec
DE 6.50 usec
TE 297.4 K
D1 1.00000000 sec
TD0 1

======== CHANNEL f1 ========
SFO1 500.0361158 MHz
NUC1 1H
P1 11.75 usec
PLW1 15.41699982 W

F2 - Processing parameters
SI 65536
SF 500.0330279 MHz
WDW EM
SSB 0
LB 0.30 Hz
GB 0
PC 1.00

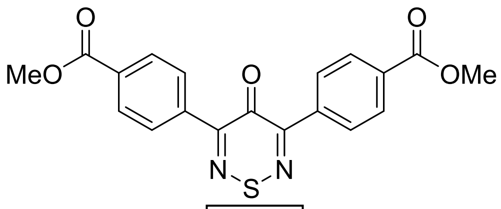

4l

500 MHz

CD_2Cl_2

Dimethyl 4,4'-(4-oxo-4H-1,2,6-thiadiazine-3,5-diyl)dibenzoate (**32a**)

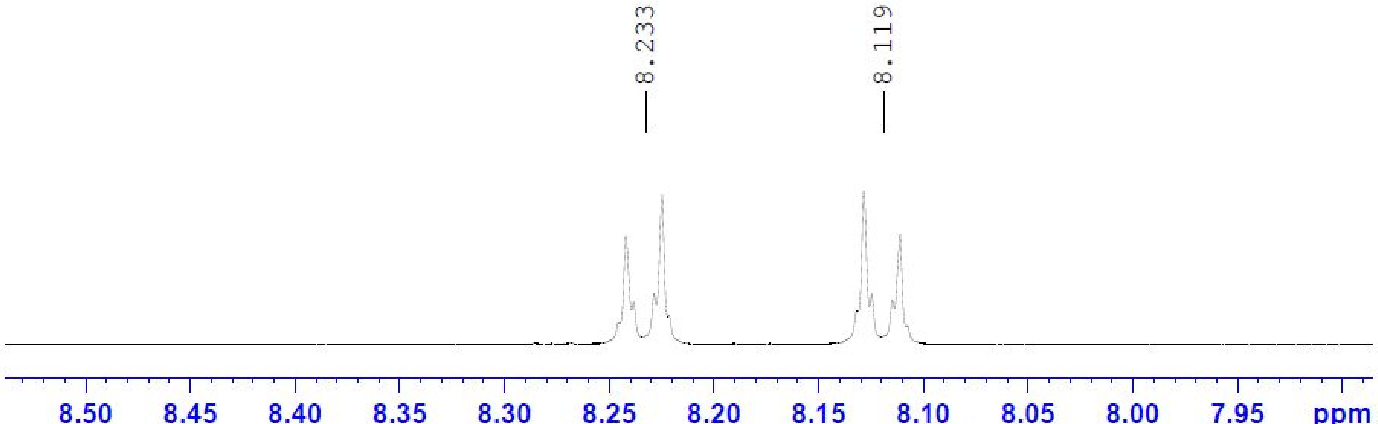

Current Data Parameters
NAME Kalogirou
EXPNO 639
PROCNO 1

F2 - Acquisition Parameters
Date_ 20190814
Time 11.51
INSTRUM spect
PROBHD 5 mm PABBO BB-
PULPROG zg30
TD 65536
SOLVENT CD2Cl2
NS 16
DS [illegible]

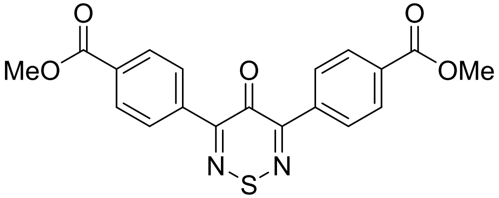

4l

125 MHz

CD$_2$Cl$_2$

Dimethyl 4,4'-(4-oxo-4H-1,2,6-thiadiazine-3,5-diyl)dibenzoate (**32a**)

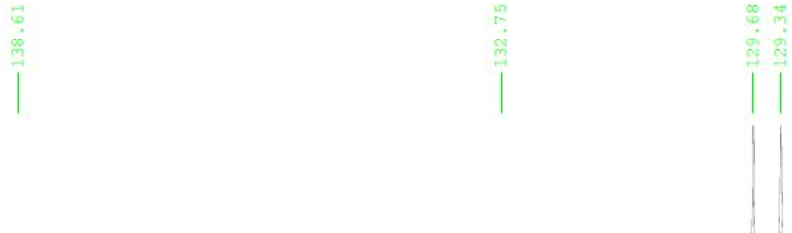

Current Data Parameters
NAME Kalogirou
EXPNO 638
PROCNO 1

F2 - Acquisition Parameters

500 MHz

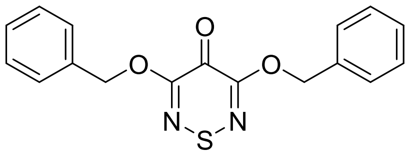

4s

CDCl$_3$

3,5-Bis(benzyloxy)-4H-1,2,6-thiadiazin-4-one (**24a**)

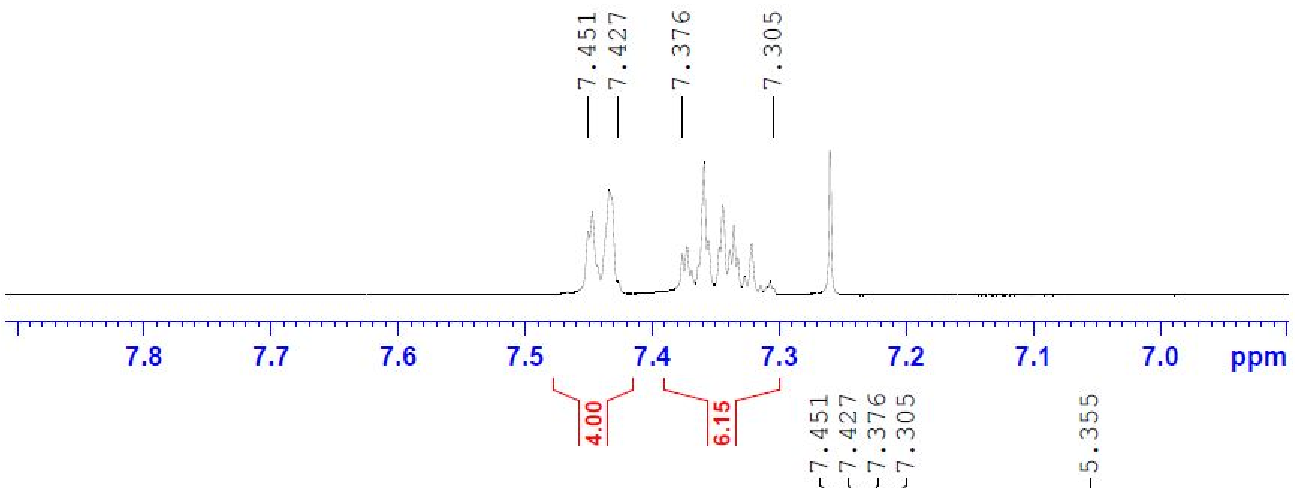

Current Data Parameters	
NAME	Kalogirou
EXPNO	924
PROCNO	1
F2 - Acquisition Parameters	
Date_	20201210
Time	14.40
INSTRUM	spect
PROBHD	5 mm PABBO BB-
PULPROG	zg30
TD	65536
SOLVENT	CDCl3
NS	16
DS	2
SWH	10000.000 Hz
FIDRES	0.152588 Hz
AQ	3.2767999 sec

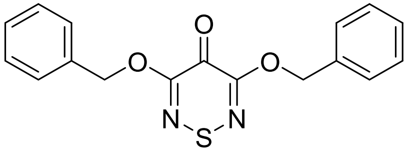

4s

125 MHz

CDCl$_3$

3,5-Bis(benzyloxy)-4H-1,2,6-thiadiazin-4-one (**24a**)

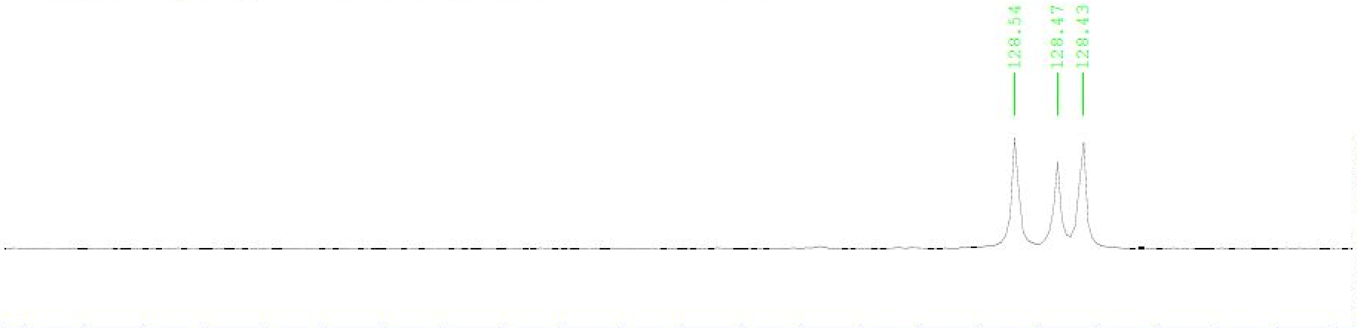

Current Data Parameters
NAME Kalogirou
EXPNO 925
PROCNO 1

F2 - Acquisition Parameters
Date_ 20201210
Time 16.10
INSTRUM spect
PROBHD 5 mm PABBO BB-

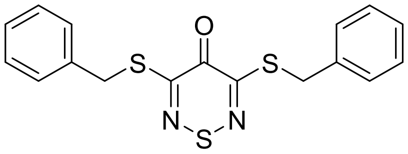

9

500 MHz

CDCl$_3$

3,5-Bis(benzylthio)-4H-1,2,6-thiadiazin-4-one (**26a**)

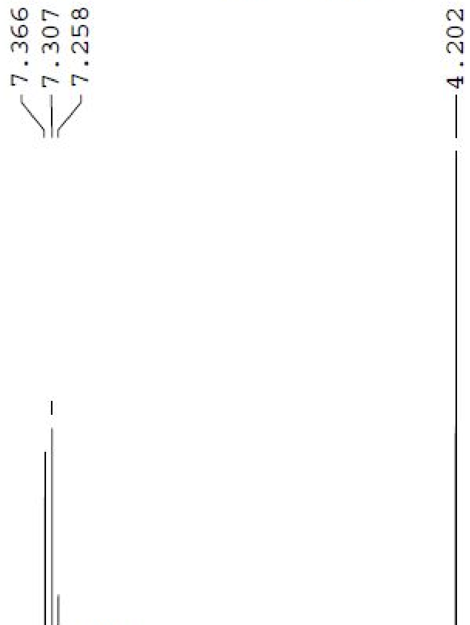

Current Data Parameters
NAME Andreas Kalogirou
EXPNO 480
PROCNO 1

F2 - Acquisition Parameters

Date_	20131118	
Time	17.07	
INSTRUM	spect	
PROBHD	5 mm PABBO BB-	
PULPROG	zg30	
TD	65536	
SOLVENT	CDCl3	
NS	16	
DS	2	
SWH	10330.578	Hz
FIDRES	0.157632	Hz
AQ	3.1719425	sec
RG	128	
DW	48.400	usec
DE	6.50	usec
TE	297.8	K
D1	1.00000000	sec
TD0	1	

125 MHz

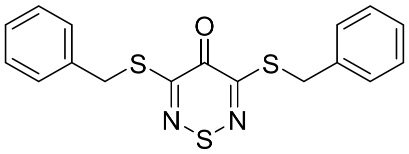

9

$CDCl_3$

3,5-Bis(benzylthio)-4H-1,2,6-thiadiazin-4-one (**26a**)

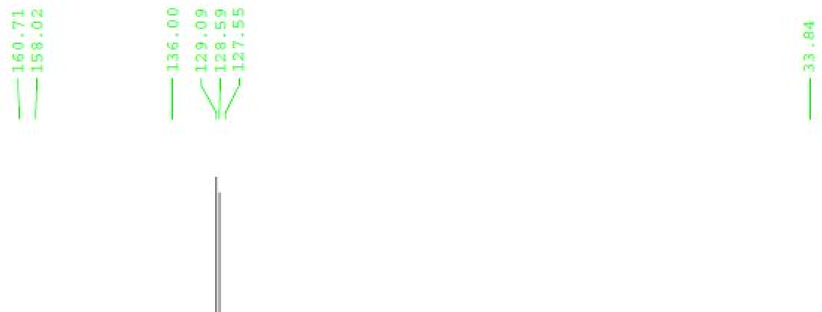


```
Current Data Parameters
NAME      Andreas Kalogirou
EXPNO                  481
PROCNO                   1

F2 - Acquisition Parameters
Date_           20131118
Time               17.11
INSTRUM            spect
PROBHD   5 mm PABBO BB
```

500 MHz

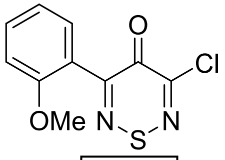

21

CDCl$_3$

3-Chloro-5-(2-methoxyphenyl)-4H-1,2,6-thiadiazin-4-one (**Int1**)

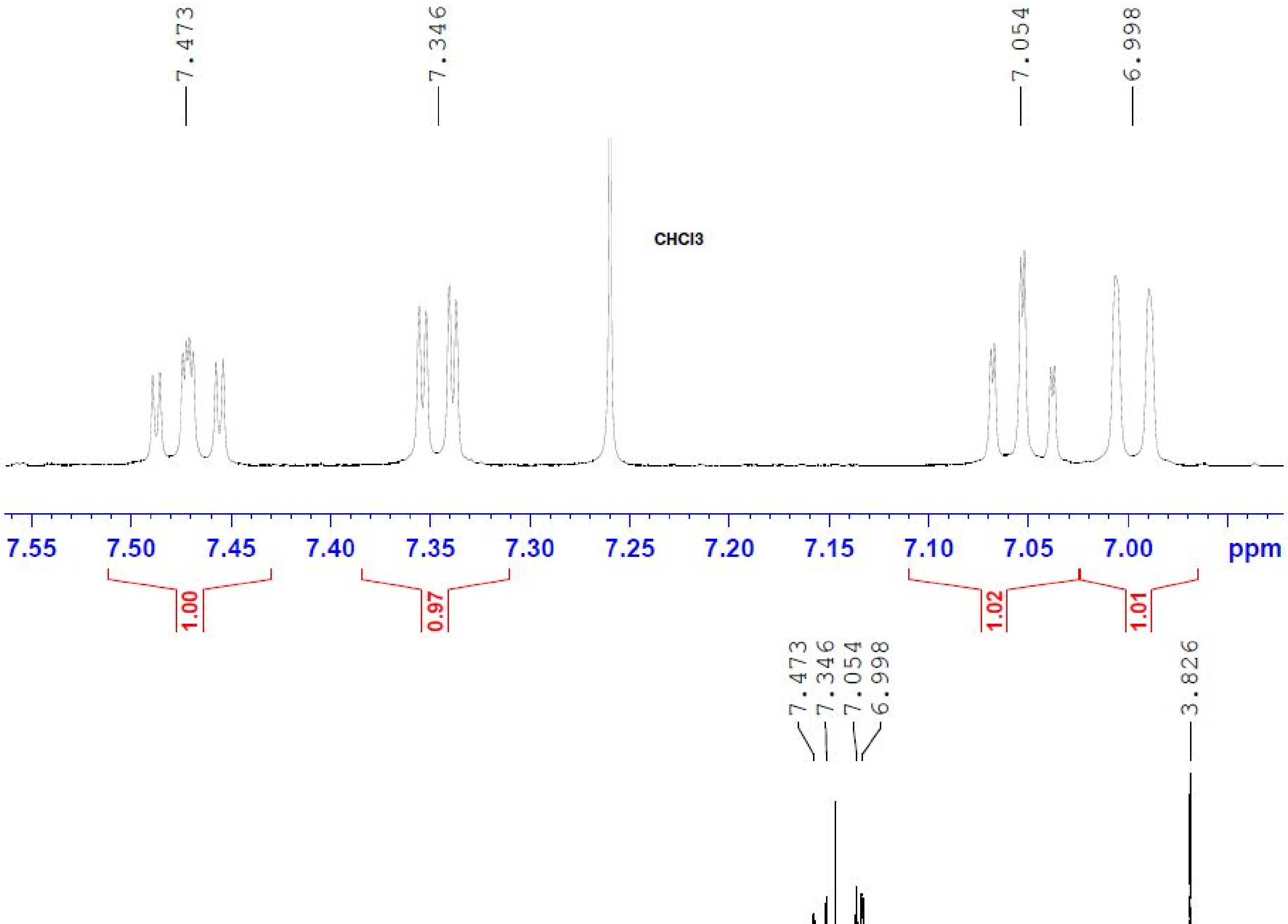


```
Current Data Parameters
NAME            Kalogirou
EXPNO                 903
PROCNO                  1

F2 - Acquisition Parameters
Date_            20201103
Time                18.43
INSTRUM             spect
PROBHD    5 mm PABBO BB-
PULPROG              zg30
TD                  65536
SOLVENT             CDCl3
NS                     16
DS                      2
SWH             10000.000 Hz
FIDRES           0.152588 Hz
AQ              3.2767999 sec
RG                    144
DW                 50.000 usec
DE                   6.50 usec
TE                  295.4 K
D1             1.00000000 sec
TD0                     1

======== CHANNEL f1 ========
SFO1          500.0361158 MHz
NUC1                   1H
P1                  12.00 usec
PLW1          14.50000000 W

F2 - Processing parameters
SI                  65536
SF            500.0330400 MHz
WDW                    EM
SSB        0
LB                   0.30 Hz
GB         0
PC                   1.00
```

125 MHz

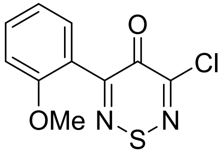

21

CDCl$_3$

CDCl$_3$

3-Chloro-5-(2-methoxyphenyl)-4H-1,2,6-thiadiazin-4-one (**Int1**).

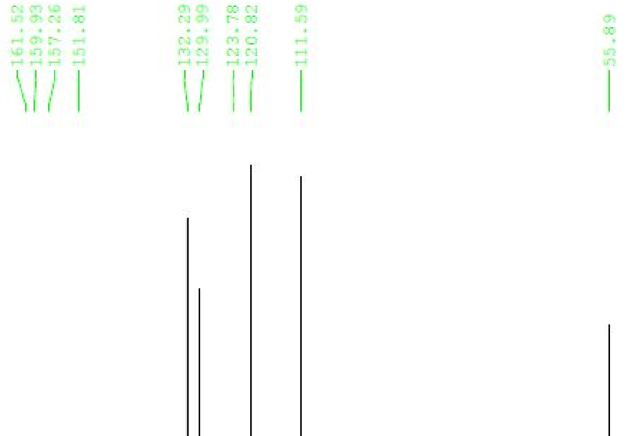

Current Data Parameters
NAME Kalogirou
EXPNO 904
PROCNO 1

F2 - Acquisition Parameters
Date_ 20201104
Time 2.11
INSTRUM spect
PROBHD 5 mm PABBO BB-
PULPROG jmod
TD 65536
SOLVENT CDCl3
NS 7000
DS 4

500 MHz

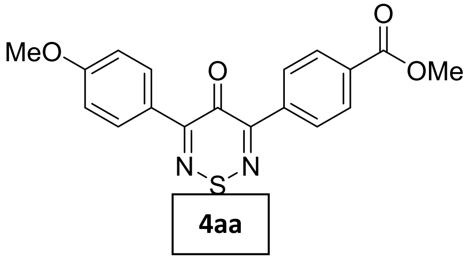

4aa

CDCl$_3$

CDCl$_3$

Methyl 4-[5-(4-methoxyphenyl)-4-oxo-4H-1,2,6-thiadiazin-3-yl]benzoate (**40a**)

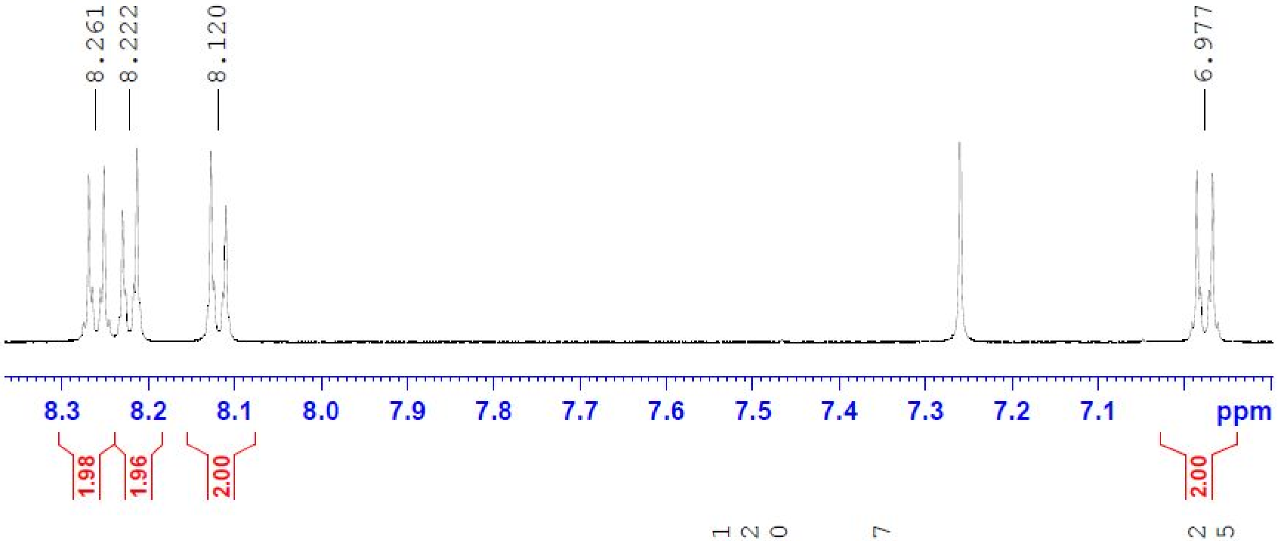

Current Data Parameters
NAME Kalogirou
EXPNO 786
PROCNO 1

F2 - Acquisition Parameters
Date_ 20200306
Time 15.29
INSTRUM spect
PROBHD 5 mm PABBO BB-
PULPROG zg30
TD 65536
SOLVENT CDCl3
NS 16
DS 2
SWH 10000.000 Hz
FIDRES 0.152588 Hz
AQ 3.2767999 sec
RG 181
DW 50.000 usec
DE 6.50 usec

125 MHz

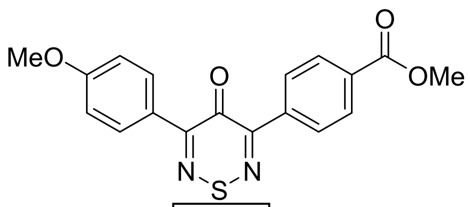

4aa

$CDCl_3$

Methyl 4-[5-(4-methoxyphenyl)-4-oxo-4H-1,2,6-thiadiazin-3-yl]benzoate (**40a**)

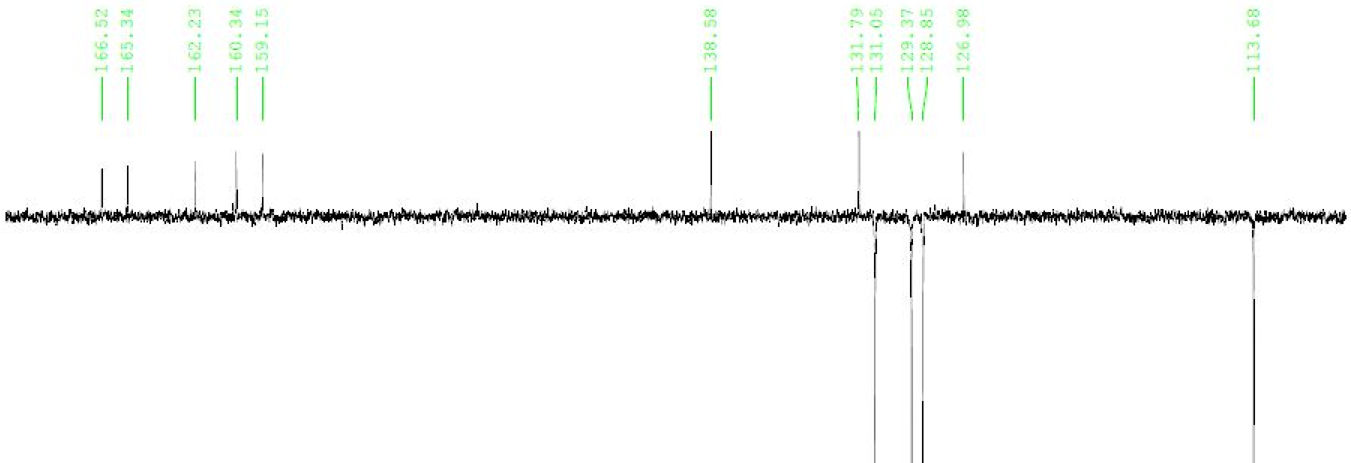

Current Data Parameters
NAME Kalogirou
EXPNO 787
PROCNO 1

F2 - Acquisition Parameters
Date_ 20200306
Time 15.34
INSTRUM spect
PROBHD 5 mm PABBO BB-
PULPROG jmod
TD 65536
SOLVENT CDCl3
NS 2749
DS 4
SWH 29761.904 Hz
FIDRES 0.454131 Hz

500 MHz

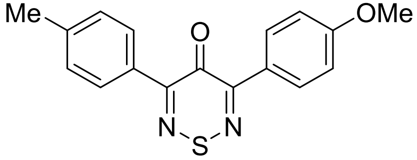

4ai

CDCl$_3$

3-(4-Methoxyphenyl)-5-(p-tolyl)-4H-1,2,6-thiadiazin-4-one (**43a**)

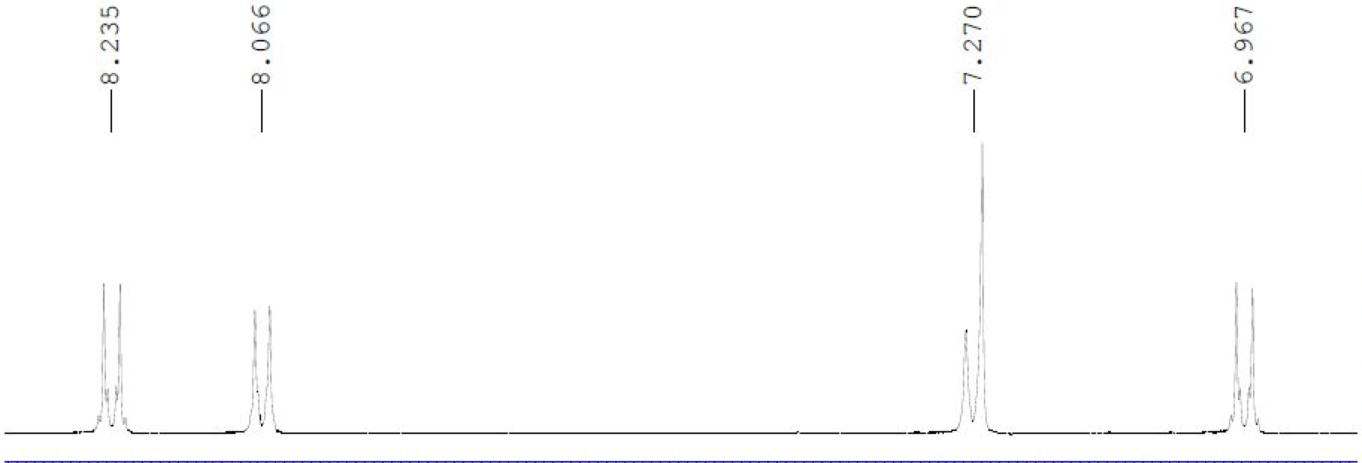


```
Current Data Parameters
NAME           Kalogirou
EXPNO                895
PROCNO                 1

F2 - Acquisition Parameters
Date_           20201029
Time               16.26
INSTRUM            spect
PROBHD   5 mm PABBO BB-
PULPROG             zg30
TD                 65536
SOLVENT            CDCl3
NS                    16
DS                     2
SWH            10000.000 Hz
FIDRES          0.152588 Hz
```

125 MHz

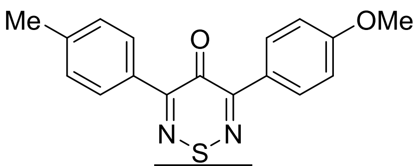

4ai

$CDCl_3$

3-(4-Methoxyphenyl)-5-(p-tolyl)-4H-1,2,6-thiadiazin-4-one (**43a**)

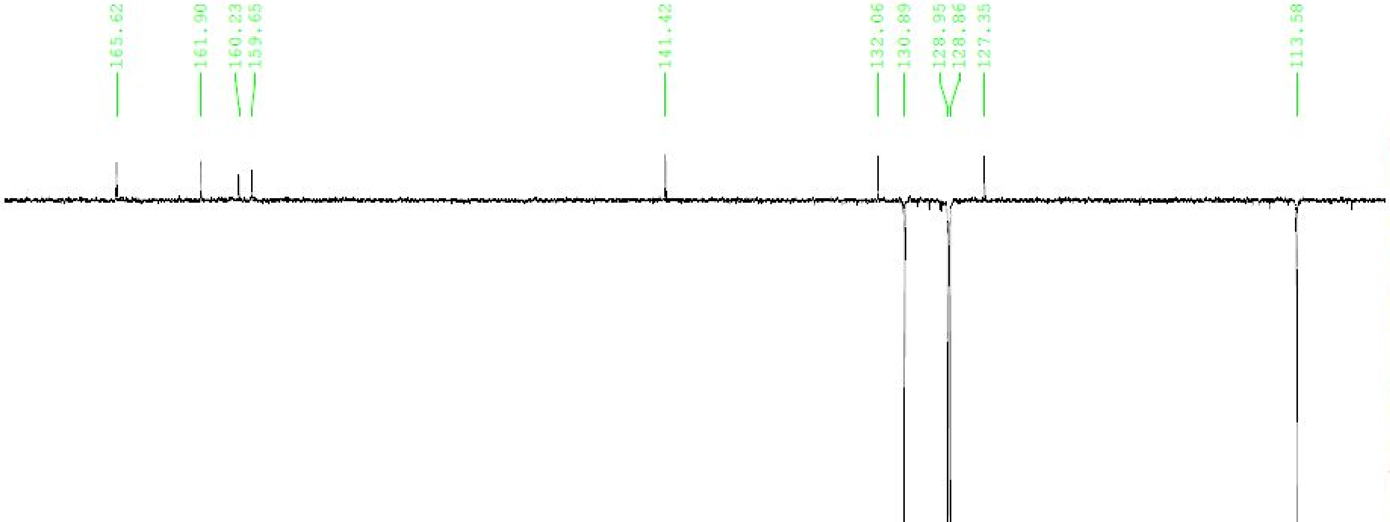

Current Data Parameters
NAME Kalogirou
EXPNO 896
PROCNO 1

F2 - Acquisition Parameters
Date_ 20201030
Time 2.01
INSTRUM spect
PROBHD 5 mm PABBO BB-
PULPROG jmod
TD 65536
SOLVENT CDCl3
NS 6800
DS 4
SWH 29761.904 Hz
FIDRES 0.454131 Hz
AQ 1.1010048 sec
RG 2050
DW 16.800 usec

500 MHz

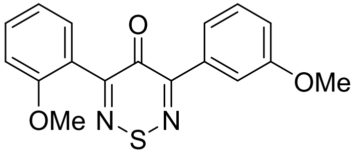

4aj

Acetone-d_6

3-(2-Methoxyphenyl)-5-(3-methoxyphenyl)-4H-1,2,6-thiadiazin-4-one (**44a**)

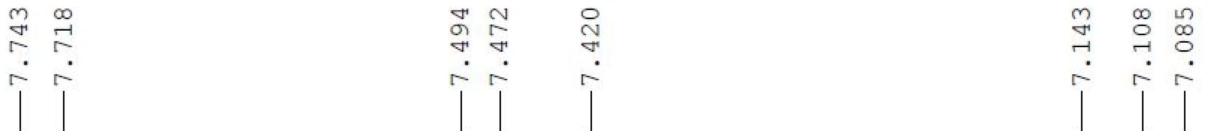

125 MHz

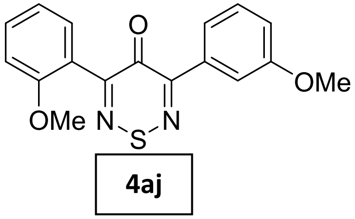

4aj

Acetone-d_6

Acetone-d_6

3-(2-Methoxyphenyl)-5-(3-methoxyphenyl)-4H-1,2,6-thiadiazin-4-one

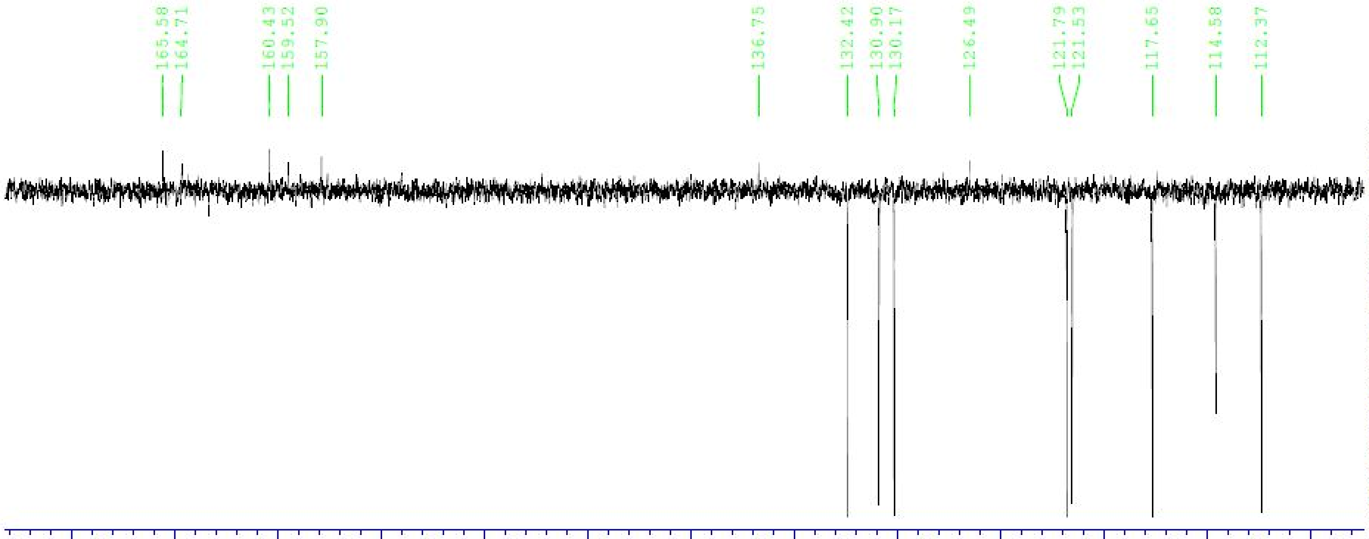

Current Data Parameters
NAME Kalogirou
EXPNO 913
PROCNO 1

F2 - Acquisition Parameters
Date_ 20201110
Time 17.52
INSTRUM spect
PROBHD 5 mm PABBO BB-
PULPROG jmod
TD 65536
SOLVENT Acetone
NS 2600
DS 4
SWH 29761.904 Hz
FIDRES 0.454131 Hz
AQ 1.1010048 sec
RG 2050
DW 16.800 usec
DE 6.50 usec

500 MHz

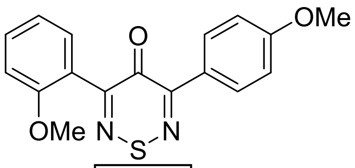

4ak

Acetone-d_6

3-(2-Methoxyphenyl)-5-(4-methoxyphenyl)-4H-1,2,6-thiadiazin-4-one (**45a**)

8.219
3.890
3.833
7.491
7.459
7.131
7.076
7.047

125 MHz

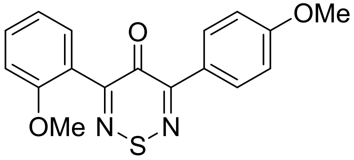

4ak

Acetone-d_6

Acetone-d_6

3-(2-Methoxyphenyl)-5-(4-methoxyphenyl)-4H-1,2,6-thiadiazin-4-one

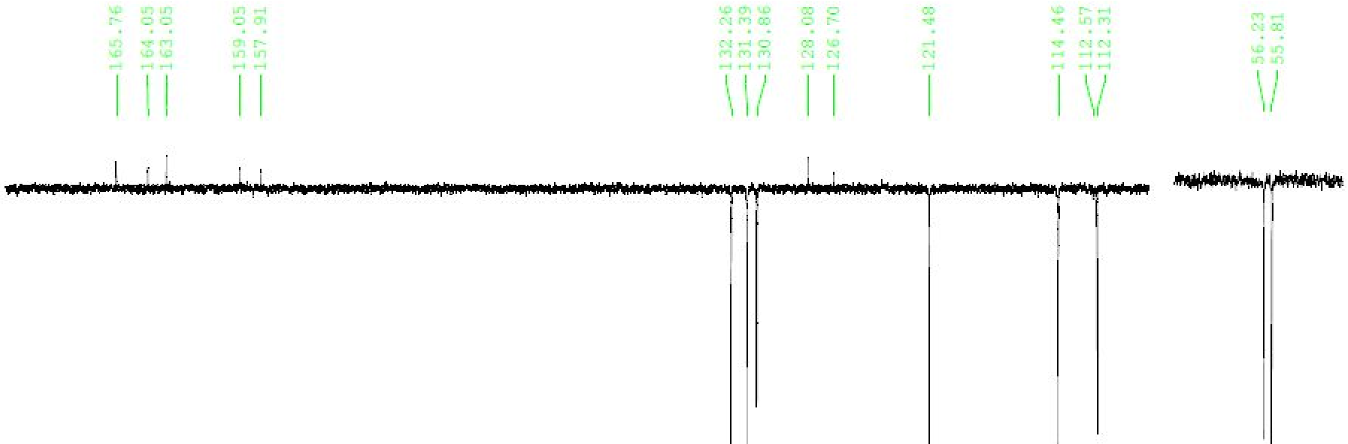

Current Data Parameters
NAME Kalogirou
EXPNO 909
PROCNO 1

F2 - Acquisition Parameters
Date_ 20201110
Time 11.24
INSTRUM spect
PROBHD 5 mm PABBO BB-
PULPROG jmod
TD 65536
SOLVENT Acetone
NS 3000
DS 4
SWH 29761.904 Hz
FIDRES [illegible]

500 MHz

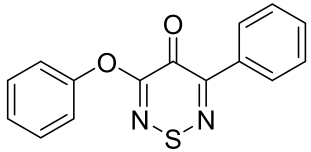

4ac

CDCl$_3$

3-Phenoxy-5-phenyl-4H-1,2,6-thiadiazin-4-one

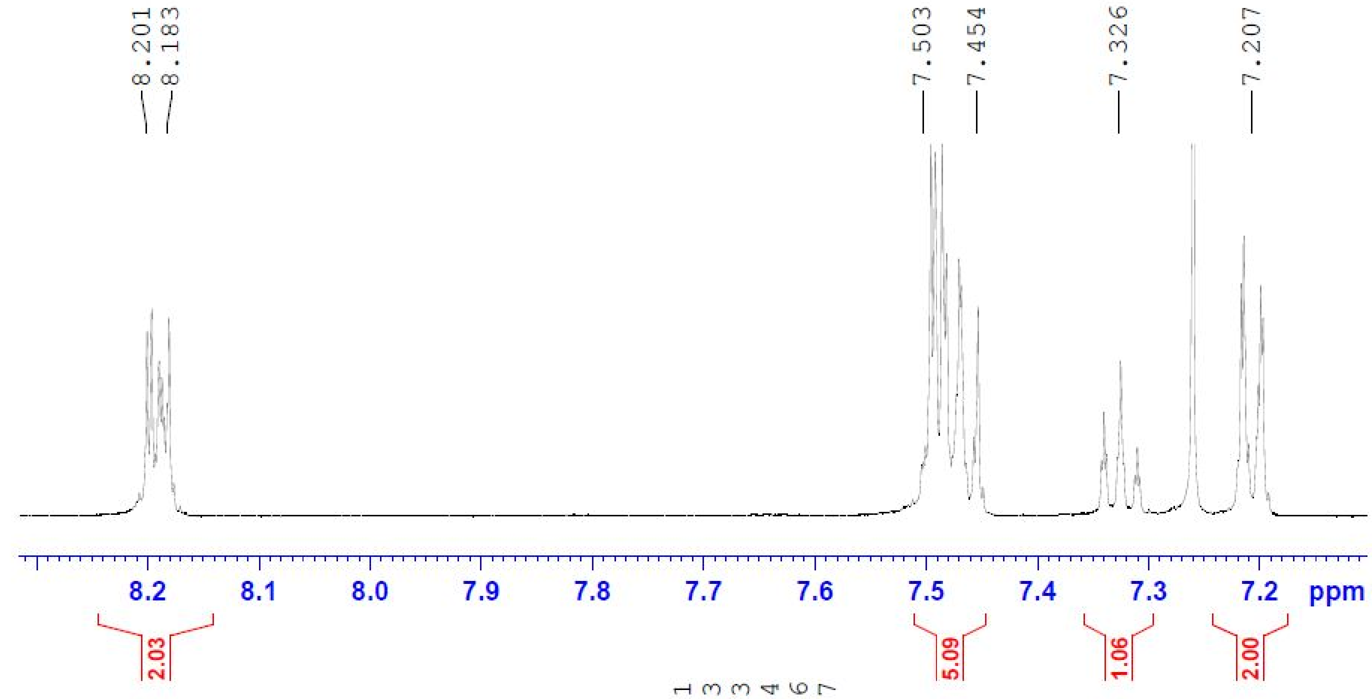

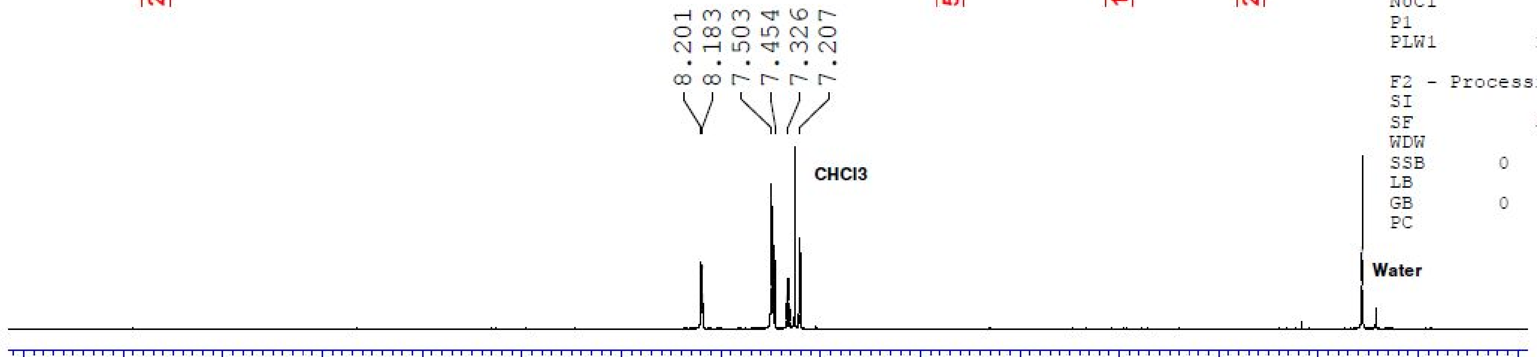


```
Current Data Parameters
NAME          Kalogirou
EXPNO               914
PROCNO                1

F2 - Acquisition Parameters
Date_          20201112
Time              17.51
INSTRUM           spect
PROBHD   5 mm PABBO BB-
PULPROG            zg30
TD                65536
SOLVENT           CDCl3
NS                   16
DS                    2
SWH           10000.000 Hz
FIDRES         0.152588 Hz
AQ            3.2767999 sec
RG                  181
DW               50.000 usec
DE                 6.50 usec
TE                294.9 K
D1           1.00000000 sec
TD0                   1

======== CHANNEL f1 ========
SFO1      500.0361158 MHz
NUC1                 1H
P1                12.00 usec
PLW1        14.50000000 W

F2 - Processing parameters
SI                65536
SF        500.0330400 MHz
WDW                  EM
SSB      0
LB                 0.30 Hz
GB       0
PC                 1.00
```

125 MHz

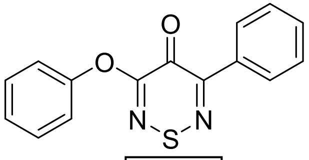

4ac

CDCl$_3$

3-Phenoxy-5-phenyl-4H-1,2,6-thiadiazin-4-one (**39a**)

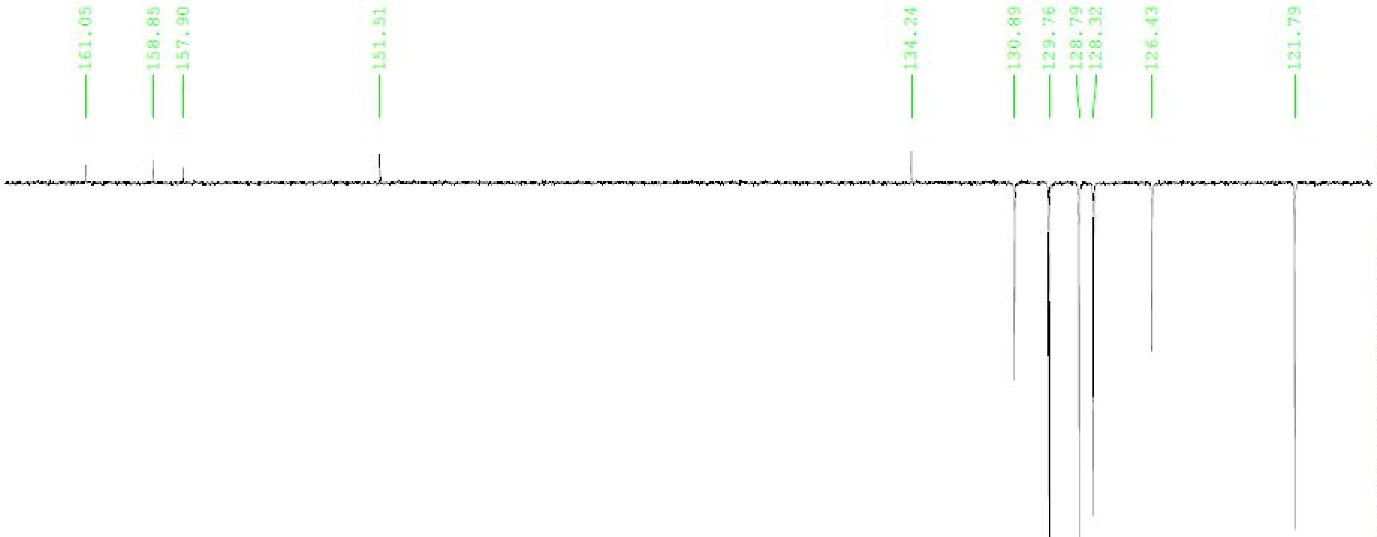

Current Data Parameters
NAME Kalogirou
EXPNO 915
PROCNO 1

F2 - Acquisition Parameters
Date_ 20201113
Time 8.51
INSTRUM spect
PROBHD 5 mm PABBO BB-
PULPROG jmod
TD 65536
SOLVENT CDCl3
NS 13500
DS 4
SWH 29761.904 Hz
FIDRES 0.454131 Hz
AQ 1.1010048 sec
RG 2050
DW 16.800 usec
DE 6.50 usec
[illegible]

500 MHz

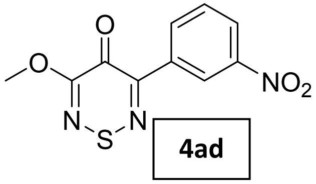

4ad

$CDCl_3$

3-Methoxy-5-(3-nitrophenyl)-4H-1,2,6-thiadiazin-4-one (**42a**)

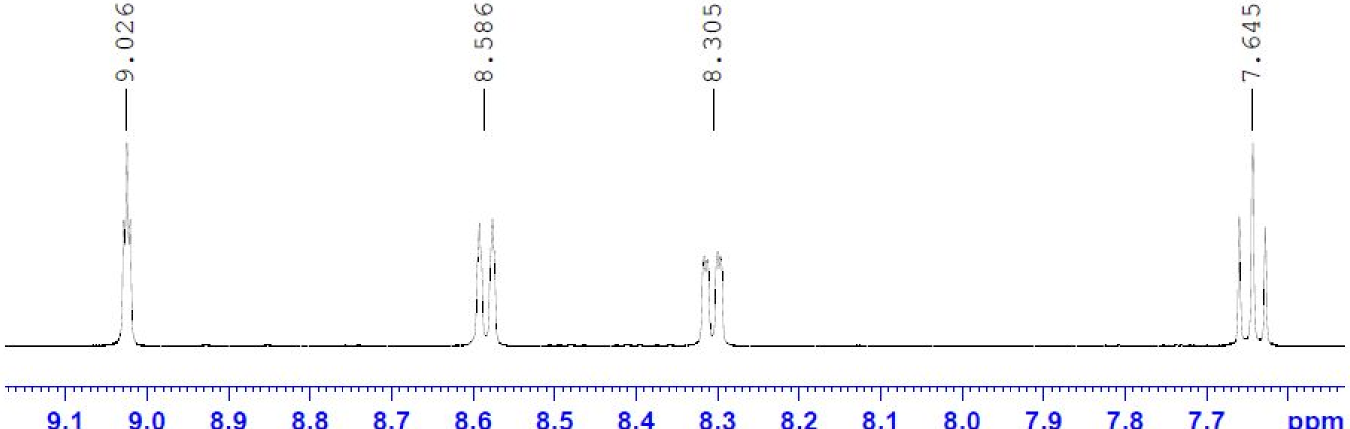


```
Current Data Parameters
NAME            Kalogirou
EXPNO                 918
PROCNO                  1

F2 - Acquisition Parameters
Date_            20201117
Time                14.18
INSTRUM             spect
PROBHD   5 mm PABBO BB-
PULPROG              zg30
TD                  65536
SOLVENT             CDCl3
NS                     16
DS                      2
```
[illegible]

125 MHz

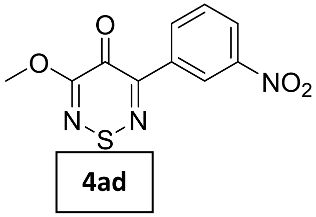

4ad

CDCl$_3$

3-Methoxy-5-(3-nitrophenyl)-4H-1,2,6-thiadiazin-4-one (**42a**)

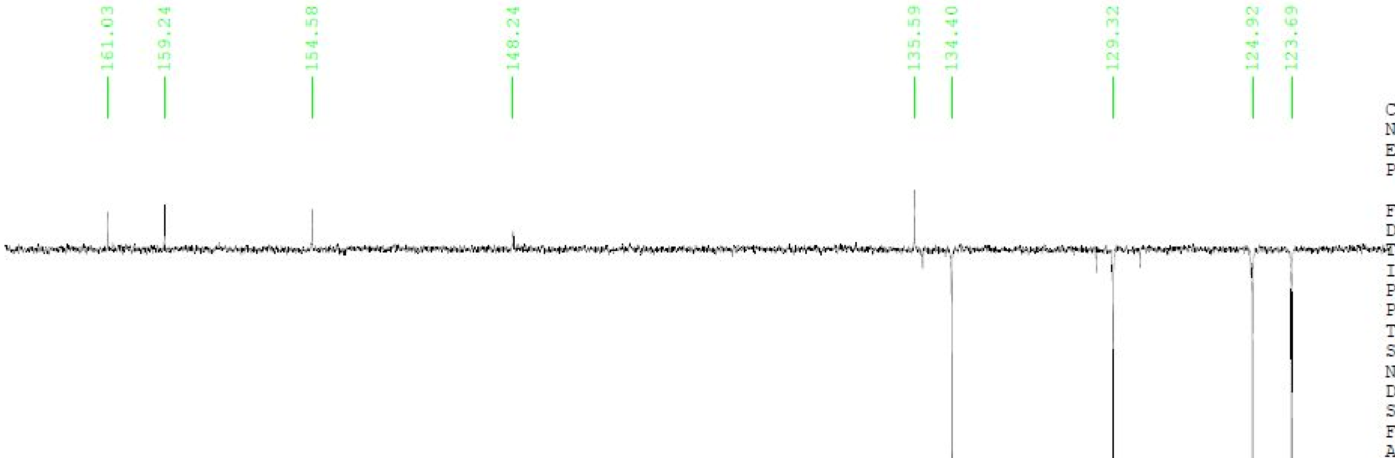

Current Data Parameters
NAME Kalogirou
EXPNO 922
PROCNO 1

F2 - Acquisition Parameters
Date_ 20201118
Time 2.45
INSTRUM spect
PROBHD 5 mm PABBO BB-
PULPROG jmod
TD 65536
SOLVENT CDCl3
NS 7500
DS 4
SWH 29761.904 Hz
FIDRES 0.454131 Hz
AQ 1.1010048 sec

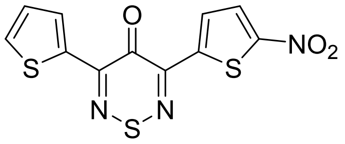

4ae

$CDCl_3$

400 MHz

3-(5-nitrothiophen-2-yl)-5-(thiophen-2-yl)-4H-1,2,6-thiadiazin-4-one (**41a**)

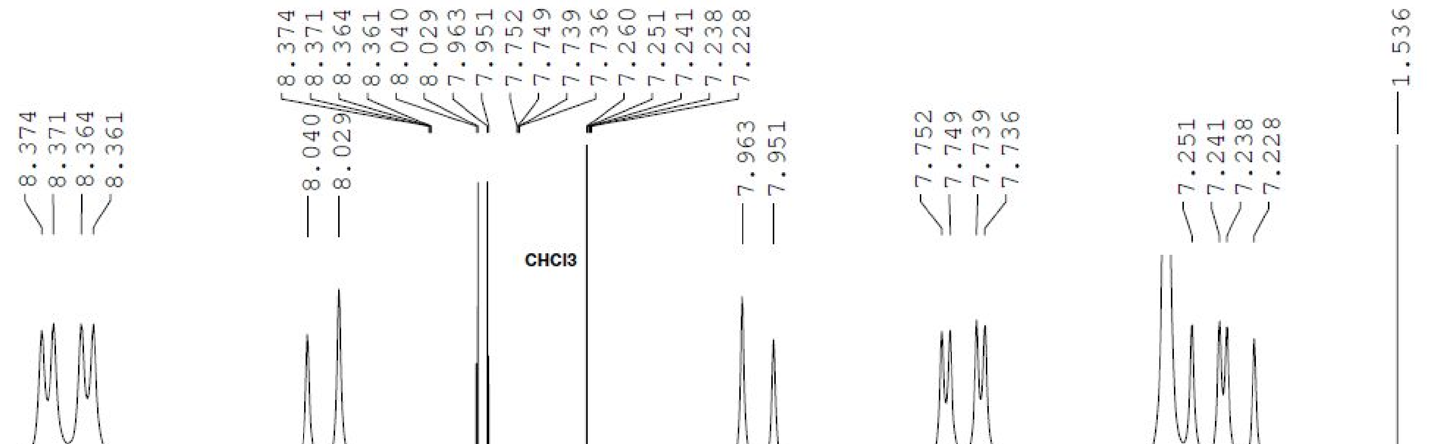

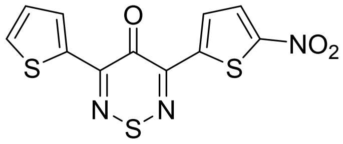

4ae

$CDCl_3$

100 MHz

3-(5-nitrothiophen-2-yl)-5-(thiophen-2-yl)-4H-1,2,6-thiadiazin-4-one (**41a**)

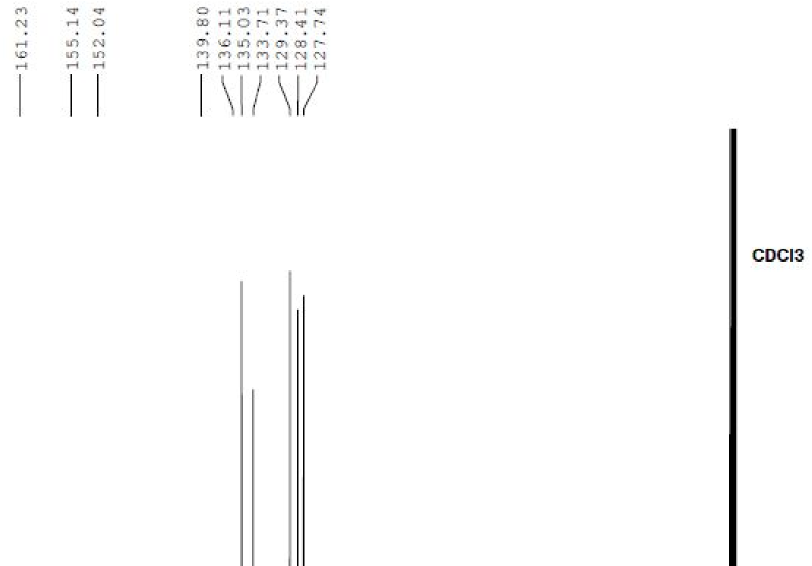

500 MHz

$CDCl_3$

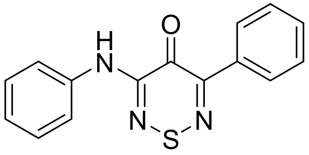

4af

3-Phenyl-5-(phenylamino)-4H-1,2,6-thiadiazin-4-one (**35a**)

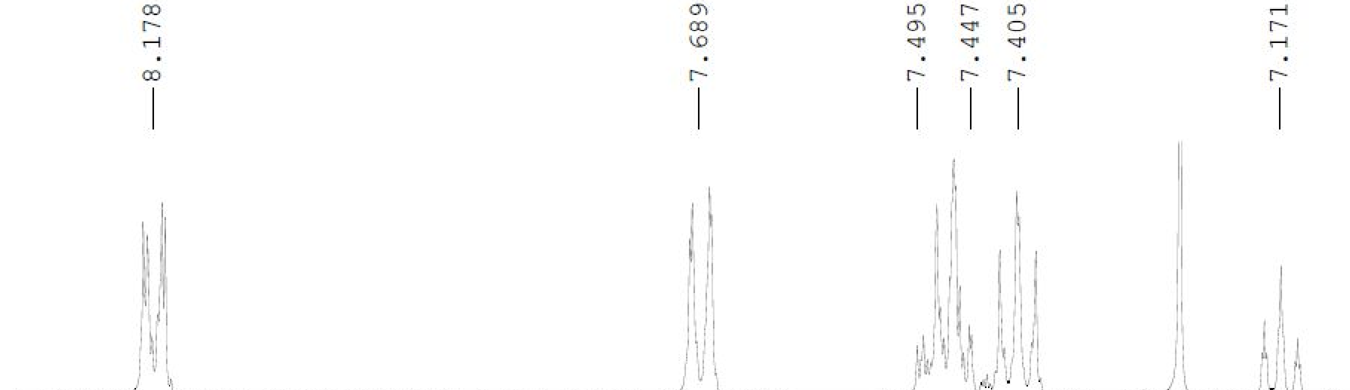


```
Current Data Parameters
NAME           Kalogirou
EXPNO                919
PROCNO                 1

F2 - Acquisition Parameters
Date_           20201117
Time               14.23
INSTRUM            spect
PROBHD   5 mm PABBO BB-
PULPROG             zg30
TD                 65536
SOLVENT            CDCl3
```

125 MHz

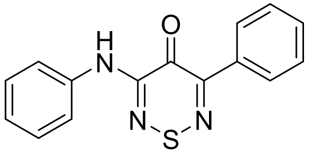

4af

$CDCl_3$

3-Phenyl-5-(phenylamino)-4H-1,2,6-thiadiazin-4-one (**35a**)

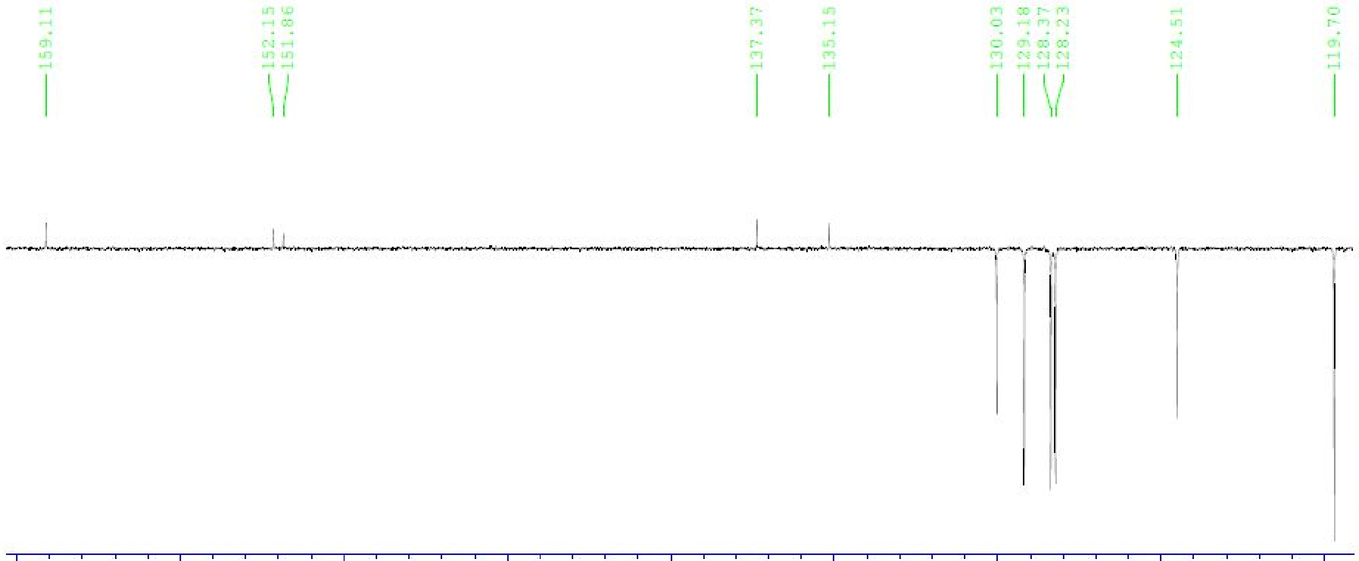

Current Data Parameters
NAME Kalogirou
EXPNO 923
PROCNO 1

F2 - Acquisition Parameters
Date_ 20201118
Time 9.22
INSTRUM spect
PROBHD 5 mm PABBO BB-
PULPROG jmod
TD 65536
SOLVENT CDCl3
NS 7500
DS 4
SWH 29761.904 Hz
FIDRES 0.454131 Hz
AQ 1.1010048 sec
RG 2050
DW 16.800 usec
DE 6.50 usec
TE 297.2 K

300 MHz

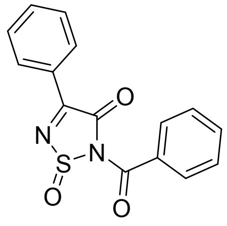

5a

CDCl$_3$

2-benzoyl-4-phenyl-1,2,5-thiadiazol-3(2H)-one 1-oxide(**1b**)

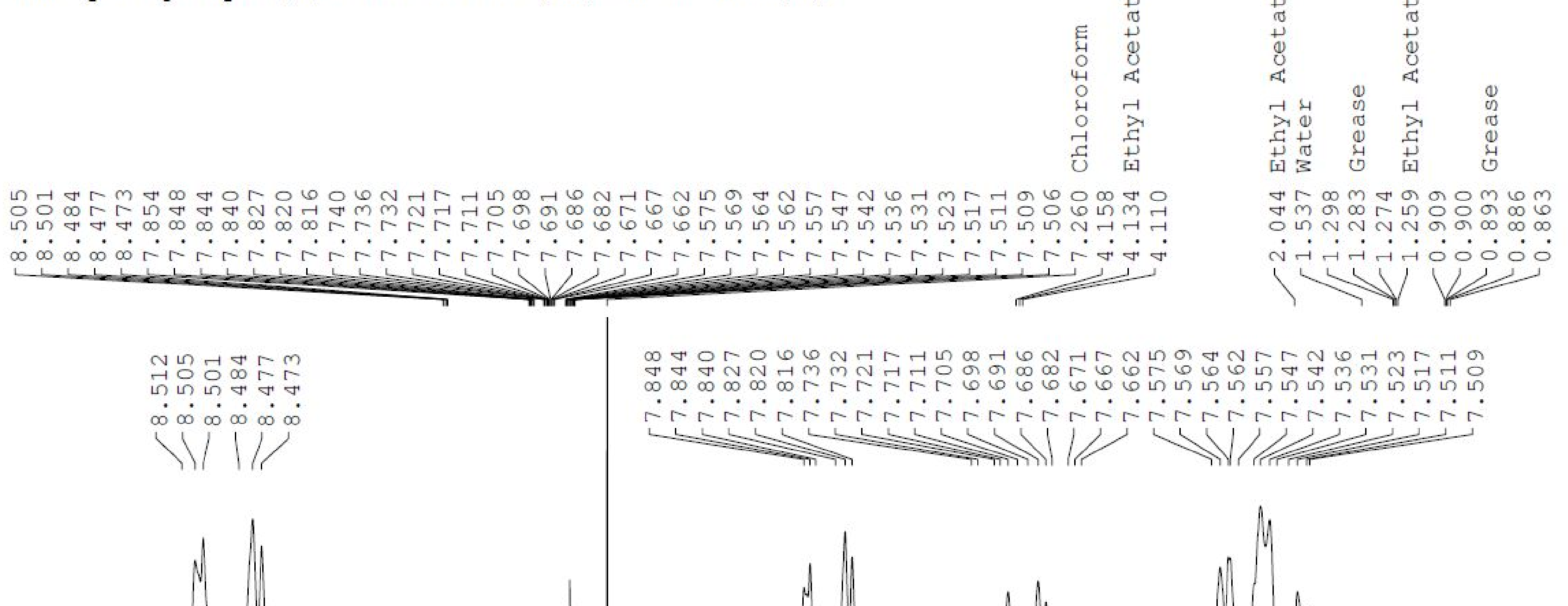

100 MHz

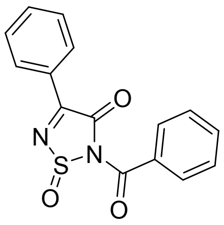

5a

$CDCl_3$

2-benzoyl-4-phenyl-1,2,5-thiadiazol-3(2H)-one 1-oxide(**1b**)

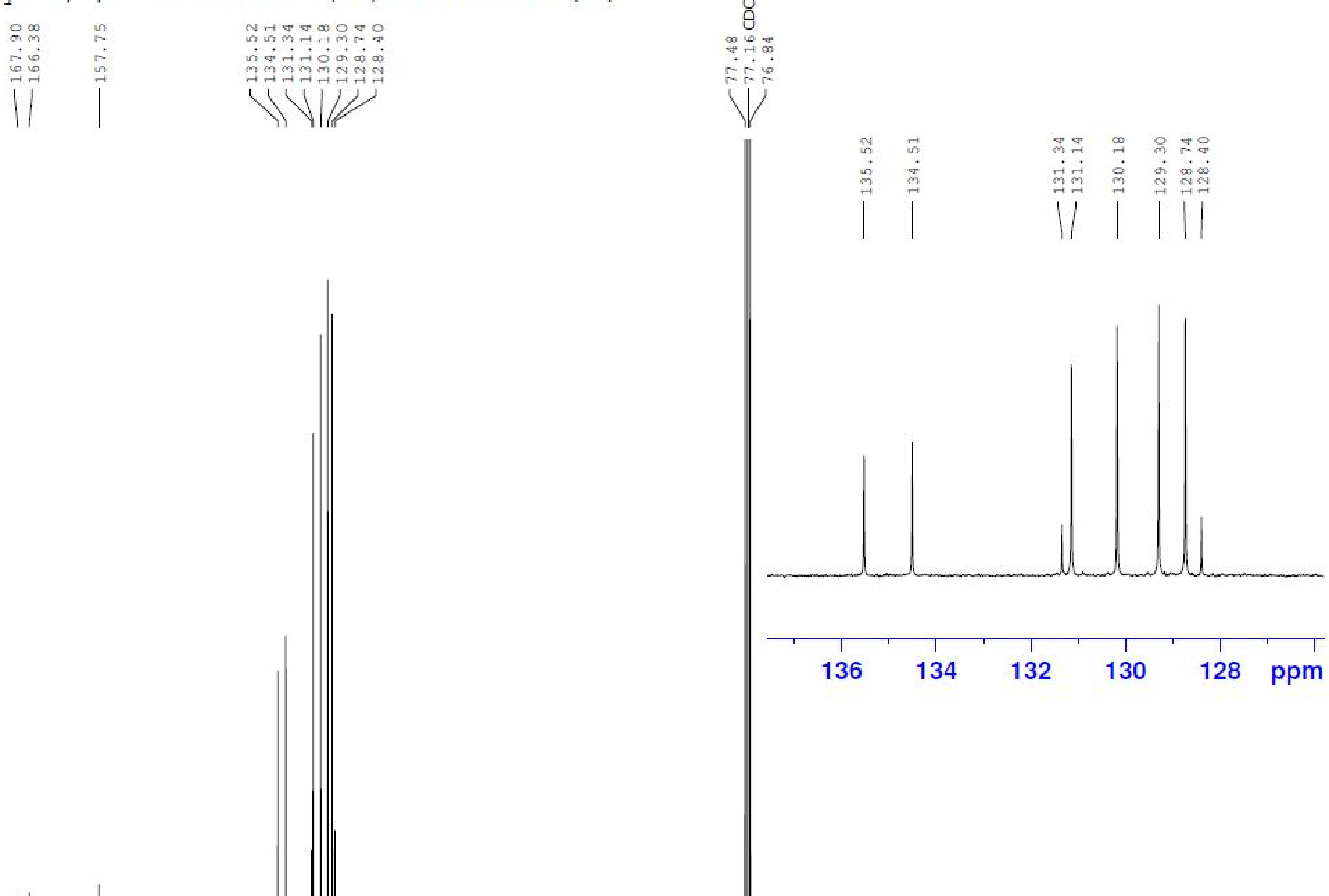

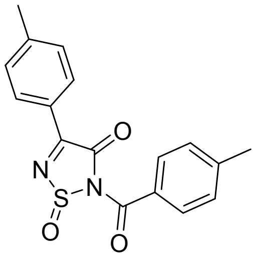

400 MHz

5d

CDCl$_3$

2-(4-methylbenzoyl)-4-(p-tolyl)-1,2,5-thiadiazol-3(2H)-one 1-oxide(**13b**)

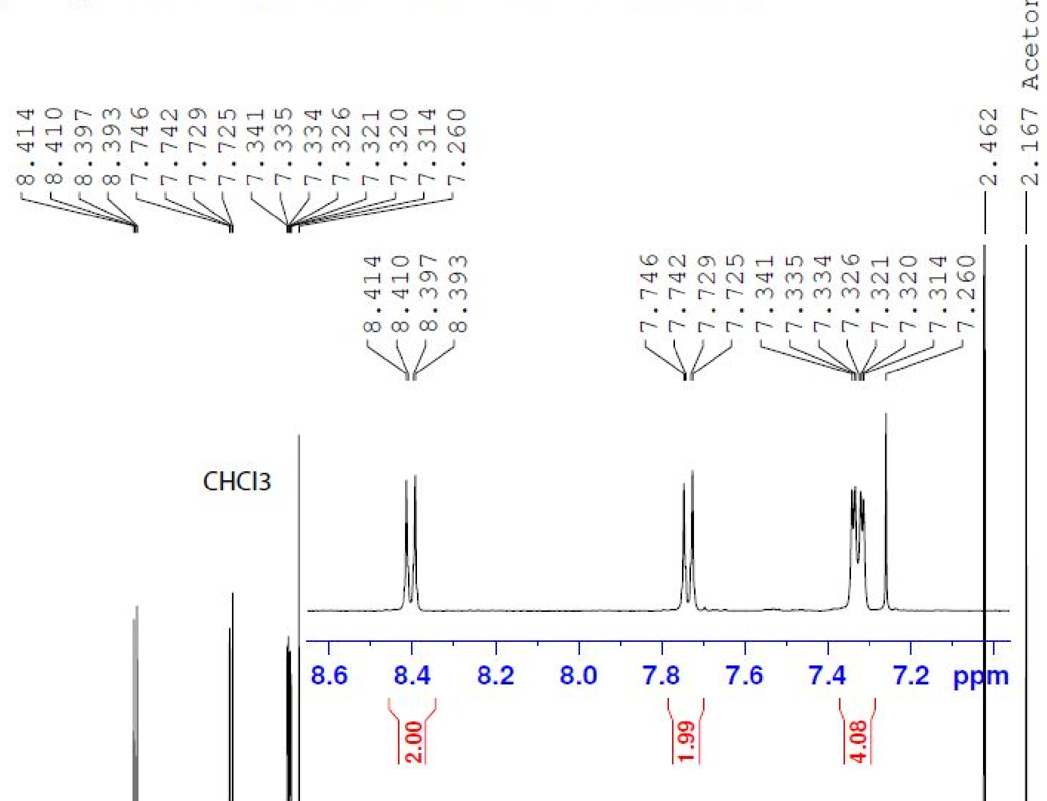

100 MHz

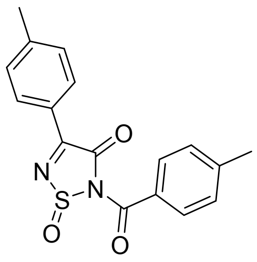

CDCl$_3$

5d

2-(4-methylbenzoyl)-4-(p-tolyl)-1,2,5-thiadiazol-3(2H)-one 1-oxide **(13b)**

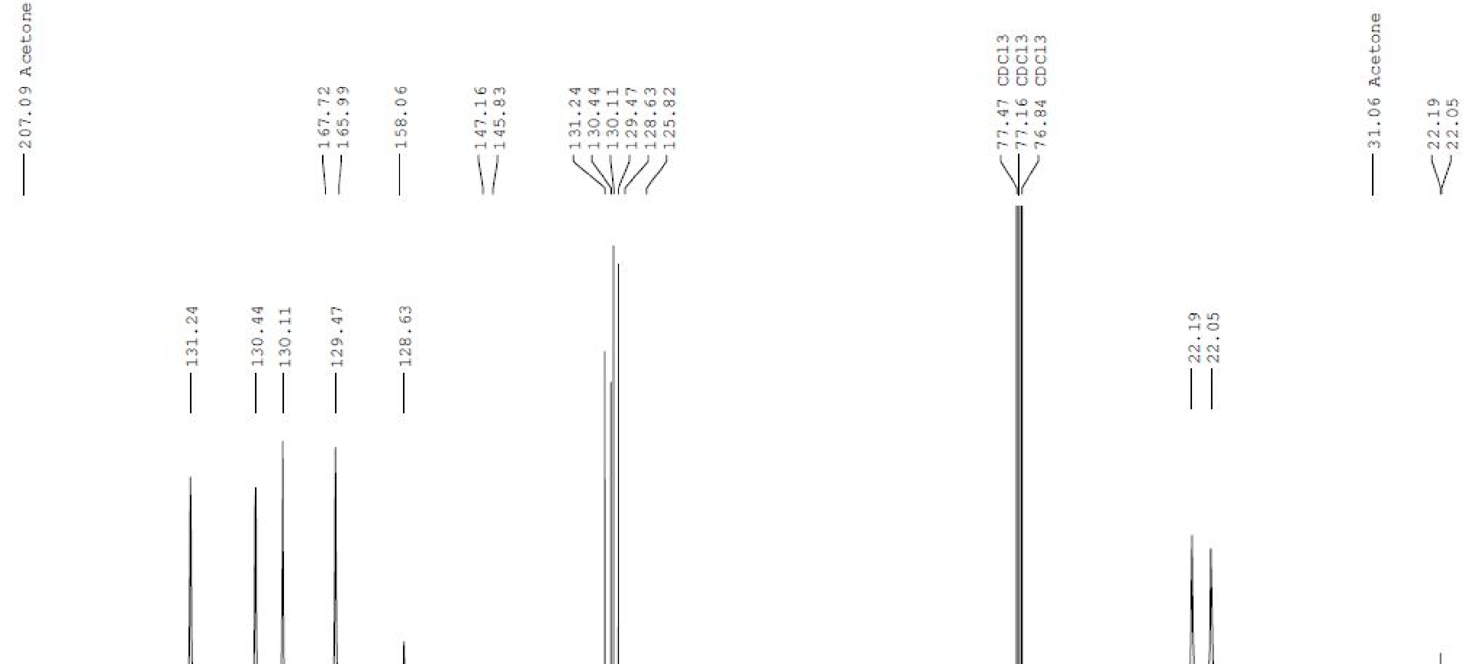

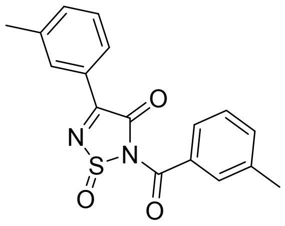

5c

400 MHz

$CDCl_3$

2-(3-methylbenzoyl)-4-(m-tolyl)-1,2,5-thiadiazol-3(2H)-one 1-oxide (**14b**)

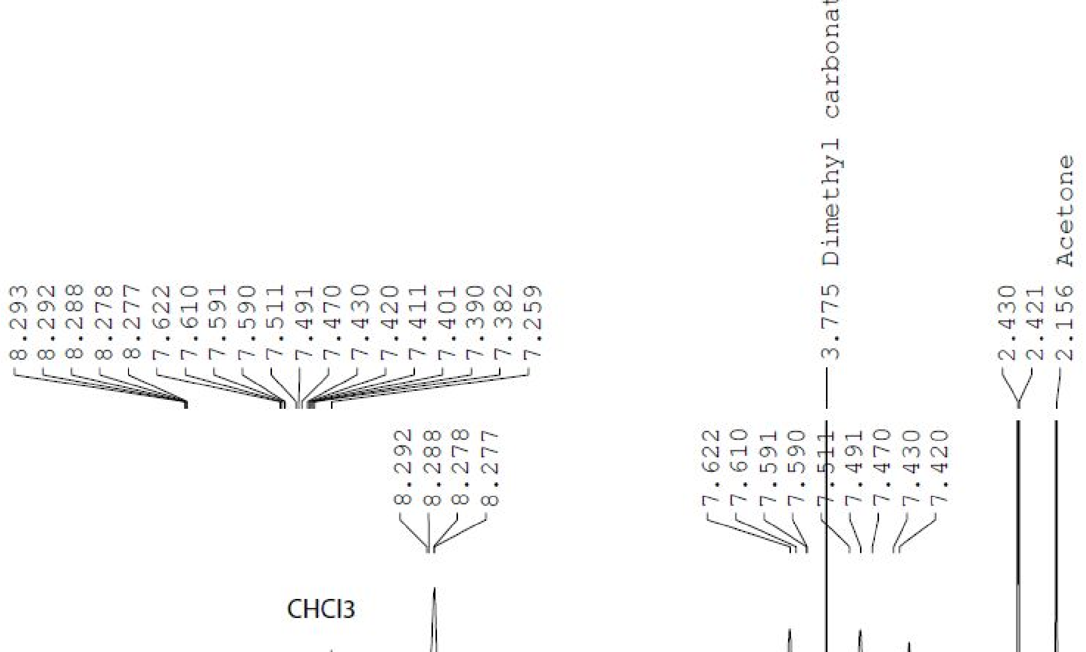

CDCl$_3$

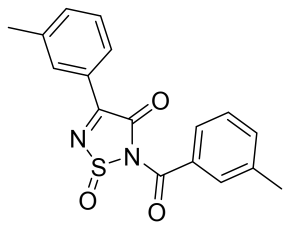

5c

100 MHz

2-(3-methylbenzoyl)-4-(m-tolyl)-1,2,5-thiadiazol-3(2H)-one 1-oxide (**14b**)

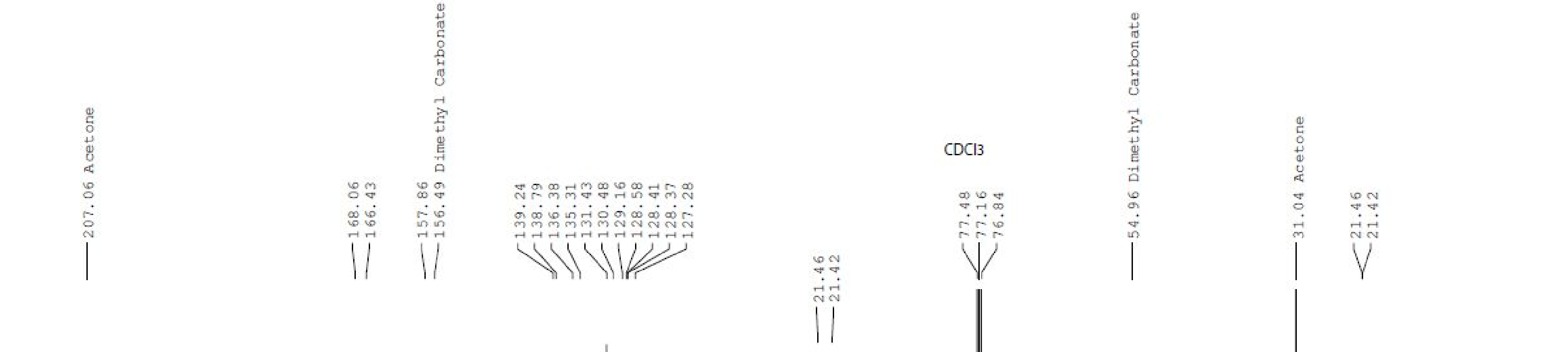

400 MHz

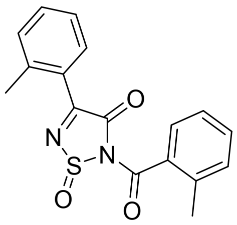

5b

CDCl$_3$

2-(2-methylbenzoyl)-4-(o-tolyl)-1,2,5-thiadiazol-3(2H)-one 1-oxide(**15b**)

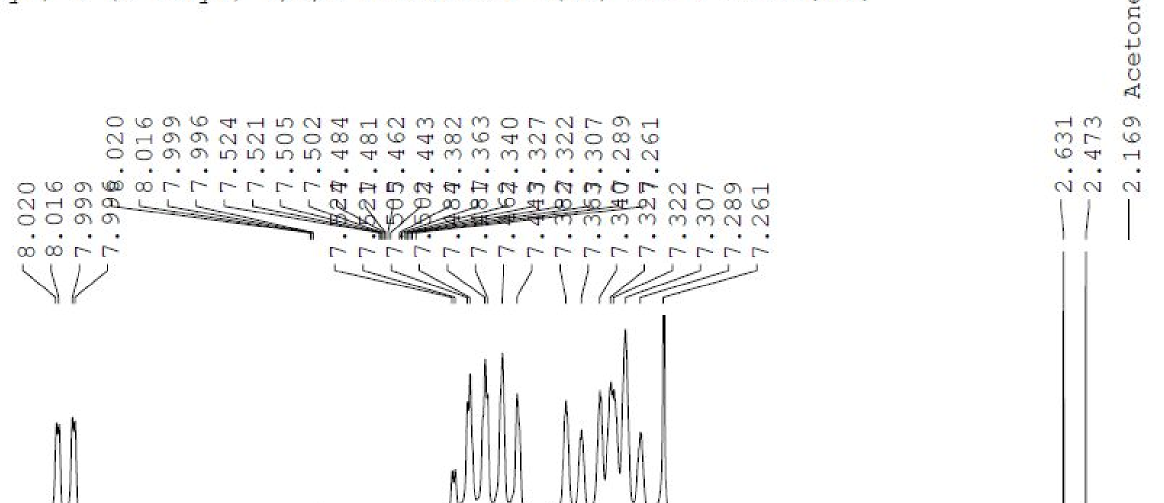

100 MHz

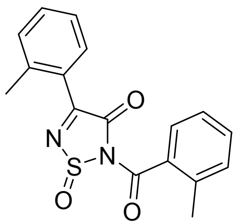

5b

$CDCl_3$

2-(2-methylbenzoyl)-4-(o-tolyl)-1,2,5-thiadiazol-3(2H)-one 1-oxide

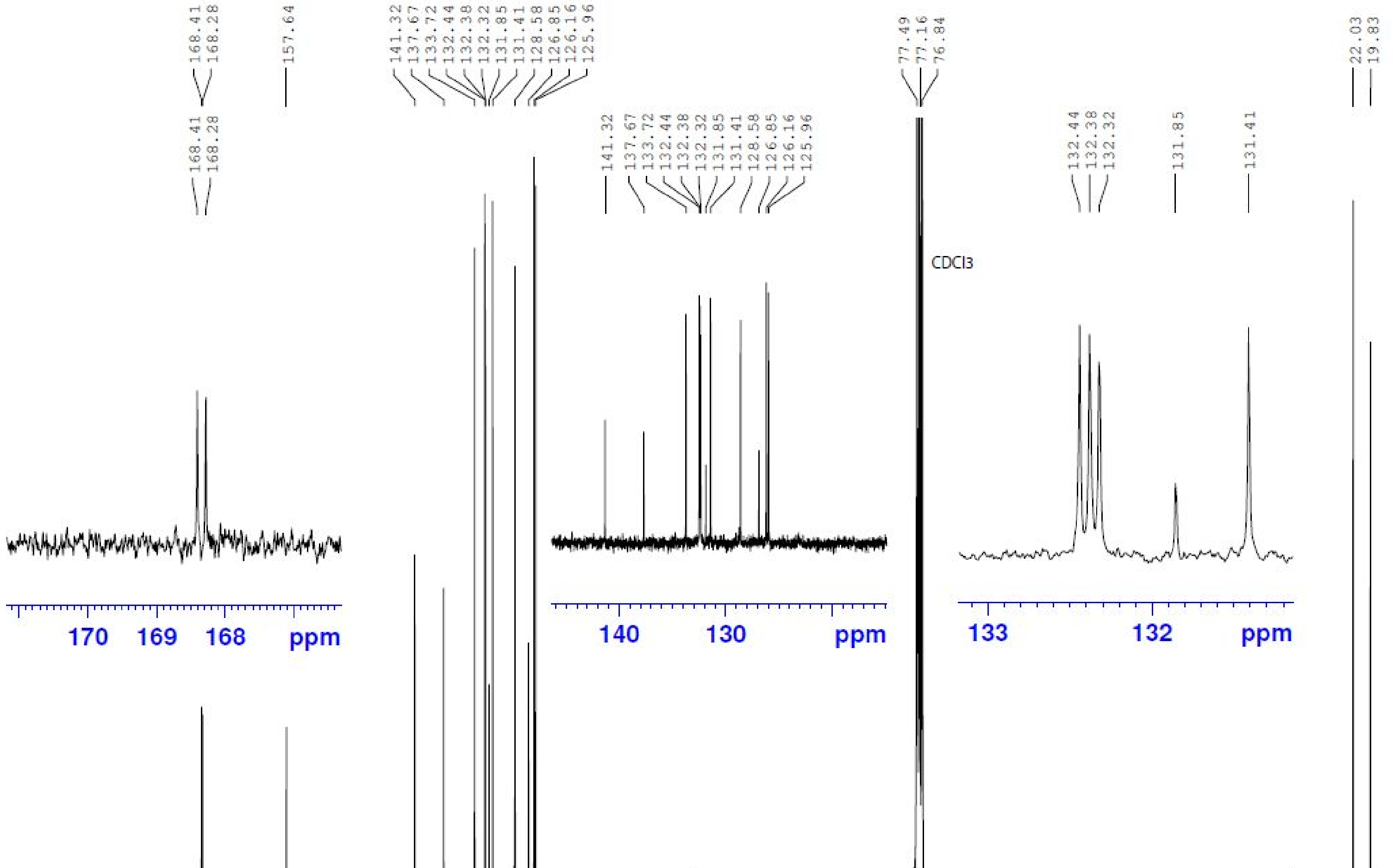

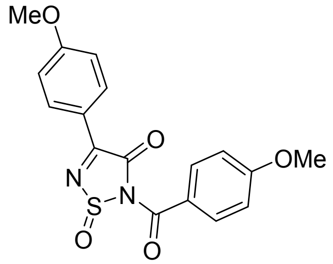

5g

CDCl$_3$

400 MHz

2-(4-methoxybenzoyl)-4-(4-methoxyphenyl)-1,2,5-thiadiazol-3(2H)-one 1-oxide (**16b**)

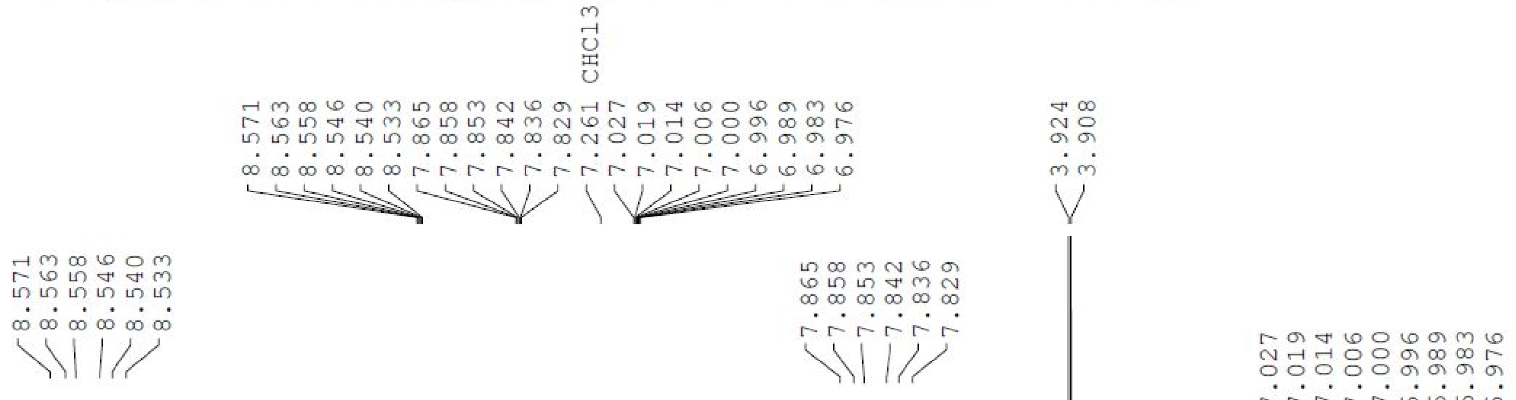

CDCl$_3$

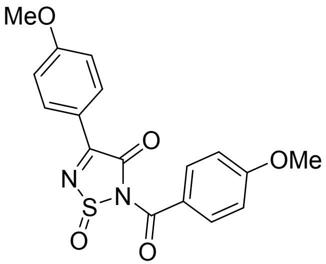

5g

100 MHz

2-(4-methoxybenzoyl)-4-(4-methoxyphenyl)-1,2,5-thiadiazol-3(2H)-one 1-oxide(**16b**)

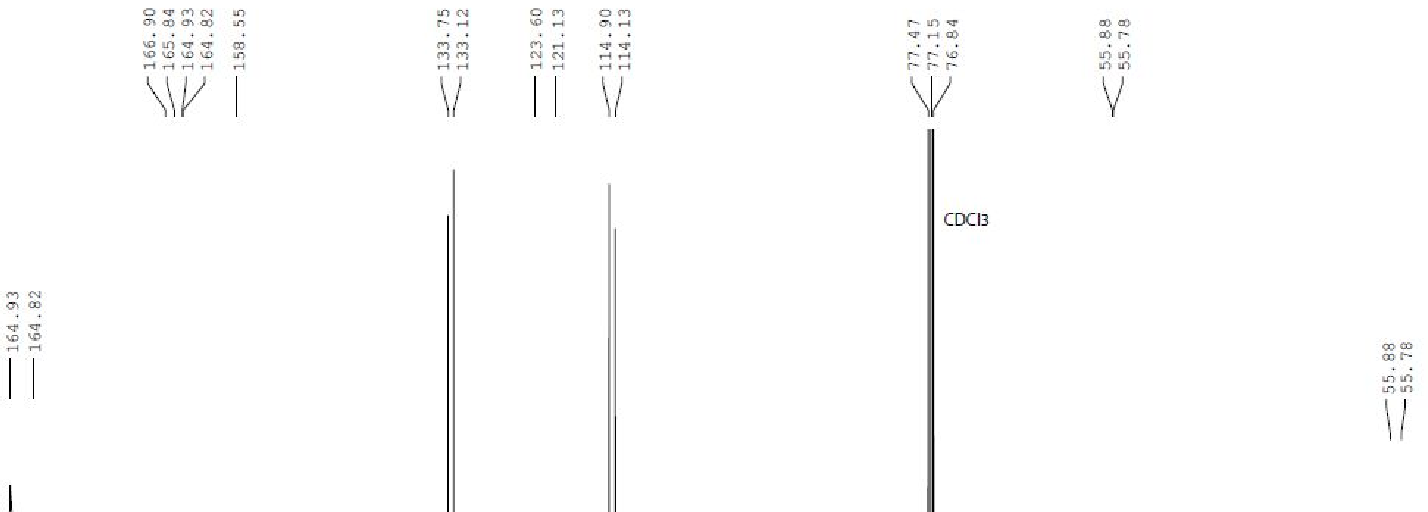

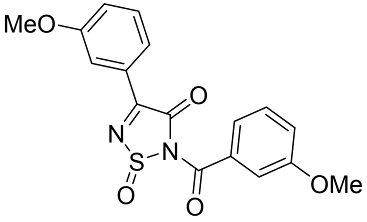

5f

CDCl$_3$

400 MHz

2-(3-methoxybenzoyl)-4-(3-methoxyphenyl)-1,2,5-thiadiazol-3(2H)-one 1-oxide (**17b**)

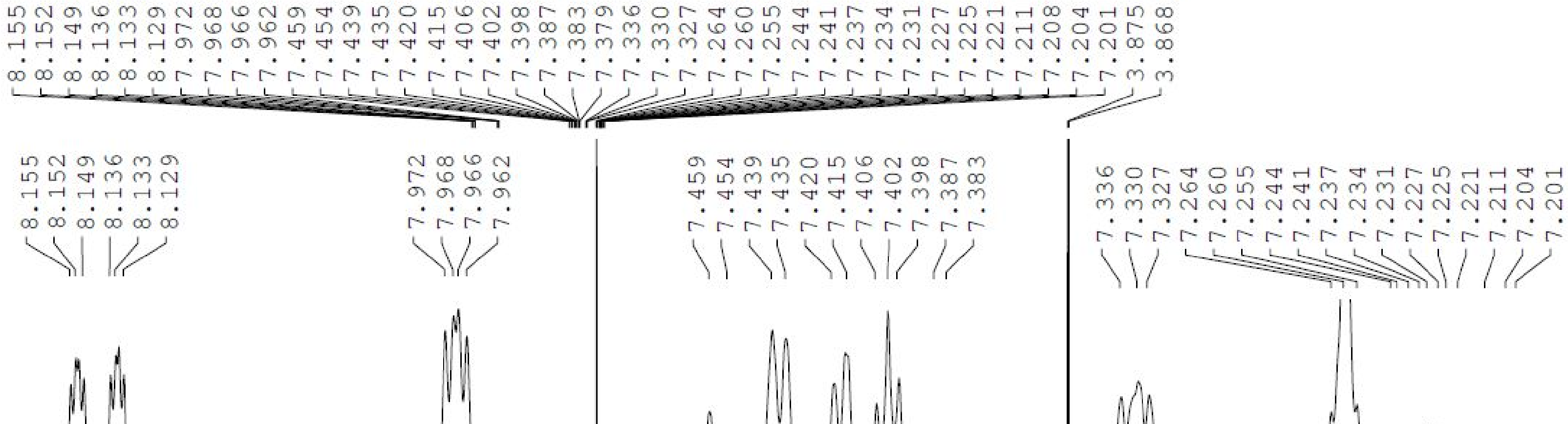

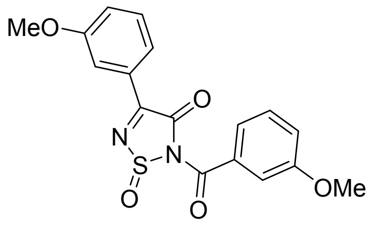

5f

$CDCl_3$

100 MHz

2-(3-methoxybenzoyl)-4-(3-methoxyphenyl)-1,2,5-thiadiazol-3(2H)-one 1-oxide **(17b)**

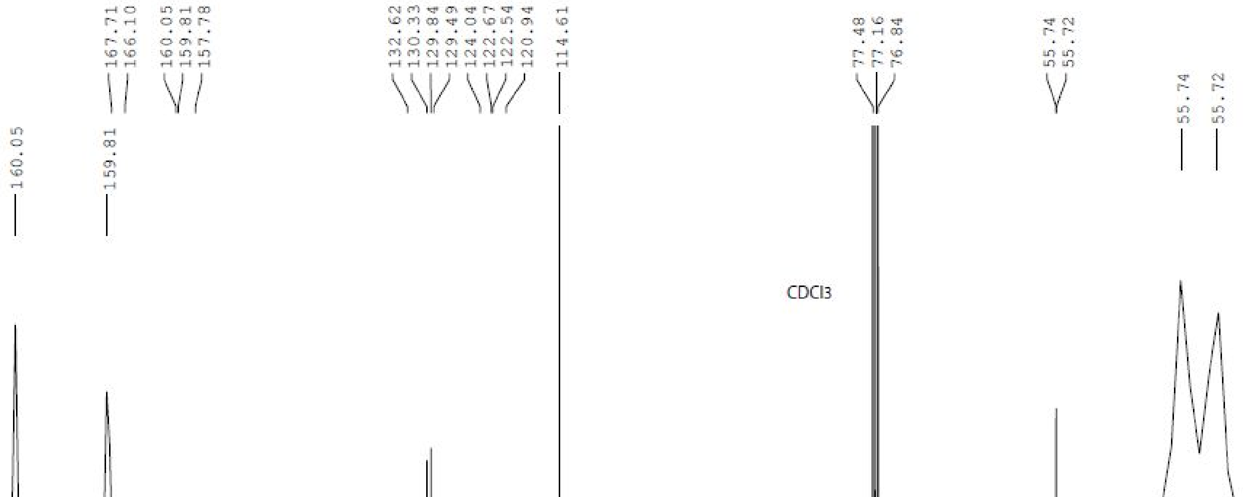

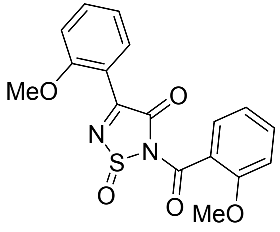

5e

CDCl$_3$

400 MHz

2-(2-methoxybenzoyl)-4-(2-methoxyphenyl)-1,2,5-thiadiazol-3(2H)-one 1-oxide **(18b)**

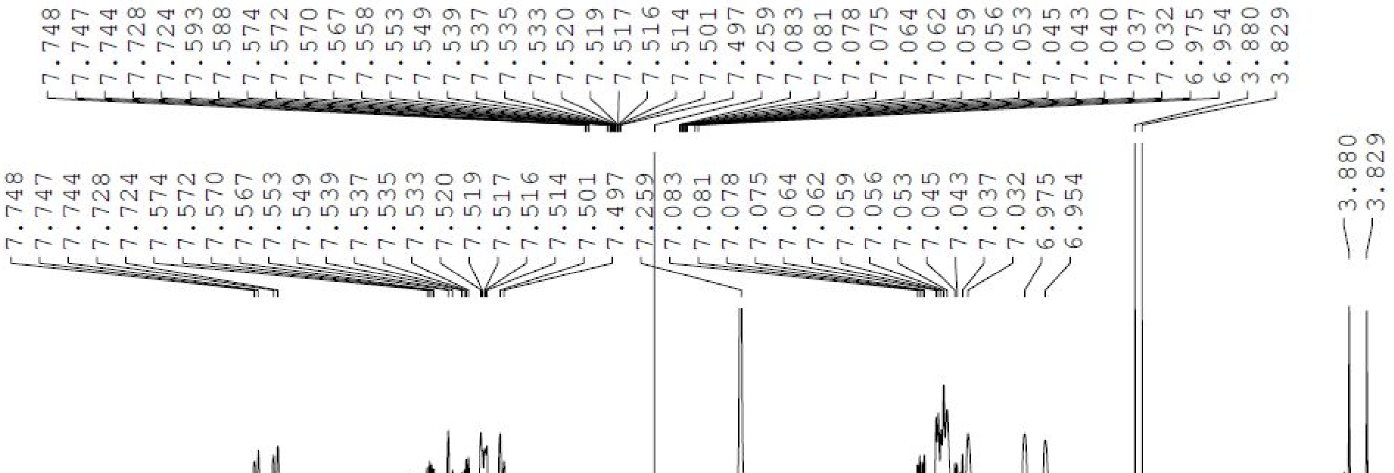

CDCl$_3$

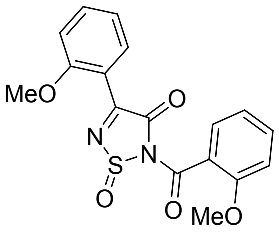

5e

100 MHz

2-(2-methoxybenzoyl)-4-(2-methoxyphenyl)-1,2,5-thiadiazol-3(2H)-one 1-oxide (**18b**)

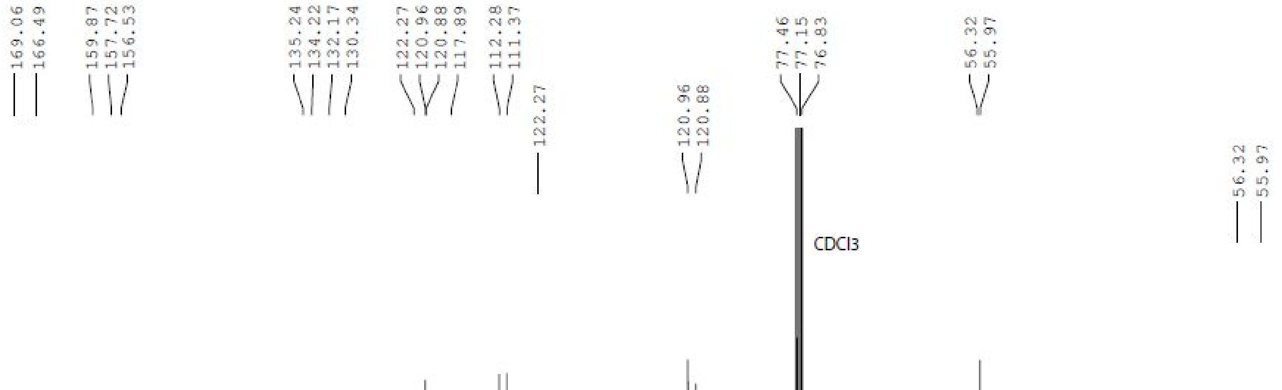

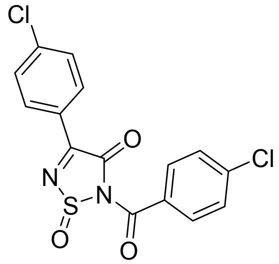

5j

CDCl$_3$

400 MHz

2-(4-chlorobenzoyl)-4-(4-chlorophenyl)-1,2,5-thiadiazol-3(2H)-one 1-oxide(**19b**)

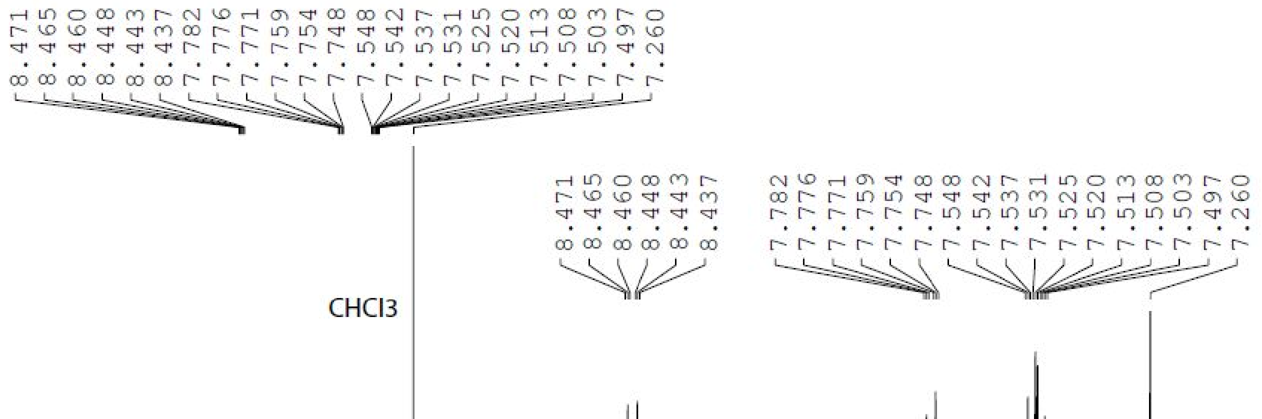

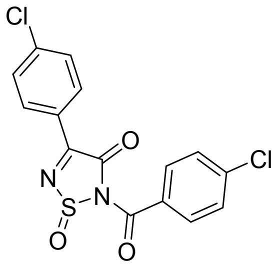

5j

CDCl$_3$

100 MHz

2-(4-chlorobenzoyl)-4-(4-chlorophenyl)-1,2,5-thiadiazol-3(2H)-one 1-oxide **(19b)**

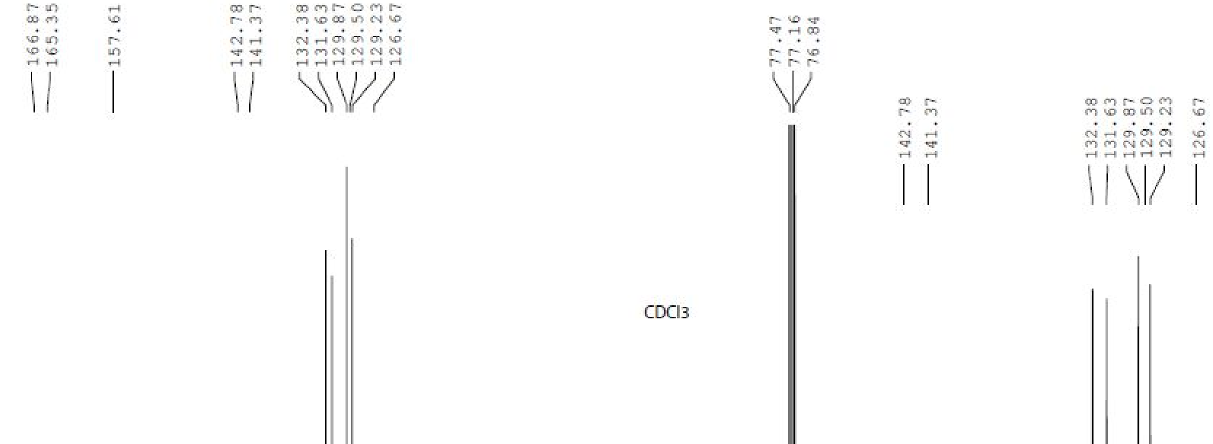

400 MHz

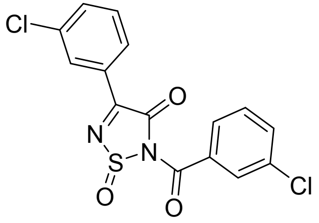

5i

$CDCl_3$

2-(3-chlorobenzoyl)-4-(3-chlorophenyl)-1,2,5-thiadiazol-3(2H)-one 1-oxide (20b)

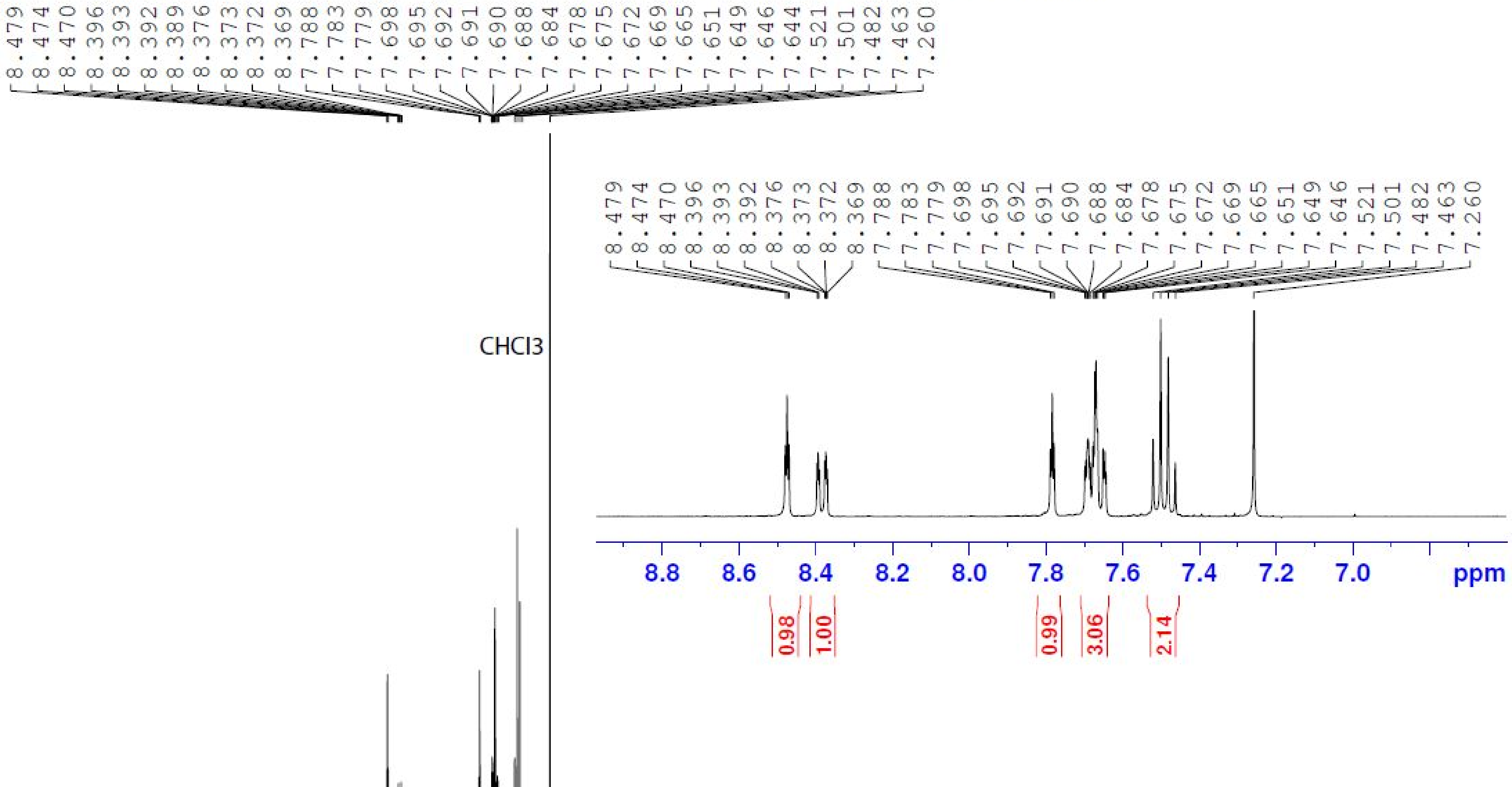

100 MHz

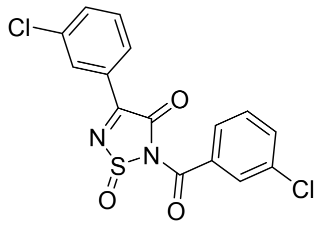

CDCl$_3$

5i

2-(3-chlorobenzoyl)-4-(3-chlorophenyl)-1,2,5-thiadiazol-3(2H)-one 1-oxide (**20b**)

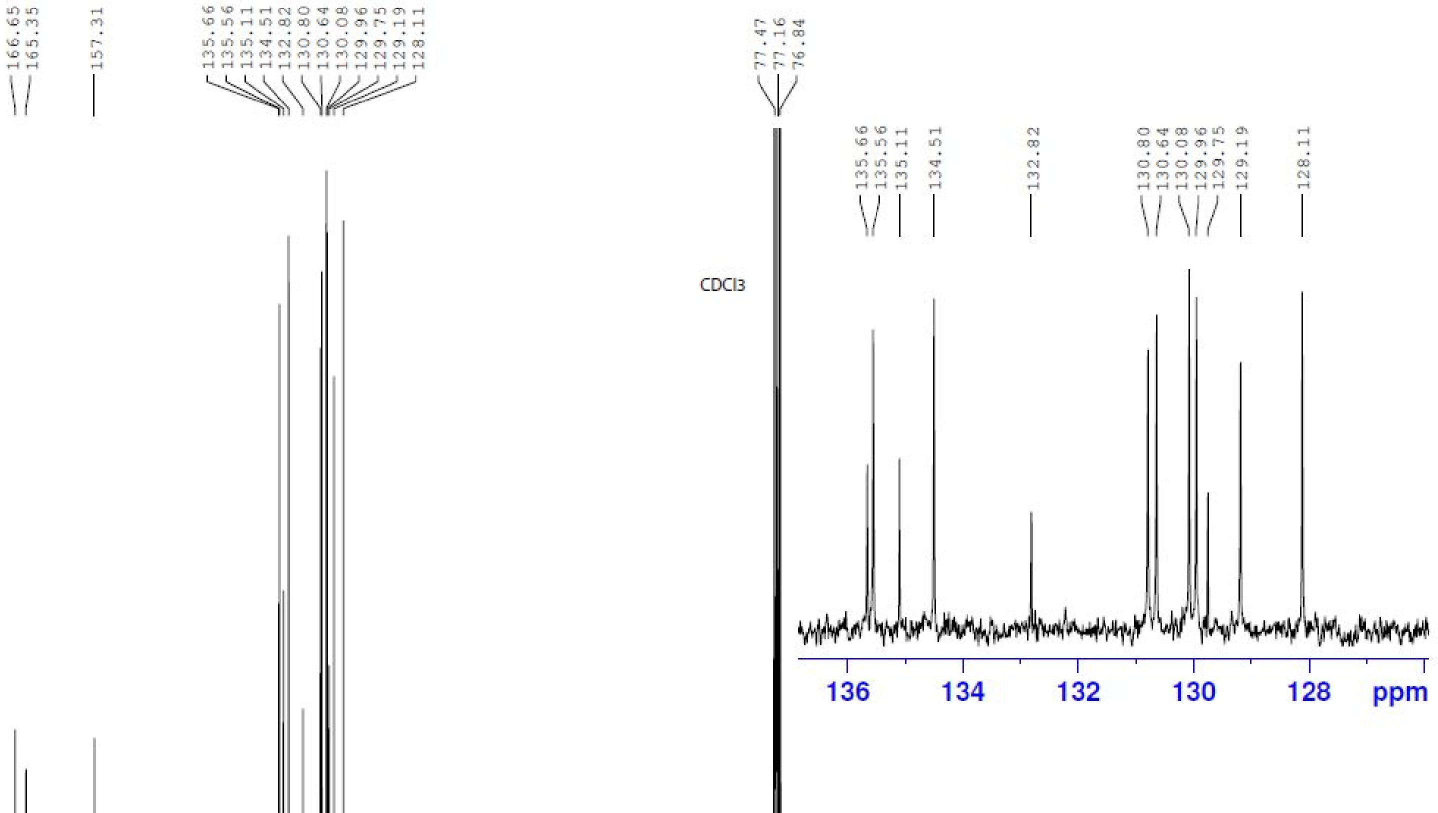

400 MHz

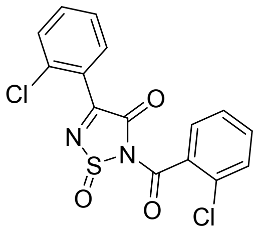

CDCl$_3$

5h

2-(2-chlorobenzoyl)-4-(2-chlorophenyl)-1,2,5-thiadiazol-3(2H)-one 1-oxide (**21b**)

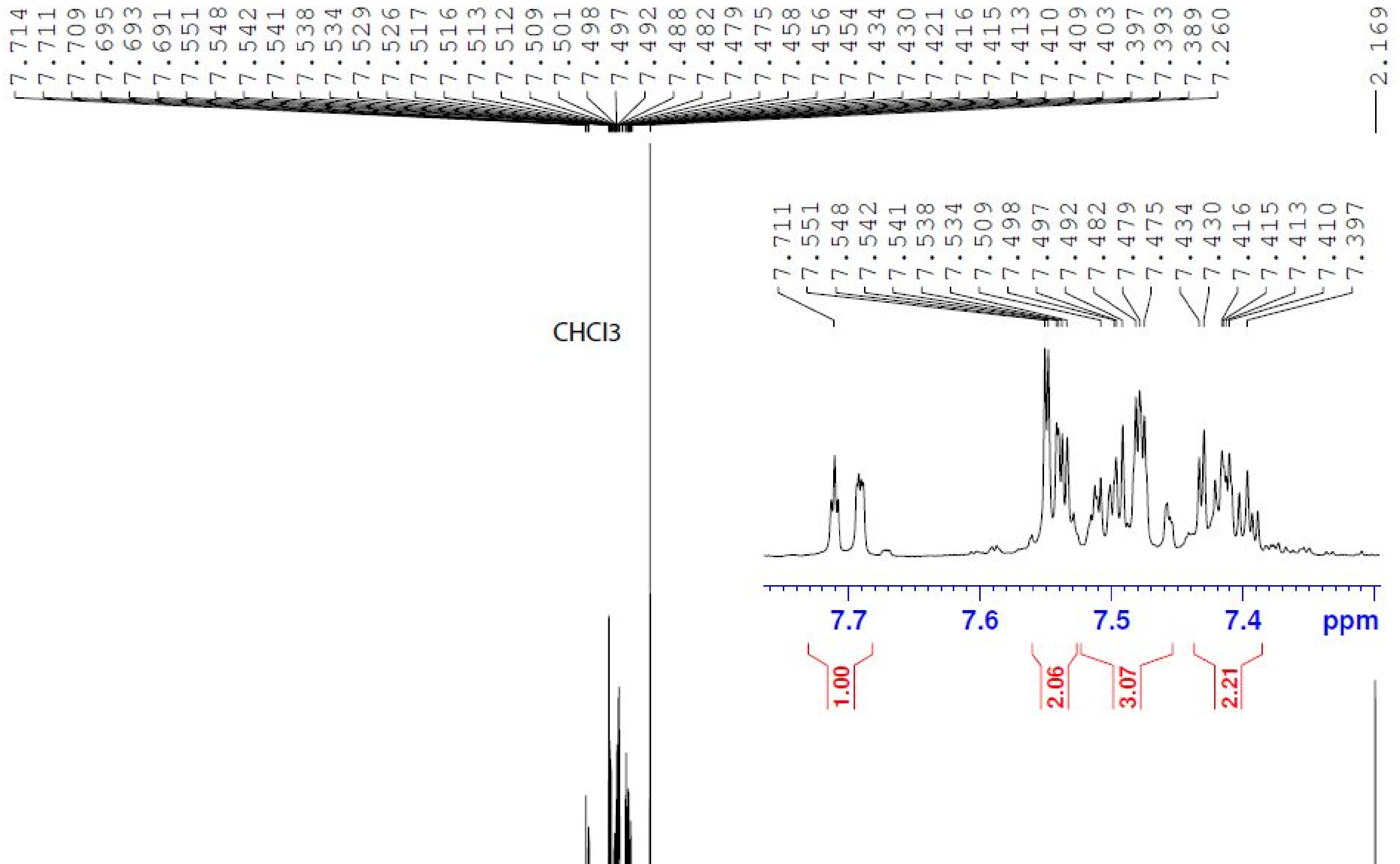

100 MHz

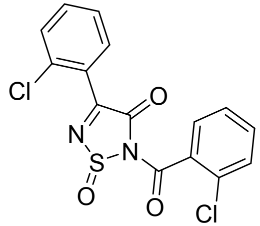

5h

CDCl$_3$

2-(2-chlorobenzoyl)-4-(2-chlorophenyl)-1,2,5-thiadiazol-3(2H)-one 1-oxide

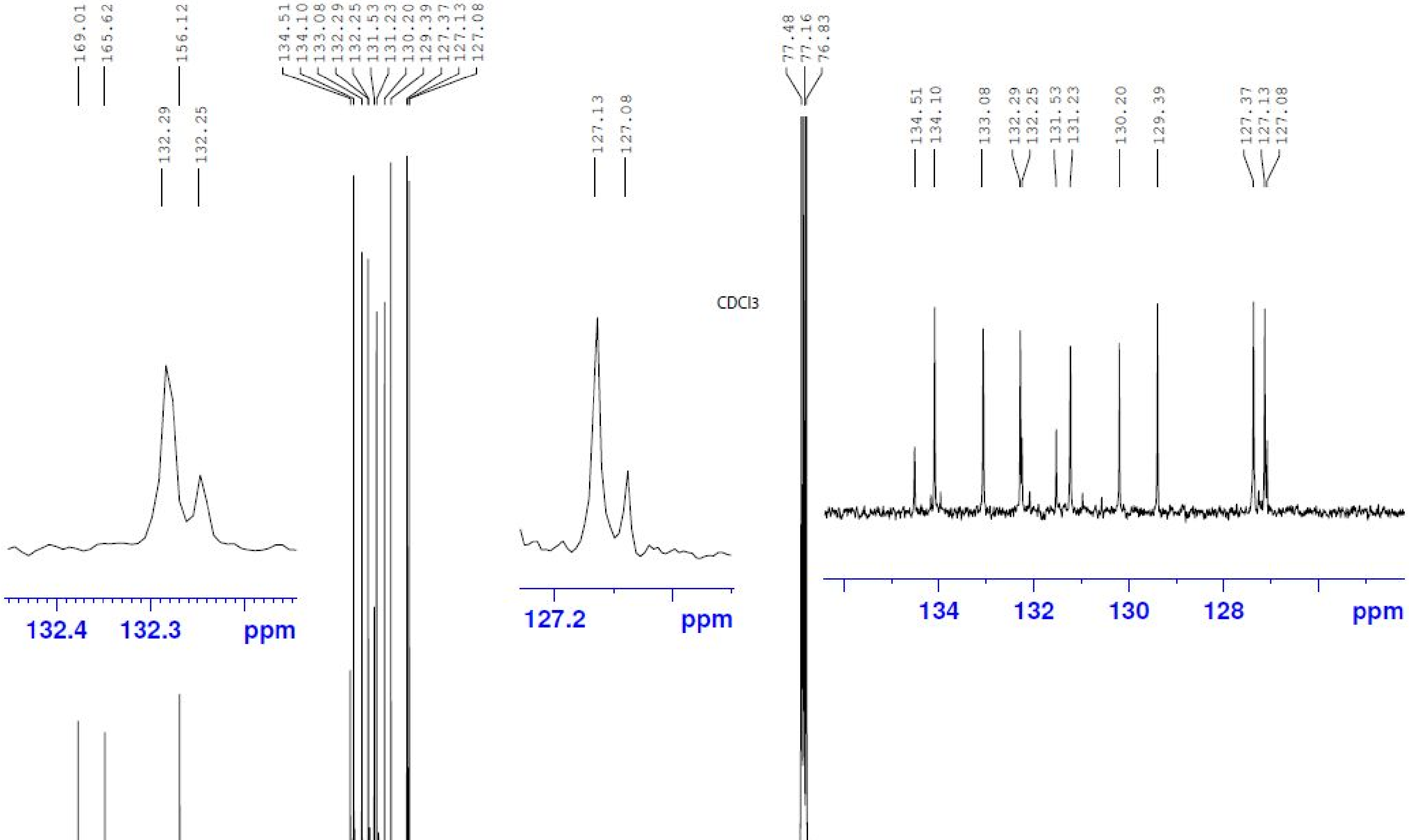

400 MHz

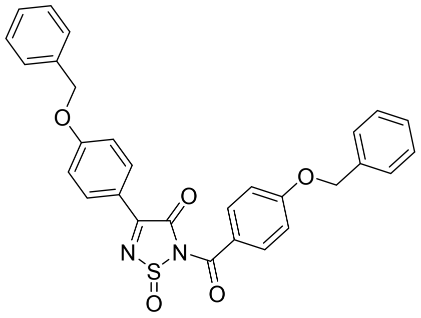

5k

CD_2Cl_2

2-(4-(benzyloxy)benzoyl)-4-(4-(benzyloxy)phenyl)-1,2,5-thiadiazol-3(2H)-one 1-oxide (**34b**)

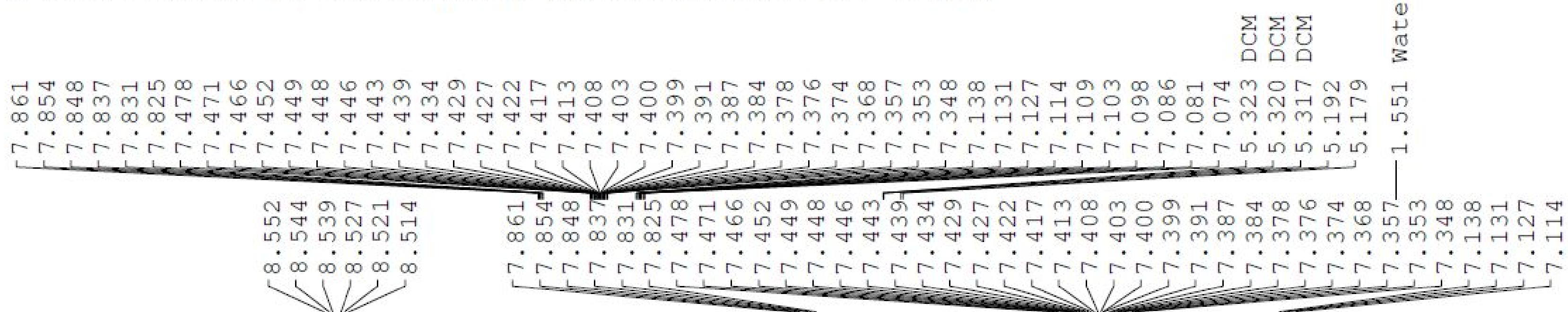

CD$_2$Cl$_2$

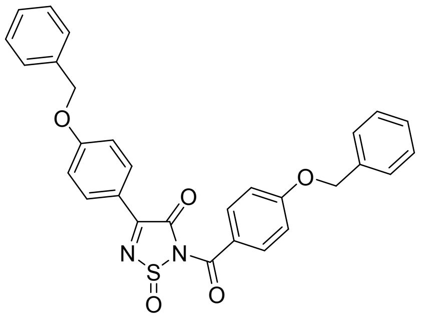

5k

100 MHz

2-(4-(benzyloxy)benzoyl)-4-(4-(benzyloxy)phenyl)-1,2,5-thiadiazol-3(2H)-one 1-oxide (**34b**)

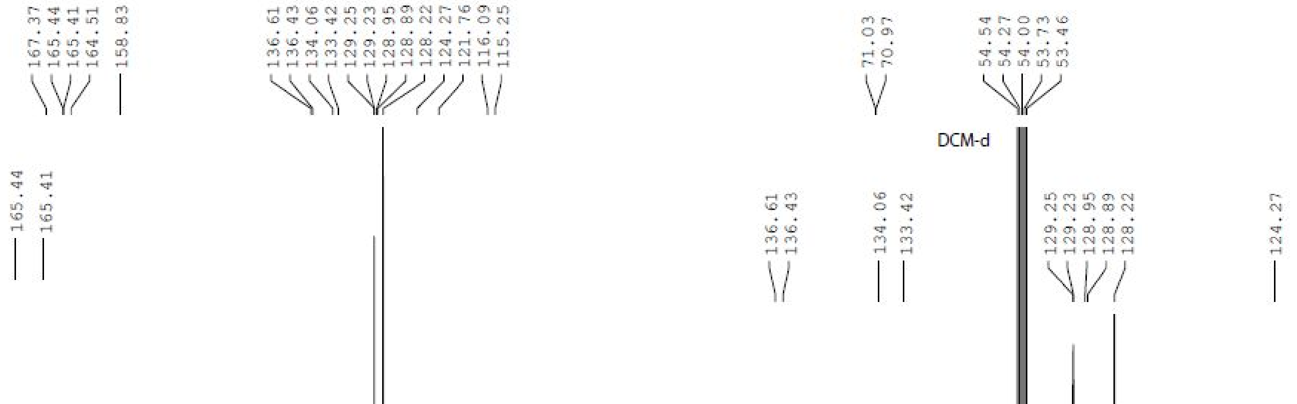

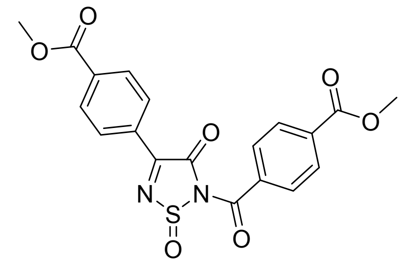

5l

CD_2Cl_2

400 MHz

Methyl 4-(5-(4-(methoxycarbonyl)benzoyl)-1-oxido-4-oxo-4,5-dihydro-1,2,5-thiadiazol-3-yl)benzoate

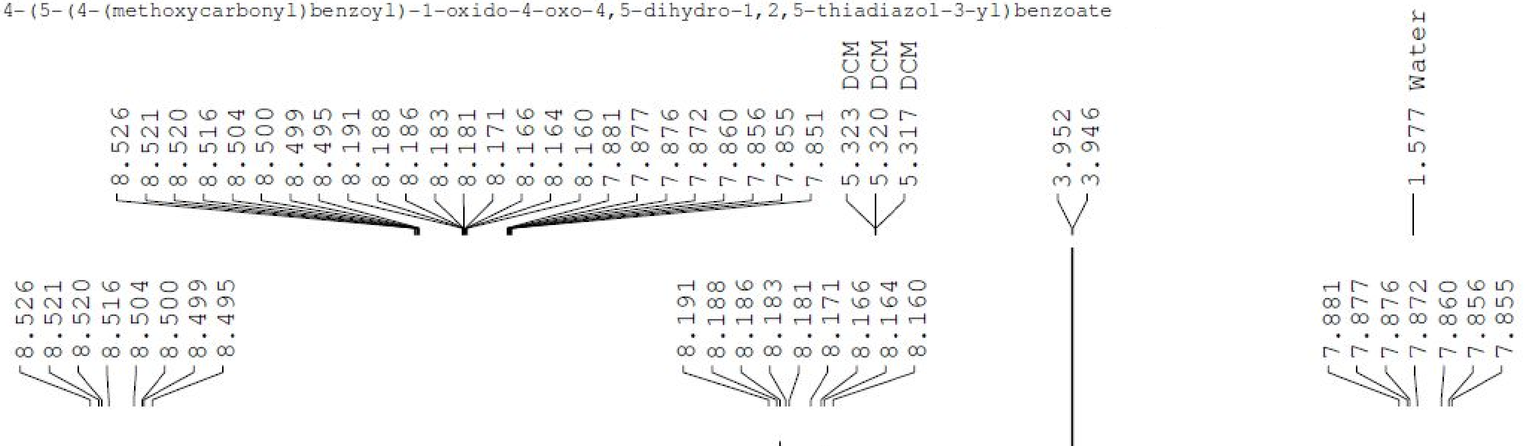

100 MHz

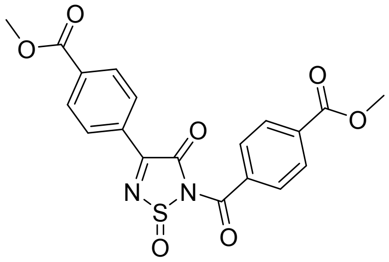

CD_2Cl_2

5l

methyl 4-(5-(4-(methoxycarbonyl)benzoyl)-1-oxido-4-oxo-4,5-dihydro-1,2,5-thiadiazol-3-yl)benzoate (**32b**)

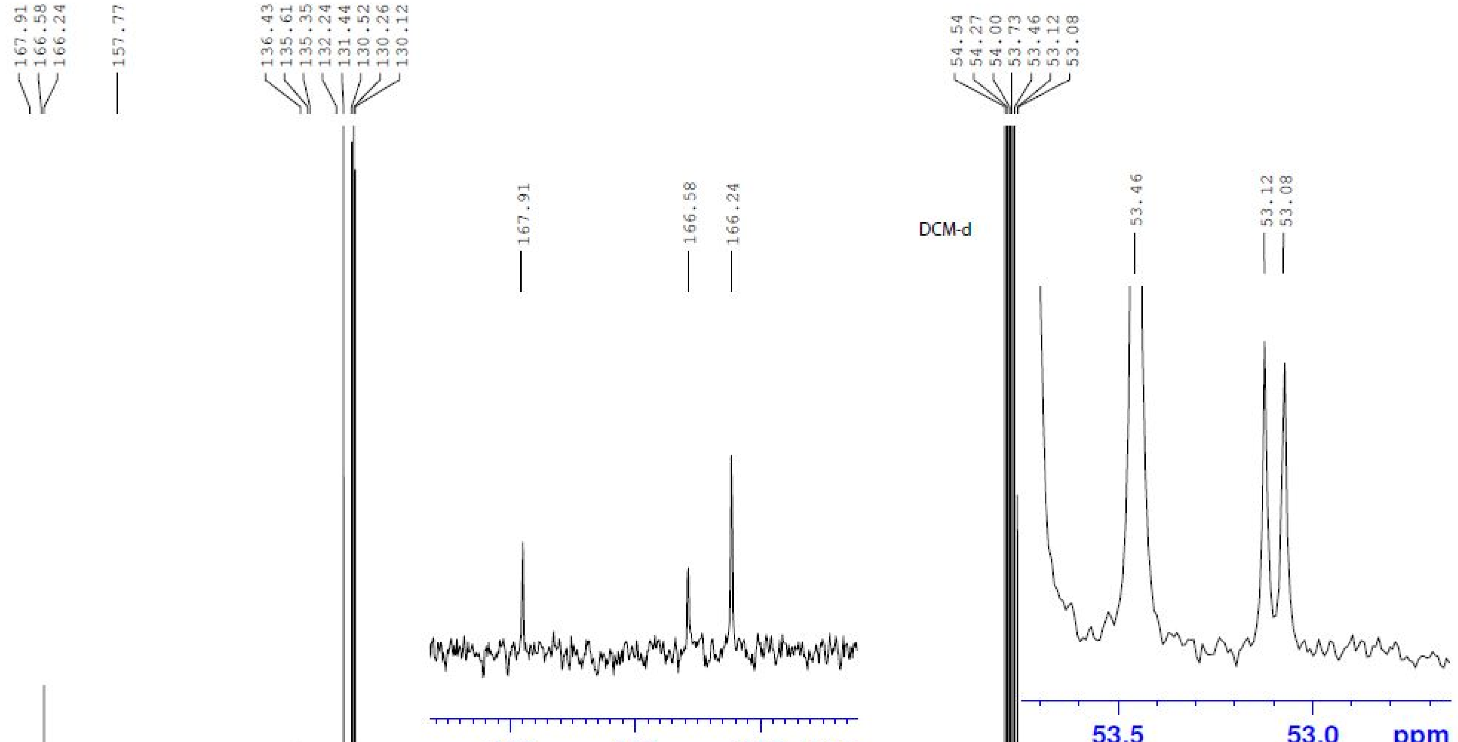

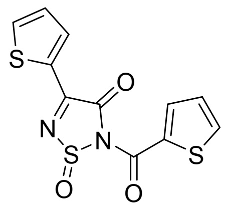

5m

CDCl$_3$

300 MHz

4-(thiophen-2-yl)-2-(thiophene-2-carbonyl)-1,2,5-thiadiazol-3(2H)-one 1-oxide(**27b**)

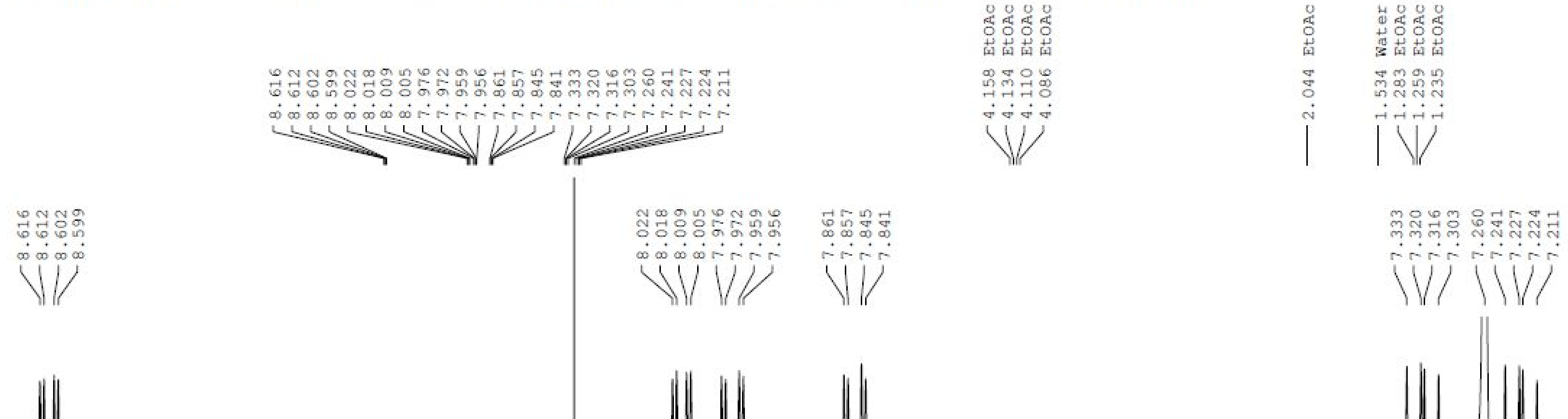

CDCl$_3$

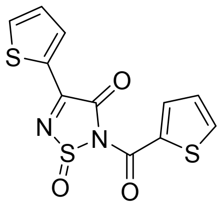

5m

100 MHz

4-(thiophen-2-yl)-2-(thiophene-2-carbonyl)-1,2,5-thiadiazol-3(2H)-one 1-oxide (**27b**)

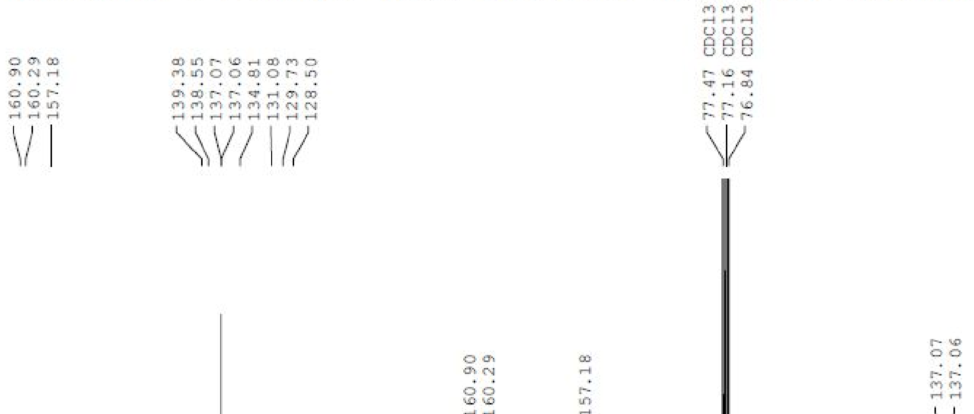

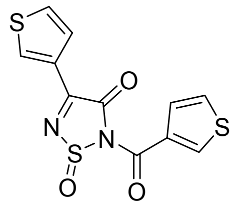

5n

$CDCl_3$

400 MHz

4-(thiophen-3-yl)-2-(thiophene-3-carbonyl)-1,2,5-thiadiazol-3(2H)-one 1-oxide **(28b)**

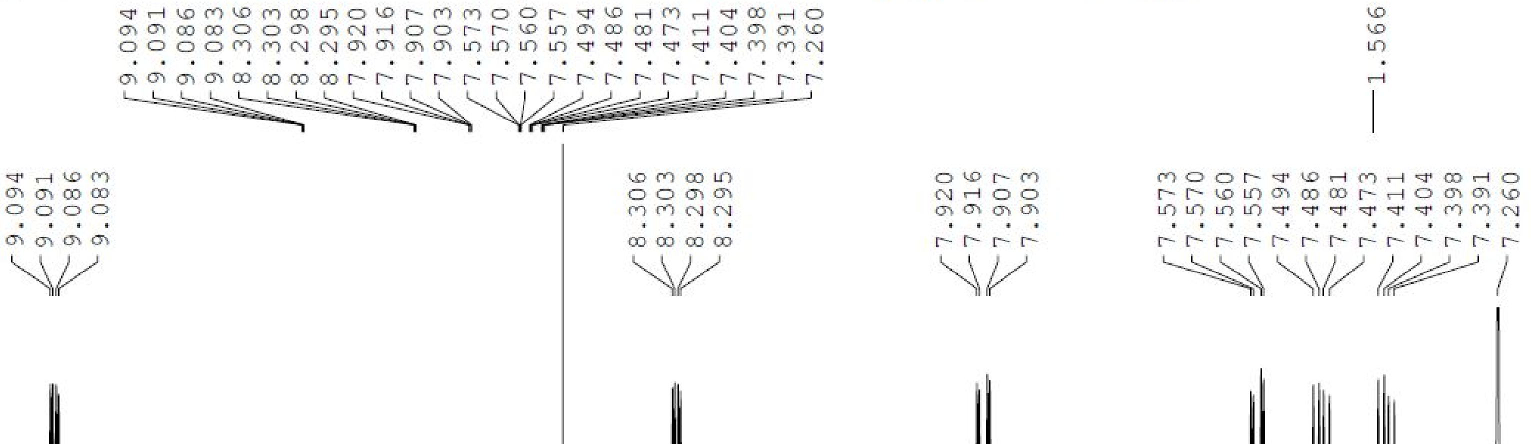

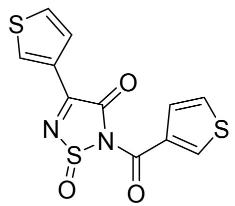

5n

CDCl$_3$

100 MHz

4-(thiophen-3-yl)-2-(thiophene-3-carbonyl)-1,2,5-thiadiazol-3(2H)-one 1-oxide(**28b**)

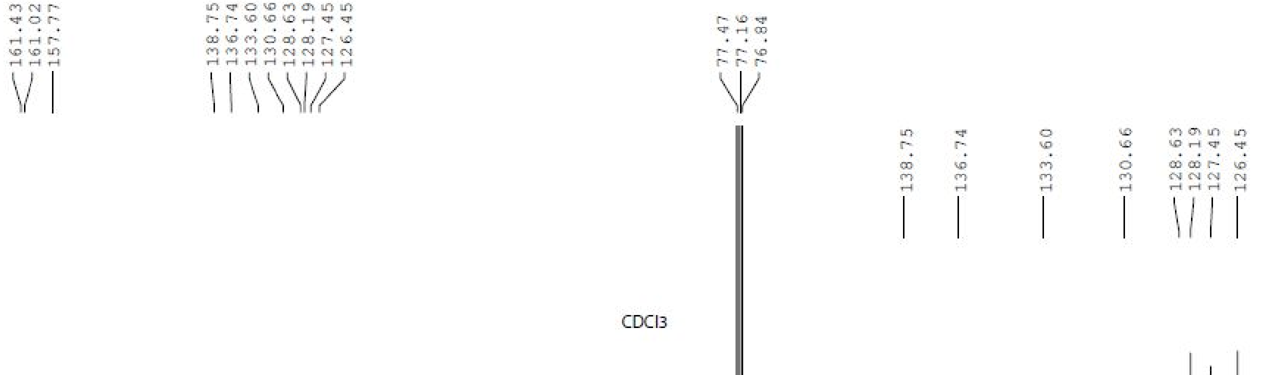

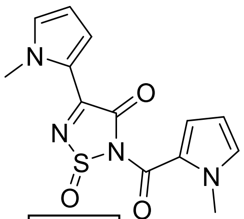

5o

400 MHz

CDCl$_3$

4-(1-methyl-1H-pyrrol-2-yl)-2-(1-methyl-1H-pyrrole-2-carbonyl)-1,2,5-thiadiazol-3(2H)-one 1-oxide (**29b**)

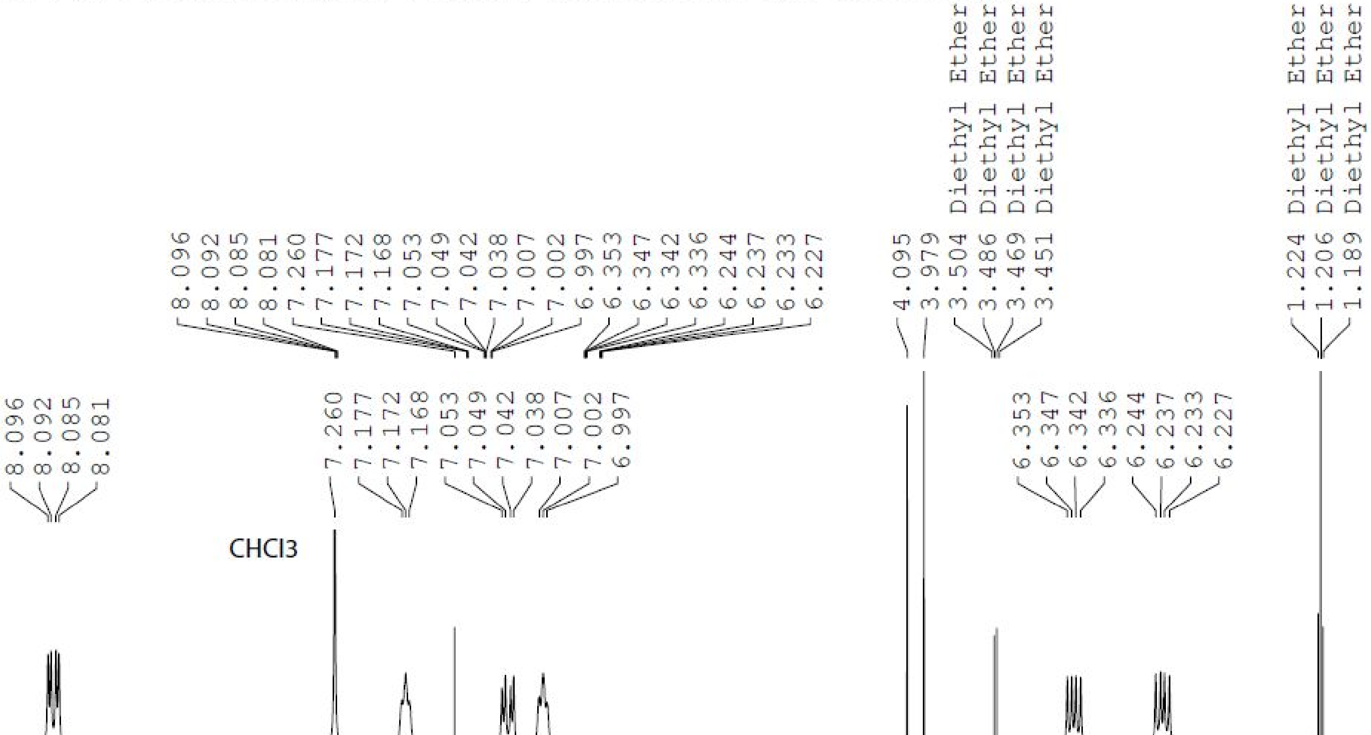

100 MHz

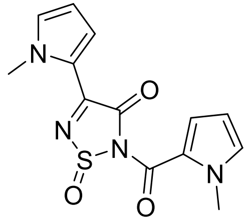

5o

CDCl$_3$

4-(1-methyl-1H-pyrrol-2-yl)-2-(1-methyl-1H-pyrrole-2-carbonyl)-1,2,5-thiadiazol-3(2H)-one 1-oxide **(29b)**

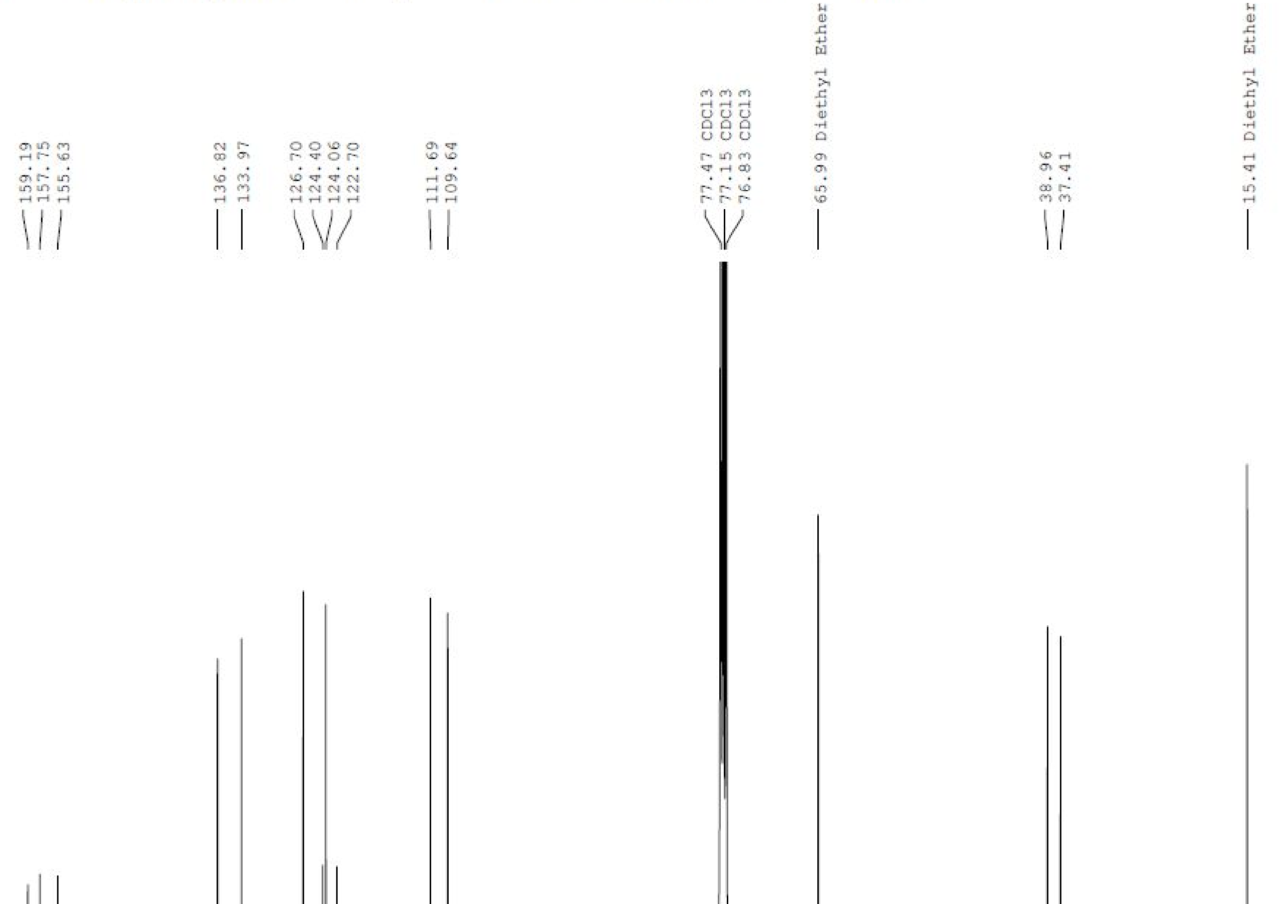

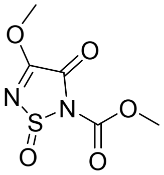

5r

300 MHz

$CDCl_3$

methyl 4-methoxy-3-oxo-1,2,5-thiadiazole-2(3H)-carboxylate 1-oxide (**22b**)

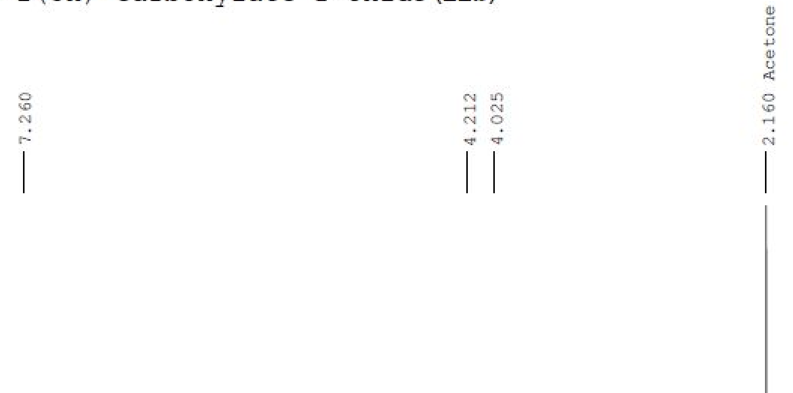

100 MHz

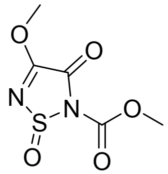

5r

CDCl$_3$

methyl 4-methoxy-3-oxo-1,2,5-thiadiazole-2(3H)-carboxylate 1-oxide **(23b)**

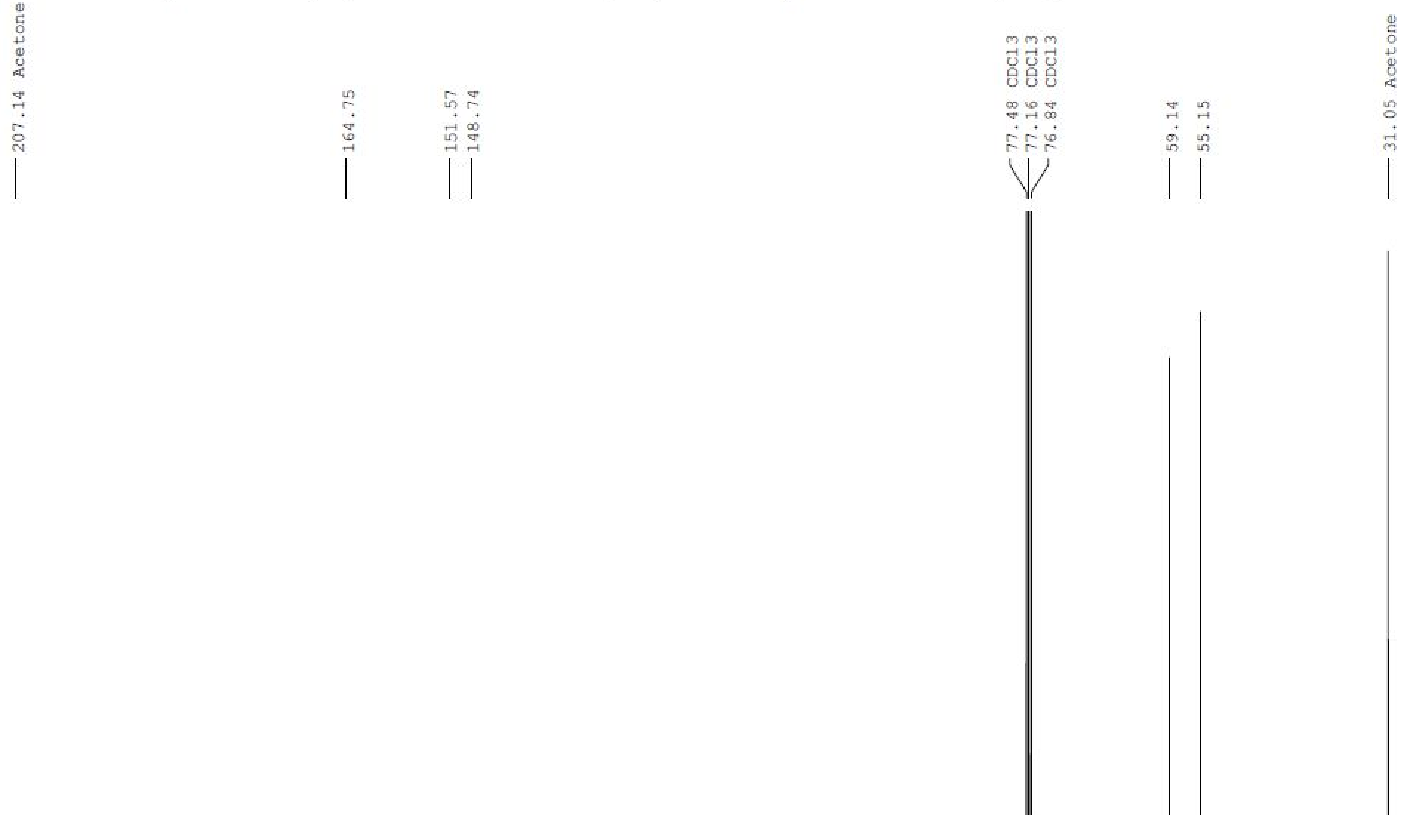

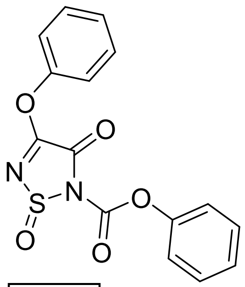

5t

Acetone-d_6

400 MHz

phenyl 3-oxo-4-phenoxy-1,2,5-thiadiazole-2(3H)-carboxylate 1-oxide (**23b**)

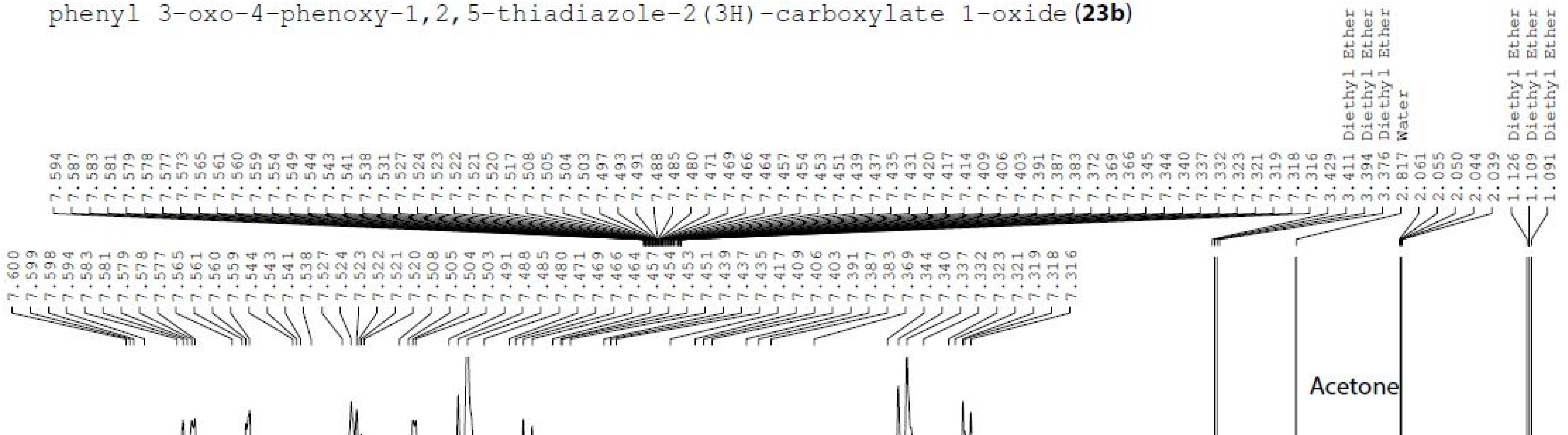

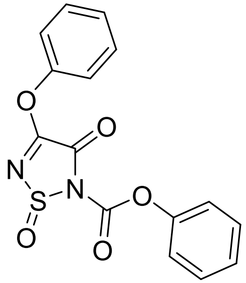

5t

Acetone-d_6

Acetone-d_6

phenyl 3-oxo-4-phenoxy-1,2,5-thiadiazole-2(3H)-carboxylate 1-oxide

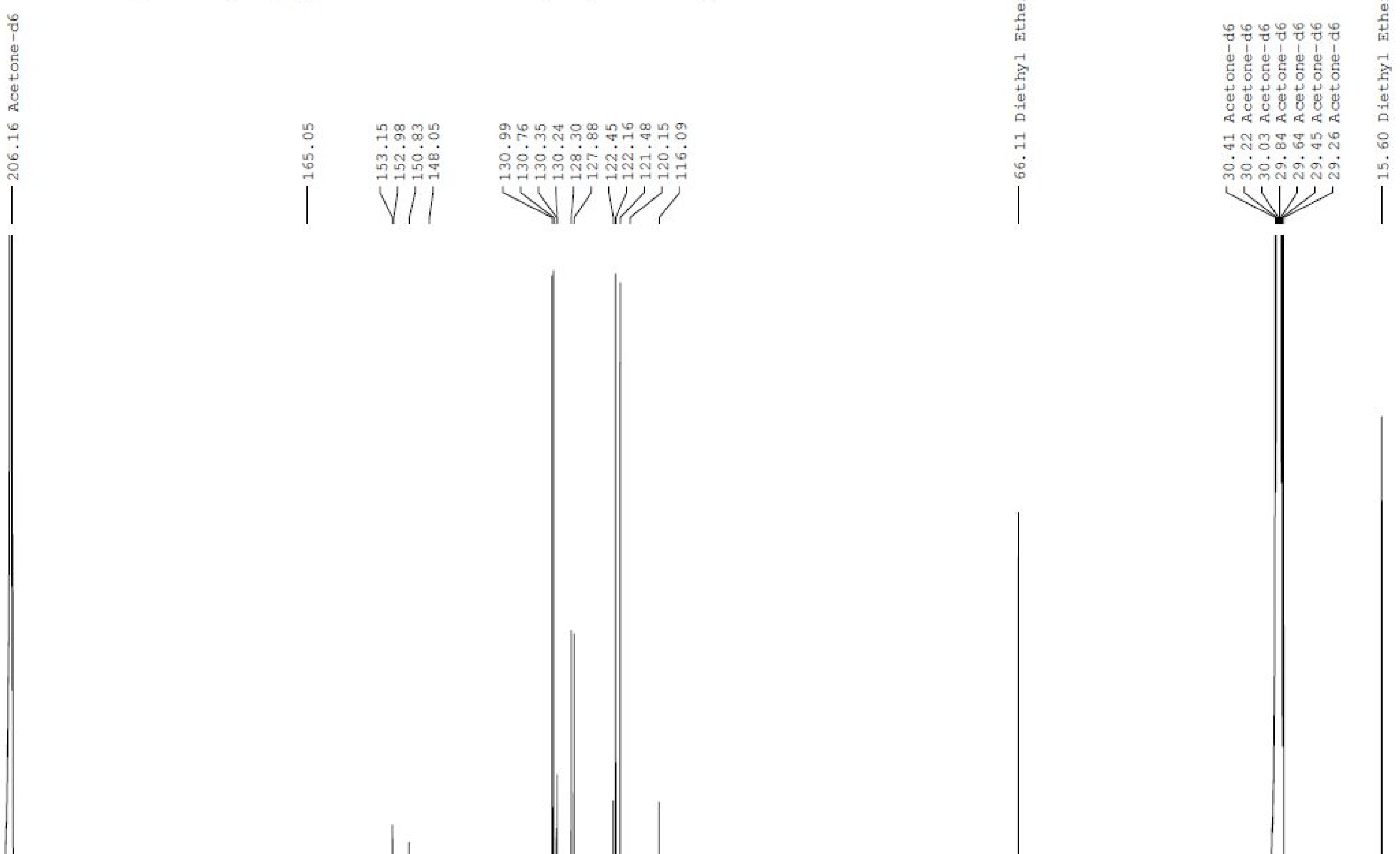

100 MHz

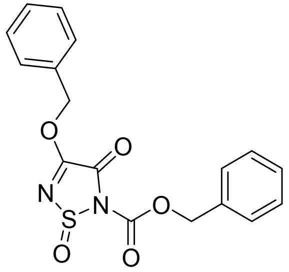

5s

$CDCl_3$

400 MHz

benzyl 4-(benzyloxy)-3-oxo-1,2,5-thiadiazole-2(3H)-carboxylate 1-oxide (**24b**)

CHCl3

7.476 7.468 7.463 7.459 7.453 7.434 7.429 7.418 7.414 7.410 7.407 7.401 7.397 7.391 7.390 7.385 7.380 7.375 7.371 7.362 7.358 7.260 5.531 5.461 5.431 5.406 5.375

7.459 7.453 7.434 7.429 7.418 7.414 7.410 7.407 7.401 7.391 7.390 7.385 7.380 7.375 7.371 7.260

5.531 5.461 5.431 5.406 5.375

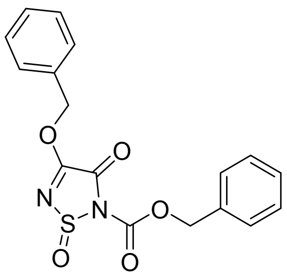

5s

CDCl$_3$

100 MHz

benzyl 4-(benzyloxy)-3-oxo-1,2,5-thiadiazole-2(3H)-carboxylate 1-oxide (**24b**)

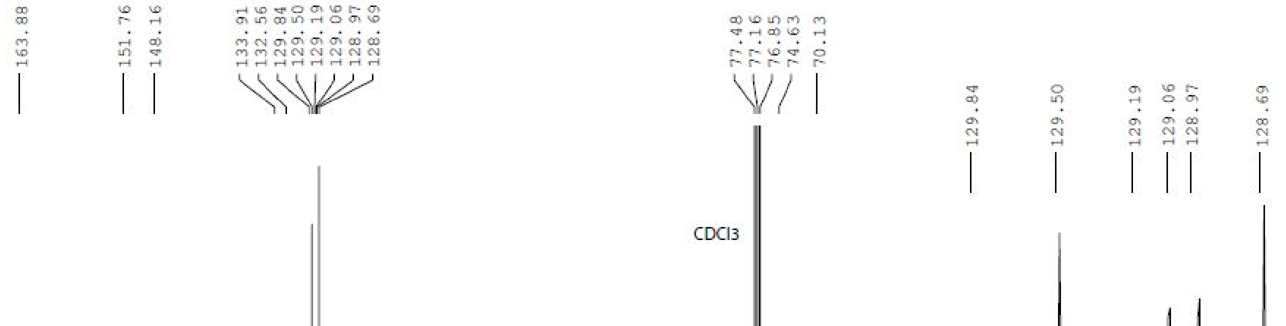

300 MHz

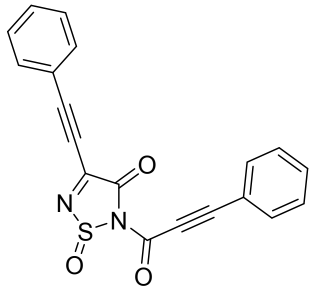

5q

CDCl$_3$

4-(phenylethynyl)-2-(3-phenylpropioloyl)-1,2,5-thiadiazol-3(2H)-one 1-oxide(**33b**)

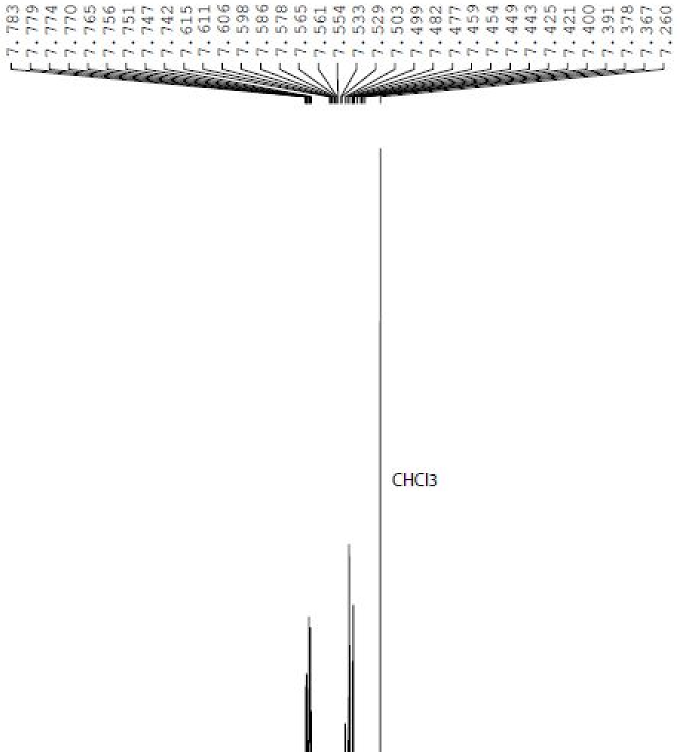

CDCl$_3$

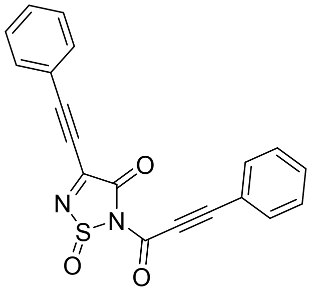

5q

100 MHz

4-(phenylethynyl)-2-(3-phenylpropioloyl)-1,2,5-thiadiazol-3(2H)-one 1-oxide(**33b**)

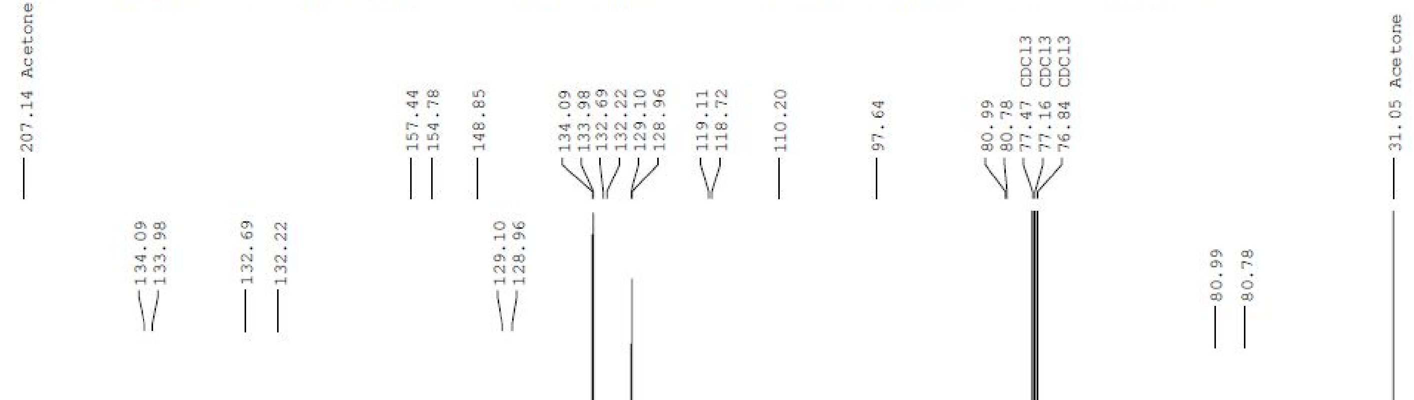

CDCl$_3$

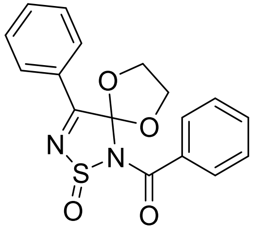

5u

400 MHz

(2-oxido-4-phenyl-6,9-dioxa-2-thia-1,3-diazaspiro[4.4]non-3-en-1-yl)(phenyl)methanone(**5b**)

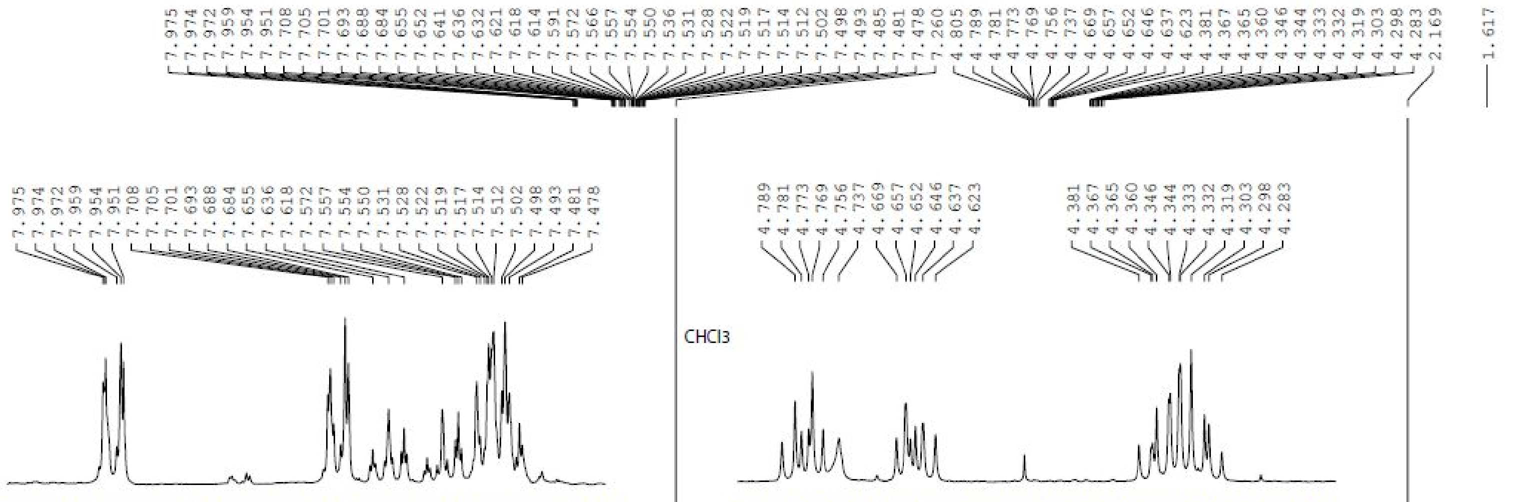

CDCl$_3$

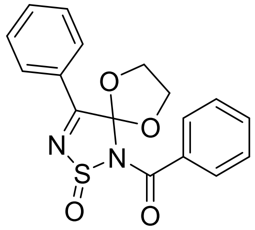

5u

100 MHz

(2-oxido-4-phenyl-6,9-dioxa-2-thia-1,3-diazaspiro[4.4]non-3-en-1-yl)(phenyl)methanone (**5b**)

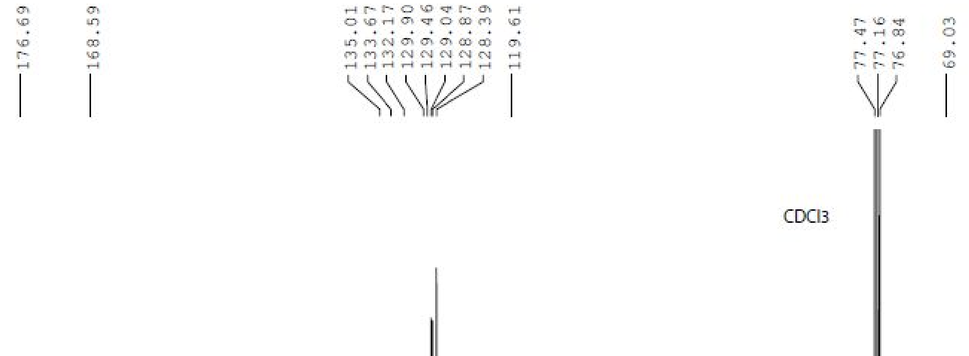

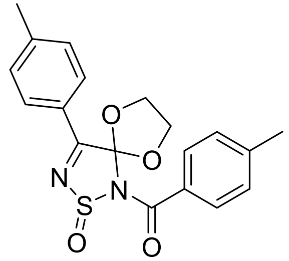

5v

$CDCl_3$

400 MHz

(2-oxido-4-(p-tolyl)-6,9-dioxa-2-thia-1,3-diazaspiro[4.4]non-3-en-1-yl)(p-tolyl)methanone (**6b**)

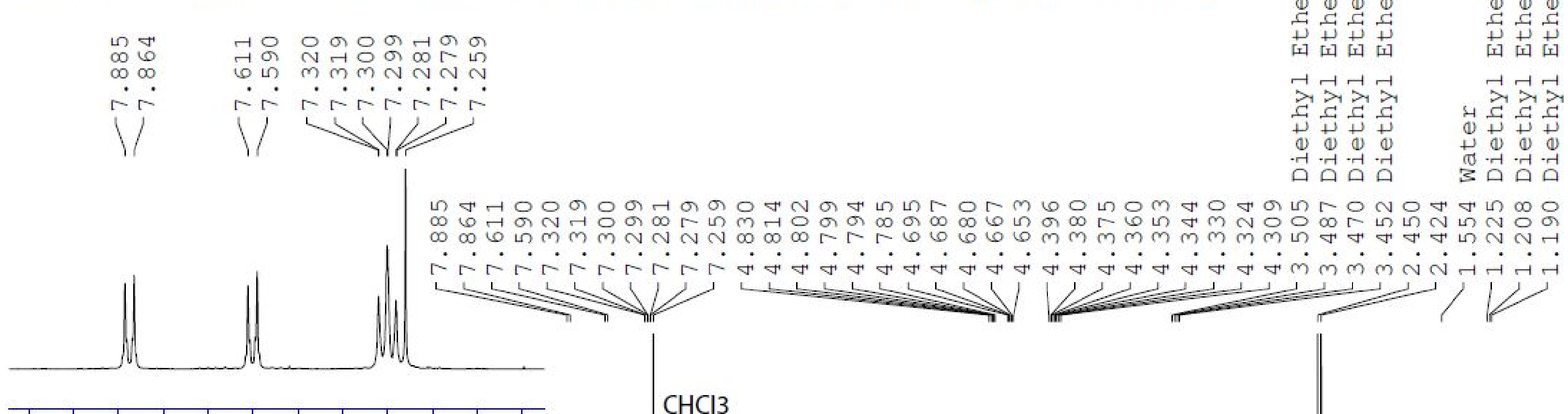

CDCl$_3$

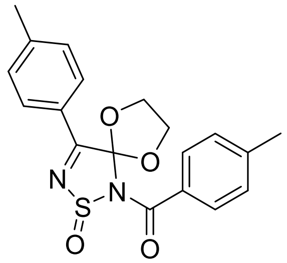

5v

100 MHz

(2-oxido-4-(p-tolyl)-6,9-dioxa-2-thia-1,3-diazaspiro[4.4]non-3-en-1-yl)(p-tolyl)methanone (**6b**)

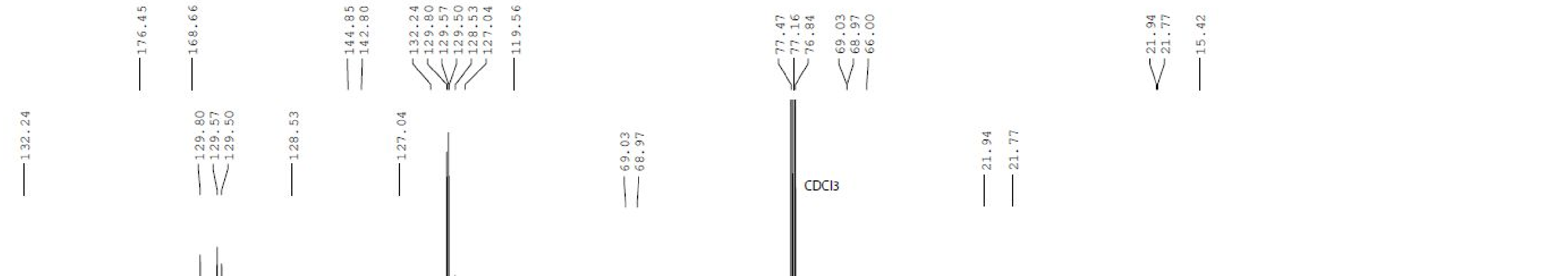

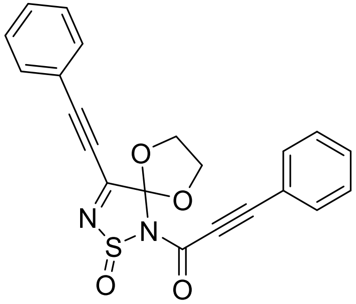

5w

CDCl$_3$

400 MHz

1-(2-oxido-4-(phenylethynyl)-6,9-dioxa-2-thia-1,3-diazaspiro[4.4]non-3-en-1-yl)-3-phenylprop-2-yn-1-one (**7b**)

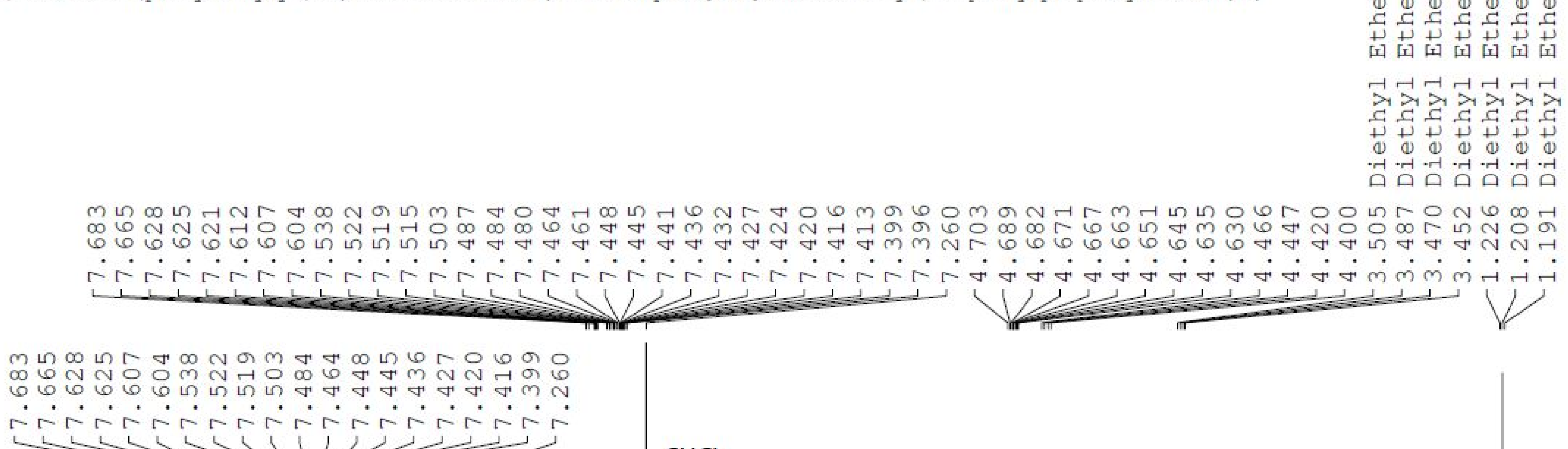

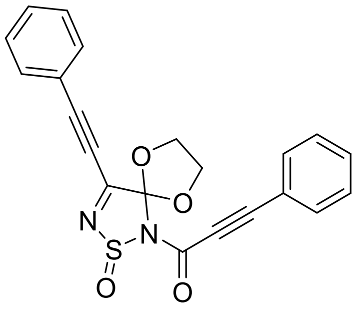

5w

CDCl$_3$

100 MHz

1-(2-oxido-4-(phenylethynyl)-6,9-dioxa-2-thia-1,3-diazaspiro[4.4]non-3-en-1-yl)-3-phenylprop-2-yn-1-one(**7b**)

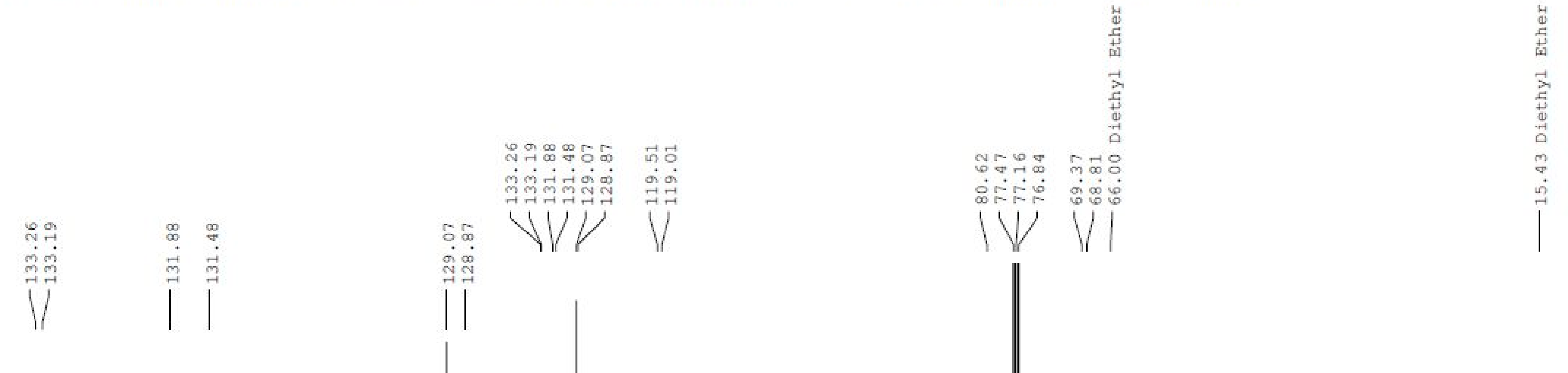

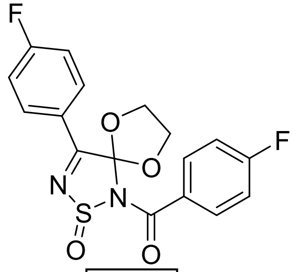

5x

CDCl$_3$

400 MHz

(4-fluorophenyl)(4-(4-fluorophenyl)-2-oxido-6,9-dioxa-2-thia-1,3-diazaspiro[4.4]non-3-en-1-yl)methanone(**8b**)

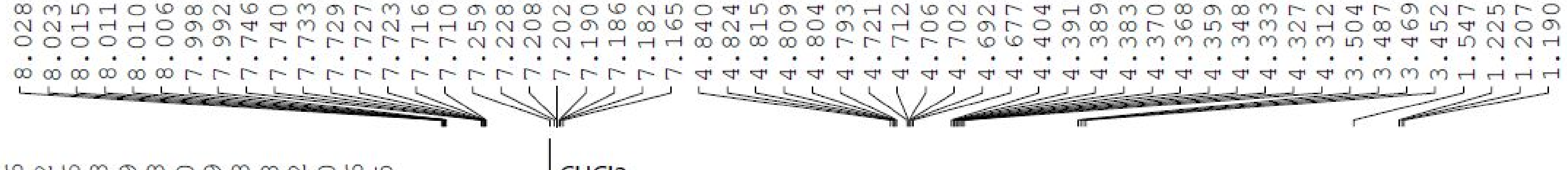

CDCl$_3$

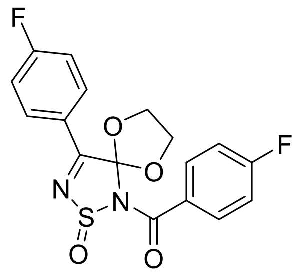

5x

100 MHz

(4-fluorophenyl)(4-(4-fluorophenyl)-2-oxido-6,9-dioxa-2-thia-1,3-diazaspiro[4.4]non-3-en-1-yl)methanone(**8b**)

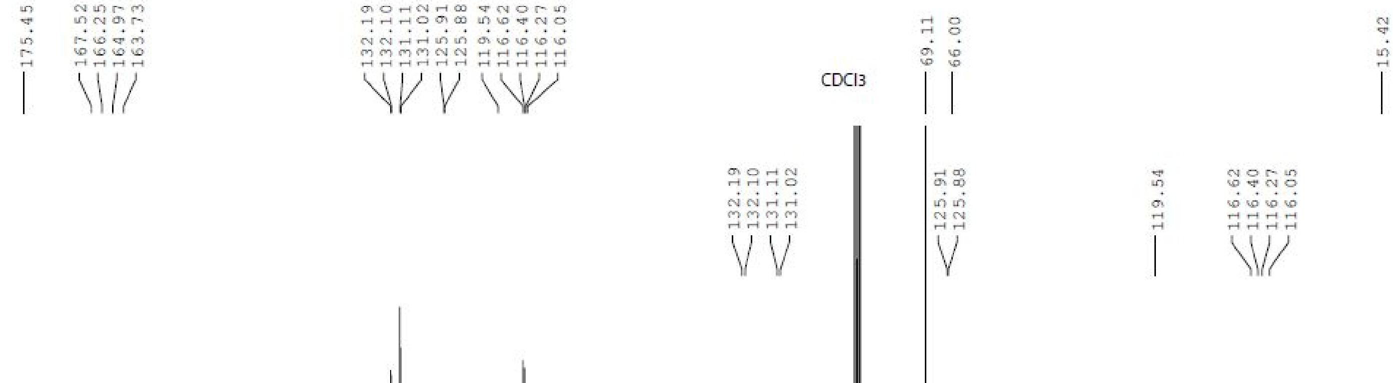

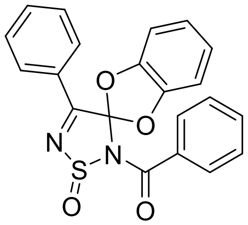

5y

CDCl$_3$

300 MHz

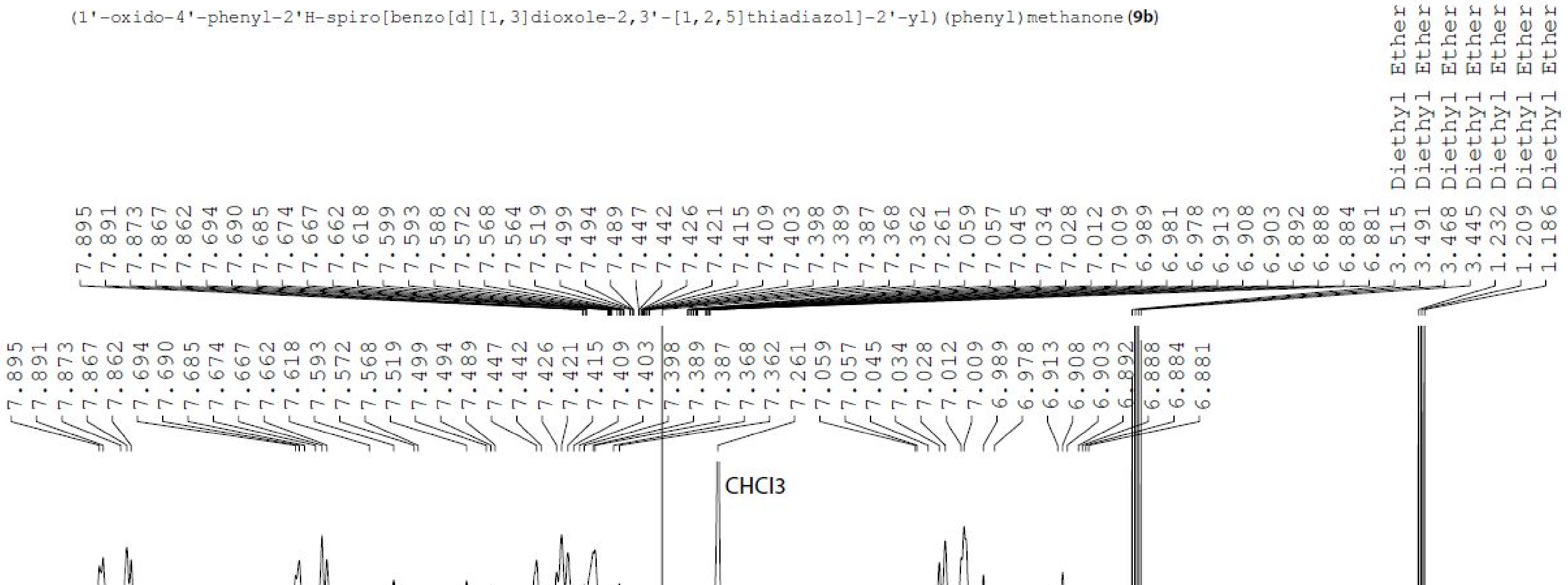

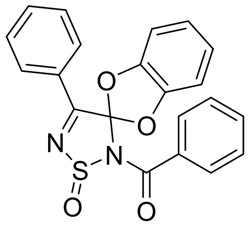

5y

$CDCl_3$

100 MHz

(1'-oxido-4'-phenyl-2'H-spiro[benzo[d][1,3]dioxole-2,3'-[1,2,5]thiadiazol]-2'-yl)(phenyl)methanone(**9b**)

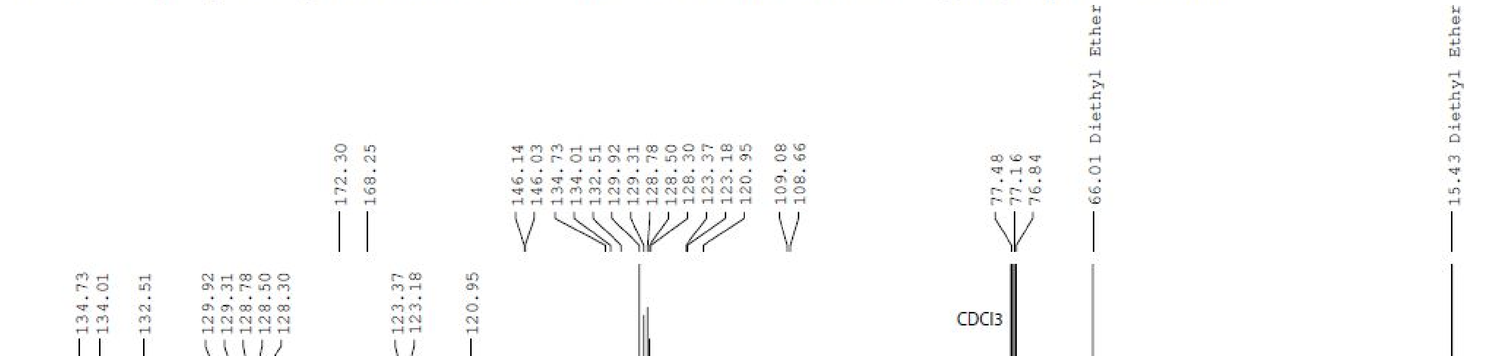

400 MHz

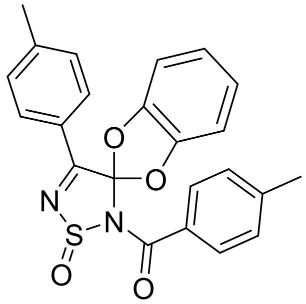

5z

CDCl$_3$

(1'-oxido-4'-(p-tolyl)-2'H-spiro[benzo[d][1,3]dioxole-2,3'-[1,2,5]thiadiazol]-2'-yl)(p-tolyl)methanone (**11b**)

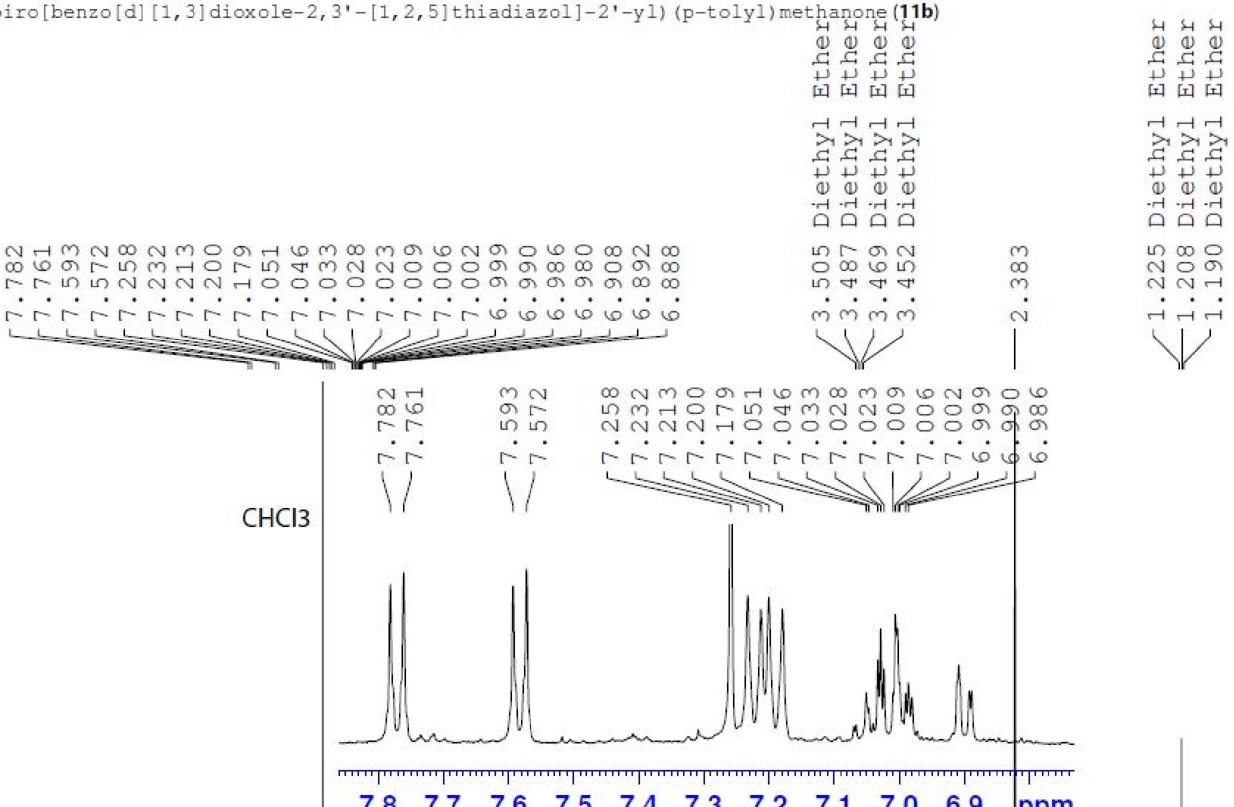

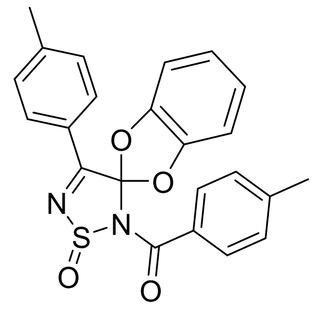

5z

CDCl$_3$

100 MHz

(1'-oxido-4'-(p-tolyl)-2'H-spiro[benzo[d][1,3]dioxole-2,3'-[1,2,5]thiadiazol]-2'-yl)(p-tolyl)methanone (**11b**)

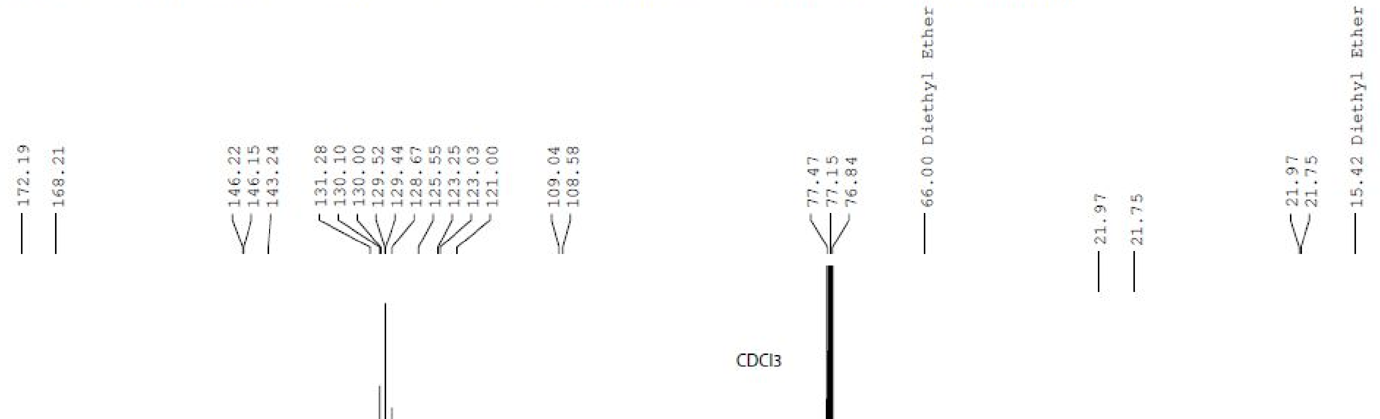

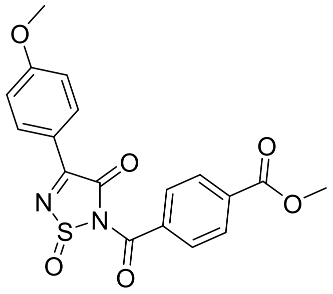

5aa

CDCl$_3$

400 MHz

methyl 4-(4-(4-methoxyphenyl)-1-oxido-3-oxo-2,3-dihydro-1,2,5-thiadiazole-2-carbonyl)benzoate (**40b**)

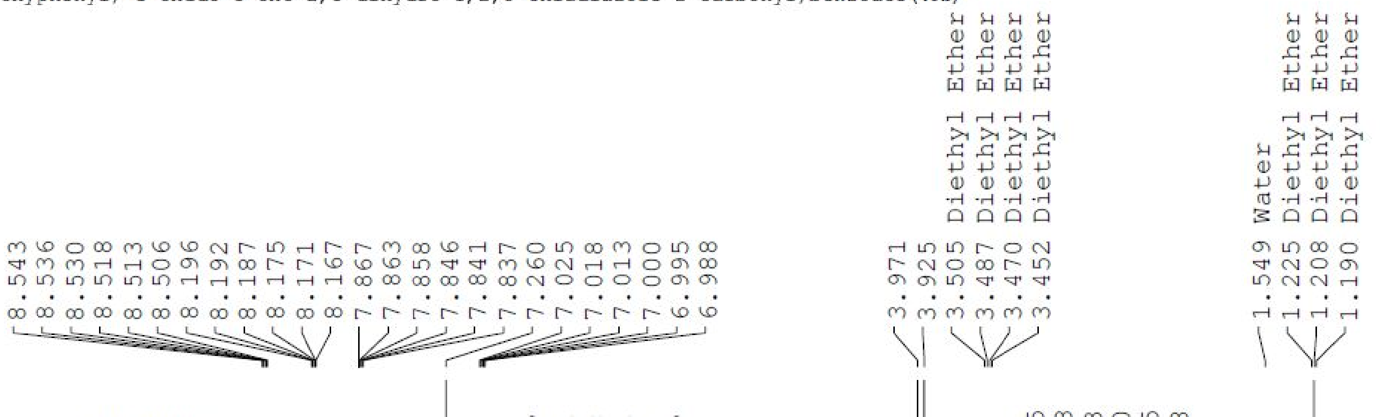

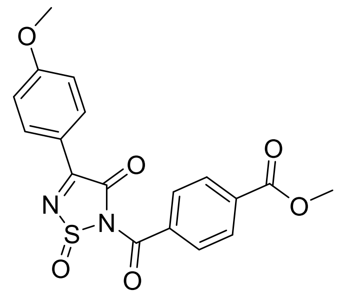

5aa

CDCl$_3$

100 MHz

methyl 4-(4-(4-methoxyphenyl)-1-oxido-3-oxo-2,3-dihydro-1,2,5-thiadiazole-2-carbonyl)benzoate(**40b**)

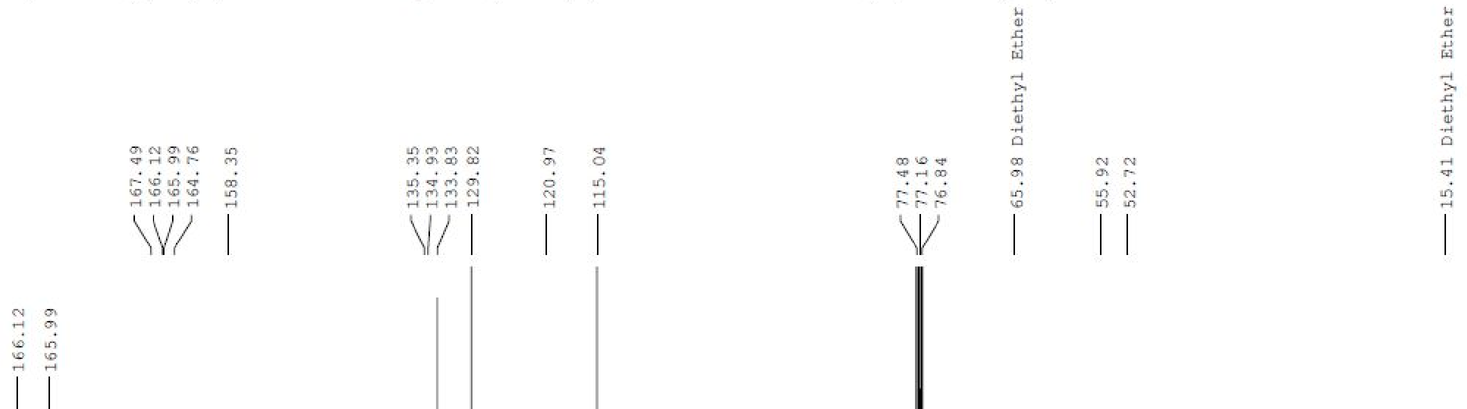

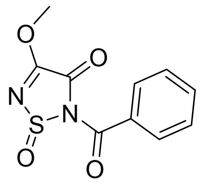

5ab

CDCl$_3$

400 MHz

2-benzoyl-4-methoxy-1,2,5-thiadiazol-3(2H)-one 1-oxide

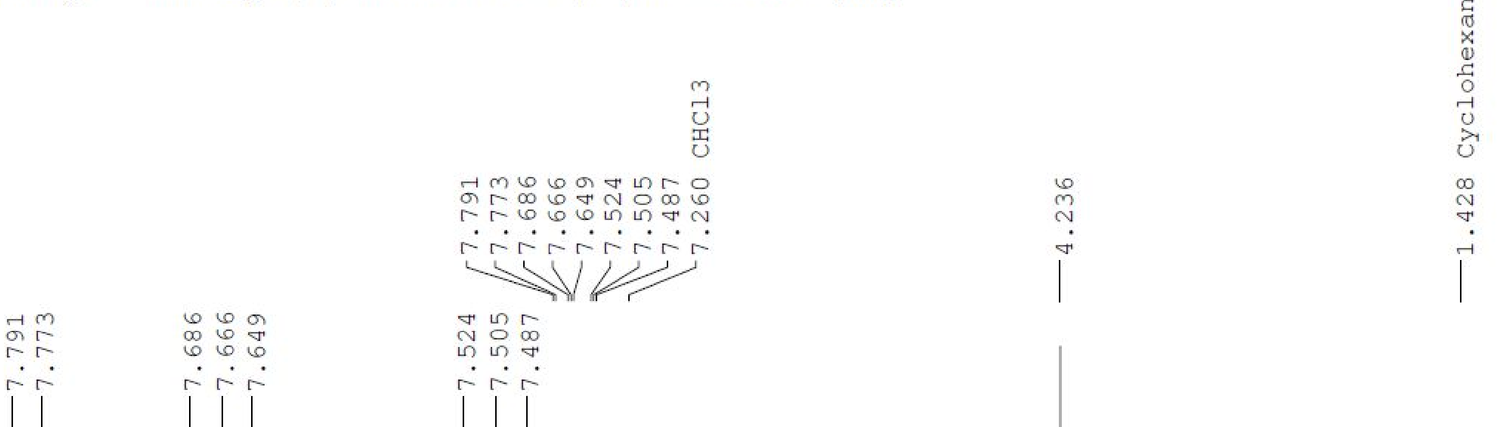

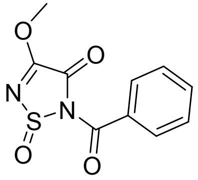

5ab

CDCl$_3$

100 MHz

2-benzoyl-4-methoxy-1,2,5-thiadiazol-3(2H)-one 1-oxide (**38b**)

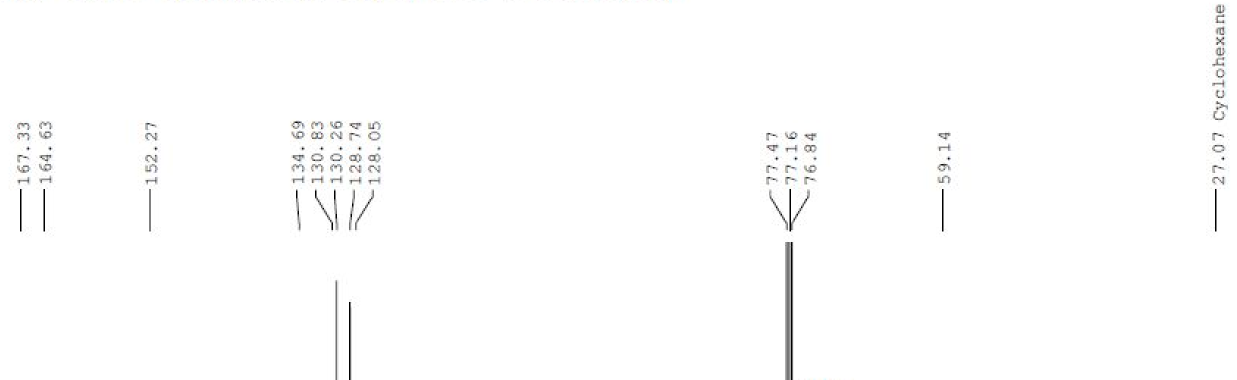

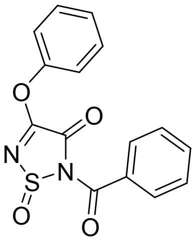

5ac

Acetone-d_6

400 MHz

2-benzoyl-4-phenoxy-1,2,5-thiadiazol-3(2H)-one 1-oxide(**39b**)

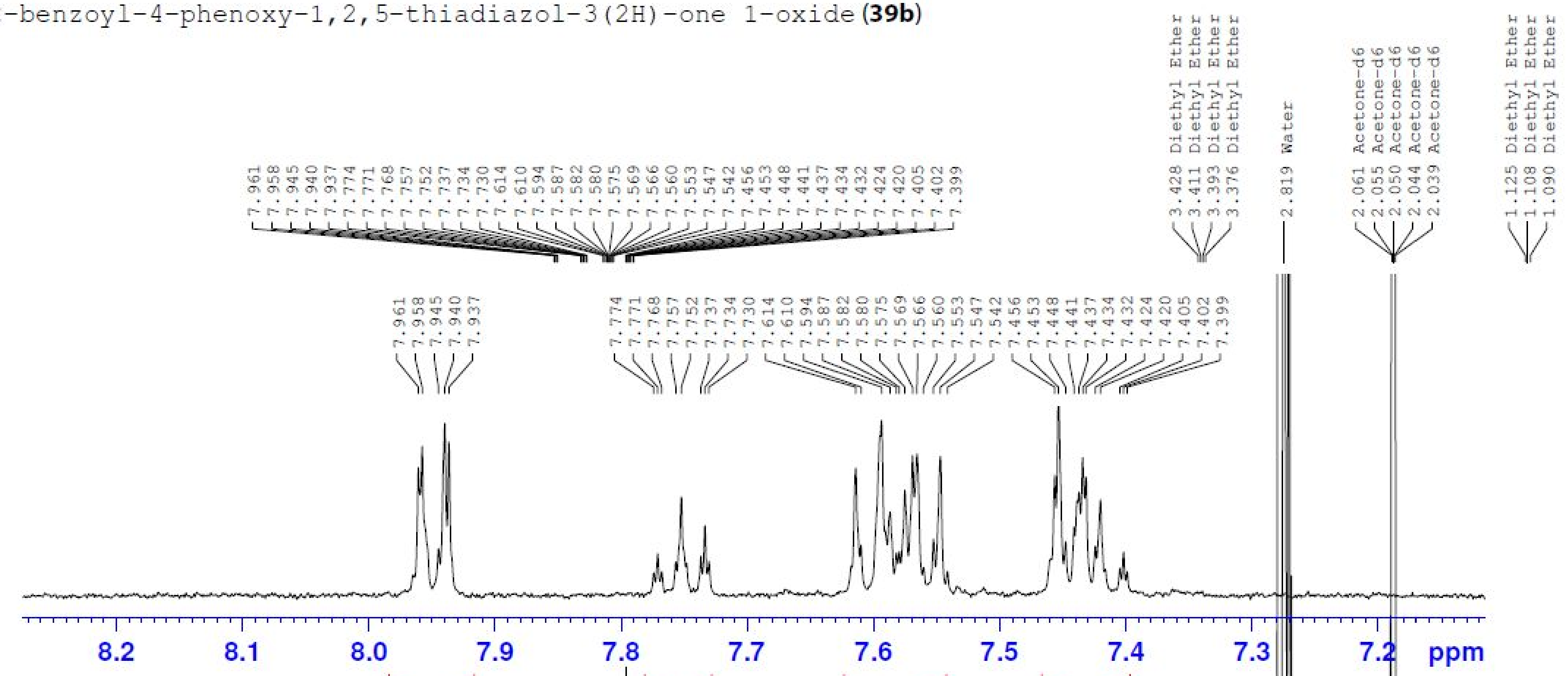

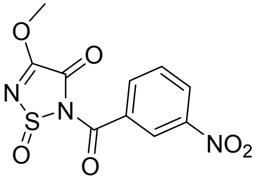

5ad

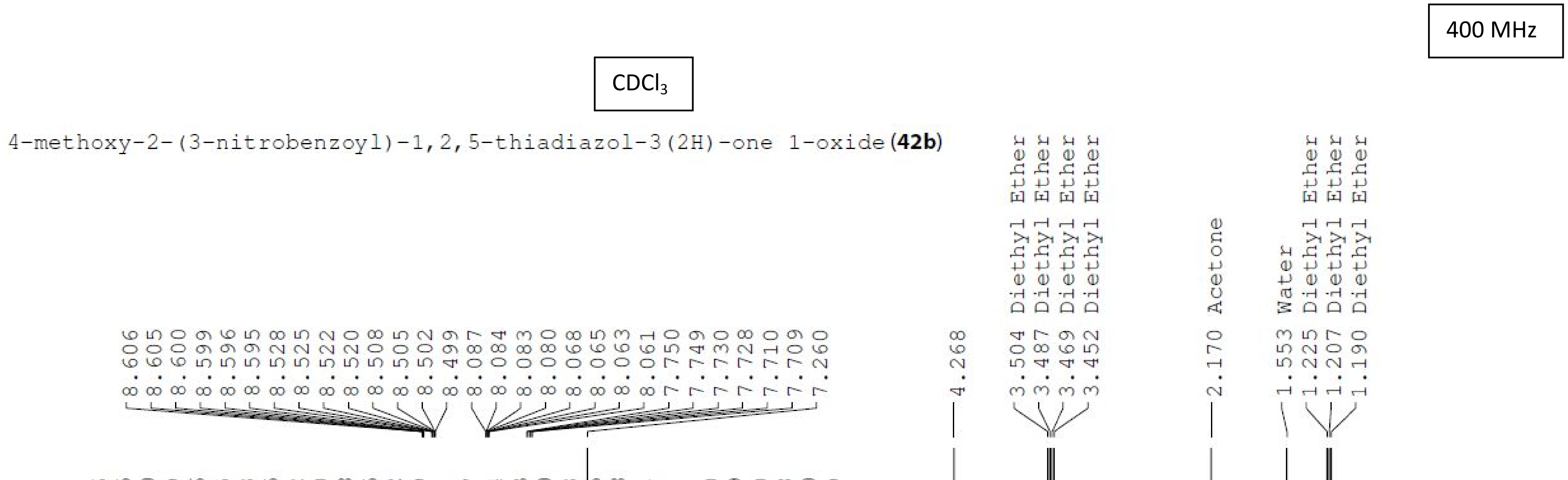
400 MHz
CDCl$_3$
4-methoxy-2-(3-nitrobenzoyl)-1,2,5-thiadiazol-3(2H)-one 1-oxide(**42b**)
8.606
8.605
8.600
8.599
8.596
8.595
8.528
8.525
8.522
8.520
8.508
8.505
8.502
8.499
8.087
8.084
8.083
8.080
8.068
8.065
8.063
8.061
7.750
7.749
7.730
7.728
7.710
7.709
7.260
4.268
3.504 Diethyl Ether
3.487 Diethyl Ether
3.469 Diethyl Ether
3.452 Diethyl Ether
2.170 Acetone
1.553 Water
1.225 Diethyl Ether
1.207 Diethyl Ether
1.190 Diethyl Ether

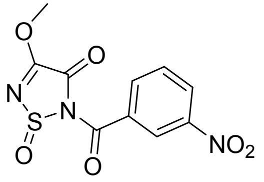

5ad

CDCl$_3$

100 MHz

4-methoxy-2-(3-nitrobenzoyl)-1,2,5-thiadiazol-3(2H)-one 1-oxide(**42b**)

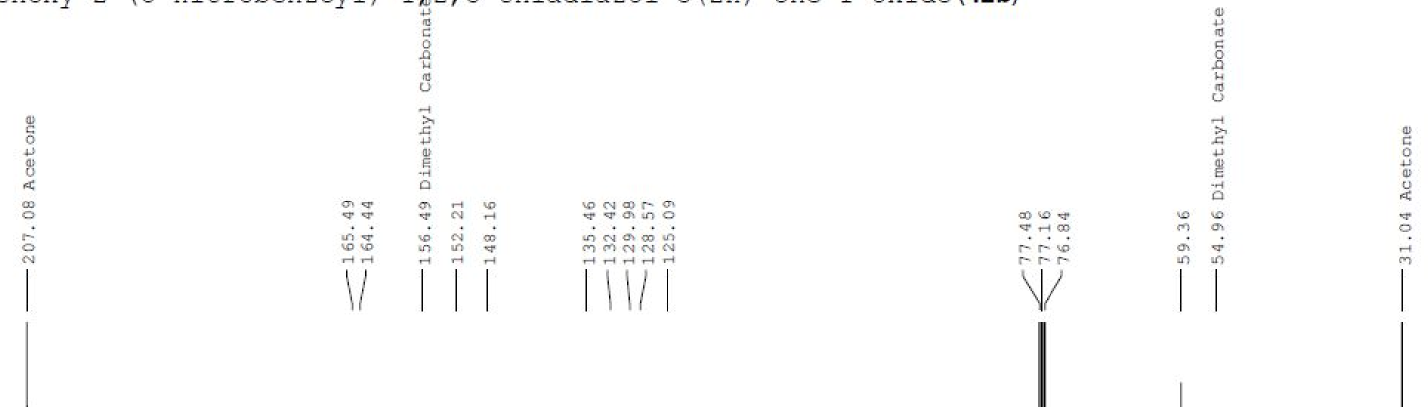

300 MHz

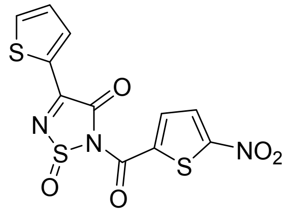

5ae

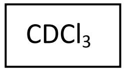

CDCl$_3$

2-(5-nitrothiophene-2-carbonyl)-4-(thiophen-2-yl)-1,2,5-thiadiazol-3(2H)-one 1-oxide (**41b**)

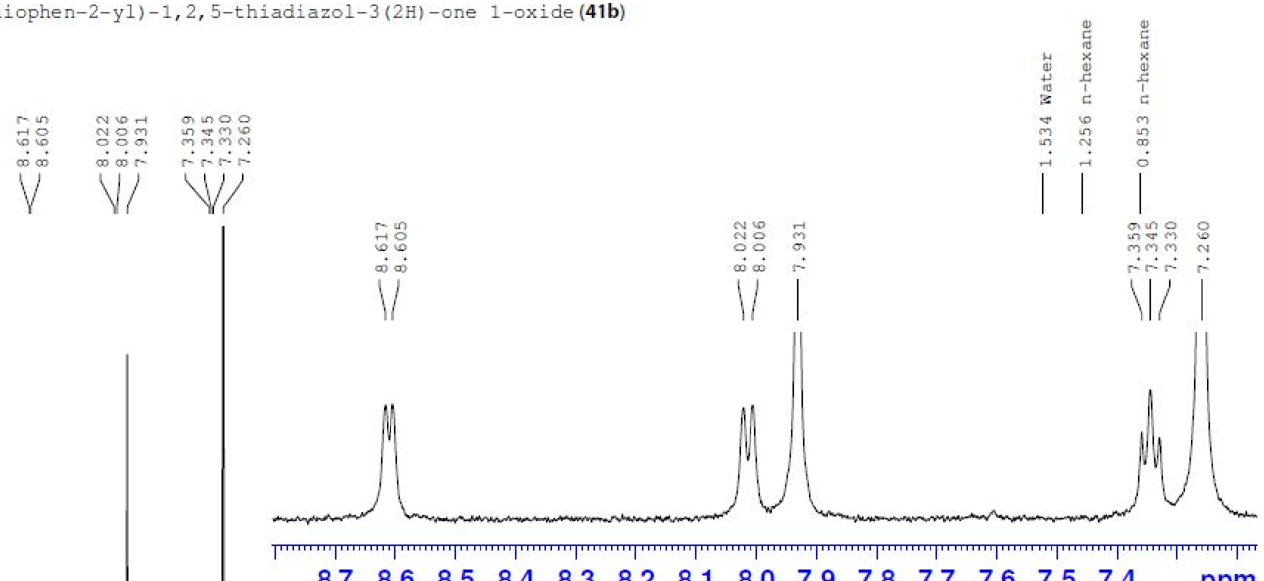

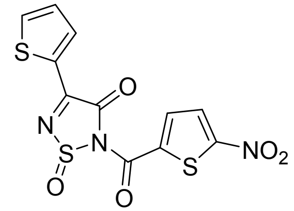

5ae

DMSO-d_6

100 MHz

2-(5-nitrothiophene-2-carbonyl)-4-(thiophen-2-yl)-1,2,5-thiadiazol-3(2H)-one 1-oxide(**41b**)

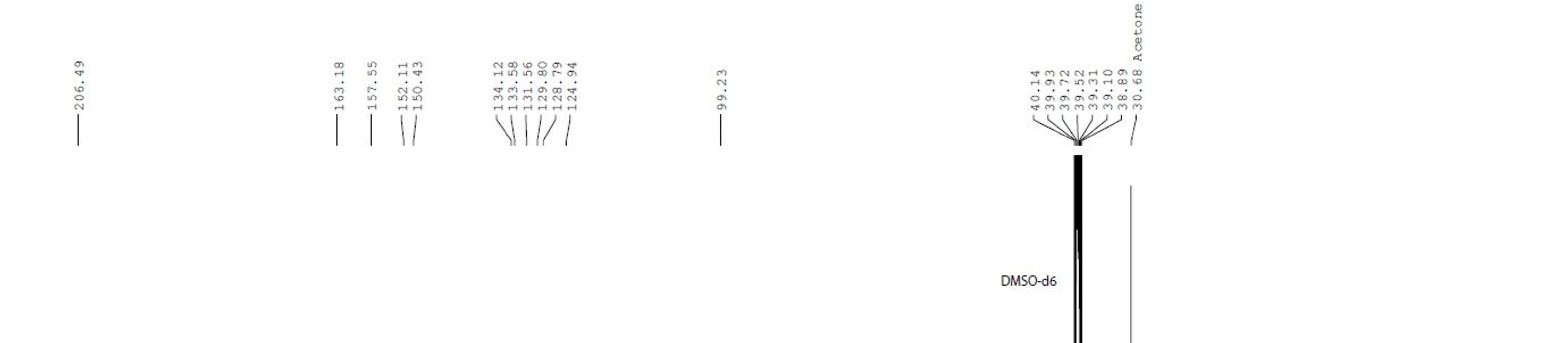

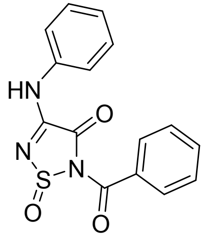

5af

CDCl$_3$

400 MHz

2-benzoyl-4-(phenylamino)-1,2,5-thiadiazol-3(2H)-one 1-oxide(**35b**)

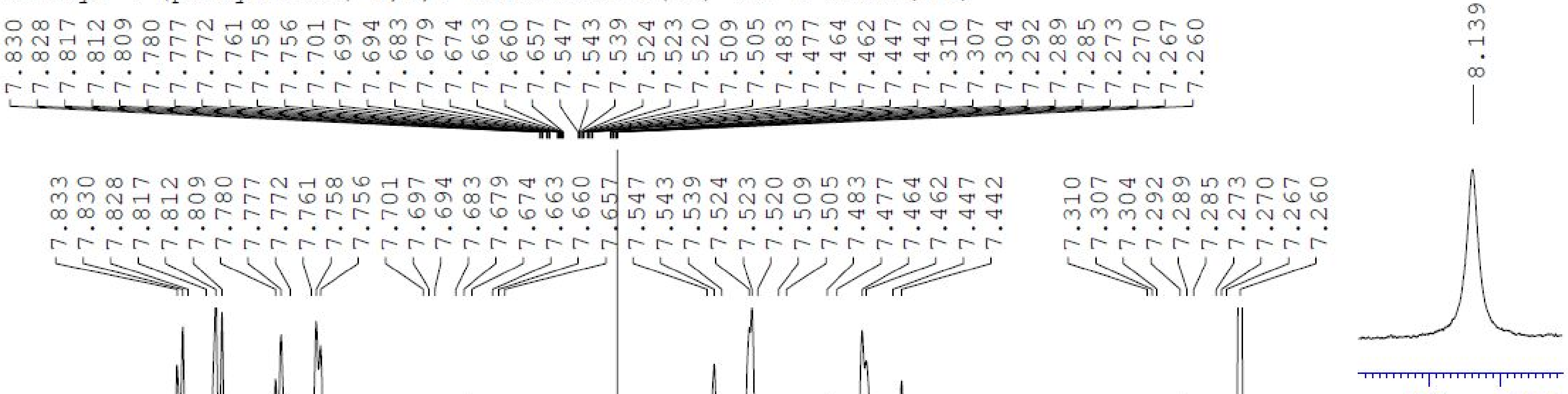

100 MHz

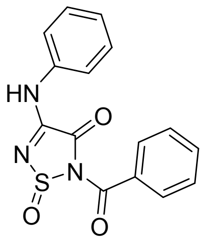

5af

CDCl$_3$

2-benzoyl-4-(phenylamino)-1,2,5-thiadiazol-3(2H)-one 1-oxide

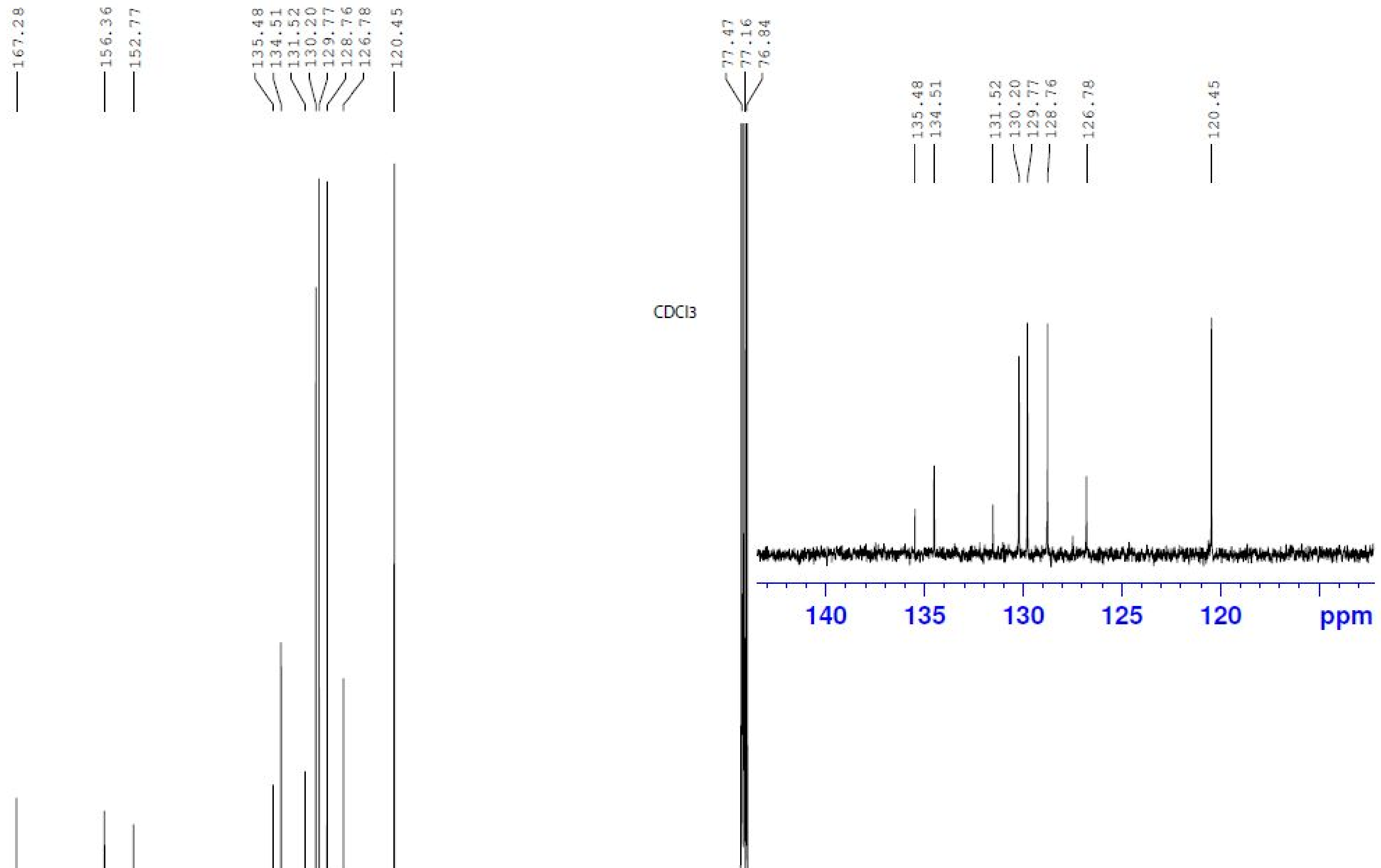

400 MHz

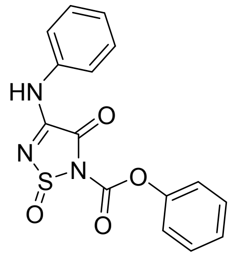

5ag

CDCl$_3$

phenyl 3-oxo-4-(phenylamino)-1,2,5-thiadiazole-2(3H)-carboxylate 1-oxide **(36b)**

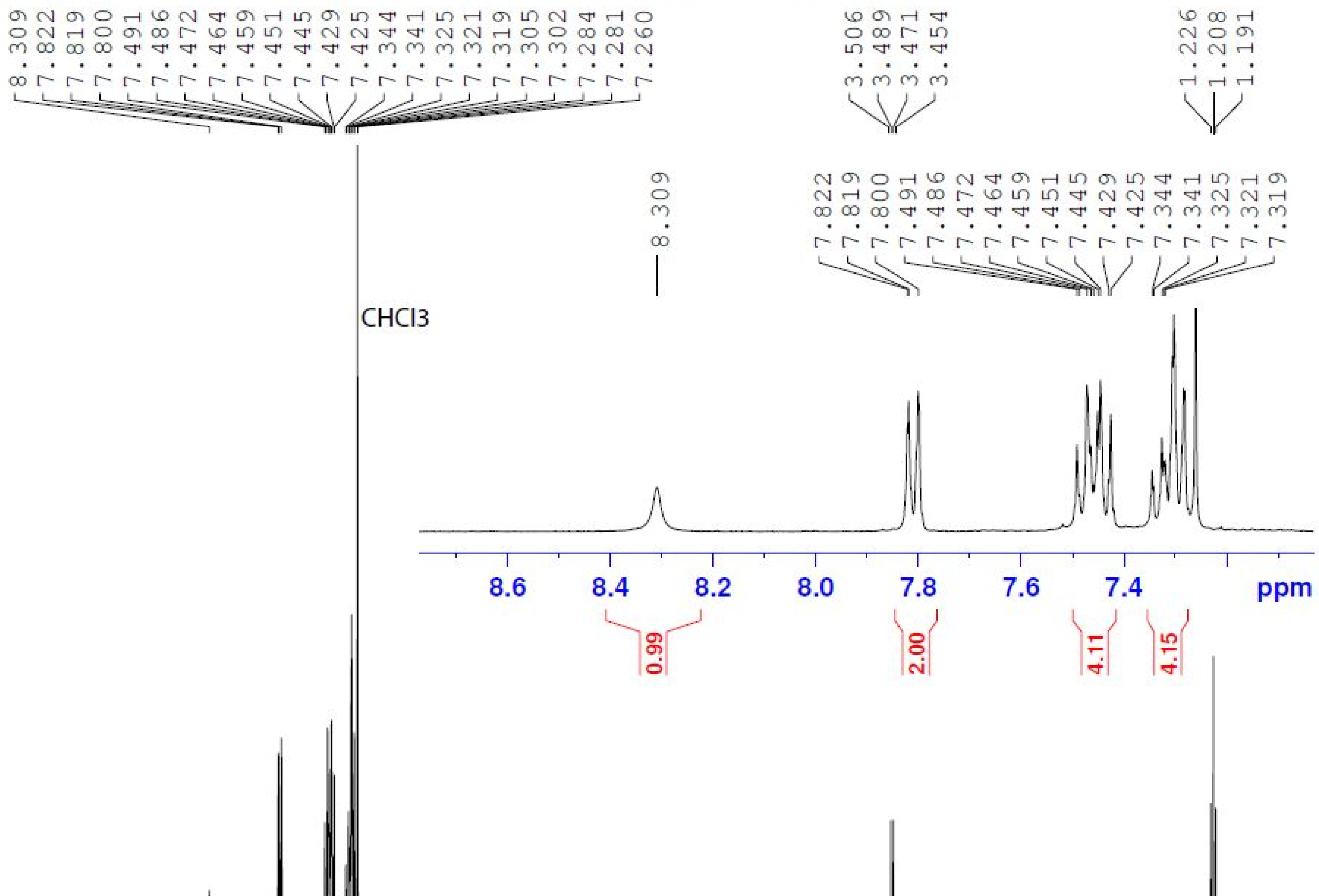

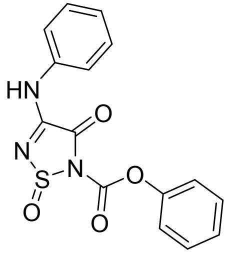

5ag

$CDCl_3$

100 MHz

phenyl 3-oxo-4-(phenylamino)-1,2,5-thiadiazole-2(3H)-carboxylate 1-oxide(**36b**)

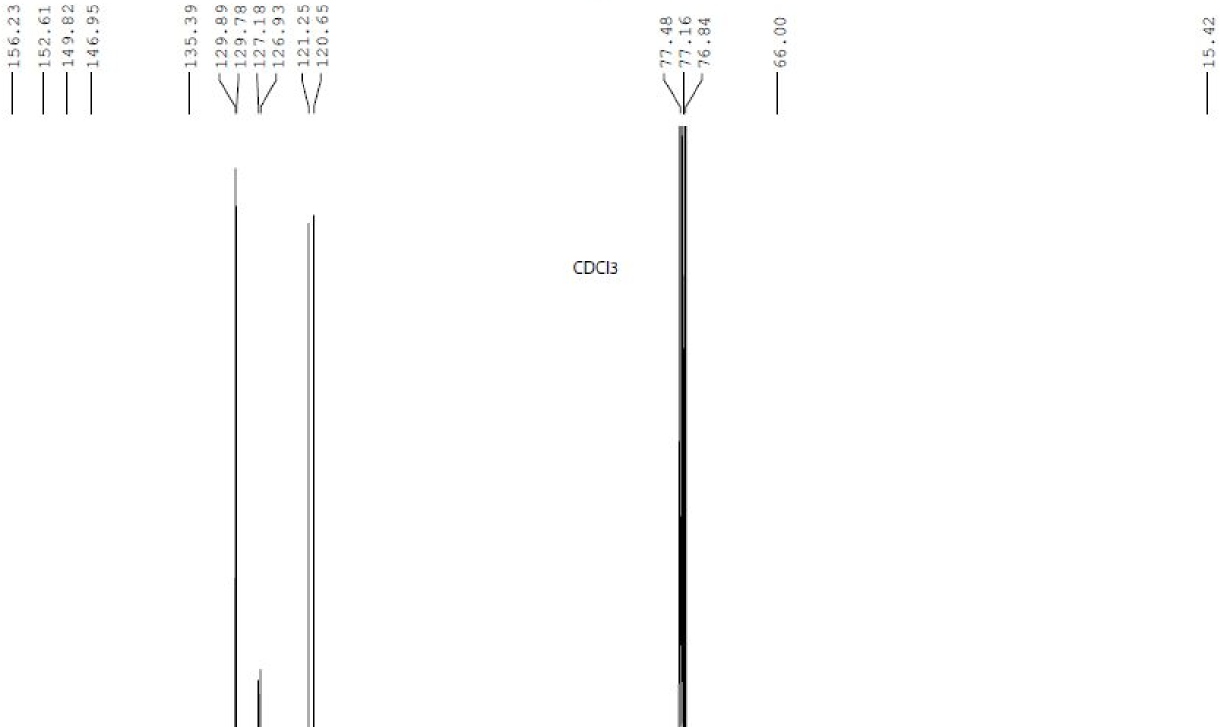

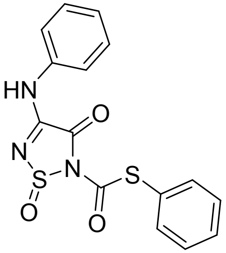

5ah

400 MHz

CDCl$_3$

S-phenyl 3-oxo-4-(phenylamino)-1,2,5-thiadiazole-2(3H)-carbothioate 1-oxide [illegible]

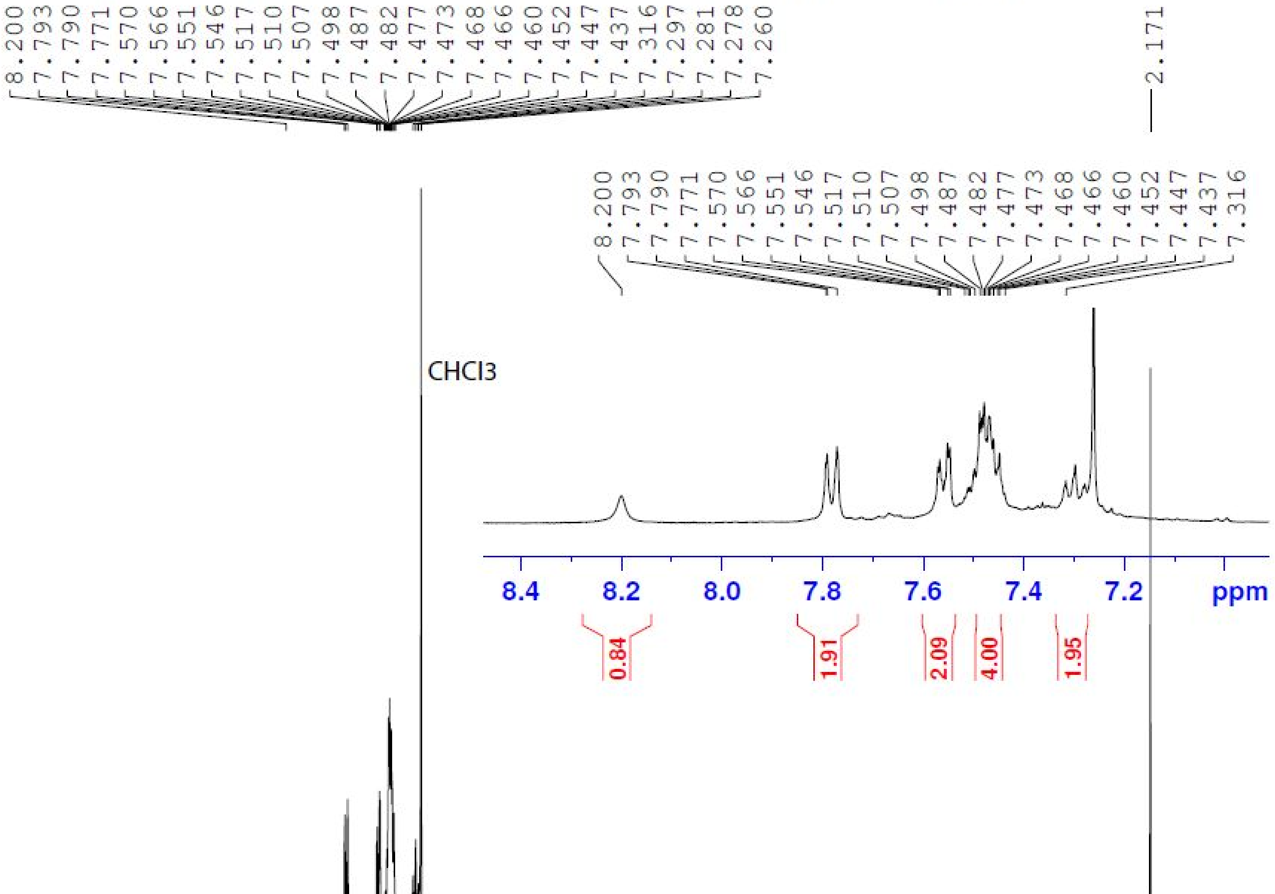

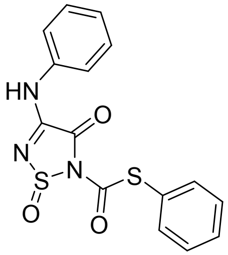

5ah

100 MHz

$CDCl_3$

S-phenyl 3-oxo-4-(phenylamino)-1,2,5-thiadiazole-2(3H)-carbothioate 1-oxide(37b)

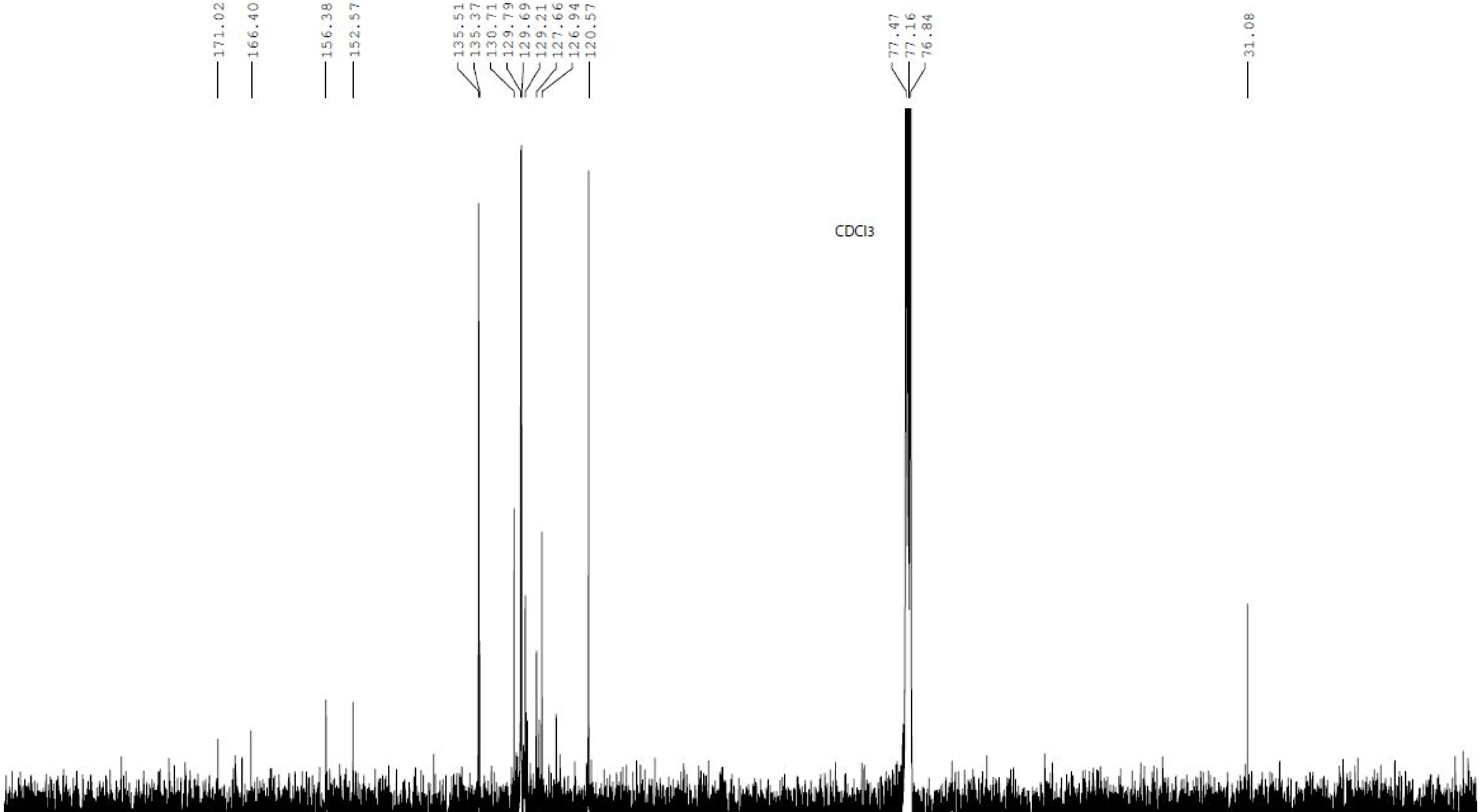

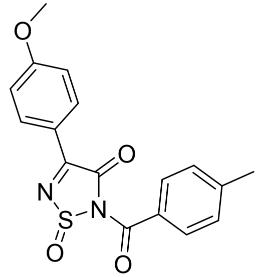

5ai (major)

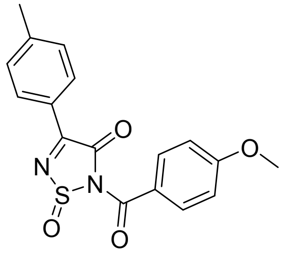

5al (minor)

CDCl$_3$

400 MHz

4-(4-methoxyphenyl)-2-(4-methylbenzoyl)-1,2,5-thiadiazol-3(2H)-one 1-oxide, major (**43b**)
and
2-(4-methoxybenzoyl)-4-(p-tolyl)-1,2,5-thiadiazol-3(2H)-one 1-oxide, minor (**43c**)

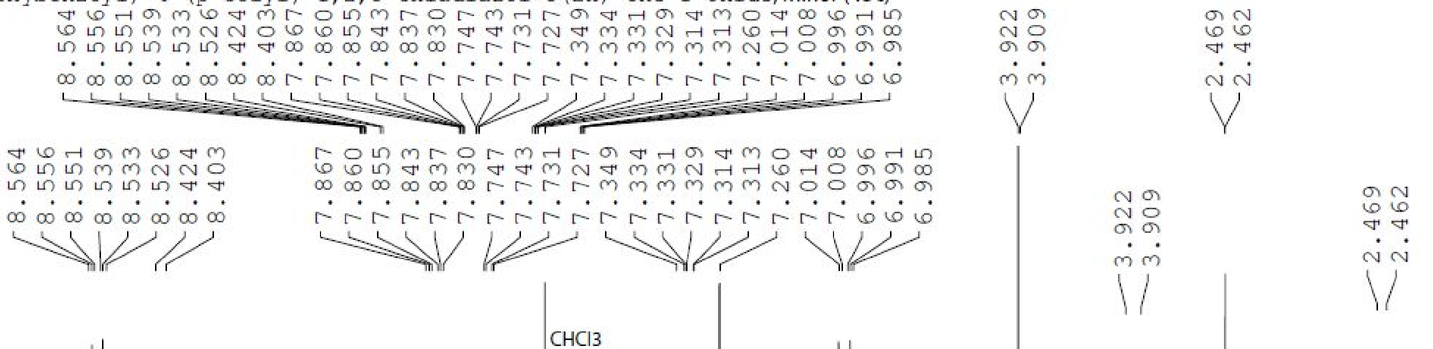

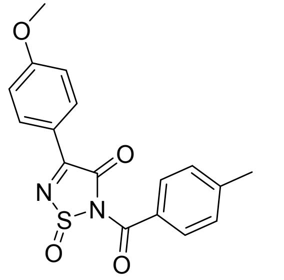

5ai (major)

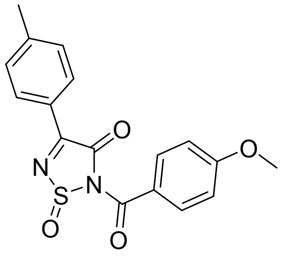

5al (minor)

CDCl$_3$

100 MHz

4-(4-methoxyphenyl)-2-(4-methylbenzoyl)-1,2,5-thiadiazol-3(2H)-one 1-oxide, major (**43b**)
and
2-(4-methoxybenzoyl)-4-(p-tolyl)-1,2,5-thiadiazol-3(2H)-one 1-oxide, minor (**43c**)

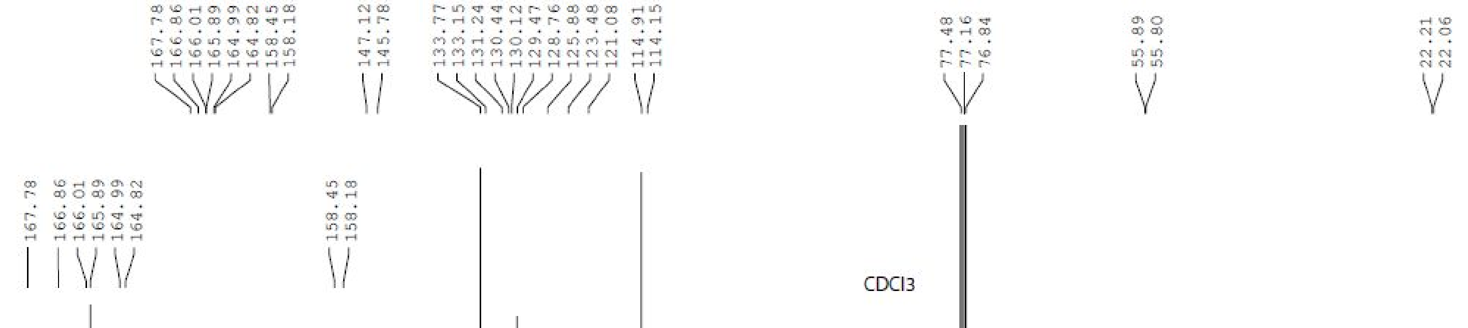

2-(2-Methoxybenzoyl)-4-(3-methoxyphenyl)-1,2,5-thiadiazol-3(2H)-one 1-oxide, major and 2-(3-methoxybenzoyl)-4-(2-methoxyphenyl)-1,2,5-thiadiazol-3(2H)-one 1-oxide, minor.

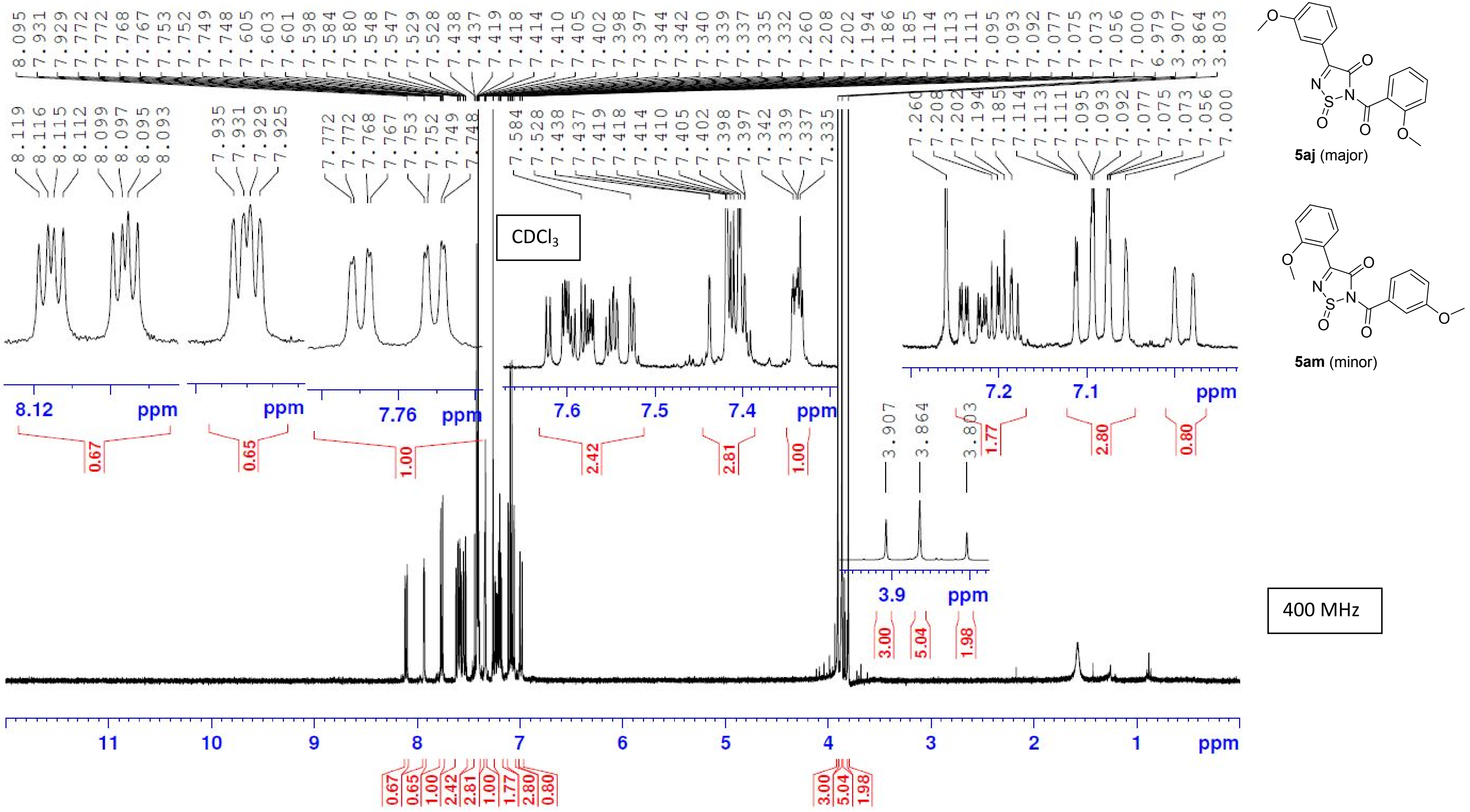

2-(2-Methoxybenzoyl)-4-(3-methoxyphenyl)-1,2,5-thiadiazol-3(2H)-one 1-oxide, major and
2-(3-methoxybenzoyl)-4-(2-methoxyphenyl)-1,2,5-thiadiazol-3(2H)-one 1-oxide, minor.

100 MHz

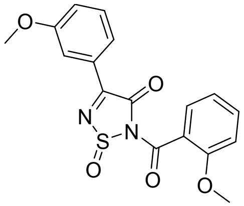

5aj (major)

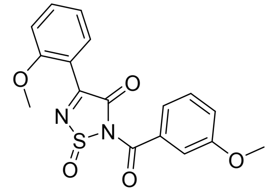

5am (minor)

CDCl$_3$

2-(4-methoxybenzoyl)-4-(3-methoxyphenyl)-1,2,5-thiadiazol-3(2H)-one 1-oxide, major (**44b**)
and
2-(3-methoxybenzoyl)-4-(4-methoxyphenyl)-1,2,5-thiadiazol-3(2H)-one 1-oxide, minor (**44c**)

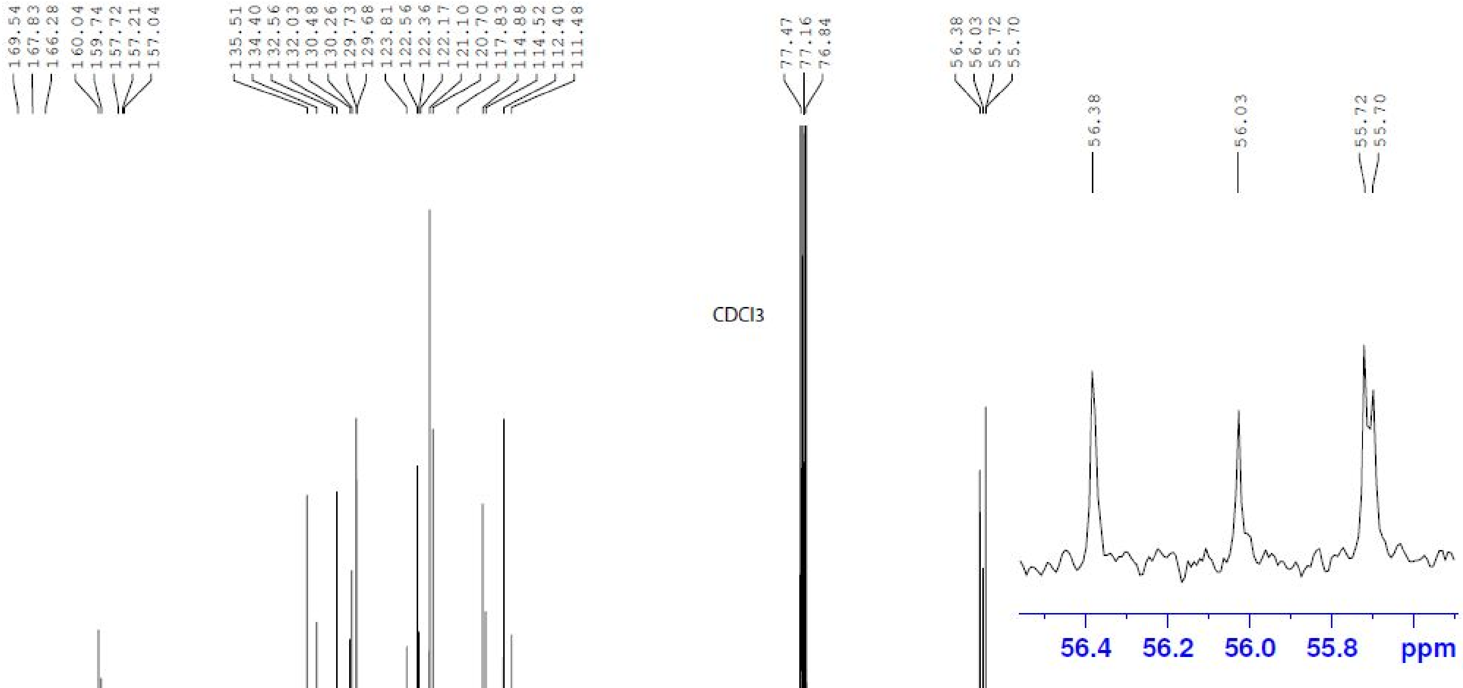

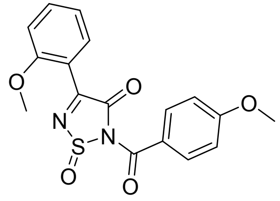

5ak (major)

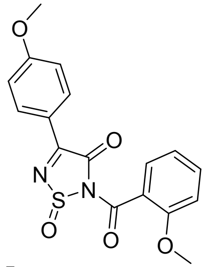

5an (minor)

CDCl$_3$

400 MHz

2-(4-methoxybenzoyl)-4-(2-methoxyphenyl)-1,2,5-thiadiazol-3(2H)-one 1-oxide, major (**45b**)
and
2-(2-methoxybenzoyl)-4-(4-methoxyphenyl)-1,2,5-thiadiazol-3(2H)-one 1-oxide, minor (**45c**)

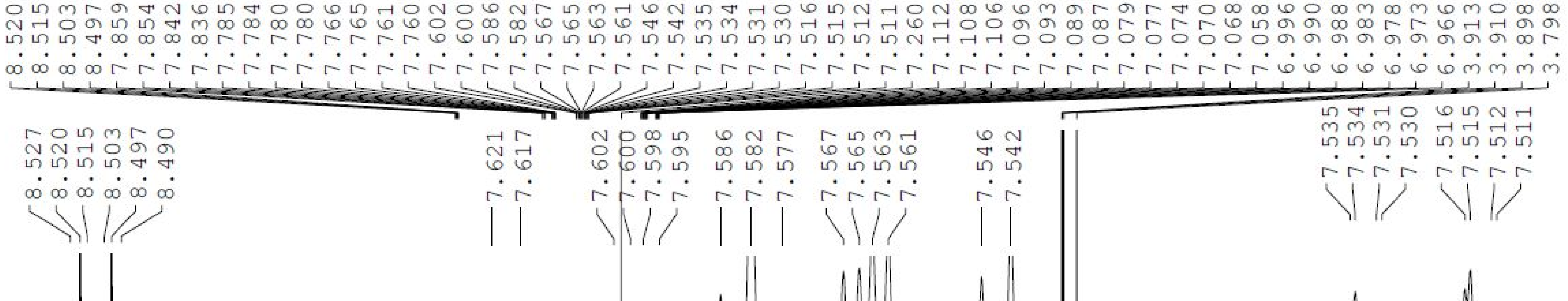

100 MHz

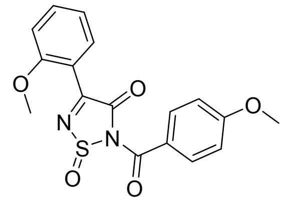

5ak (major)

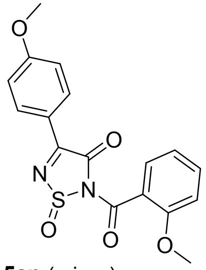

5an (minor)

CDCl$_3$

2-(4-methoxybenzoyl)-4-(2-methoxyphenyl)-1,2,5-thiadiazol-3(2H)-one 1-oxide, major (**45b**)
and
2-(2-methoxybenzoyl)-4-(4-methoxyphenyl)-1,2,5-thiadiazol-3(2H)-one 1-oxide, minor (**45c**)

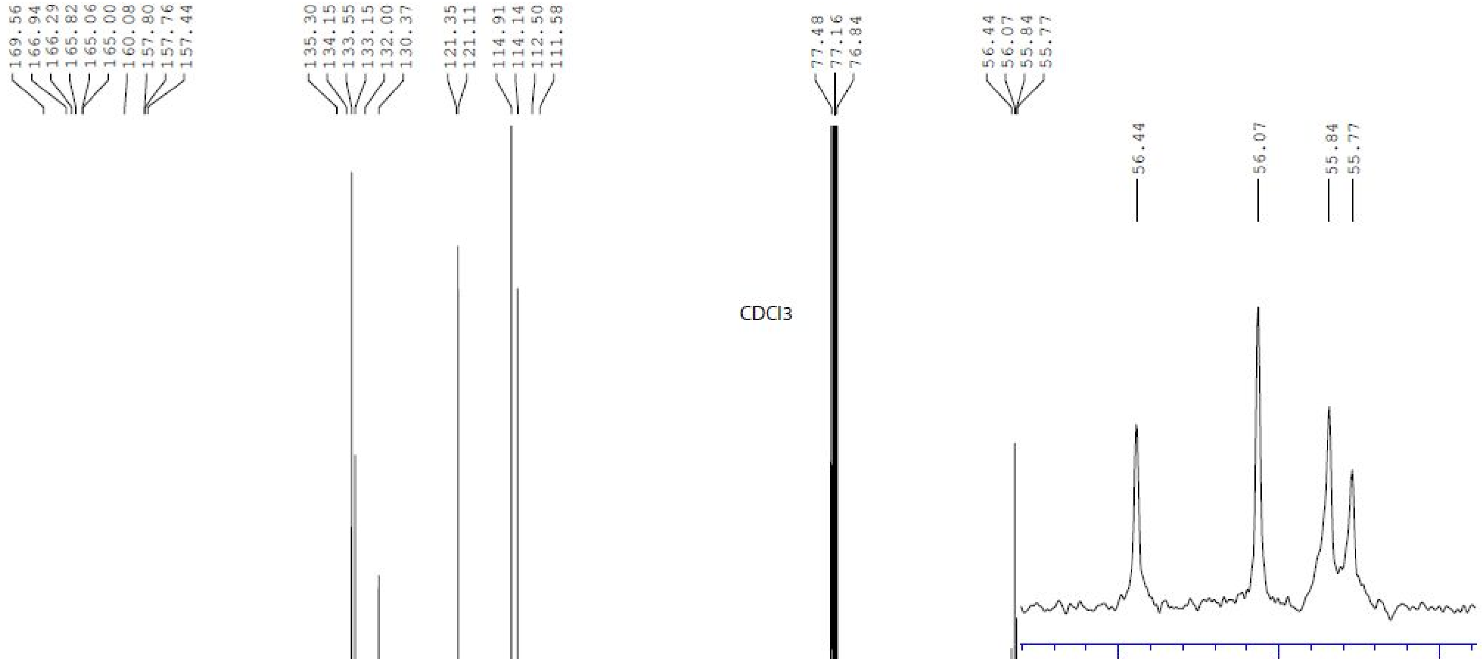

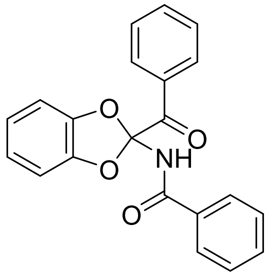

27

CDCl$_3$

400 MHz

N-(2-benzoylbenzo[d][1,3]dioxol-2-yl)benzamide

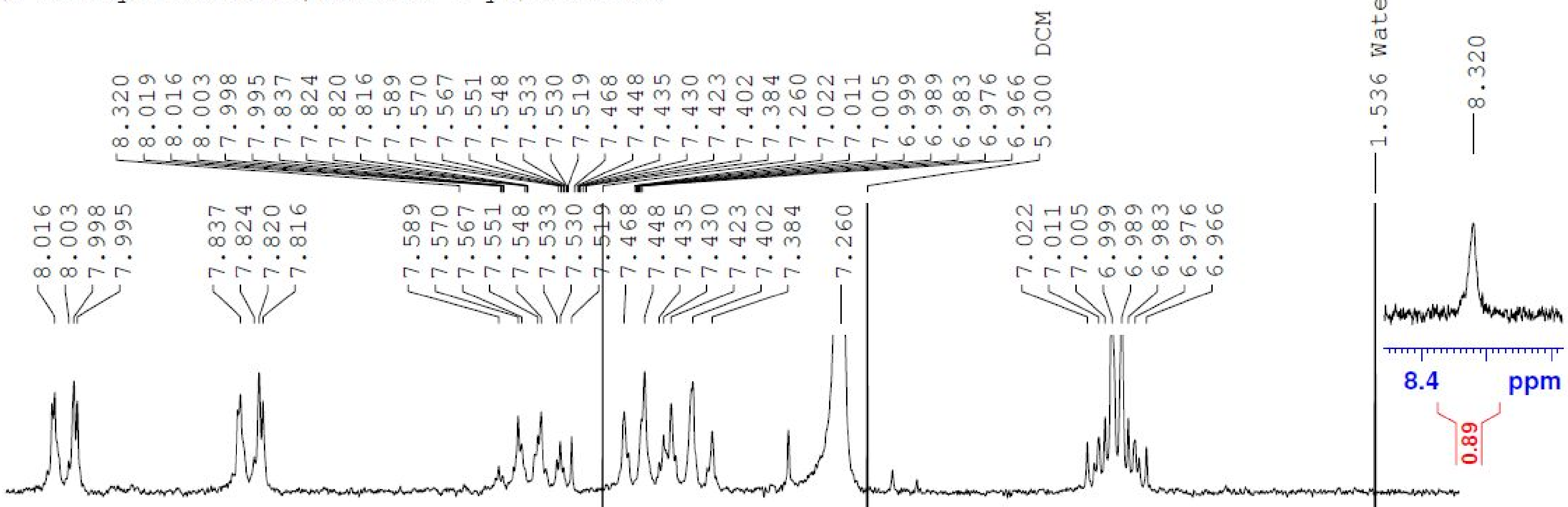

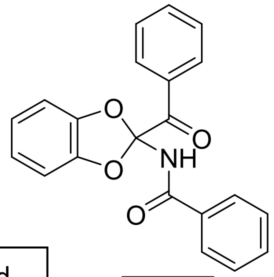

Acetone-d_6

27

100 MHz

N-(2-benzoylbenzo[d][1,3]dioxol-2-yl)benzamide (**9c**)

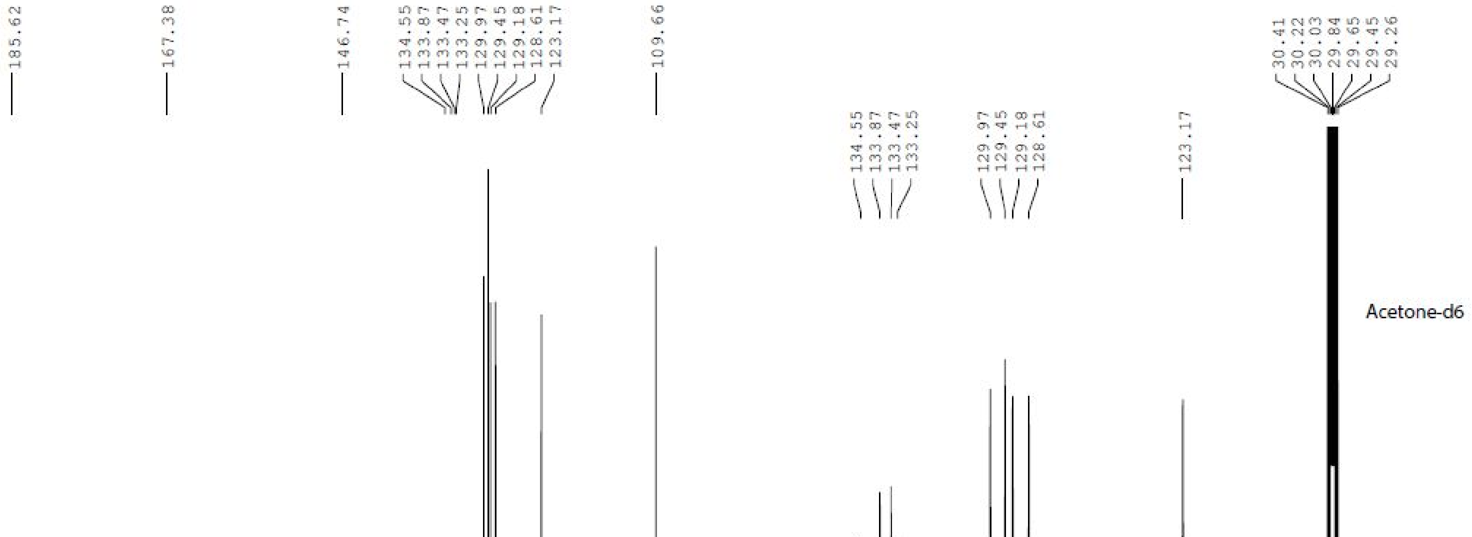

Supplement: Supplementary file 1 — ol3c02673_si_001.pdf [file ol3c02673_si_001.pdf]
